# The Discovery and Development of an Iridium‐Catalyzed N→C Alkyl Transfer Reaction

**DOI:** 10.1002/anie.202509193

**Published:** 2025-08-28

**Authors:** Erin C. Boddie, Phillippa Cooper, L. Anders Hammarback, Richard J. Mudd, Lyman J. Feron, John F. Bower

**Affiliations:** ^1^ Department of Chemistry University of Liverpool Crown Street Liverpool L69 7ZD UK; ^2^ School of Chemistry University of Bristol Bristol BS8 1TS UK; ^3^ Oncology R&D AstraZeneca The Discovery Centre Cambridge Biomedical Campus 1 Francis Crick Avenue Cambridge CB2 0AA UK

**Keywords:** C─H Activation, Enantioselective, Hydroamination, Hydroarylation, Transfer Alkylation

## Abstract

Under Ir‐catalyzed conditions, *N*‐2°‐alkyl or *N*‐3°‐alkyl substituents of diverse tertiary aryl amides can be exchanged with the *ortho*‐aryl C─H bond of the aromatic unit. These alkyl transfer processes employ a homochiral diphosphonite ligand, and this enforces notable levels of enantioconvergency for non‐stereodefined 2°‐alkyl substituents. Related processes allow the *intermolecular* transfer of *N*‐2°‐alkyl substituents, providing a convenient means of introducing difficult‐to‐install *ortho*‐alkyl units.

## Graphical abstract

Under Ir‐catalyzed conditions, *N*‐2°‐alkyl or *N*‐3°‐alkyl substituents of diverse tertiary aryl amides can be exchanged with the *ortho*‐aryl C─H bond of the aromatic unit. Related processes allow the *intermolecular* transfer of *N*‐2°‐alkyl substituents, providing a convenient means of introducing difficult‐to‐install *ortho*‐alkyl units.

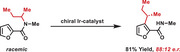

The appendage of C(sp^3^) units to nucleophilic centers is typically achieved using highly electrophilic alkyl halides (Scheme 1, Equation 1).^[^ ^1^ ^]^ Less reactive and more tractable electrophiles can be engaged under metal‐catalyzed conditions. Most prominently, hydrogen borrowing methodologies allow readily available alcohols (X = O) or amines (X = NR) to function as alkylating agents via a dehydrogenation‐condensation‐hydrogenation sequence (Equation 2).^[^ ^2^, ^3^, ^4^ ^]^ These heteroatom→carbon alkyl transfer processes require relatively nucleophilic reaction partners and are triggered by metal‐catalyzed activation of the α‐C─H bond of the proelectrophile. In this manuscript we outline an alternative metal‐catalyzed framework for achieving N→C alkyl transfer reactions that does not involve a condensation event and instead employs a metal catalyst to activate both reaction partners (Equation 3). This apparently involves sequential β‐amino elimination^[^ ^5^, ^6^ ^]^ and branched selective alkene hydroarylation events,^[^ ^7^, ^8^, ^9^ ^]^ wherein the activation of a β‐amino C(sp^3^)─H bond and an aryl C(sp^2^)─H bond occur, respectively. The N─C bond breaking and C─C bond forming sequence allows intra‐ and intermolecular alkyl transfers to low nucleophilicity aromatic units, and can also be used to execute conceptually powerful enantioconvergent processes. The observations described herein are unusual and set the stage for the design of numerous related processes.

**Scheme 1:**
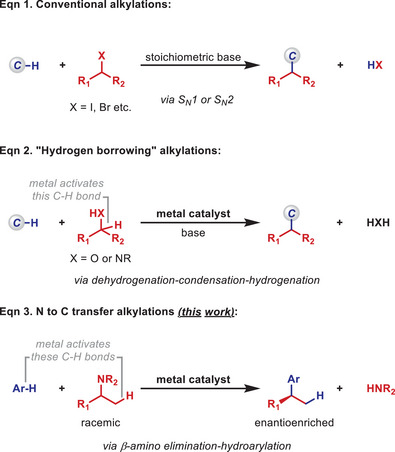
Introduction.

We have previously reported a range of Ir‐catalyzed alkene hydroarylation reactions that are initiated by carbonyl‐directed C(sp^2^)─H activation of the aromatic partner, and these processes are notable for their prescriptive ligand requirements.^[^ ^7^, ^8^, ^9^ ^]^ During efforts to extend the scope to amide‐directed alkene hydroheteroarylation reactions, we explored the cross‐coupling of **1a** with styrene (Scheme 2a). Using an Ir(I)‐system modified with specially designed SPINOL‐based diphosphonite **L‐4**,^[^ ^10^ ^]^ target **2** was formed in 85% yield and with complete branched selectivity. Unexpectedly, we also observed traces (3% yield) of **3**, where dealkylation of one the N‐*i*‐Pr groups had occurred alongside the expected alkene hydroheteroarylation process. As a control experiment, we exposed **1a** to the same reaction conditions, but in the absence of styrene. Remarkably, in this experiment, transfer of an N‐*i*‐Pr unit to C3 of the furan moiety occurred to give **4a** in 45% yield. Subsequently, studies were undertaken to optimize this process (Scheme 2b). Initially, these efforts focussed on identifying an optimal ligand system. A wide range of commercially available or “in house” P,P‐ligands were evaluated, but the only promising systems were those related to **L‐4**, with **L‐2** (R^1^ = Br) and **L‐3** (R^1^ = 2‐naphthyl) offering comparable levels of efficiency (further optimization studies are detailed in the SI). Using **L‐4,** we optimized the reaction solvent, temperature, time and pre‐catalyst counterion leading to the conditions outlined in Entry 12, which deliver **4a** in 95% isolated yield in 1,2‐dichlorobenzene (1,2‐DCB). During the course of this study, parent SPINOL became commercially available and this allowed easy access to **L‐5** (R^1^ = H). Evaluation of this ligand on a subset of substrates, revealed that it is effective in certain cases, but does not offer the generality of **L‐4** (see the Supporting Information). Thus, the presence of an appropriate R^1^ substituent is important for optimal efficiencies.

**Scheme 2:**
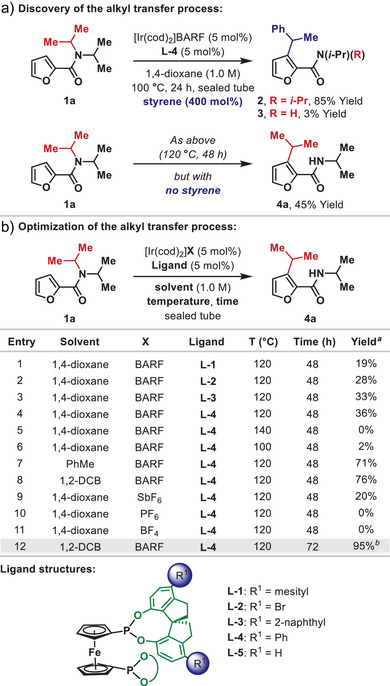
Discovery and Optimization of the Alkyl Transfer Process. ^a)^Yield determined by ^1^H NMR analysis of crude material against an internal standard. ^b)^Isolated yield.

To explore scope, we evaluated optimized conditions initially in processes involving the transfer of isopropyl units (Table 1a). As can be seen, the spectator N‐alkyl unit (R^2^) can be a primary or secondary alkyl group (**4b‐d**); in all cases, only transfer of the isopropyl unit was observed. Secondary amides (e.g. **NH‐1a**) are not suitable substrates, and do not undergo alkyl transfer to give primary amide products. The protocol tolerates additional substitution on the furan unit (**4f‐j**), although diminished yields were observed for **4h** and **4i**, possibly due to competing Ir‐insertion into the C‐halogen bonds. Other types of heteroaromatic cores can also be employed, including pyrroles (**4k**), thiophenes (**4l**), and benzofused systems (**4m** and **4n**). Efficient isopropyl transfer onto a benzenoid ring was also observed to generate **4o** in 67% yield.^[^ ^11^ ^]^ The selective alkyl transfers observed for **4b‐e** suggested that other types of α‐methylated secondary alkyl substituents should participate. Gratifyingly, exposure of *s*‐butyl system **1p** to optimized conditions delivered target **4p** in 81% yield (Table 1b). Notably, **4p** was formed in 88:12 e.r. from racemic **1p**, which shows that the homochirality of **L‐4** is able to effect stereoconvergency during the alkyl transfer process (vide infra).^[^ ^12^, ^13^, ^14^ ^]^ Similar stereoconvergencies were obtained for **4q‐t**, which involve the transfer of higher secondary alkyl units. Transfer of tertiary alkyl units is also possible as demonstrated by *t*‐butyl transfer to give **4u** in 95% yield. A similar result was obtained from **1e**, where **4e’** was formed selectively in 62% yield, and **4e** (see Table 1a), was not observed. Overall, the key requirements for the process are a) that the substituents on the transferring group must all be alkyl, b) one of them must be a methyl, and c) the transferring group must be secondary or tertiary. Accordingly, no reaction was observed when **1v** and **1w** were exposed to optimized conditions, whereas **1x** led to partial decomposition alongside 65% recovered starting material (Table 1c). Other representative limitations are outlined in the Supporting Information.

**Table 1:** Scope of the Intramolecular Reaction. О = C‐center that was attached to the amide nitrogen in the starting material.

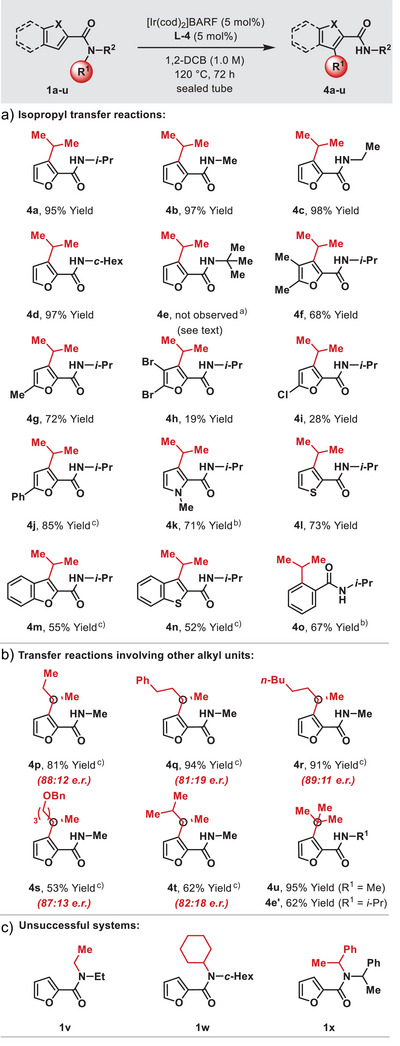

^a)^Selective transfer of the t‐butyl group occurred instead to give **4e’** in 62% yield (see b).

^b)^The reaction was run at 130 °C.

^c)^[Ir(cod)_2_]BARF (10 mol%) and **L‐4** (10 mol%) were used.

Preliminary investigations have been undertaken to elucidate key mechanistic features of the process. When ***deuterio*‐1a**, in which one of the isopropyl groups is labelled, was exposed to optimized conditions, preferential transfer of the non‐deuterated isopropyl group occurred (∼2:1 selectivity) to give ***deuterio*‐4a** (Scheme 3a). Here, partial scrambling of the labels between the methyl groups and the methine positions was also observed. An analogous experiment using ***deuterio*‐1a’**, led to transfer of the C3‐deuteration to the methyl groups of the migrating isopropyl unit only. Collectively, these results are consistent with a C─N cleavage pathway proceeding via a sequence of amide‐directed β‐C(sp^3^)─H activation^[^ ^15^, ^16^, ^17^ ^]^ and β‐amino elimination (**1a** to **II** to **III**) (Scheme 3b).^[^ ^18^ ^]^ Preliminary DFT studies using a simplified ligand system support the viability of the conversion of **I** to **II** to **III** (see the Supporting Information).^[^ ^19^ ^]^ Amongst several possibilities, reversible β‐hydride elimination from an α‐C(sp^3^)─H oxidative addition derived intermediate (not depicted) could account for the methyl to methine deuterium transfer observed in the N‐isopropyl unit of ***deuterio*‐4a**.^[^ ^20^, ^21^ ^]^ N‐H reductive elimination from **III** then provides **V** and propylene, which are converted to **4a** via our previously proposed branched selective alkene hydroarylation mechanism.^[^ ^7^, ^9^, ^22^, ^23^, ^24^, ^25^ ^]^ The viability of this latter sequence is supported by the conversion of **NH‐1b** and alkene **5** to **4q** under optimized reaction conditions (Scheme 3c). The alkyl transfer conditions use sealed tubes: when **1a** was exposed to the reaction conditions in an open vessel, target **4a** was not observed, and only mono‐N‐dealkylation occurred to provide **NH‐1a**. We attribute this to evaporation of propylene after dissociation from **III**. The enantioconvergent processes in Table 1b provide further evidence for alkene dissociation from **III**, because these require a mechanism that ablates preexisting stereochemical information, thereby allowing the catalyst to selectively functionalize one of the two prochiral faces of the alkene.^[^ ^26^ ^]^

**Scheme 3:**
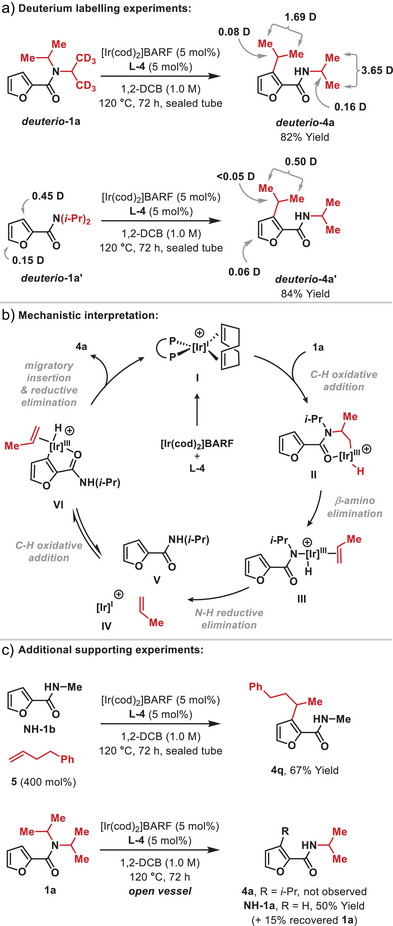
Mechanistic Studies.

β‐Amino‐elimination involving an Ir‐center has been reported previously within the context of stoichiometric studies on alkene migratory insertion into Ir─N bonds.^[^ ^5^, ^6^ ^]^ Compared to this prior work, the conversion of **1a** to **V** is unusual because the process is instead initiated by C─H activation. Indeed, we are unaware of any related examples of C─H activation triggered β‐amino eliminations under Ir‐catalyzed conditions. To the best of our knowledge, even the seemingly simple selective N‐dealkylation of **1a** to **NH‐1a** in Scheme 3c is unique. Interestingly, the conversion to **1a** to **V** is essentially the reverse of Hartwig and co‐workers’ Ir‐catalyzed alkene hydroamination reaction, with key points of difference being the use of a distinct P,P‐ligand system and a cationic rather than neutral Ir‐pre‐catalyst.^[^ ^6^ ^]^ We suggest that the structural, electronic and steric requirements of the transferring alkyl group (see Table 1c and the earlier text) reflect the need i) to facilitate β‐C(sp^3^)─H oxidative addition (methyl groups are expected to be preferred for this), ii) to suppress α‐C(sp^3^)─H oxidative addition and iii) to suppress competing β‐hydride elimination from **II** to allow β‐amino elimination.

It should be noted that we cannot fully discount other pathways for the N‐dealkylation process. For example, we have considered the possibility that N─C cleavage occurs via a “hidden” (Lewis‐)acid promoted S_N_1 ionization pathway;^[^ ^27^, ^28^, ^29^, ^30^, ^31^, ^32^ ^]^ however, several observations are inconsistent with this, and supporting experiments are detailed in the SI. Most significantly: 1) exposure of ***deuterio*‐1a** to neat MsOH results in mono‐isopropyl loss to give ***deuterio*‐NH‐1a** with a KIE of ∼1, which is much lower than the isotope effect observed in Scheme 3a for ***deuterio*‐1a** to ***deuterio*‐4a**; 2) MsOH promoted N‐dealkylation of *N*,*N*‐dicyclohexyl system **1w** is approximately two times faster than that of *N*,*N‐*diisopropyl amide **1a**, yet only the latter reacts under iridium‐catalyzed conditions; 3) under Ir‐catalyzed conditions, selective transfer of the isopropyl group of **1d** is observed to give **4d** in high yield, yet under MsOH promoted N‐dealkylation conditions, the cyclohexyl unit is cleaved preferentially (∼4:1 selectivity); 4) exposure of **1a** to MsOH (10 mol%) or TfOH (10 mol%) in 1,2‐DCB at 120 °C for 72 h gives >90% recovery of starting material, with no signs of N‐dealkylation. Thermally promoted (retro‐ene‐type) N‐dealkylation is discounted because such processes require extreme conditions for secondary alkyl groups;^[^ ^33^ ^]^ indeed, **1l** was recovered intact after being heated at 140 °C in 1,2‐DCB for 24 h. The conditions for the alkyl transfer process are very similar to those used in our recently reported alkene hydroarylation method,^[^ ^9^ ^]^ where, with the exception of the result in Scheme 2a, N,N‐diisopropyl groups remained intact. Thus, C─N cleavage only occurs at significant levels when other productive Ir‐catalyzed pathways (i.e., hydroarylation of an exogenous alkene) are absent.

The suggestion that the alkene does not remain bound to the iridium center after the N─H reductive elimination step is supported further by the result outlined in Scheme 4a, where crossover of an isopropyl group from **1y** was observed to give **N‐*i*‐Pr‐4y** (19% yield), in addition to expected product **4y** (11% yield) and dealkylated side product **NH‐1y** (23% yield). In combination with the mechanistic analysis in Scheme 3b, this result suggested that *intermolecular* alkyl transfer processes might be viable. To this end we explored the viability of using a variety of distinct alkyl transfer reagents for the conversion of **1v** to **4v**, from which selected examples are shown in Scheme 4b (further details are given in the Supporting Information). Of these, only N‐acetyl systems **6a** and **6b** provided appreciable conversions to **4v**, which was formed in 19% and 21% yield, respectively.^[^ ^34^ ^]^ Due to ease of synthesis, reagent **6a** was selected for further optimization studies, the most productive of which involved the evaluation of secondary amide directing groups to mimic the intermediacy of **V** in Scheme 3b. Based on this, we evaluated isopropyl transfer from **6a** to **NH‐1a** (R^1 ^= *i*‐Pr), and, remarkably, this provided **4a** in 72% yield (Scheme 4c). Efficiencies that exceed the intramolecular protocol in Table 1 were observed for **4g**, **4i**, **4m** and **4o**,^[^ ^11^ ^]^ and a variety of other systems (**4z‐4bb**) could also be accessed. This intermolecular protocol is especially suitable for the transfer of isopropyl groups, although, as outlined in Scheme 4d, a *s*‐butyl group could also be transferred to give **4z’** in 91% yield.^[^ ^35^ ^]^ At the current level of development, efforts to transfer higher or more functionalized alkyl units have so far been largely unsuccessful, and this aspect is a focus of ongoing studies in our laboratory.

**Scheme 4:**
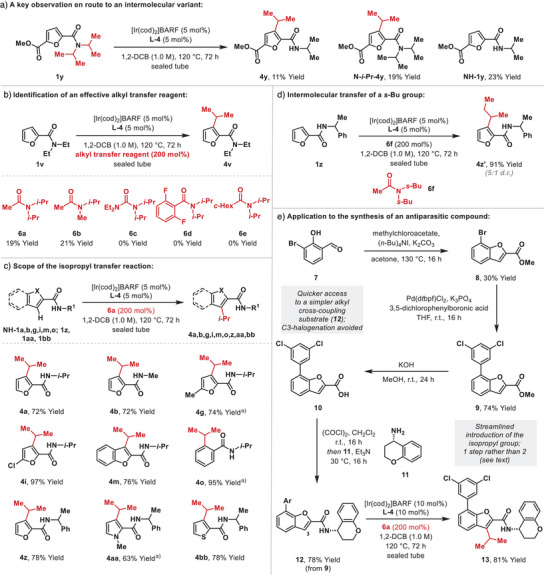
Development of an Intermolecular Variant. ^a)^[Ir(cod)_2_]BARF (10 mol%) and **L‐4** (10 mol%) were used.

The ability to transfer isopropyl groups intermolecularly is of synthetic interest, because the alternate alkene hydroarylation approach (cf. Scheme 3c) requires the handling of gaseous propylene, and conventional cross‐coupling strategies are often challenging. To provide a point of comparison to the latter, we have applied the method to the synthesis of antiparasitic compound **13** (Scheme 4e).^[^ ^36^ ^]^ A three step (unoptimized) synthesis of carboxylic acid **10** was devised from aldehyde **7**. Amide coupling with known amine **11** ^[^ ^37^ ^]^ provided catalysis substrate **12** in 78% yield. Exposure of this to optimized conditions in the presence of alkyl transfer reagent **6a** generated target **13** in 81% yield.^[^ ^38^ ^]^ Notably, the N‐substituent and the aryl chlorides of **12** remained intact during this process. Compared to the reported route, our synthesis of **13** is three steps shorter because the alkyl transfer process a) does not require prefunctionalization at C3 of the benzofuran and b) allows the direct installation of the isopropyl unit. With respect to the latter, the previous synthesis of **13** employed a conventional Suzuki cross‐coupling approach, wherein initial installation of an isopropenyl group was followed by a discrete alkene reduction step. The present method is more direct and does not require the preparation of an isopropenyl boronic acid reagent.

In summary, we describe the discovery and development of unique iridium‐catalyzed N→C(sp^2^) alkyl transfer reactions. As outlined in the introduction, the processes are distinct from prevailing alkylation methods, in part, because C─H activation of both reaction partners occurs en route to the target. Although β‐amino elimination involving iridium‐centers has been described previously,^[^ ^5^ ^]^ the current processes are noteworthy in a) triggering this step via an initial C─H activation event, and b) in integrating it into a productive process. Indeed, the initial stages of the mechanism are essentially the reverse of previously reported alkene hydroamination reactions,^[^ ^6^ ^]^ and this then allows C─C bond formation to occur via C─H activation triggered alkene hydroarylation. Thus, the processes could be termed as “retrohydroamination‐hydroarylation” transfer reactions, where, unusually, C─N bond breaking retro‐migratory insertion and C─C bond forming migratory insertion are combined in a productive manner. The most powerful variants developed so far are the enantioconvergent processes in Table 1b and the intermolecular processes in Scheme 4. The latter uses N‐alkyl amides as tractable surrogates for gaseous alkenes, and one could envisage developing related processes that allow the introduction of other low molecular weight fragments. For example, the transfer of fluorinated alkyl groups would be desirable, and this would be most attractive if β‐N‐elimination could be replaced by β‐O‐elimination, because then the alkyl transfer reagent could be accessed from readily available fluorinated alcohols. Beyond the synthetic advances, the present study is also instructive from the viewpoint of research project development. Notably, the crucial, yet easy to overlook observation that a side product (**3**) formed in 3% yield led to a prototype methodology that a) is interesting from a reactivity viewpoint, and b) addresses issues of synthetic significance.

## Conflict of Interests

The authors declare no conflict of interest.

## Supporting information



Supporting Information

## Data Availability

The data that support the findings of this study are available in the Supporting Information of this article.
