# Supplementary material for: The Discovery and Development of an Iridium‐Catalyzed N→C Alkyl Transfer Reaction
Source: Angew Chem Int Ed Engl. 2025 Aug 28;64(40):e202509193. doi: 10.1002/anie.202509193 (PMC12462736; doi:10.1002/anie.202509193)
Supplement: Supplementary file 1 — Supporting Information [file ANIE-64-e202509193-s001.pdf]

The Discovery and Development of an Iridium-Catalyzed N→C Alkyl Transfer Reaction

Erin C. Boddie,† Phillippa Cooper,‡ L. Anders Hammarback,† Richard, J. Mudd,† Lyman. J. Feron,§ and John F. Bower*,†

† Department of Chemistry, University of Liverpool, Crown Street, Liverpool, L69 7ZD, United Kingdom

‡ School of Chemistry, University of Bristol, Bristol, BS8 1TS, United Kingdom

§ Medicinal Chemistry, Oncology, IMED Biotech Unit, AstraZeneca, Cambridge, United Kingdom

Supporting Information

Table of Contents

General Information 2

Discovery of the Alkyl Transfer Process (Scheme 2A) 4

SPINOL Ligand Synthesis (Scheme 2B) 6

Full Optimization of Intramolecular Transfer (Scheme 2B) 11

Intramolecular Isopropyl N→C Alkyl Transfer Reactions: Substrates (Table 1A) 13

Intramolecular Isopropyl N→C Alkyl Transfer Reactions: Products (Table 1A) 20

Intramolecular N→C Alkyl Transfer Involving Other Alkyl Units: Substrates (Table 1B) 26

Intramolecular N→C Alkyl Transfer Involving Other Alkyl Units: Products (Table 1B) 32

Evaluation of **L-5** (Derived from Commercially Available SPINOL) 38

Unsuccessful Systems (Table 1C) 39

Mechanistic Studies (Scheme 3A) 41

A Key Observation: Intermolecular Variant (Scheme 4A) 43

Identification of an Effective Alkyl Transfer Reagent (Scheme 4B) 45

Alternative Transfer Reagents Screened with 1v 48

Intermolecular N→C Isopropyl Transfer: Substrates (Scheme 4C) 49

Intermolecular N→C Isopropyl Transfer: Products (Scheme 4C) 52

Intermolecular N→C *sec*-Butyl Transfer (Scheme 4D) 54

Application to the Synthesis of an Antiparasitic Compound (Scheme 4E) 55

Computational Chemistry 61

Control experiments to probe the possibility of "hidden" acid promoted N-dealkylation 80

References 84

NMR Spectra for Novel Compounds 86

General Information

All materials for which a synthetic route is not described or referenced were purchased from commercial sources. All reagents requiring purification were purified using standard laboratory techniques according to methods published by Perrin, Armarego, and Perrin (Pergamon Press, 1966). Catalytic reactions were carried out in flame-dried Young-type resealable tubes. All catalysis reactions were performed using pre-dried solvents unless stated otherwise. Anhydrous solvents were obtained by distillation using standard procedures or from passage through Innovative Technology PureSolv MD system. Et_3N was distilled over CaH_2 and stored over activated 4Å molecular sieves under N_2. Styrene was distilled by Hickman distillation under N_2. The removal of solvents *in vacuo* was achieved employing rotary evaporators connected with diaphragm pumps. Materials were then dried on a high-vacuum line prior to analysis. Flash column chromatography (FCC) was performed using silica gel (Aldrich 40-63 μm, 230-400 mesh). Ferrocene-SPINOL ligands were purified by FCC on deactivated silica gel (stirred for 16 h with 10% w/v Et_3N). Thin layer chromatography (TLC) was performed using aluminum backed 60 F254 silica plates. Visualization was achieved using UV fluorescence, a basic $KMnO_4$ solution and heat or a phosphomolybdic acid solution and heat. Proton nuclear magnetic resonance (NMR) spectra were recorded on a Bruker DRX 500 Cryoprobe or a Bruker Avance 400. ^{1}H NMR spectra were recorded at 400 MHz or 500 MHz as stated. ^{13}C NMR spectra were recorded at 126 MHz or 101 MHz Chemical shifts (δ) are given in parts per million (ppm). Peaks are described as singlets (s), doublets (d), triplets (t), quartets (q), quintets (qt), sextets (sext), heptets (hept), multiplets (m) and broad (br). Coupling constants (*J*) are quoted to the nearest 0.5 Hz. All full assignments of NMR spectra were based on 2D NMR data (COSY, HSQC and HMBC and in some cases DEPT-135). Where compounds were analyzed as a mixture of isomers (*inc.* rotamers), they are referred to as *A* and *B*. NMR yields were determined by employing 1,4-dinitrobenzene or 1,4-dimethoxybenzene as an internal standard. Infrared (IR) spectra were recorded using a PerkinElmer Spectrum 100 FT-IR spectrometer as thin films or solids compressed a diamond plate. The wavenumbers ($\upsilon_{\max}$) are reported in cm^{-1}, using the intensities broad (br), strong (s), medium (m) and weak (w). High resolution mass spectrometry (HRMS) was determined at the University of Liverpool on Agilent 6540A Accurate-Mass QToF MS with Agilent Jetstream Source (ESI) or Agilent 7200 Series ToF MS (CI). Melting points were determined using a Reichert melting point apparatus (not corrected). Optical rotations were measured using an ADP400$^+$ polarimeter at the concentration and temperature stated. Enantiomeric excesses were determined using an Agilent 1290 Infinity chiral SFC with conditions as stated for each compound.

Experimental Procedures and Data

General Procedure A: Preparation of Amide Substrates from Carboxylic Acids

To a flame-dried flask was added carboxylic acid (100 mol%), CH_2Cl_2 (0.30 M) and DMF (0.1 mL/mmol). The reaction mixture was cooled to 0 °C and stirred for 5 min. Oxalyl chloride (130 mol%) was added dropwise over 5 min and the reaction mixture stirred at r.t. for 16 h under an atmosphere of N_2. The resulting mixture was concentrated *in vacuo* to afford the acid chloride which was used directly without further purification.

To a solution of the specified amine (130 mol%) and Et_3N (150 mol%) in CH_2Cl_2 (0.30 M) was added the acid chloride (100 mol%) dropwise at 0 °C. The reaction mixture was then stirred at r.t. for 16 h under an atmosphere of N_2. H_2O (10.0 mL/mmol) was added and the solution was extracted with CH_2Cl_2 (3 × 10.0 mL/mmol). The combined dorganic layers were washed with saturated aq. $NaHCO_3$ (10.0 mL/mmol) then H_2O (10.0 mL/mmol), dried (Na_2SO_4), filtered and concentrated *in vacuo*. The product was purified by FCC, under the conditions noted, to afford the target compound.

General Procedure B: Intramolecular N→C Alkyl Migration

To a flame-dried resealable Schlenk tube was added the specified (hetero)aromatic amide (100 mol%), Ir pre-catalyst (5.00–10.0 mol%) and the specified ligand (5.00–10.0 mol%). The tube was fitted with a rubber septum and evacuated and purged with N_2 three times. The specified solvent was added, the rubber septum was replaced with a Young's tap, and the reaction sealed under an atmosphere of N_2. The reaction was transferred to a heating block pre-heated at the specified temperature and stirred for 24–72 h as specified. The reaction was cooled to r.t. and concentrated *in vacuo*. Purification by FCC under the conditions noted afforded the target compound.

General Procedure C: Intermolecular N→C Alkyl Migration

To a flame-dried resealable Schlenk tube was added the specified (hetero)aromatic amide (100 mol%), amide coupling partner (200 mol%), Ir pre-catalyst (5.00–10.0 mol%) and ligand (5.00–10.0 mol%). The tube was fitted with a rubber septum and evacuated and purged with N_2 three times. The specified solvent was added, the rubber septum was replaced with a Young's tap and the reaction sealed under an atmosphere of N_2. The reaction was transferred to a heating block pre-heated at the specified temperature and stirred for 24–72 h as required. The reaction was cooled to r.t. and concentrated *in vacuo*. Purification by FCC under the conditions noted afforded the target compound.

General Procedure D: Synthesis of SPINOL-Ferrocene Ligands

To a flame-dried Schlenk tube was added the specified SPINOL derivative (200 mol%), 1,1-bis(dichlorophosphino)ferrocene (100 mol%) and DMAP (20.0 mol%). The tube was evacuated and refilled with N_2, and THF/CH_2Cl_2 (0.04 M, 2:1) were added. The solution was cooled to 0 °C and Et_3N (480 mol%) was added dropwise *via* syringe. The solution was warmed to r.t. and stirred for 16 h before being filtered through a pad of Celite® using Et_2O. The filtrate was concentrated *in vacuo* and purification by FCC under the conditions noted afforded the desired SPINOL-ferrocene ligands.

Discovery of the Alkyl Transfer Process (Scheme 2A)

N,*N*-Diisopropyl-3-(1-phenylethyl)furan-2-carboxamide (2)

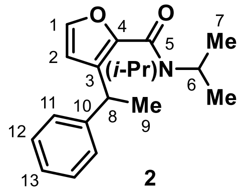

2

To a flame-dried resealable Schlenk tube was added **1a** (27.9 mg, 0.14 mmol), [Ir(cod)$_2$]BARF (9.10 mg, 0.007 mmol) and **L-4** (7.58 mg, 0.007 mmol). The tube was fitted with a rubber septum and evacuated and purged with N_2 three times. Styrene (65.5 µL, 0.57 mmol) and 1,4-dioxane (0.14 mL) were added, the rubber septum replaced with a Young's tap, and the reaction sealed under an atmosphere of N_2. The reaction was transferred to a heating block pre-heated at 100 °C and stirred for 24 h. The reaction was then cooled to r.t. and concentrated *in vacuo*. Purification by FCC (10–20% Et_2O/Hex) afforded the target compound (36.2 mg, 85%) as a colorless oil; $[\alpha]_D^{25}$ +77.9 (c = 0.20, $CHCl_3$); ν_{max}/cm^{-1}: 2968 (m), 2982 (m), 1622 (s), 1439 (s); ^1H NMR (400 MHz, $CDCl_3$): δ 7.27 –7.24 (4H, m, C**11**-H, C**12**-H), 7.22 (1H, d, *J* = 1.9 Hz, C**1**-H), 7.15 (1H, m, C**13**-H), 6.33 (1H, d, *J* = 1.9 Hz, C**2**-H), 4.44 (1H, q, *J* = 7.2 Hz, C**8**-H), 3.57 (2H, br, C**6**-H), 1.57 (3H, d, *J* = 7.2 Hz, C**9**-H), 1.25 (12H, br, C**7**-H); ^{13}C NMR (101 MHz, $CDCl_3$): δ 161.8 (C**5**), 146.0 (C**10**), 144.5 (C**4**), 140.8 (C**1**), 132.0 (C**3**), 128.3 (C**12**), 127.3 (C**11**), 126.0 (C**13**), 110.9 (C**2**), 34.8 (C**8**), 29.7 (C**6**), 21.1 (C**9**), 20.7 (C**7**); HRMS: (ESI$^+$) calculated for $C_{19}H_{25}NO_2Na$ 322.1777. Found $[M+Na]^+$ 322.1779. *The e.r. of this compound was determined to be 87:13 by SFC analysis against a racemic standard.*

SFC conditions: DAICEL CHIRALPAK-IB (25.0 cm), 99:1 CO_2:MeOH, 2.0 mL/min, 254 nm; Retention times: t_{minor} = 9.90 min, t_{major} = 10.40 min.

N-Isopropyl-3-(1-phenylethyl)furan-2-carboxamide (3)

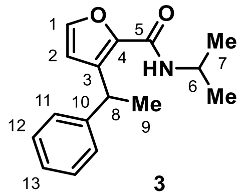

3

To a flame-dried resealable Schlenk tube was added **1a** (27.9 mg, 0.14 mmol), [Ir(cod)$_2$]BARF (9.10 mg, 0.007 mmol) and **L-4** (7.58 mg, 0.007 mmol). The tube was fitted with a rubber septum and evacuated and purged with N_2 three times. Styrene (65.5 µL, 0.57 mmol) and 1,4-dioxane (0.14 mL) were added, the rubber septum replaced with a Young's tap, and the reaction sealed under an atmosphere of N_2. The reaction was transferred to a heating block pre-heated at 100 °C and stirred for 24 h. The reaction was then cooled to r.t. and concentrated *in vacuo*. Purification by FCC (10–20% Et_2O/Hex) afforded the target compound (1.10 mg, 3%) as a colorless oil; ν_{max}/cm^{-1}: 3302 (br), 2868 (m), 2927 (m), 1644 (s); ^1H NMR (400 MHz, $CDCl_3$): δ 7.30–7.26 (2H, m, C**11**-H), 7.22–7.18 (3H, m, C**12**-H, C**1**-H), 7.10 (1H, m, C**13**-H), 6.31 (1H, d, *J* = 1.8 Hz, C**2**-H), 6.05 (1H, br, NH), 5.06 (1H, q, *J* = 7.2 Hz, C**8**-H), 4.18 (1H, dhept, *J* = 8.0, 6.6 Hz, C**6**-H), 1.50 (3H, d, *J* = 7.2 Hz, C**9**-H), 1.18–1.15

(6H, m, C**7**-H); ^{13}C NMR (101 MHz, $CDCl_3$): δ 156.7 (C**5**), 145.4 (C**10**), 142.3 (C**1**), 141.3 (C**4**), 136.2 (C**3**), 128.3 (C**12**), 127.4 (C**11**), 126.3 (C**13**), 112.2 (C**2**), 40.8 (C**6**), 34.4 (C**8**), 23.0 (C**7**), 21.1 (C**9**); HRMS: (ESI^+) calculated for $C_{16}H_{20}NO_2$ 258 .1489. Found $[M+H]^+$ 258.1497.

SPINOL Ligand Synthesis (Scheme 2B)

1,5-Bis(3-methoxyphenyl)pentan-3-one (S1)

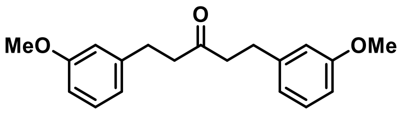

S1

To a solution of NaOH (22.7 g, 568 mmol) in H_2O (200 mL) and EtOH (200 mL) at 0 °C was added a solution of acetone (8.14 mL, 111 mmol) and *m*-anisaldehyde (27.0 mL, 222 mmol) in EtOH (50.0 mL) dropwise over 30 min. During this time the reaction mixture turned from colorless to yellow. After the addition was complete the reaction mixture was stirred at r,t, for a further 2 h after which time it was diluted with CH_2Cl_2 (150 mL) and the layers were separated. The organic layer was washed with H_2O/brine (1:1, 140 mL), dried (Na_2SO_4), filtered and concentrated *in vacuo* to give crude 1,5-bis(3-methoxyphenyl)-1,4-pentadien-3-one as a viscous, yellow oil. This material was used directly in the next step without further purification.

The crude 1,5-bis(3-methoxyphenyl)-1,4-pentadien-3-one (assuming 111 mmol) was dissolved in EtOAc (300 mL) and Pd/C (760 mg, 10% Pd, 7.20 mmol) was added under N_2. The atmosphere was saturated with H_2 gas (1 atm) and stirred vigorously under a balloon of H_2 until full consumption of starting material. The reaction mixture was then degassed with Ar, filtered through a pad of Celite® and concentrated *in vacuo*. Purification by FCC (10–20% EtOAc/Hex) afforded the title compound (9.09 mg, 30%) as a colorless oil; ^{1}H NMR (500 MHz, $CDCl_3$): δ 7.20–7.17 (2H, m, Ar<u>H</u>), 6.76–6.71 (6H, m, Ar<u>H</u>), 3.79 (6H, s, OC<u>H</u>$_3$), 2.88–2.85 (4H, t, J = 7.7 Hz, C<u>H</u>$_2$), 2.72–2.69 (4H, t, J = 7.7 Hz, C<u>H</u>$_2$); ^{13}C NMR (126 MHz, $CDCl_3$): δ 209.1 (<u>C</u>O), 160.0 (Ar<u>C</u>), 142.7 (Ar<u>C</u>), 129.7, 120.4, 114.1, 111.4 (4 × Ar<u>C</u>H), 55.1 (O<u>C</u>H$_3$), 44.6 (<u>C</u>H$_2$), 29.9 (<u>C</u>H$_2$).

The spectroscopic properties for this compound were consistent with the data available in the literature.[1]

4,4'-Dibromo-7,7'-dimethoxy-2,2',3,3'-tetrahydro-1,1'-spirobi[indene] (S2)

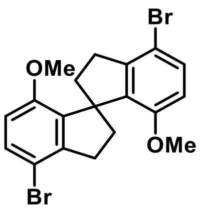

S2

To a solution of **S1** (10.3 g, 34.5 mmol) in acetone (70.0 mL) at 0 °C was added *N*-bromosuccinimide (11.3 g, 109 mmol) portion-wise under N_2, followed by 1 M aq. HCl (1.00 mL, 1.00 mmol). After 1 min, the cloudy reaction mixture turned clear which indicated reaction completion. The mixture was then concentrated *in vacuo* and the residue was re-dissolved in Et_2O (200 mL), washed with H_2O (100 mL), brine (100 mL), dried (Na_2SO_4), filtered and concentrated *in vacuo* to give crude 1,5-bis(2-bromo-5-methoxyphenyl)pentan-3-one as a colorless oil. The material was used directly in the next step without further purification.

A heterogeneous solution of the crude 1,5-bis(2-bromo-5-methoxyphenyl)pentan-3-one (13.9 g, 32.8 mmol) and phosphotungstic acid hydrate (14.2 g, 1.50 mmol) in toluene (180 mL) was heated at 140 °C under Dean-Stark

conditions for 48 h (the progress of the reaction was carefully monitored by ^{1}H NMR spectroscopy). The reaction mixture was then filtered through a pad of Celite®, washing with $CHCl_3$ (200 mL). The filtrate was concentrated *in vacuo* and purification by trituration with ice-cold Et_2O afforded the title compound (6.79 g, 47%) as a colorless solid; m.p. 154–156 °C (Et_2O); ^{1}H NMR (500 MHz, $CDCl_3$): δ 7.26 (2H, d, J = 8.5 Hz, Ar<u>H</u>), 6.52 (2H, d, J = 8.5 Hz, Ar<u>H</u>), 3.52 (6H, s, OC<u>H</u>$_3$), 3.09–3.03 (2H, m, C<u>H</u>$_2$), 2.98–2.91 (2H, m, C<u>H</u>$_2$), 2.36–2.28 (2H, m, C<u>H</u>$_2$), 2.18–2.13 (2H, m, C<u>H</u>$_2$); ^{13}C NMR (126 MHz, $CDCl_3$): δ 155.6, 144.9, 138.1 (3 × Ar<u>C</u>), 130.4 (Ar<u>C</u>H), 110.9 (Ar<u>C</u>H), 110.5 (Ar<u>C</u>), 61.9 (<u>C</u>), 55.4 (O<u>C</u>H$_3$), 38.0 (<u>C</u>H$_2$), 33.2 (<u>C</u>H$_2$).

The spectroscopic properties for this compound were consistent with the data available in the literature.[1]

4,4'-Dibromo-2,2',3,3'-tetrahydro-1,1'-spirobi[indene]-7,7'-diol (S3)

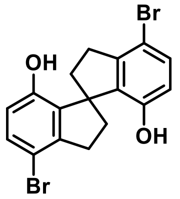

S3

To a Schlenk tube containing CH_2Cl_2 (30.0 mL) at -78 °C and under an atmosphere of N_2 was added BBr_3 (34.0 mL, 34.4 mmol) in one portion *via* syringe. Separately, a -78 °C solution of **S2** (5.00 g, 11.5 mmol) in CH_2Cl_2, (110 mL) was prepared. The BBr_3 solution was cannula-transferred to the solution of **S2** over 10 min. The reaction mixture was warmed to r.t. and stirred under an atmosphere of N_2 until complete conversion to product was observed by TLC analysis (approx. 48 h). The mixture was diluted with CH_2Cl_2 (60.0 mL), cooled to 0 °C and carefully quenched with saturated aq. $NaHCO_3$ (125 mL). The layers were separated and the organic layer washed with brine (100 mL), dried (Na_2SO_4), filtered and concentrated *in vacuo*. Purification by FCC (30% EtOAc/Hex) afforded the title compound (4.60 g, 98%) as a colorless foam; m.p. 134–136 °C ($CHCl_3$); ^{1}H NMR (500 MHz, $CDCl_3$): δ 7.32 (2H, d, J = 8.5 Hz, Ar<u>H</u>), 6.61 (2H, d, J = 8.5 Hz, Ar<u>H</u>), 4.53 (2H, s, O<u>H</u>), 3.11–2.97 (4H, m, C<u>H</u>$_2$), 2.36–2.31 (2H, m, C<u>H</u>$_2$), 2.25–2.19 (2H, m, C<u>H</u>$_2$); ^{13}C NMR (126 MHz, $CDCl_3$): δ 152.0 (Ar<u>C</u>), 145.5 (Ar<u>C</u>), 132.6 (Ar<u>C</u>H), 132.2 (Ar<u>C</u>), 116.6 (Ar<u>C</u>H), 110.0 (Ar<u>C</u>), 60.3 (<u>C</u>), 36.7 (<u>C</u>H$_2$), 32.7 (<u>C</u>H$_2$).

The spectroscopic properties for this compound were consistent with the data available in the literature.[2]

(*S*)-4,4'-Dibromo-2,2',3,3'-tetrahydro-1,1'-spirobi[indene]-7,7'-diyl bis((1*R*,2*S*,5*R*)-2-isopropyl-5-methylcyclohexyl) bis(carbonate) (S4)

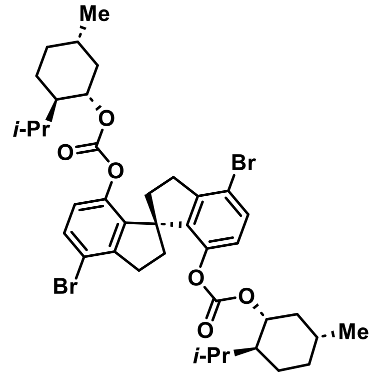

S4

To a solution of NaOH (2.00 g, 50.2 mmol) in H_2O (40.0 mL) was added **S3** (4.60 g, 11.3 mmol) and a solution of tetrabutylammonium bromide (1.67 g, 5.20 mmol) in $CHCl_3$ (40.0 mL). The mixture was cooled to 0 °C and (1*R*)-(-)-menthyl chloroformate (7.30 mL, 33.9 mmol) was added dropwise over 10 min. The reaction mixture was warmed to r.t. and stirred for 10 min. After this time, the layers were separated and the aqueous layer was extracted with CH_2Cl_2 (2 × 50.0 mL). The combined organic layers were washed with brine (50.0 mL), dried (Na_2SO_4), filtered and concentrated *in vacuo*. Recrystallization of the residue from hot hexane (approx. 350 mL) and trituration with acetone afforded the title compound (2.43 g, 40%) as a colorless solid. (Note: the title compound can also be purified by direct trituration of the residue with ice-cold acetone); $[\alpha]_D^{24}$ -157.8 (c = 0.23, $CHCl_3$); m.p. 211–213 °C (Et_2O); ^{1}H NMR (500 MHz, $CDCl_3$): δ 7.35 (2H, d, *J* = 8.5 Hz, Ar$\underline{H}$), 6.87 (2H, d, *J* = 8.5 Hz, Ar$\underline{H}$), 4.39–4.33 (2H, td, *J* = 11.0, 4.4 Hz, C$\underline{H}$), 3.10–2.96 (4H, m, C$\underline{H}$), 2.31–2.20 (4H, m, C$\underline{H}$), 1.67–1.60 (6H, m, C$\underline{H}$), 1.44–1.36 (2H, m, C$\underline{H}$), 1.33–1.27 (2H, m, C$\underline{H}$), 1.00–0.78 (18H, m, C$\underline{H}$), 0.71 (6H, d, *J* = 7.0 Hz, C$\underline{H}$); ^{13}C NMR (126 MHz, $CDCl_3$): δ 152.7 ($\underline{C}$O), 146.8 (Ar$\underline{C}$), 145.5 (Ar$\underline{C}$), 140.5 (Ar$\underline{C}$H), 131.2 (Ar$\underline{C}$H), 122.6 (Ar$\underline{C}$H), 116.5 (Ar$\underline{C}$), 79.3 ($\underline{C}$H), 61.7 ($\underline{C}$). 46.7 ($\underline{C}$H), 40.2 ($\underline{C}H_2$), 38.4 ($\underline{C}H_2$), 34.0 ($\underline{C}H_2$), 32.9 ($\underline{C}H_2$), 31.3 ($\underline{C}$H), 26.1 ($\underline{C}H_2$), 23.2 ($\underline{C}H_2$), 22.0 ($\underline{C}H_3$), 20.7 ($\underline{C}H_3$), 16.1 ($\underline{C}H_3$).

The spectroscopic properties for this compound were consistent with the data available in the literature.[3]

(*S*)-4,4'-Diphenyl-2,2',3,3'-tetrahydro-1,1'-spirobi[indene]-7,7'-diol (S5)

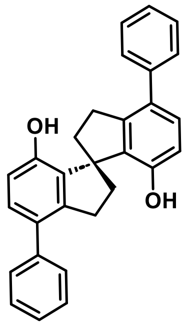

S5

A Schlenk tube was charged with S**4** (750 mg, 0.97 mmol), $Pd(PPh_3)_4$ (70.0 mg, 0.06 mmol), phenyl boronic acid (415 mg, 3.40 mmol) and Na_2CO_3 (413 mg, 3.90 mmol). The tube was evacuated and refilled with N_2 before degassed DME (4.00 mL), H_2O (4.00 mL) and EtOH (2.00 mL) were added. The tube was sealed and heated to 100 °C for 16 h. The reaction mixture was then cooled to r.t. and filtered through a pad of Celite® using CH_2Cl_2 as the eluent. After concentration *in vacuo*, the crude mixture was filtered through a pad of silica using EtOAc/Hex (20:80) and again concentrated *in vacuo*. The crude product was dissolved in THF (10.0 mL), H_2O (10.0 mL) and EtOH (10.0 mL), and KOH (100 mg, 1.75 mmol) was added. After heating at reflux for 1 h, solvents were removed *in vacuo*, H_2O (15.0 mL) was added, the mixture was acidified to a pH of 1 with 1 M aq. HCl and extracted with Et_2O (3 × 15.0 mL). The combined organic layers were washed with brine (15.0 mL), dried (Na_2SO_4), filtered and concentrated *in vacuo*. Purification by FCC (10–20% EtOAc/Hex) afforded the title compound (0.33 g, 84%) as a colorless solid; $[\alpha]_D^{23}$ -95.3 (c = 0.21, $CHCl_3$); m.p. 163–165 °C ($CHCl_3$); ^1H NMR (500 MHz, $CDCl_3$): δ 7.50–7.43 (8H, m, ArH), 7.37–7.33 (2H, m, ArH), 7.28 (2H, d, *J* = 8.2 Hz, ArH), 6.83 (2H, d, *J* = 8.2 Hz, ArH), 4.73 (s, 2H, OH), 3.27–3.17 (2H, m, CH_2), 3.07 (2H, dd, *J* = 16.2, 8.2 Hz, CH_2), 2.43 (2H, dd, *J* = 12.9, 8.8 Hz, CH_2), 2.28–2.21 (2H, m, CH_2); ^{13}C NMR (126 MHz, $CDCl_3$): δ 152.3 (ArC), 143.3 (ArC), 140.7 (ArC), 131.8 (ArC), 130.6 (ArCH), 130.4 (ArCH), 128.6 (ArCH), 128.3 (ArC), 126.7 (ArCH), 115.1 (ArCH), 57.9 (C), 37.3 (CH_2), 31.3 (CH_2).

The spectroscopic properties for this compound were consistent with the data available in the literature.[4]

(*S*)-L4

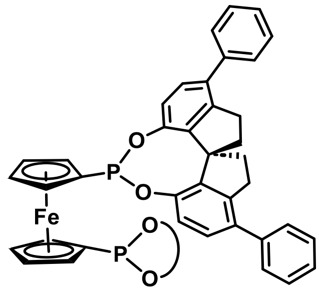

L4

General Procedure D: Compound **S5** (171 mg, 0.42 mmol), 1,1-bis(dichlorophosphino)ferrocene (81.8 mg, 0.22 mmol), DMAP (5.20 mg, 0.04 mmol) and Et_3N (0.15 mL, 1.02 mmol) were employed. Purification by FCC (2% Et_2O/Hex, SiO_2 deactivated with NEt_3 (10% w/v)) afforded the title compound (123 mg, 28%) as an orange solid;

$[\alpha]_D^{23}$ -190.9 (c = 0.12, CH_2Cl_2); m.p. decomposition at 170 °C (pentane); ^{1}H NMR (500 MHz, $CDCl_3$): δ 7.49–7.28 (22H, m, ArH), 7.15 (2H, d, J = 7.9 Hz, ArH), 6.85 (2H, d, J = 8.2 Hz, ArH), 6.13 (2H, d, J = 8.1 Hz, ArH), 4.70–4.57 (4H, m, ferrocene), 4.43–4.26 (2H, m, ferrocene), 3.77–3.71 (2H, m, ferrocene), 3.27–3.14 (4H, m, CH_2), 2.86–2.78 (4H, m, CH_2), 2.36–2.30 (4H, m, CH_2), 2.07–1.95 (4H, m, CH_2); ^{13}C NMR (126 MHz, $CDCl_3$): 149.4 (ArC), 145.6 (ArC), 142.8 (ArC), 142.2 (ArC), 141.0 (ArC), 140.9 (ArC), 140.8 (ArC), 140.7 (ArC), 135.3 (ArC), 134.5 (ArC), 129.4 (ArCH), 128.8 (ArCH), 128.7 (ArCH), 128.3 (ArCH), 128.2 (ArCH), 127.7 (ArCH), 126.9 (ArCH), 126.7 (ArCH), 123.0 (ArCH), 121.5 (ArCH), 73.3 (ferrocene), 73.0 (ferrocene), 72.6 (ferrocene), 71.8 (ferrocene), 70.9 (ferrocene), 59.5 (C), 38.5 (CH_2), 37.9 (CH_2), 31.2 (CH_2), 30.7 (CH_2); ^{31}P NMR (202 MHz, $CDCl_3$): δ 159.67.

The spectroscopic properties for this compound were consistent with the data available in the literature.[4]

***(R)*-L5**

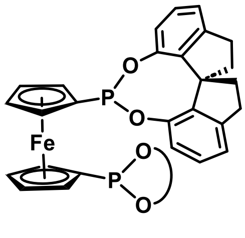

L5

General Procedure D: (*S*)-2,2',3,3'-tetrahydro-1,1'-spirobi[1H-indene]-7,7'-diol (150 mg, 0.60 mmol), 1,1-bis(dichlorophosphino)ferrocene (115 mg, 0.28 mmol), DMAP (7.26 mg, 0.06 mmol) and Et_3N (0.20 mL, 1.43 mmol) were employed. Purification by FCC (5% Et_2O/Hex, SiO_2 deactivated with NEt_3 (10% w/v)) afforded the title compound (119 mg, 54%) as an orange solid; $[\alpha]_D^{20}$ +454.0 (c = 0.16, $CHCl_3$); m.p. decomposition at 158 °C (pentane); ^{1}H NMR (500 MHz, $CDCl_3$): δ 7.23 (1H, t, J = 7.8 Hz, ArH), 7.07 (1H, d, J = 7.3 Hz, ArH), 7.02 (1H, d, J = 7.8 Hz, ArH), 6.89 (1H, d, J = 7.3 Hz, ArH), 6.77 (1H, t, J = 7.8 Hz, ArH), 6.01 (1H, d, J = 7.8 Hz, ArH), 4.59–4.56 (2H, m, ferrocene), 4.54–4.51 (2H, m, ferrocene), 4.30–4.28 (2H, m, ferrocene), 3.67–3.65 (2H, m, ferrocene), 3.11–2.98 (4H, m, CH_2), 2.87–2.77 (4H, m, CH_2), 2.24–2.17 (4H, m, CH_2), 2.00–1.94 (4H, m, CH_2); ^{13}C NMR (126 MHz, $CDCl_3$): 150.0 (ArC), 146.2 (ArC), 145.4 (ArC), 144.8 (ArC), 142.5 (ArC), 140.7 (ArC), 128.8 (ArCH), 127.1 (ArCH), 122.2 (ArCH), 121.3 (ArCH), 120.7 (ArCH), 120.7 (ArCH), 120.4 (ArCH), 73.1 (ferrocene), 72.8 (ferrocene), 72.4 (ferrocene), 71.8 (ferrocene), 70.6 (ferrocene), 59.9 (C), 38.5 (CH_2), 37.9 (CH_2), 31.0 (CH_2), 30.6 (CH_2); ^{31}P NMR (202 MHz, $CDCl_3$): δ 159.36.

The spectroscopic properties for this compound were consistent with the data available in the literature.[4]

*(S)-**L5** was also prepared and mixed in a 1:1 ratio with (R)-**L5** to prepare the racemates of **4p** to **4t** and **4z'**.*

Full Optimization of Intramolecular Transfer (Scheme 2B)

Screening of Ferrocene-SPINOL ligands

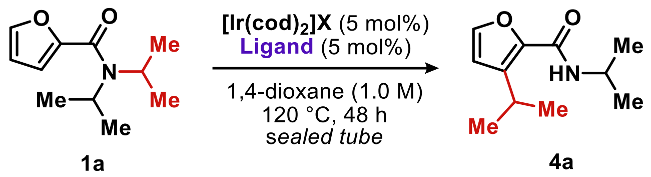

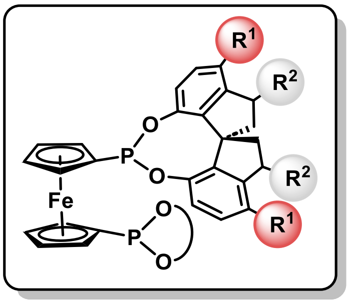

Ligand	X	R^1	R^2	Yield
L-1	BARF	mesityl	H	19%
L-2	BARF	Br	H	28%
L-3	BARF	2-naphthyl	H	33%
L-4	BARF	phenyl	H	47%
L-6	BARF	phenyl	phenyl	0%
L-7	BARF	phenyl	phenyl	29%
L-8	BARF	pyrenyl	H	50%
L-9	BARF	3,5-*t*-butylphenyl	H	60%
L-10	BARF	pentafluorophenyl	H	9%
L-11	BARF	pentamethylphenyl	H	18%
L-12	BARF	TMS	H	9%
L-13	BARF	H	*p*-methoxyphenyl	0%
L-14	BARF	Br	2-naphthyl	0%
L-15	BARF	H	*p*-fluorophenyl	19%
L-16	BARF	F	phenyl	0%

Screening of alternative ligands

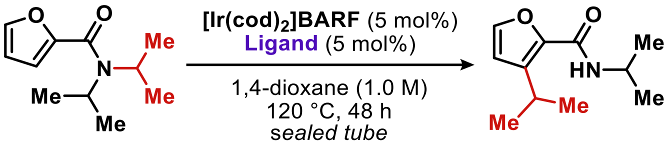

1a **4a**

Ligand	Yield
(*R*)-BINOL	0%
(*R*)-DM-Segphos	0%
(*R*)-BINAP	0%
(*R*)-Josiphos	0%
dppp	0%
dppe	0%
d^{F}ppp	0%
L-17	0%
L-18	0%
L-19	0%
L-20	8%
L-21	0%
L-22	0%
L-23	0%
L-24	0%
L-25	0%
L-26	0%
L-27	0%

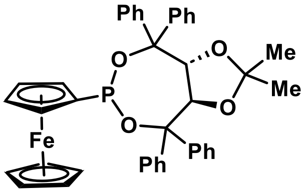

L-17

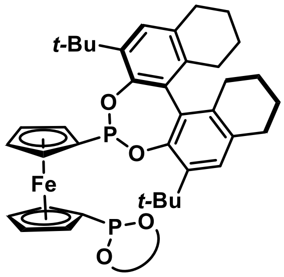

L-18

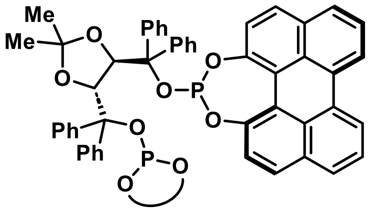

L-19

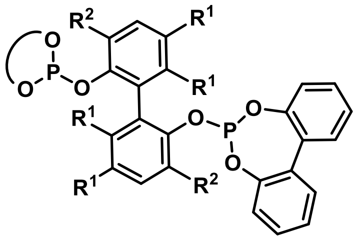

L-20 (R^1 = H, R^2 = H)
L-21 (R^1 = Me, R^2 = H)
L-22 (R^1 = Me, R^2 = Me)
L-23 (R^1 = Me, R^2 = *t*-Bu)

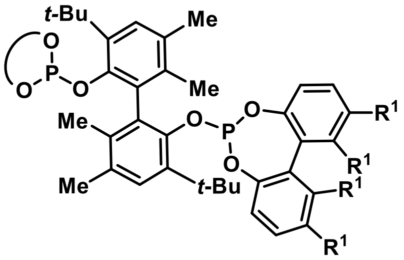

L-24 (R^1 = *t*-Bu)
L-25 (R^1 = F)
L-26 (R^1 = OMe)

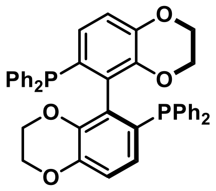

L-27

*Optimization with **L-4***

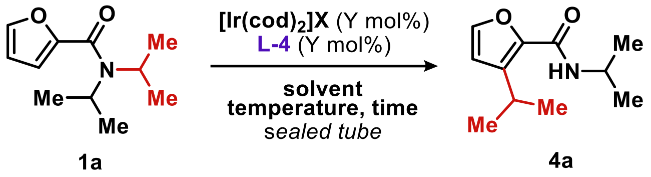

X	Y	Solvent	[C]	Time (h)	T (°C)	Yield
BARF	5	1,4-dioxane	1 M	48 h	120	45%
BARF	5	1,4-dioxane	0.5 M	48 h	120	29%
SbF_6	5	1,4-dioxane	1 M	48 h	120	20%
PF_6	5	1,4-dioxane	1 M	48 h	120	0%
BF_4	5	1,4-dioxane	1 M	48 h	120	0%
OTf	5	1,4-dioxane	1 M	48 h	120	0%
BARF	5	DCE	1 M	48 h	120	0%
BARF	5	THF	1 M	48 h	120	0%
BARF	5	mesitylene	1 M	48 h	120	27%
BARF	5	CPME	1 M	48 h	120	0%
BARF	5	toluene	1 M	48 h	120	71%
BARF	5	1,2-DCB	1 M	48 h	120	76%
BARF	5	1,2-DCB	1 M	48 h	100	2%
BARF	5	1,2-DCB	1 M	48 h	140	0%
BARF	5	1,4-dioxane	1 M	72 h	120	60%
BARF	5	1,2-DCB	1 M	72 h	120	95%
BARF	2.5	1,2-DCB	1 M	72 h	120	28%

Intramolecular Isopropyl N→C Alkyl Transfer Reactions: Substrates (Table 1A)

N,*N*-Diisopropylfuran-2-carboxamide (1a)

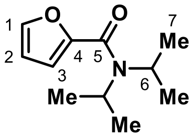

1a

General Procedure A: 2-Furoic acid (1.80 g, 15.6 mmol), diisopropylamine (4.40 mL, 31.3 mmol), oxalyl chloride (1.50 mL, 17.3 mmol) and Et_3N (2.82 mL, 20.3 mmol) were employed. Purification by FCC (5–20% EtOAc/Hex) afforded the title compound (2.45 g, 89%) as a pale yellow oil; ^1H NMR (500 MHz, $CDCl_3$): δ 7.42–7.41 (1H, dd, J = 1.8, 0.9 Hz, C**1**-H), 6.83–6.82 (1H, dd, J = 3.4, 0.9 Hz, C**3**-H), 6.43–6.42 (1H, dd, J = 3.4, 1.8 Hz, C**2**-H), 3.96 (2H, br, C**6**-H), 1.37 (12H, s, C**7**-H); ^{13}C NMR (126 MHz, $CDCl_3$): δ 160.3 (C**5**), 149.5 (C**4**), 142.8 (C**1**), 114.0 (C**3**), 110.9 (C**2**), 48.9 (C**6**), 20.9 (C**7**).

The spectroscopic properties for this compound were consistent with the data available in the literature.[5]

***N*-Methyl-*N*-isopropylfuran-2-carboxamide (1b)**

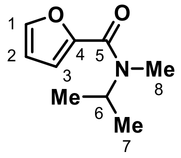

1b

General Procedure A: 2-Furoic acid (788 mg, 7.03 mmol), *N*-methylpropan-2-amine (1.00 mL, 9.57 mmol), oxalyl chloride (0.81 mL, 9.57 mmol) and Et_3N (11.04 mL, 16.7 mmol) were employed. Purification by FCC (10–30% EtOAc/Hex) afforded the title compound (490 mg, 40%, 0.5:0.5 mixture of rotamers *A*:*B*) as a brown solid; m.p. 46-48 °C (hexane); ν_{max}/cm^{-1}: 2975 (br), 1610 (m), 1489 (m); ^1H NMR (500 MHz, $CDCl_3$) δ 7.40 (1H, br, C**1**-H, *A*+*B*), 6.84 (1H, br, C**3**-H, *A*+*B*), 6.49 (1H, dd, *J* = 3.4, 1.8 Hz, C**2**-H, *A*+*B*), 4.81–4.50 (1H, m, C**6**-H, *A*+*B*), 2.92 (3H, br, C**8**-H, *A*+*B*), 1.12 (6H, br, C**7**-H, *A*+*B*); ^{13}C NMR (126 MHz, $CDCl_3$) δ 160.2 (C**5**, *A*+*B*), 148.4 (C**4**, *A*+*B*), 143.5 (C**1**, *A*+*B*), 115.6 (C**3**, *A*+*B*), 110.0 (C**2**, *A*+*B*), 48.7 (C**6**, *A*), 45.2 (C**6**, *B*), 29.6 (C**8**, *B*), 26.8 (C**8**, *A*), 20.6 (C**7**, *A*), 19.3 (C**7**, *B*); HRMS: (ESI^+) calculated for $C_9H_{14}NO_2$ 168.1025. Found $[M+H]^+$ 168.1017.

***N*-Ethyl-*N*-isopropylfuran-2-carboxamide (1c)**

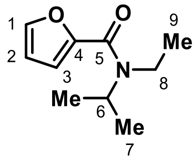

1c

General Procedure A: 2-Furoic acid (495 mg, 4.42 mmol), *N*-ethylpropan-2-amine (500 mg, 5.74 mmol), oxalyl chloride (0.49 mL, 5.84 mmol) and Et_3N (0.92 mL, 6.63 mmol) were employed. Purification by FCC (10–30% EtOAc/Hex) afforded the title compound (259 mg, 32%) as a colorless oil; ν_{max}/cm^{-1}: 2973 (br), 1614 (m), 1424 (m); ^1H NMR (500 MHz, $CDCl_3$): δ 7.46 (1H, dd, *J* = 1.8, 0.8 Hz, C**1**-H), 6.95 (1H, br, C**3**-H), 6.46 (1H, dd, *J* = 3.4, 1.8 Hz, C**2**-H), 4.62 (1H, br, C**6**-H), 3.47 (2H, br, C**8**-H), 1.27–1.25 (9H, m, C**9**-H, C**7**-H); ^{13}C NMR (126 MHz, $CDCl_3$): δ 169.5 (C**5**), 160.1 (C**4**), 143.3 (C**1**), 135.8 (C**3**), 111.1 (C**2**), 50.0 (C**6**), 35.2 (C**8**), 22.1 (C**9** or C**7**), 19.0 (C**9** or C**7**); HRMS: (ESI^+) calculated for $C_{10}H_{16}NO_2$ 182.1181. Found $[M+H]^+$ 182.1177.

***N*-Cyclohexyl-*N*-isopropylfuran-2-carboxamide (1d)**

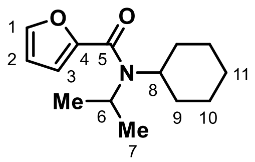

1d

General Procedure A: 2-Furoic acid (91.3 mg, 0.815 mmol), *N*-isopropylcyclohexanamine (0.17 mL, 1.06 mmol), oxalyl chloride (0.09 mL, 1.06 mmol) and Et_3N (0.17 mL, 1.22 mmol) were employed. Purification by FCC (20–40% EtOAc/Hex) afforded the title compound (113 mg, 60%) as a brown oil; ν_{max}/cm^{-1}: 3385 (br), 2932 (m), 1619 (s), 1449 (m); ^1H NMR (500 MHz, $CDCl_3$): δ 7.43 (1H, dd, *J* = 1.8, 0.8 Hz, C**1**-H), 6.81 (1H, dd, *J* =

3.5, 0.8 Hz, C**3**-H), 6.44 (1H, dd, J = 3.4, 1.8 Hz, C**2**-H), 3.74 (2H, br, C**6**-H, C**8**-H), 1.84–1.72 (4H, m, C**9**-H), 1.63 (1H, m, C**11**-H), 1.38 (6H, br, C**7**-H), 1.31–1.13 (4H, m, C**10**-H); ^{13}C NMR (126 MHz, $CDCl_3$): δ 169.2 (C**5**), 149.6 (C**4**), 142.9 (C**1**), 113.7 (C**3**), 110.9 (C**2**), 54.0 (CH), 46.4 (CH), 25.0 (C**11**), 24.7 (C**9**), 20.3 (C**10**), 19.3 (C**7**); HRMS: (ESI$^+$) calculated for $C_{14}H_{22}NO_2$ 236.1651. Found $[M+H]^+$ 236.1644.

***N*-(*tert*-Butyl)-*N*-isopropylfuran-2-carboxamide (1e)**

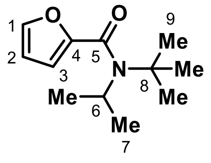

1e

General Procedure A: 2-Furoic acid (1.25 g, 11.2 mmol), *N*-isopropyl-2-methylpropan-2-amine (1.45 mL, 9.14 mmol), oxalyl chloride (0.77 mL, 9.14 mmol) and Et_3N (1.47 mL, 10.6 mmol) were employed. Purification by FCC (0–40% EtOAc/Hex) afforded the title compound (185 mg, 8%) as a brown oil; ν_{max}/cm^{-1}: 2973 (br), 1615 (s), 1485 (m); ^{1}H NMR (500 MHz, $CDCl_3$): δ 7.38 (1H, br, C**1**-H), 6.74 (1H, d, J = 3.4 Hz, C**3**-H), 6.39 (1H, dd, J = 3.4, 1.8 Hz, C**2**-H), 3.86 (1H, hept, J = 7.0 Hz, C**6**-H), 1.40 (9H, s, C**9**-H), 1.30 (6H, d, J = 7.0 Hz, C**7**-H); ^{13}C NMR (126 MHz, $CDCl_3$): δ 164.2 (C**5**), 151.4 (C**4**), 142.4 (C**1**), 113.4 (C**3**), 111.3 (C**2**), 58.6 (C**8**), 49.6 (C**6**), 29.2 (C**9**), 23.4 (C**7**); HRMS: (ESI$^+$) calculated for $C_{12}H_{19}NO_2Na$ 232.1313. Found $[M+Na]^+$ 232.1306.

***N*,*N*-Diisopropyl-4,5-dimethylfuran-2-carboxamide (1f)**

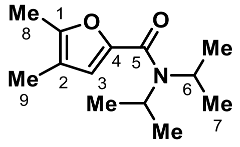

1f

General Procedure A: 4,5-dimethylfuran-2-carboxylic acid (150 mg, 1.07 mmol), diisopropylamine (0.20 mL, 1.39 mmol), oxalyl chloride (0.12 mL, 1.39 mmol) and Et_3N (0.22 mL, 1.61 mmol) were employed. Purification by FCC (10–20% EtOAc/Hex) afforded the title compound (0.22 g, 92%) as a yellow solid; m.p. 51–53 °C (hexane); ν_{max}/cm^{-1}: 2964 (m), 1611 (s), 1432 (s); ^{1}H NMR (500 MHz, $CDCl_3$): δ 6.58 (1H, s, C**3**-H), 4.00 (2H, br, C**6**-H), 2.23 (3H, s, CH_3), 1.95 (3H, s, CH_3), 1.36 (12H, d, J = 6.8 Hz, C**7**-H); ^{13}C NMR (126 MHz, $CDCl_3$): δ 160.6 (C**5**), 148.9 (C), 146.2 (C), 117.4 (C**1**), 115.6 (C**3**), 48.1 (br, C**6**), 21.0 (C**7**), 11.6 (CH_3), 9.8 (CH_3); HRMS: (ESI$^+$) calculated for $C_{13}H_{21}NO_2$ 224.1651. Found $[M+H]^+$ 224.1645.

***N*,*N*-Diisopropyl-5-methylfuran-2-carboxamide (1g)**

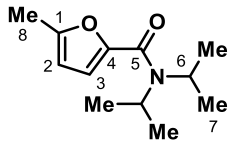

1g

General Procedure A: 5-methylfuran-2-carboxylic acid (500 mg, 3.96 mmol), diisopropylamine (0.60 mL, 4.21 mmol), oxalyl chloride (0.36 mL, 4.21 mmol) and Et_3N (0.83 mL, 5.94 mmol) were employed. Purification by

FCC (10–20% EtOAc/Hex) afforded the title compound (0.60 g, 72%) as a yellow solid; m.p. 38–40 °C (hexane); ^{1}H NMR (500 MHz, $CDCl_3$): δ 6.50 (1H, d, J = 3.2 Hz, C**1**-H), 6.05 (1H, d, J = 3.2 Hz, C**2**-H), 4.02 (2H, br, C**6**-H), 2.35 (3H, d, J = 0.9 Hz, C**8**-H), 1.39 (12H, d, J = 6.8 Hz, C**7**-H); ^{13}C NMR (126 MHz, $CDCl_3$) δ 160.5 (C**5**), 153.4 (C**1**), 147.5 (C**4**), 115.1 (C**3**), 107.1 (C**2**), 48.1 (br, C**6**), 20.9 (C**7**), 13.7 (C**8**).

The spectroscopic properties for this compound were consistent with the data available in the literature.[6]

4,5-Dibromo-*N*,*N*-diisopropylfuran-2-carboxamide (1h)

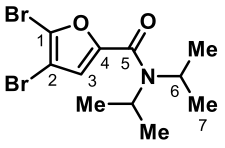

1h

General Procedure A: 4,5-dibromofuran-2-carboxylic acid (232 mg, 0.86 mmol), diisopropylamine (0.16 mL, 1.12 mmol), oxalyl chloride (0.10 mL, 1.12 mmol) and Et_3N (0.18 mL, 1.29 mmol) were employed. Purification by FCC (20% EtOAc/Hex) afforded the title compound (230 mg, 76%) as a colorless solid; m.p. 57–59 °C (pentane); ν_{max}/cm^{-1}: 3088 (m), 1624 (s), 1490 (m), 1329 (s), 747 (s); ^{1}H NMR (500 MHz, $CDCl_3$): δ 6.82 (1H, s, C**3**-H), 3.90 (2H, br, C**6**-H), 1.37 (12H, s, C**7**-H); ^{13}C NMR (126 MHz, $CDCl_3$) δ 158.2 (C**5**), 151.0 (C), 124.0 (C**1**), 118.4 (C), 103.0 (C**3**), 49.4 (C**6**), 20.8 (C**7**); HRMS: (ESI^{+}) calculated for $C_{11}H_{16}{}^{79}Br_2NO2$ 351.9548. Found $[M+H]^{+}$ 351.9541.

5-Chloro-*N*,*N*-diisopropylfuran-2-carboxamide (1i)

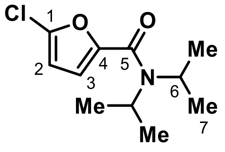

1i

General Procedure A: 4-chlorofuran-2-carboxylic acid (500 mg, 3.41 mmol), diisopropylamine (0.63 mL, 4.43 mmol), oxalyl chloride (0.38 mL, 4.43 mmol) and Et_3N (0.71 mL, 5.12 mmol) were employed. Purification by FCC (0–20% EtOAc/Hex) afforded the title compound (0.53 g, 68%) as a colorless solid; m.p. 47–49 °C (pentane); ν_{max}/cm^{-1}: 2970.9 (m), 1625 (s), 1487 (s), 1332 (m), 776 (s); ^{1}H NMR (500 MHz, $CDCl_3$) δ 6.83 (1H, d, J = 3.5 Hz, C**3**-H), 6.23 (1H, d, J = 3.5 Hz, C**2**-H), 3.95 (2H, br, C**6**-H), 1.37 (12H, br, C**7**-H); ^{13}C NMR (126 MHz, $CDCl_3$): δ 159.0 (C**5**), 148.8 (C), 137.3 (C), 116.4 (C**3**), 107.8 (C**2**), 47.8 (br, C**6**), 20.9 (C**7**); HRMS: (ESI^{+}) calculated for $C_{11}H_{26}{}^{35}ClNO_2$ 230.0948. Found $[M+H]^{+}$ 230.0940.

***N,N*-Diisopropyl-5-phenylfuran-2-carboxamide (1j)**

Synthesis of 5-bromo-N,N-diisopropylfuran-2-carboxamide

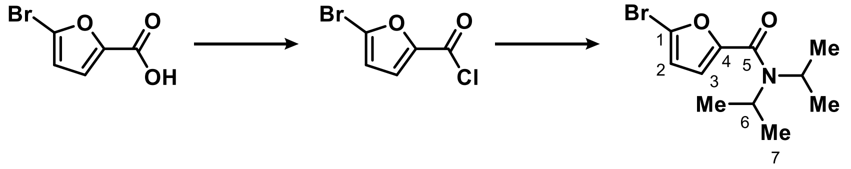

General Procedure A: 2-Furoic acid (3.00 g, 15.7 mmol), diisopropylamine (2.88 mL, 20.4 mmol), oxalyl chloride (1.75 mL, 20.4 mmol) and Et_3N (2.38 mL, 23.6 mmol) were employed. Purification by FCC (10–20% EtOAc/Hex) afforded the title compound (2.17 g, 50%) as a colorless solid; m.p. 59–61 °C (pentane); ^{1}H NMR (500 MHz, $CDCl_3$): δ 6.81 (1H, d, *J* = 3.5 Hz, C**3**-H), 6.36 (1H, d, *J* = 3.5 Hz, C**2**-H), 3.95 (2H, br, C**6**-H), 1.36 (12H, br, C**7**-H); ^{13}C NMR (126 MHz, $CDCl_3$): δ 159.1 (C**5**), 151.1 (C**4**), 123.2 (C**1**), 116.6 (C**3**), 112.9 (C**2**), 48.2 (C**6**), 20.8 (C**7**).

The spectroscopic properties for this compound were consistent with the data available in the literature.[7]

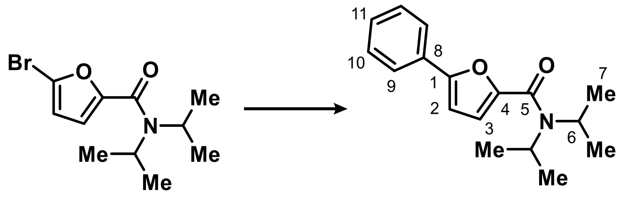

1j

To a solution of 5-bromofuran-2-carboxylic acid (541 mg, 1.97 mmol) in DMF (7.00 mL) was added phenylboronic acid (276 mg, 2.26 mmol), $Pd(PPh_3)_4$ (227 mg, 0.20 mmol), K_3PO_4 (834 mg, 3.93 mmol). The reaction was stirred at 105 °C for 16 h under an atmosphere of N_2, then quenched with H_2O (20.0 mL), acidified to a pH of 1 with 1 M aq. HCl, EtOAc (20.0 mL) added, and the layers separated. The aqueous layer was extracted with EtOAc (3 × 20.0 mL), dried ($MgSO_4$), filtered and concentrated *in vacuo*. Purification by FCC (0–40% EtOAc/Hex) afforded the title compound (463 mg, 87%) as a pale yellow solid; m.p. 52–55 °C (pentane); ν_{max}/cm^{-1}: 3067 (w), 1612 (s), 1332 (s); ^{1}H NMR (500 MHz, $CDCl_3$) δ 7.69–7.67 (2H, m, C**8**-H), 7.41–7.38 (2H, m, C**9**-H), 7.30 (1H, m, C**10**-H), 6.96 (1H, d, *J* = 3.5 Hz, C**3**-H), 6.69 (1H, d, *J* = 3.5 Hz, C**2**-H), 4.02 (2H, br, C**6**-H), 1.43 (12H, br, C**7**-H); ^{13}C NMR (126 MHz, $CDCl_3$): δ 160.2 (C**5**), 154.2 (C**4**), 150.8 (C**8**), 148.8 (C**1**), 130.2 (C**4**), 128.8 (C**9**), 128.2 (C**10**), 124.2 (C**8**), 116.7 (C**3**), 106.3 (C**2**), 48.0 (C**6**), 21.0 (C**7**); HRMS: (ESI$^+$) calculated for $C_{17}H_{22}NO_2$ 272.1651. Found $[M+H]^+$ 272.1650.

***N*,*N*-Diisopropyl-1-methyl-1*H*-pyrrole-2-carboxamide (1k)**

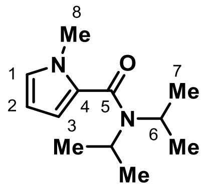

1k

General Procedure A: 2-Methyl-2-pyrrole carboxylic acid (626 mg, 5.00 mmol), diisopropylamine (0.92 mL, 6.50 mmol), oxalyl chloride (0.56 mL, 6.50 mmol) and Et_3N (1.00 mL, 7.50 mmol) were employed. Purification by FCC (10–20% EtOAc/Hex) afforded the title compound (700 mg, 34%) as an orange oil; ^{1}H NMR (500 MHz, $CDCl_3$): δ 6.63 (1H, m, C**1**-H), 6.23 (1H, m, C**3**-H), 6.05 (1H, m, C**2**-H), 4.02 (2H, br, C**6**-H), 3.72 (3H, s, C**8**-H), 1.35 (12H, d, *J* = 6.9 Hz, C**7**-H); ^{13}C NMR (126 MHz, $CDCl_3$): δ 163.8 (C**5**), 127.9 (C**4**), 124.5 (C**1**), 109.7 (C**3**), 106.6 (C**2**), 48.3 (C**8**), 35.4 (C**6**), 21.1 (C**7**).

The spectroscopic properties for this compound were consistent with the data available in the literature.[8]

***N*,*N*-Diisopropylthiophene-2-carboxamide (1l)**

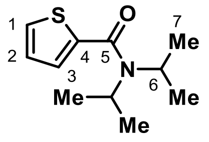

1l

General Procedure A: 2-Thiophene carboxylic acid (640 mg, 5.00 mmol), diisopropylamine (0.90 mL, 6.50 mmol), oxalyl chloride (0.56 mL, 6.50 mmol) and Et_3N (1.00 mL, 7.50 mmol) were employed. Purification by FCC (10–20% EtOAc/Hex) afforded the title compound (700 mg, 63%) as a pale orange solid; m.p. 58–61 °C (pentane); ^{1}H NMR (500 MHz, $CDCl_3$): δ 7.40–7.39 (1H, dd, *J* = 5.0, 1.2 Hz, C**1**-H), 7.22–7.21 (1H, dd, *J* = 3.7, 1.2 Hz, C**3**-H), 7.04–7.02 (1H, dd, *J* = 5.0, 3.7 Hz, C**2**-H), 3.99 (2H, br, C**6**-H), 1.39 (12H, d, *J* = 4.3 Hz C**7**-H); ^{13}C NMR (126 MHz, $CDCl_3$): δ 163.9 (C**5**), 139.9 (C**4**), 127.3 (C**1**), 126.8 (C**3**), 126.4 (C**2**), 48.9 (br, C**6**), 20.9 (C**7**).

The spectroscopic properties for this compound were consistent with the data available in the literature.[5]

***N*,*N*-Diisopropylbenzofuran-2-carboxamide (1m)**

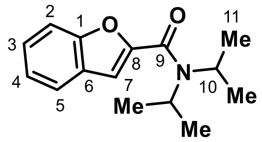

1m

General Procedure A: Benzofuran-2-carboxylic acid (2.00 g, 12.3 mmol), diisopropylamine (2.30 mL, 16.0 mmol), oxalyl chloride (1.37 mL, 16.0 mmol) and Et_3N (2.57 mL, 18.5 mmol) were employed. Purification by FCC (0–10% EtOAc/Hex) afforded the title compound (2.59 g, 86%) as a colorless solid; m.p. 95–97 °C (hexane); ν_{max}/cm^{-1}: 2965 (m), 1611 (s), 1548 (s), 1431 (s), 1328 (s); ^{1}*H* NMR (500 MHz, $CDCl_3$) δ 7.65 (1H, dt, *J* = 7.8,

1.0 Hz, C**5**-H), 7.52 (1H, dd, *J* = 8.3, 1.0 Hz, C**2**-H), 7.38 (1H, ddd, *J* = 8.3, 7.2, 1.3 Hz, C**4**-H), 7.29 (1H, m, C**3**-H), 7.13 (1H, d, *J* = 1.0 Hz, C**7**-H), 4.00 (2H, br, C**10**-H), 1.44 (12H, br, C**11**-H); ^{13}C NMR (126 MHz, $CDCl_3$): δ 160.9 (C**9**), 154.2 (C**6**), 150.8 (C**8**), 127.3 (C**1**), 125.8 (C**4**), 123.4 (C**3**), 122.0 (C**5**), 111.7 (C**2**), 109.0 (C**7**), 48.6 (C**10**), 20.8 (C**11**). HRMS: (ESI^+) calculated for $C_{15}H_{20}NO_2$ 246.1494. Found $[M+H]^+$ 246.1487.

***N,N*-Diisopropylbenzo[*b*]thiophene-2-carboxamide (1n)**

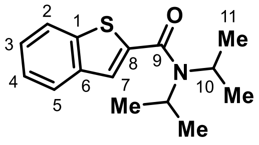

1n

General Procedure A: Benzothiophene-2-carboxylic acid (2.00 g, 11.2 mmol), diisopropylamine (2.10 mL, 14.6 mmol), oxalyl chloride (1.25 mL, 14.6 mmol) and Et_3N (2.34 mL, 16.8 mmol) were employed. Purification by FCC (10% EtOAc/Hex) afforded the title compound (2.36 g, 81%) as a colorless solid; m.p. 65–67 °C (hexane); ν_{max}/cm^{-1}: 2958 (br), 1622 (m), 1317 (m); ^{1}H NMR (500 MHz, $CDCl_3$): δ 7.90–7.77 (2H, m, C**2**-H, C**5**-H), 7.45–7.36 (3H, m, C**3**-H, C**4**-H, C**7**-H), 3.95 (2H, br, C**10**-H), 1.39 (12H, C**11**-H); ^{13}C NMR (126 MHz, $CDCl_3$): δ 164.2 (C**9**), 139.7 (C), 139.4 (C), 138.9 (C), 125.3 (C**2**), 124.7 (C**5**), 124.8 (C**3** or C**4**), 122.8 (C**7**), 122.3 (C**3** or C**4**), 48.6 (C**10**), 20.9 (C**11**); HRMS: (ESI^+) calculated for $C_{15}H_{20}NOS$ 262.1266. Found $[M+H]^+$ 262.1264.

***N,N*-Diisopropylbenzamide (1o)**

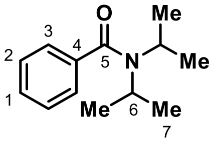

1o

To a solution of diisopropylamine (1.46 mL, 10.4 mmol) and Et_3N (1.67 mL, 12.0 mmol) in CH_2Cl_2 (20.0 mL) at 0 °C was added dropwise benzoyl chloride (0.93 mL, 8.00 mmol) and the mixture stirred at r.t. for 16 h. H_2O (20.0 mL) was added and the layers separated. The aqueous layer was extracted with CH_2Cl_2 (3 × 20.0 mL) and the organic layers combined and washed with brine (20.0 mL), dried (Na_2SO_4), filtered and concentrated *in vacuo*. Purification by FCC (20% EtOAc/Hex) afforded the title compound (1.51 g, 92%) as a colorless solid; m.p. 69–71 °C (hexane); ^{1}H NMR (500 MHz, $CDCl_3$): 7.39–7.34 (3H, m, ArH), 7.31 (2H, m, ArH), 3.66 (2H, br, C**6**-H), 1.33 (12H, br, C**7**-H); ^{13}C NMR (126 MHz, $CDCl_3$): δ 171.0 (C**5**), 139.1 (C**4**), 128.6 (ArC), 128.4 (ArC), 125.6 (ArC), 50.6 (C**6**, br), 46.1 (C**6**, br), 20.7 (C**7**).

The spectroscopic properties for this compound were consistent with the data available in the literature.[9]

Intramolecular Isopropyl N→C Alkyl Transfer Reactions: Products (Table 1A)

N,3-Diisopropylfuran-2-carboxamide (4a)

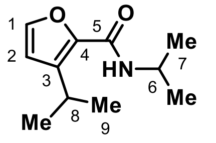

4a

General Procedure B: Compound **1a** (19.5 mg, 0.10 mmol), [Ir(cod)$_2$]BARF (6.40 mg, 0.005 mmol), **L-4** (5.30 mg, 0.005 mmol) and 1,2-DCB (0.10 mL) were employed at 120 °C for 72 h. Purification by FCC (5–20% EtOAc/Hex) afforded the title compound (18.5 mg, 95%) as a colorless oil; ν_{max}/cm^{-1}: 3301 (m), 2970 (m), 1643 (s); ^{1}H NMR (500 MHz, CDCl$_3$): δ 7.27 (1H, d, *J* = 1.8 Hz, C**1**-H), 6.44 (1H, d, *J* = 1.8 Hz, C**2**-H), 6.14 (1H, br, NH), 4.26 (1H, dhept, *J* = 8.1, 6.5 Hz, C**6**-H), 3.83 (1H, hept, *J* = 6.9 Hz, C**8**-H), 1.27 (6H, d, *J* = 6.5 Hz, C**7**-H), 1.22 (6H, d, *J* = 6.9 Hz, C**9**-H); ^{13}C NMR (125 MHz, CDCl$_3$): δ 158.8 (C**5**), 142.2 (C**1**), 140.9 (C**4**) 138.5 (C**3**), 111.2 (C**2**), 40.7 (C**6**), 24.2 (C**8**), 23.1 (C**9**), 22.9 (C**7**); HRMS: (ESI$^+$) calculated for $C_{11}H_{18}NO_2$ 196.1332. Found [M+H]$^+$ 196.1329.

3-Isopropyl-*N*-methylfuran-2-carboxamide (4b)

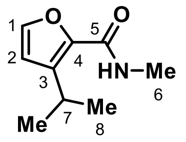

4b

General Procedure B: Compound **1b** (16.7 mg, 0.10 mmol), [Ir(cod)$_2$]BARF (6.40 mg, 0.005 mmol), **L-4** (5.30 mg, 0.005 mmol) and 1,2-DCB (0.10 mL) were employed at 120 °C for 72 h. Purification by FCC (20% EtOAc/Hex) afforded the title compound (16.2 mg, 97%) as a colorless oil; ν_{max}/cm^{-1}: 3439 (br), 2968 (w), 1644 (m); ^{1}H NMR (500 MHz, CDCl$_3$): δ 7.30 (1H, d, *J* = 1.8 Hz, C**1**-H), 6.46 (1H, d, *J* = 1.8 Hz, C**2**-H), 6.34 (1H, br, NH), 3.83 (1H, hept, *J* = 6.9 Hz, C**7**-H), 2.97 (3H, d, *J* = 5.0 Hz, C**6**-H), 1.22 (6H, d, *J* = 6.9 Hz, C**8**-H); ^{13}C NMR (126 MHz, CDCl$_3$): δ 160.3 (C**5**), 142.4 (C**1**), 140.9 (C**4**), 138.6 (C**3**), 111.1 (C**2**), 25.5 (C**6**), 24.2 (C**7**), 23.1 (C**8**); HRMS: (ESI$^+$) calculated for $C_9H_{14}NO_2$ 168.1025. Found [M+H]$^+$ 168.1020.

N-Ethyl-3-isopropylfuran-2-carboxamide (4c)

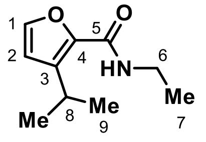

4c

General Procedure B: Compound **1c** (18.1 mg, 0.10 mmol), [Ir(cod)$_2$]BARF (6.40 mg, 0.005 mmol), **L-4** (5.30 mg, 0.005 mmol) and 1,2-DCB (0.10 mL) were employed at 120 °C for 72 h. Purification by FCC (5–10%

EtOAc/Hex) afforded the title compound (17.7 mg, 98%) as a colorless oil; ν_{max}/cm^{-1}: 3284 (br), 2967 (w), 1631 (m), 1522 (m); ^{1}H NMR (500 MHz, $CDCl_3$): δ 7.27 (1H, br, C**1**-H), 6.43 (1H, br, C**2**-H), 6.30 (1H, br, NH), 3.80 (1H, hept, J = 6.9 Hz, C**8**-H), 3.48–3.39 (2H, m, C**6**-H), 1.22 (3H, t, J = 7.3 Hz, C7-H), 1.19 (3H, d, J = 6.9 Hz, C**9**-H); ^{13}C NMR (126 MHz, $CDCl_3$): δ 159.5 (C**5**), 142.3 (C**1**), 140.9 (C**4**), 138.6 (C**3**), 111.1 (C**2**), 33.7 (C**6**), 24.2 (C**8**), 23.1 (C**9**), 15.0 (C7); HRMS: (ESI$^+$) calculated for $C_{10}H_{16}NO_2$ 182.1181. Found [M+H]$^+$ 182.1176.

***N*-Cyclohexyl-3-isopropylfuran-2-carboxamide (4d)**

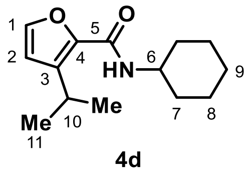

4d

General Procedure B: Compound **1d** (23.5 mg, 0.10 mmol), [Ir(cod)$_2$]BARF (6.40 mg, 0.005 mmol), **L-4** (5.30 mg, 0.005 mmol) and 1,2-DCB (0.10 mL) were employed at 120 °C for 72 h. Purification by FCC (10% EtOAc/Hex) afforded the title compound (22.3 mg, 97%) as a colorless oil; ν_{max}/cm^{-1}: 3434 (br), 2930 (w), 1639 (m), 1520 (m); ^{1}H NMR (500 MHz, $CDCl_3$): δ 7.27 (1H, d, J = 1.8 Hz, C**1**-H), 6.43 (1H, d, J = 1.8 Hz, C**2**-H), 3.92 (1H, tdt, J = 10.7, 8.1, 4.1 Hz, C**6**-H), 3.79 (1H, hept, J = 6.9 Hz, C**10**-H), 2.00–1.95 (2H, m, C**7**-H), 1.74 (2H, dqt J = 11.9, 4.1 Hz, C**8**-H), 1.65–1.60 (2H, m, C**9**-H), 1.44–1.35 (2H, m, C**8**-H), 1.26–1.15 (8H, m, C7-H, C**11**-H); ^{13}C NMR (126 MHz, $CDCl_3$): δ 158.7 (C**5**), 142.2 (C**1**), 141.0 (C**4**), 138.5 (C**3**), 111.2 (C**2**), 47.6 (C**6**), 33.3 (C**7**), 25.6 (C**9**), 24.9 (C**8**), 24.2 (C**10**), 23.1 (C**11**); HRMS: (ESI$^+$) calculated for $C_{14}H_{22}NO_2$ 236.1651. Found [M+H]$^+$ 236.1645.

3-(*tert*-Butyl)-*N*-isopropylfuran-2-carboxamide (4e')

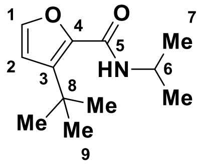

4e'

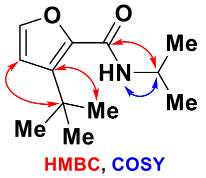

General Procedure B: Compound **1e** (20.9 mg, 0.10 mmol), [Ir(cod)$_2$]BARF (6.40 mg, 0.005 mmol), **L-4** (5.30 mg, 0.005 mmol) and 1,2-DCB (0.10 mL) were employed at 120 °C for 72 h. Purification by FCC (10–20% EtOAc/Hex) afforded the title compound (13.0 mg, 62%) as a colorless oil; ν_{max}/cm^{-1}: 3440 (br), 2965 (w), 1655 (s), 1517 (m); ^{1}H NMR (500 MHz, $CDCl_3$): δ 7.24 (1H, d, J = 1.8 Hz, C**1**-H), 6.43 (1H, d, J = 1.8 Hz, C**2**-H), 6.26 (1H, br, NH), 4.21 (1H, m, C**6**-H), 1.41 (9H, s, C9-H), 1.24 (6H, d, J = 6.6 Hz, C**7**-H); ^{13}C NMR (126 MHz, $CDCl_3$): δ 158.4 (C**5**), 142.0 (C**4**), 140.8 (C**3**), 140.7 (C**1**), 112.7 (C**2**), 41.0 (C**6**), 30.9 (C**8**), 30.0 (C**9**), 22.9 (C**7**); HRMS: (ESI$^+$) calculated for $C_{12}H_{20}NO_2$ 210.1494. Found [M+H]$^+$ 210.1491.

The selective transfer of the *t*-Bu group was confirmed by 2D NMR analysis: (1) an HMBC correlation was observed between (a) C9-H and C3; (b) C2-H and C8; (c) C6-H and C5; (2) a COSY correlation was observed between NH and CH-6.

N,3-Diisopropyl-4,5-dimethylfuran-2-carboxamide (4f)

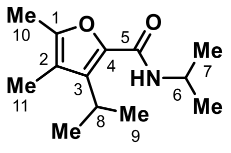

4f

General Procedure B: Compound **1f** (22.3 mg, 0.10 mmol), [Ir(cod)$_2$]BARF (6.40 mg, 0.005 mmol), **L-4** (5.30 mg, 0.005 mmol) and 1,2-DCB (0.10 mL) were employed at 120 °C for 72 h. Purification by FCC (5–10% EtOAc/Hex) afforded the title compound (15.2 mg, 68%) as a colorless oil (which solidified upon standing); ν_{max}/cm^{-1}: 3268 (br), 2966 (w), 1629 (s), 1526 (m); ^{1}H NMR (500 MHz, CDCl$_3$): δ 6.10 (1H, br, NH), 4.21 (1H, dhept, J = 8.1, 6.5 Hz, C**6**-H), 3.91 (1H, hept, J = 7.2 Hz, C**8**-H), 2.20 (3H, s, C**10**-H), 1.98 (3H, s, C**11**-H), 1.25 (6H, d, J = 7.2 Hz, C**9**-H), 1.23 (6H, d, J = 6.5 Hz, C**7**-H); ^{13}C NMR (126 MHz, CDCl$_3$): δ 159.2 (C**5**), 148.7 (C**1**), 139.0 (C**4**), 137.9 (C**3**), 116.9 (C**2**), 40.6 (C**6**), 24.3 (C**8**), 23.0 (C**7**), 21.2 (C**9**), 11.6 (C**10**), 10.0 (C**11**); HRMS: (ESI$^+$) calculated for $C_{13}H_{22}NO_2$ 224.1651. Found [M+H]$^+$ 224.1645.

N,3-Diisopropyl-5-methylfuran-2-carboxamide (4g)

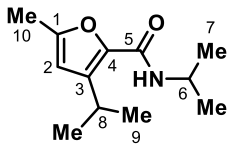

4g

General Procedure B: Compound **1g** (20.9 mg, 0.10 mmol), [Ir(cod)$_2$]BARF (6.40 mg, 0.005 mmol), **L-4** (5.30 mg, 0.005 mmol) and 1,2-DCB (0.10 mL) were employed at 120 °C for 72 h. Purification by FCC (5–10% EtOAc/Hex) afforded the title compound (15.0 mg, 72%) as a colorless oil (which solidified upon standing); ν_{max}/cm^{-1}: 3303 (br), 2929 (m), 1638 (s), 1519 (s); ^{1}H NMR (500 MHz, CDCl$_3$): δ 6.06–6.04 (2H, m, NH, C**2**-H), 4.22 (1H, dhept, J = 8.2, 6.5 Hz, C**6**-H), 3.75 (1H, hept, J = 7.0 Hz, C**8**-H), 2.29 (3H, d, J = 0.9 Hz, C**10**-H), 1.23 (6H, d, J = 6.5 Hz, C**7**-H), 1.16 (6H, d, J = 7.0 Hz, C**9**-H); ^{13}C NMR (126 MHz, CDCl$_3$): δ 159.0 (C**5**), 152.6 (C**1**), 140.0 (C**3**), 139.4 (C**4**), 107.6 (C**2**), 40.6 (C**6**), 24.0 (C**8**), 23.1 (C**9**), 23.0 (C**7**), 13.8 (C**10**); HRMS: (ESI$^+$) calculated for $C_{12}H_{20}NO_2$ 210.1494. Found [M+H]$^+$ 210.1494.

4,5-Dibromo-N,3-diisopropylfuran-2-carboxamide (4h)

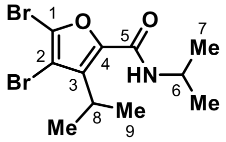

4h

General Procedure B: Compound **1h** (35.3 mg, 0.10 mmol), [Ir(cod)$_2$]BARF (6.40 mg, 0.005 mmol), **L-4** (5.30 mg, 0.005 mmol) and 1,2-DCB (0.10 mL) were employed at 120 °C for 72 h. Purification by FCC (0–20% EtOAc/Hex) afforded the title compound (6.70 mg, 19%) as a colorless oil; ν_{max}/cm^{-1}: 3312 (br), 2967 (w), 1638

(s), 1531 (m); ^{1}H NMR (500 MHz, $CDCl_3$): δ 6.82 (1H, br, N<u>H</u>), 4.20 (1H, dhept, *J* = 8.0, 6.7 Hz, C**6**-<u>H</u>), 3.96 (1H, hept, *J* = 7.2 Hz, C**8**-<u>H</u>), 1.32 (6H, d, *J* = 6.7 Hz, C**7**-<u>H</u>), 1.25 (6H, d, *J* = 7.2 Hz, C**9**-<u>H</u>); ^{13}C NMR (126 MHz, $CDCl_3$): δ 163.0 (C**5**), 151.0 (C**4**), 124.9 (C**1**), 123.9 (C**2**), 103.0 (C**3**), 41.2 (C**6**), 24.8 (C**8**), 22.8 (C**9**), 20.8 (C**7**); HRMS: (ESI$^+$) calculated for $C_{11}H_{16}{}^{79}BrNO_2$ 351.9548. Found $[M+H]^+$ 351.9540.

5-Chloro-*N*,3-diisopropylfuran-2-carboxamide (4i)

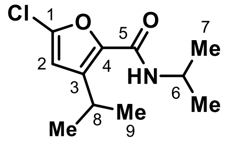

4i

General Procedure B: Compound **1i** (23.0 mg, 0.10 mmol), [Ir(cod)$_2$]BARF (6.40 mg, 0.005 mmol), **L-4** (5.30 mg, 0.005 mmol) and 1,2-DCB (0.10 mL) were employed at 120 °C for 72 h. Purification by FCC (5–20% EtOAc/Hex) afforded the title compound (6.44 mg, 28%) as a colorless oil (which solidified upon standing); ν_{max}/cm^{-1}: 3319 (br), 2966 (w), 1640 (s), 1524 (m); ^{1}H NMR (500 MHz, $CDCl_3$): δ 6.24 (1H, s, C**2**-<u>H</u>), 6.01 (1H, br, N<u>H</u>), 4.22 (1H, dhept, *J* = 8.1, 6.6 Hz, C**6**-<u>H</u>), 3.78 (1H, hept, *J* = 6.9 Hz, C**8**-<u>H</u>), 1.24 (6H, d, *J* = 6.6 Hz, C**8**-<u>H</u>), 1.17 (6H, d, *J* = 6.9 Hz, C**6**-<u>H</u>); ^{13}C NMR (126 MHz, $CDCl_3$): δ 157.7 (C**5**), 140.9 (C**1**), 140.5 (C**4**), 136.8 (C**3**), 108.1 (C**2**), 40.9 (C**6**), 24.4 (C**8**), 22.9 (C**8**), 22.9 (C**6**); HRMS: (ESI$^+$) calculated for $C_{11}H_{17}{}^{35}ClNO_2$ 230.0948. Found $[M+H]^+$ 230.0943.

***N*,3-Diisopropyl-5-phenylfuran-2-carboxamide (4j)**

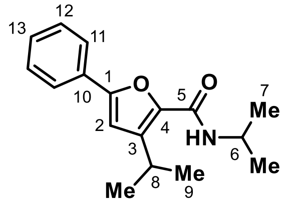

4j

General Procedure B: Compound **1j** (27.1 mg, 0.10 mmol), [Ir(cod)$_2$]BARF (12.8 mg, 0.01 mmol), **L-4** (10.6 mg, 0.01 mmol) and 1,2-DCB (0.10 mL) were employed at 120 °C for 72 h. Purification by FCC (10–30% EtOAc/Hex) afforded the title compound (23.0 mg, 85%) as a colorless oil (which solidified upon standing); ν_{max}/cm^{-1}: 2969 (m), 1762 (m), 1639 (s), 1450 (m); ^{1}H NMR (500 MHz, $CDCl_3$): δ 7.70–7.67 (2H, m, C**11**-<u>H</u>), 7.43–7.40 (2H, m, C**12**-<u>H</u>), 7.32 (1H, t, *J* = 7.5 Hz, C**13**-<u>H</u>), 6.70 (1H, s, C**2**-<u>H</u>), 6.15 (1H, br, N<u>H</u>), 4.28 (1H, m, C**6**-<u>H</u>), 3.83 (1H, m, C**8**-<u>H</u>), 1.29 (6H, d, *J* = 6.5 Hz, C**7**-<u>H</u>), 1.24 (6H, d, *J* = 6.9 Hz, C**9**-<u>H</u>); ^{13}C NMR (126 MHz, $CDCl_3$): δ 168.8 (C**5**), 158.8 (C**4**), 153.4 (C**1**), 140.8 (C**10**), 129.9 (C**3**), 128.8 (C**12**), 128.8 (C**13**), 124.4 (C**11**), 124.4 (C**2**), 40.8 (C**6**), 24.4 (C**8**), 23.1 (C**7**), 23.0 (C**9**); HRMS: (ESI$^+$) calculated for $C_{17}H_{22}NO_2$ 272.1651. Found $[M+H]^+$ 272.1650.

N,3-Diisopropyl-1-methyl-1H-pyrrole-2-carboxamide (4k)

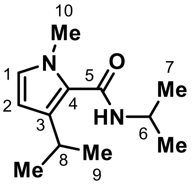

4k

General Procedure B: Compound **1k** (20.8 mg, 0.10 mmol), [Ir(cod)$_2$]BARF (6.40 mg, 0.005 mmol), **L-4** (5.30 mg, 0.005 mmol) and 1,2-DCB (0.10 mL) were employed at 130 °C for 72 h. Purification by FCC (10–40% EtOAc/Hex) afforded the title compound (14.8 mg, 71%) as a colorless oil; ν_{max}/cm^{-1}: 3302 (br), 2929 (br), 1640 (m), 1519 (m); ^{1}H NMR (500 MHz, CDCl$_3$): δ 6.58 (1H, d, J = 2.7 Hz, C**1**-H), 5.99 (1H, d, J = 2.7 Hz, C**2**-H), 5.51 (1H, br, NH), 4.24 (1H, m, C**6**-H), 3.76 (3H, s, C**10**-H), 3.05 (1H, m, C**8**-H), 1.24 (12H, dd, J = 6.7, 4.3 Hz, C**7**-H, C**9**-H,); ^{13}C NMR (126 MHz, CDCl$_3$): 162.1 (C**5**), 133.3 (C**4**), 125.5 (C**1**), 123.4 (C**3**), 104.8 (C**2**), 41.4 (C**10**), 36.1 (C**16**), 26.3 (C**8**), 24.5 (C**7**), 23.0 (C**9**); HRMS: (ESI$^+$) calculated for $C_{12}H_{21}NO_2$ 209.1654. Found [M+H]$^+$ 209.1652.

N,3-Diisopropylthiophene-2-carboxamide (4l)

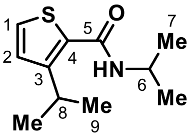

4l

General Procedure B: Compound **1l** (21.1 mg, 0.10 mmol), [Ir(cod)$_2$]BARF (6.40 mg, 0.005 mmol), **L-4** (5.30 mg, 0.005 mmol) and 1,2-DCB (0.10 mL) were employed at 120 °C for 48 h. Purification by FCC (10–20% EtOAc/Hex) afforded the title compound (15.4 mg, 73%) as a colorless oil; ν_{max}/cm^{-1}: 3260 (m), 2970 (m), 1619 (s); ^{1}H NMR (500 MHz, CDCl$_3$): δ 7.24 (1H, d, J = 5.2 Hz, C**1**-H), 7.04 (1H, d, J = 5.2 Hz, C**2**-H), 5.60 (1H, br, NH), 4.24 (1H, dhept, J = 7.9, 6.5 Hz, C**6**-H), 3.79 (1H, hept, J = 6.9 Hz, C**8**-H), 1.27 (12H, dd, J = 6.7, 2.1 Hz, C**7**-H, C**9**-H); ^{13}C NMR (126 MHz, CDCl$_3$): δ 162.5 (C**5**), 152.9 (C**1**), 129.6 (C**4**), 127.6 (C**2**) 125.9 (C**3**), 42.0 (C**6**), 27.9 (C**8**) 23.7 (C**9**), 22.8 (C7); HRMS: (ESI$^+$) calculated for $C_{11}H_{18}NOS$ 211.1104. Found [M+H]$^+$ 212.1101.

N,3-Diisopropylbenzofuran-2-carboxamide (4m)

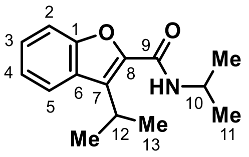

4m

General Procedure B: Compound **1m** (24.5 mg, 0.10 mmol), [Ir(cod)$_2$]BARF (12.8 mg, 0.01 mmol), **L-4** (10.6 mg, 0.01 mmol) and 1,2-DCB (0.10 mL) were employed at 120 °C for 72 h. Purification by FCC (0–40%

EtOAc/Hex) afforded the title compound (13.5 mg, 55%) as a colorless solid; m.p. 125 –128 °C (EtOH); ν_{max}/cm^{-1}: 3323 (br), 2967 (w), 1642 (s), 1523 (m); ^{1}H NMR (500 MHz, $CDCl_3$): δ 8.01 (1H, m, C**5**-H), 7.80 (1H, m, C**2**-H), 7.40–7.35 (2H, m, C**3**-H, C**4**-H), 5.75 (1H, br, NH), 4.25 (1H, m, C**10**-H), 4.01 (1H, hept, *J* = 7.2 Hz, C**12**-H), 1.49 (6H, d, *J* = 7.2 Hz, C**11**-H), 1.27 (6H, d, *J* = 6.6 Hz, C**13**-H); ^{13}C NMR (126 MHz, $CDCl_3$): δ 163.4 (C**5**), 144.8 (C**7**), 138.9 (C**6**), 138.8 (C**1**), 129.9 (C**8**), 125.6 (C**3** or C**4**), 124.6 (C**5**), 124.2 (C**3** or C**4**), 122.9 (C**2**), 42.3 (C**10**), 28.2 (C**12**), 22.8 (C**13**), 21.7 (C**11**); HRMS: (ESI^{+}) calculated for $C_{15}H_{20}NO_2$ 246.1494. Found [M+H]$^{+}$ 246.1498.

***N*,3-Diisopropylbenzo[*b*]thiophene-2-carboxamide (4n)**

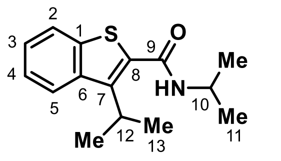

4n

General Procedure B: Compound **1n** (26.1 mg, 0.10 mmol), [Ir(cod)$_2$]BARF (12.8 mg, 0.01 mmol), **L-4** (10.6 mg, 0.01 mmol) and 1,2-DCB (0.10 mL) were employed at 120 °C for 72 h. Purification by FCC (0–40% EtOAc/Hex) afforded the title compound (13.6 mg, 52%) as a colorless solid; m.p. 123–126 °C (EtOH); ν_{max}/cm^{-1}: 3308 (br), 2967 (w), 1634 (s), 1336 (m); ^{1}H NMR (500 MHz, $CDCl_3$): δ 7.81 (1H, dt, *J* = 8.0, 1.0 Hz, C**5**-H), 7.45 (1H, dt, *J* = 8.4, 1.0 Hz, C**2**-H), 7.38 (1H, ddd, *J* = 8.4, 7.2, 1.0 Hz, C**3**-H), 7.26-7.21 (1H, m, C**4**-H), 6.50 (1H, d, *J* = 7.9 Hz, NH), 4.35–4.24 (2H, m, C**10**-H, C**12**-H), 1.44 (6H, d, *J* = 7.1 Hz, C**11**-H), 1.30 (6H, d, *J* = 6.6 Hz, C**13**-H); ^{13}C NMR (126 MHz, $CDCl_3$): δ 159.5 (C**5**), 153.5 (C**1**), 141.4 (C**8**), 132.1 (C**7**), 127.8 (C**6**), 126.5 (C**3**), 123.0 (C**5**), 122.7 (C**4**), 111.8 (C**2**), 41.1 (C**10** or C**12**), 24.7 (C**10** or C**12**), 22.9 (C**10**), 22.1 (C**12**); HRMS: (ESI^{+}) calculated for $C_{15}H_{20}NOS$ 262.1266. Found [M+H]$^{+}$ 262.1259.

***N*,2-Diisopropylbenzamide (4o)**

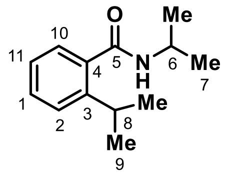

4o

General Procedure B: Compound **1o** (20.5 mg, 0.10 mmol), [Ir(cod)$_2$]BARF (12.8 mg, 0.01 mmol), **L-4** (10.6 mg, 0.01 mmol) and 1,2-DCB (0.10 mL) were employed at 120 °C for 72 h. Purification by FCC (0–50% EtOAc/Hex) afforded the title compound (13.8 mg, 67%) as a colorless oil; ν_{max}/cm^{-1}: 2973 (w), 1745 (m), 1463 (m); ^{1}H NMR (500 MHz, $CDCl_3$): δ 7.38–7.35 (2H, m, C**10**-H, C**1**-H), 7.30 (1H, m, C**2**-H), 7.18 (1H, m, C**11**-H), 5.53 (1H, br, NH), 4.30 (1H, m, C**6**-H), 3.34 (1H, hept, *J* = 6.9 Hz, C**8**-H), 1.25 (12H, dd, *J* = 6.7 Hz, 2.2 Hz, C**13**-H, C**8**-H); ^{13}C NMR (126 MHz, $CDCl_3$): δ 176.7 (C**5**), 146.4 (C**3**), 129.8 (C**4**), 126.40 (C**1**), 126.0 (C**11**), 125.6 (C**10**), 125.6 (C**2**), 41.8 (C**6**), 29.9 (C**8**), 24.1 (C**7**), 22.8 (C**9**); HRMS: (ESI^{+}) calculated for $C_{13}H_{20}NO$ 206.1545. Found [M+H]$^{+}$ 206.1544.

Intramolecular N→C Alkyl Transfer Involving Other Alkyl Units: Substrates (Table 1B)

N-(*sec*-Butyl)-*N*-methylfuran-2-carboxamide (1p)

Synthesis of methyl sec-butylcarbamate

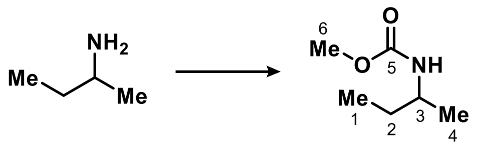

To a solution of butan-2-amine (1.00 mL, 12.4 mmol) in CH_2Cl_2 (30.0 mL) was added Et_3N (2.14 mL, 15.4 mmol) and DMAP (151 mg, 1.24 mmol) and the resulting mixture cooled to 0 °C. Methyl chloroformate (1.07 mL, 13.9 mmol) was added dropwise and the reaction mixture was then stirred at r.t. for 16 h under an atmosphere of N_2. The mixture was diluted with CH_2Cl_2 (30.0 mL) and H_2O (30.0 mL) was added. The organic layer was washed with 1 M aq. HCl (2 × 30.0 mL) and H_2O (30.0 mL), dried ($MgSO_4$), filtered and concentrated *in vacuo*, to afford the title compound as a colorless oil which was used directly without further purification; ^{1}H NMR (500 MHz, $CDCl_3$): δ 4.48 (1H, br, N<u>H</u>), 3.70 –3.60 (4H, m, C**3**-<u>H</u>, C**6**-<u>H</u>), 1.49–1.45 (2H, m, C**2**-<u>H</u>), 1.14 (3H, d, *J* = 6.6 Hz, C**4**-<u>H</u>), 0.92 (3H, d, *J* = 7.3 Hz, C**1**-<u>H</u>).

Synthesis of N-methylbutan-2-aminium chloride

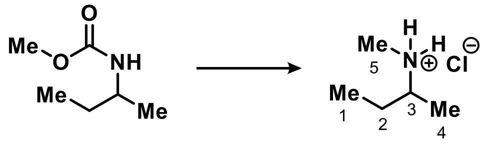

To a solution of $LiAlH_4$ (670 mg, 17.6 mmol) in Et_2O (30.0 mL) was added methyl *sec*-butylcarbamate (assuming 12.4 mmol) and the resulting mixture was stirred at 30 °C for 3 h then cooled to 0 °C. Et_2O (5.00 mL), H_2O (1.00 mL) and 15% w/v aq. NaOH (1.00 mL) were added and the resulting mixture warmed to r.t. and stirred for 15 min. $MgSO_4$ was added and the mixture stirred for a further 15 min before the insoluble salts were removed *via* filtration. The filtrate was subsequently cooled to 0 °C and 2 M HCl (in Et_2O) (24.8 mL, 49.6 mmol) added dropwise. The mixture was stirred at r.t. for 1 h and then concentrated *in vacuo* to yield the crude HCl salt, *N*-methylbutan-2-aminium chloride**,** as a colorless oil which was used directly without further purification; ^{1}H NMR (500 MHz, D_2O): δ 3.18 (1H, tq, *J* = 13.4, 7.1 Hz, C**3**-<u>H</u>), 2.67 (3H, s, C**5**-<u>H</u>), 1.76 (1H, dqd, *J* = 13.4, 7.6 Hz, C**2**-<u>H</u>), 1.58 (1H, m, C**2**-<u>H</u>), 1.28 (3H, d, *J* = 7.1 Hz, C**4**-<u>H</u>), 0.96 (3H, t, *J* = 7.6 Hz, C**1**-<u>H</u>).

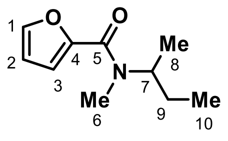

1p

General Procedure A: 2-Furoic acid (0.93 g, 8.27 mmol), *N*-methylbutan-2-aminium chloride (assuming 12.4 mmol), oxalyl chloride (0.91 mL, 10.75 mmol) and Et_3N (3.45 mL, 24.8 mmol) were employed. Purification by FCC (30–40% EtOAc/Hex) afforded the title compound (396 mg, 30%, 0.5:0.5 mixture of rotamers *A*:*B*) as a

colorless oil; ν_{max}/cm^{-1}: 3477 (br), 2967 (m), 1615 (s), 1405 (m); ^{1}H NMR (500 MHz, $CDCl_3$): δ 7.50 (1H, br, C**1**-H, *A*+*B*), 6.96 (1H, br, *J* = 3.5, C**3**-H, *A*+*B*), 6.48 (1H, dd, *J* = 3.5, 1.8 Hz, C**2**-H, *A*+*B*), 4.70 (0.5H, br, C**7**-H, *A*), 4.27 (0.5H, br, C**7**-H, *B*), 3.06 (1.5H, C**6**-H, *A*), 2.91 (1.5H, C**6**-H, *B*), 1.63–1.48 (2H, m, C**9**-H, *A*+*B*), 1.27–1.19 (3H, C**10**-H, *A*+*B*), 0.91–0.85 (3H, m, C**8**-H, *A*+*B*); ^{13}C NMR (126 MHz, $CDCl_3$): δ 168.5 (C**5**, *A*+*B*), 148.3 (C**4**, *A*+*B*), 143.6 (C**1**, *A*), 143.3 (C**1**, *B*), 116.0 (C**3**, *A*), 115.0 (C**3**, *B*), 111.1 (C**2**, *A*+*B*), 54.8 (C**7**, *B*), 50.9 (C**7**, *A*), 29.6 (C**6**, *B*), 27.5 (C**6**, *A*), 26.9 (C**8**, *B*), 26.5 (C**8**, *A*), 19.2 (C**10**, *A*), 18.9 (C**10**, *B*), 10.9 (C**9**, *A*+*B*); HRMS: (ESI$^+$) calculated for $C_{10}H_{16}NO_2$ 182.1181. Found [M+H]$^+$ 182.1176.

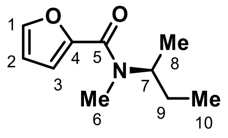

1p

The enantioenriched analogue of **1p** (>98:2 e.r.) was prepared *via* the same method as above by employing (*S*)-butan-2-amine; $[\alpha]_D^{20}$ +107.7 (c = 0.82, $CHCl_3$).

***N*-Methyl-*N*-(4-phenylbutan-2-yl)furan-2-carboxamide (1q)**

Synthesis of N-methyl-5-phenylbutan-2-amine

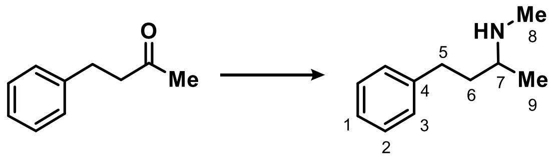

To a solution of benzylacetone (1.93 g, 11.9 mmol) and 40% methylamine in MeOH (3.53 mL, 35.7 mmol) was added $NaBH_4$ (541 mg, 14.3 mmol) at 0 °C and the resulting mixture stirred at r.t. for 16 h under an atmosphere of N_2. H_2O (20.0 mL) was added and the solution acidified with 1 M aq. HCl to a pH of 1. CH_2Cl_2 (20.0 mL) was then added, the layers separated, and the aqueous layer extracted with CH_2Cl_2 (3 × 20.0 mL). The aqueous layer was basified with 1 M aq. NaOH to a pH of 13 and Et_2O (20.0 mL) added. The layers were separated and the aqueous layer was extracted with Et_2O (3 × 20.0 mL). The organic layers were combined, dried ($MgSO_4$), filtered and concentrated *in vacuo* to afford the title compound as a colorless oil which was used directly without further purification; ^{1}H NMR (500 MHz, $CDCl_3$): 7.32–7.29 (2H, m, ArH), 7.23–7.19 (3H, m, ArH), 2.73–2.57 (2H, m, C**5**-H), 2.43 (3H, s, C**8**-H), 1.82 (1H, m, C**6**-H), 1.65 (1H, m, C**6**-H), 1.43 (1H, br, NH), 1.13 (3H, d, *J* = 6.3 Hz, C**9**-H).

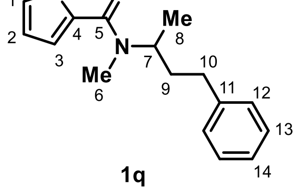

1q

General Procedure A: 2-Furoic acid (0.660 g, 5.89 mmol), *N*-methyl-5-phenylbutan-2-amine (1.25 g, 7.66 mmol), oxalyl chloride (0.66 mL, 7.66 mmol), and Et_3N (1.23 mL, 8.83 mmol) were employed. Purification by

FCC (0–30% EtOAc/Hex) afforded the title compound (1.03 g, 61%, 0.54:0.46 mixture of rotamers *A*:*B*) as a colorless oil; ν_{max}/cm^{-1}: 2978 (w), 1615 (s), 1615 (s), 1402 (m); ^{1}H NMR (500 MHz, $CDCl_3$): δ 7.46 (1H, br, C**1**-H, *A*+*B*), 7.28–7.12 (5H, m, ArH), 6.94 (1H, br, C**3**-H, *A*+*B*), 6.46 (1H, br, C**2**-H, *A*+*B*), 4.87 (0.54, br, C**7**-H, *A*), 4.42 (0.46, br, C**7**-H, *B*), 3.09 (3H, br, C**6**-H), 2.76–2.61 (2H, br, C**10**-H), 2.02 (1H, br, C**9**-H), 1.88 (1H, m, C**9**-H), 1.38–1.31 (3H, br, C**8**-H); ^{13}C NMR (126 MHz, $CDCl_3$): δ 161.1 (C**5**, *B*), 160.6 (C**5**, *A*), 148.5 (C**4**, *A*), 148.2 (C**4**, *B*), 143.7 (C**1**, *A*), 143.4 (C**1**, *B*), 141.8 (C**11**, *B*), 141.1 (C**11**, *A*), 128.4 (ArCH), 128.3 (ArCH), 125.9 (ArCH), 116.1 (C**3**, *A*), 115.4 (C**3**, *B*), 111.1 (C**2**, *A*+*B*), 52.4 (C**7**, *B*), 49.4 (C**7**, *A*), 36.2 (C**9**, *B*), 35.5 (C**9**, *A*), 33.0 (C**10**, *A*), 32.6 (C**10**, *B*), 29.8 (C**6**, *A*), 27.0 (C**6**, *B*), 19.1 (C**8**, *B*), 17.8 (C**8**, *A*); HRMS: (ESI$^+$) calculated for $C_{16}H_{20}NO_2$ 258.1494. Found $[M+H]^+$ 258.1504.

***N*-Methyl-*N*-(octan-2-yl)furan-2-carboxamide (1r)**

Synthesis of N-methyloctan-2-amine

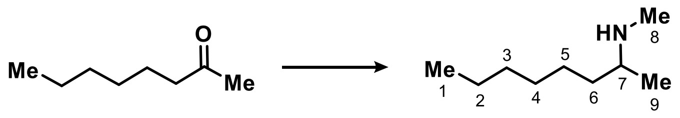

2-Octanone (2.00 mL, 12.8 mmol) and 2 M methylamine in MeOH (1.52 mL, 15.4 mmol) were stirred in DCE (40.0 mL) at r.t. for 1 h and then sodium triacetoxyborohydride (3.80 g, 17.9 mmol) was added along with acetic acid (0.73 mL, 12.8 mmol). The resulting mixture was stirred at r.t. for 16 h, under an atmosphere of N_2, before being quenched with 1 M aq. NaOH (40.0 mL). EtOAc (40.0 mL) was added and the layers separated. The aqueous layer was extracted with EtOAc (3 × 40.0 mL), the organic layers combined and washed with brine (40.0 mL), then dried ($MgSO_4$) and concentrated *in vacuo* to afford the title compound as a colorless oil which was used directly without further purification; ^{1}H NMR (500 MHz, $CDCl_3$): 2.52 (1H, m, C**7**-H), 2.42 (3H, s, C**8**-H), 1.34–1.26 (10H, m, CH_2), 1.04 (3H, d, J = 6.4 Hz, C**9**-H), 0.92-0.89 (3H, m, C**1**-H).

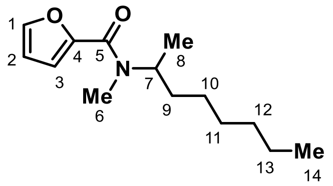

1r

General Procedure A: 2-Furoic acid (1.10 g, 9.80 mmol), *N*-methyloctan-2-amine (assuming 12.8 mmol), oxalyl chloride (1.08 mL, 12.7 mmol) and Et_3N (2.05 mL, 14.7 mmol) were employed. Purification by FCC (0–20% EtOAc/Hex) afforded the title compound (994 mg, 45%, 0.5:0.5 mixture of rotamers *A*:*B*) as a colorless oil; ν_{max}/cm^{-1}: 2928 (m), 1619 (s), 1403 (m); ^{1}H NMR (500 MHz, $CDCl_3$): δ 7.47 (1H, br, C**1**-H, *A*+*B*), 6.90 (1H, br, C**3**-H, *A*+*B*), 6.46 (1H, dd, J = 3.5, 1.8 Hz, C**2**-H, *A*+*B*), 4.75 (0.5H, br, C**7**-H, *A*), 4.33 (0.5H, br, C**7**-H, *B*), 2.95 (3H, br, C**6**-H, *A*+*B*), 1.64–1.52 (2H, br, C**9**-H, *A*+*B*), 1.43 (1H, br, CH_2, *A*+*B*), 1.24 (10H, br, CH_2 and C**8**-H, *A*+*B*), 0.86 (3H, t, J = 6.8 Hz, C**14**-H, *A*+*B*); ^{13}C NMR (126 MHz, $CDCl_3$): δ 161.1 (C**5**, *A*), 160.4 (C**5**, *B*), 148.5 (C**4**, *A*+*B*), 143.6 (C**1**, *A*), 143.2 (C**1**, *B*), 115.9 (C**3**, *A*), 114.9 (C**3**, *B*), 111.1 (C**2**, *A*+*B*), 53.1 (C**7**, *A*), 49.3 (C7, *B*), 34.5 (C**9**, *A*), 33.6 (C**9**, *B*), 31.7 (CH_2, *A*+*B*), 29.7 (C**6**, *A*+*B*), 29.1 (CH_2, *A*+*B*), 26.8 (CH_2, *A*) 26.4 (CH_2, *B*),

22.6 (C̲H$_2$, *A*+*B*), 19.2 (C**8**, *A*), 17.7 (C**8**, *B*), 14.1 (C**14**, *A*+*B*); HRMS: (ESI$^+$) calculated for $C_{14}H_{24}NO_2$ 238.1807. Found [M+H]$^+$ 238.1813.

***N*-Methyl-*N*-(3-methylbutan-2-yl)furan-2-carboxamide (1s)**

Synthesis of N-3-dimethylbutan-2-aminium chloride

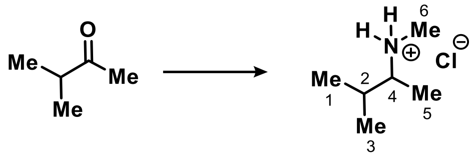

Titanium (IV) isopropoxide (15.8 mL, 52.8 mmol) was added to 40% methylamine in MeOH (12.0 mL, 121 mmol) and then 3-methylbutanone (4.31 mL, 40.0 mmol) was added dropwise at 0 °C under an atmosphere of N_2. The resulting solution was stirred at r.t. for 16 h, before $NaBH_4$ (1.51 g, 40.0 mmol) was added and the mixture stirred for a further 24 h. H_2O (20.0 mL) was added and the precipitate removed by filtration, then washed with Et_2O (20.0 mL). The resulting layers of the filtrate were separated and the aqueous layer extracted with Et_2O (3 × 20.0 mL). The combined organic layers were cooled to 0 ºC and 2 M aq. HCl (in Et_2O) (60.0 mL, 120 mmol) was added dropwise. The mixture was stirred at r.t. for 1 h and then concentrated *in vacuo* to yield the crude HCl salt, *N*-3-dimethylbutan-2-aminium chloride, as an orange solid, which was used directly without further purification; ^{1}H NMR (500 MHz, D_2O): δ 3.04 (1H, m, C**4**-H̲), 2.61 (3H, s, C**6**-H̲), 1.95 (1H, qqd, *J* = 6.8, 6.8, 4.8 Hz, C**2**-H̲), 1.14 (3H, d, *J* = 6.8 Hz, C**5**-H̲), 0.90 (3H, d, *J* = 6.8 Hz, C**1**-H̲ or C**3**-H̲), 0.86 (3H, d, *J* = 6.8 Hz, C**1**-H̲ or C**3**-H̲).

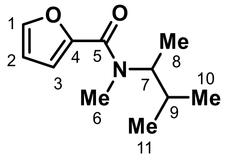

1s

General Procedure A: 2-Furoic acid (87.4 mg, 0.78 mmol), *N*-3-dimethylbutan-2-aminium chloride (assuming 1.01 mmol), oxalyl chloride (0.09 mL, 1.01 mmol) and Et_3N (0.43 mL, 3.12 mmol) were employed. Purification by FCC (20–40% EtOAc/Hex) afforded the title compound (105 mg, 69%, 0.6:0.4 mixture of rotamers *A*:*B*) as a colorless oil; ν_{max}/cm^{-1}: 29723 (m), 1634 (s), 1487 (m), 1364 (s); ^{1}H NMR (500 MHz, $CDCl_3$): δ 7.48 (1H, br, C**1**-H̲, *A*+*B*), 6.94 (1H, br, C**3**-H̲, *A*+*B*), 6.47 (1H, br, C**2**-H̲, *A*+*B*), 4.40 (0.6H, C**7**-H̲, *A*), 3.92 (0.4H, C**7**-H̲, *B*), 3.06 (1.8H, br, C**6**-H̲, *A*), 2.88 (1.2H, br, C**6**-H̲, *B*), 1.77 (1H, m, C**9**-H̲, *A*+*B*), 1.28–1.18 (3H, m, C**8**-H̲, *A*+*B*), 0.98–0.83 (6H, m, C**10**-H̲, C**11**-H̲, *A*+*B*); ^{13}C NMR (126 MHz, $CDCl_3$): δ 161.3 (C**5**, *B*), 160.5 (C**4**, *A*), 148.6 (C**4**, *A*+*B*), 143.6 (C**1**, *A*), 143.2 (C**1**, *B*), 115.9 (C**3**, *A*), 115.1 (C**3**, *B*), 111.1 (C**2**, *A*+*B*), 59.6 (C**7**, *B*), 55.6 (C**7**, *A*), 31.8 (C**9**, *B*), 31.1 (C**9**, *A*), 30.3 (C**6**, *A*), 27.4 (C**6**, *B*), 20.0 (C**10**, *A*+*B*), 20.0 (C**11**, *A*+*B*), 17.3 (C**8**, *B*), 15.9 (C**8**, *A*); HRMS: (ESI$^+$) calculated for $C_{11}H_{18}NO_2$ 196.1338. Found [M+H]$^+$ 196.1342.

N-(5-(Benzyloxy)pentan-2-yl)-*N*-methylfuran-2-carboxamide (1s)

Synthesis of 5-(benzyloxy)pentan-2-one

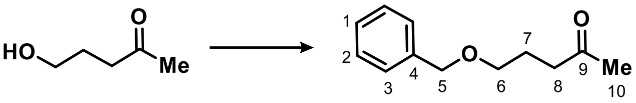

To 5-hydroxy-2-pentanone (2.00 mL, 19.7 mmol) was added benzyl chloride (2.60 mL, 21.9 mmol) and *N*-ethyldiisopropylamine (6.67 mL, 39.2 mmol) and the resulting mixture stirred at 150 °C for 2 h under an atmosphere of N_2. Et_2O (20.0 mL) and 10% aq. $NaHSO_4$ (20.0 mL) were added and aqueous layer extracted with Et_2O (3 × 20.0 mL). The organic layers were combined, dried ($MgSO_4$), filtered and concentrated *in vacuo*. Purification by FCC (20% EtOAc/Hex) afforded the title compound (2.52 g, 67%) as a yellow oil; ^{1}H NMR (500 MHz, $CDCl_3$): δ 7.30–7.20 (5H, m, ArH), 4.42 (2H, s, C**5**-H), 3.42 (1H, t, *J* = 6.1 Hz, C**6**-H), 2.49 (2H, t, *J* = 7.3 Hz, C**8**-H), 2.07 (3H, s, C**10**-H), 1.83 (2H, tt, *J* = 7.3, 6.1 Hz, C**7**-H); ^{13}C NMR (126 MHz, $CDCl_3$): δ 208.7 (C**9**), 138.4 (C**4**), 128.4 (ArCH), 127.7 (ArCH), 127.6 (ArCH), 77.9 (C**5**), 69.3 (C**6**), 40.4 (C**8**), 30.0 (C**10**), 23.9 (C**7**).

The spectroscopic properties for this compound were consistent with the data available in the literature.[10]

Synthesis of 5-(benzyloxy)-N-methylpentan-2-amine

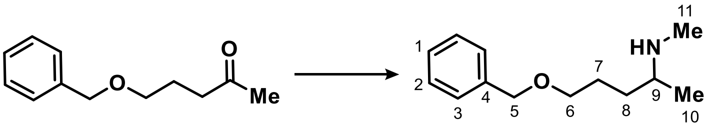

Titanium (IV) isopropoxide (5.06 mL, 16.9 mmol) was added to a solution of 2 M methylamine in MeOH (3.56 mL, 36.0 mmol), followed by 5-(benzyloxy)pentan-2-one (2.30 g, 12.0 mmol) and the resulting solution stirred at r.t. for 5 h. $NaBH_4$ (0.57 g, 15.0 mmol) was added and the mixture stirred for a further 16 h. H_2O (50.0 mL) was added, the precipitate removed by filtration, then washed with Et_2O (40.0 mL). The resulting layers were separated and the aqueous layer extracted with Et_2O (3 × 20.0 mL). The organic layers were combined, dried ($MgSO_4$), filtered and concentrated *in vacuo* to afford the title compound as a colorless oil which was used directly without further purification; ^{1}H NMR (500 MHz, $CDCl_3$): δ 7.36–7.26 (5H, m, ArH), 4.50 (2H, s, C**5**-H), 2.53 (1H, m, NH), 2.39 (3H, s, C**11**-H), 1.69–1.50 (4H, m, CH_2), 1.39–1.32 (2H, m, CH_2), 1.04 (3H, d, *J* = 6.3 Hz, C**10**-H);

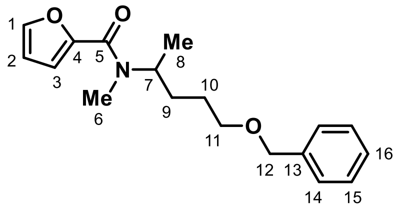

1s

General Procedure A: 2-Furoic acid (0.67 g, 6.00 mmol), 5-(benzyloxy)-N-methylpentan-2-amine (assuming 7.80 mmol), oxalyl chloride (0.66 mL, 7.80 mmol) and Et_3N (1.25 mL, 9.00 mmol) were employed. Purification by FCC (0–40% EtOAc/Hex) afforded the title compound (461 mg, 26%, 0.55:0.45 mixture of rotamers *A*:*B*) as a colorless oil; ν_{max}/cm^{-1}: 2860 (br), 1617 (s), 1404 (m); ^{1}H NMR (500 MHz, $CDCl_3$): δ 7.46 (1H, br, C**1**-H, *A*+*B*),

7.35–7.27 (5H, m, ArH), 6.93 (1H, br, C**3**-H, *A*+*B*), 6.46 (1H, br, C**2**-H, *A*+*B*), 4.79 (0.55H, br, C**7**-H, *A*), 4.48 (2H, br, C**12**-H), 4.38 (0.45H, br, C**7**-H, *B*), 3.45 (2H, br, C**11**-H, *A*+*B*), 2.95 (3H, br, C**6**-H, *A*+*B*), 1.66–1.57 (4H, m, C**9**-H and C**10**-H, *A*+*B*), 1.22 (3H, br, C**8**-H, *A*+*B*); ^{13}C NMR (126 MHz, $CDCl_3$): δ 161.1 (C**5**, *A*), 160.4 (C**5**, *B*), 148.5 (C**4**, *A*+*B*), 143.7 (C**1**, *A*), 143.3 (C**1**, *B*), 138.5 (C**13**, *A*+*B*), 128.4 (ArCH), 127.6 (ArCH), 127.6 (ArCH), 116.0 (C**3**, *A*), 115.2 (C**3**, *B*), 111.1 (C**2**, *A*+*B*), 72.9 (C**12**, *A*+*B*), 69.9 (C**11**, *A*+*B*), 52.9 (C**7**, *B*), 49.1 (C**7**, *A*), 31.1 (C**9** or C**10**, *B*), 30.1 (C**9** or C**10**, *A*), 29.7 (C**6**, *A*+*B*), 26.7 (C**9** or C**10**, *A*+*B*), 19.2 (C**8**, *B*), 17.8 (C**8**, *A*); HRMS: (ESI$^+$) calculated for $C_{18}H_{24}NO_3$ 302.1756. Found $[M+H]^+$ 302.1747.

***N*-Methyl-*N*-(3-methylbutan-2-yl)furan-2-carboxamide (1t)**

Synthesis of N-3-dimethylbutan-2-aminium chloride

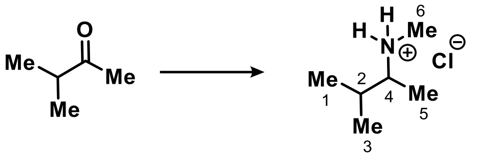

Titanium (IV) isopropoxide (15.8 mL, 52.8 mmol) was added to 40% methylamine in MeOH (12.0 mL, 121 mmol) and then 3-methylbutanone (4.31 mL, 40.0 mmol) was added dropwise at 0 °C under an atmosphere of N_2. The resulting solution was stirred at r.t. for 16 h, before $NaBH_4$ (1.51 g, 40.0 mmol) was added and the mixture stirred for a further 24 h. H_2O (20.0 mL) was added and the precipitate removed by filtration, then washed with Et_2O (20.0 mL). The resulting layers of the filtrate were separated and the aqueous layer extracted with Et_2O (3 × 20.0 mL). The combined organic layers were cooled to 0 ºC and 2 M aq. HCl (in Et_2O) (60.0 mL, 120 mmol) was added dropwise. The mixture was stirred at r.t. for 1 h and then concentrated *in vacuo* to yield the crude HCl salt, *N*-3-dimethylbutan-2-aminium chloride, as an orange solid, which was used directly without further purification; ^{1}H NMR (500 MHz, D_2O): δ 3.04 (1H, m, C**4**-H), 2.61 (3H, s, C**6**-H), 1.95 (1H, qqd, *J* = 6.8, 6.8, 4.8 Hz, C**2**-H), 1.14 (3H, d, *J* = 6.8 Hz, C**5**-H), 0.90 (3H, d, *J* = 6.8 Hz, C**1**-H or C**3**-H), 0.86 (3H, d, *J* = 6.8 Hz, C**1**-H or C**3**-H).

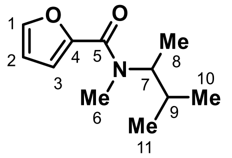

1t

General Procedure A: 2-Furoic acid (87.4 mg, 0.78 mmol), *N*-3-dimethylbutan-2-aminium chloride (assuming 1.01 mmol), oxalyl chloride (0.09 mL, 1.01 mmol) and Et_3N (0.43 mL, 3.12 mmol) were employed. Purification by FCC (20–40% EtOAc/Hex) afforded the title compound (105 mg, 69%, 0.6:0.4 mixture of rotamers *A*:*B*) as a colorless oil; ν_{max}/cm^{-1}: 29723 (m), 1634 (s), 1487 (m), 1364 (s); ^{1}H NMR (500 MHz, $CDCl_3$): δ 7.48 (1H, br, C**1**-H, *A*+*B*), 6.94 (1H, br, C**3**-H, *A*+*B*), 6.47 (1H, br, C**2**-H, *A*+*B*), 4.40 (0.6H, C**7**-H, *A*), 3.92 (0.4H, C**7**-H, *B*), 3.06 (1.8H, br, C**6**-H, *A*), 2.88 (1.2H, br, C**6**-H, *B*), 1.77 (1H, m, C**9**-H, *A*+*B*), 1.28–1.18 (3H, m, C**8**-H, *A*+*B*), 0.98–0.83 (6H, m, C**10**-H, C**11**-H, *A*+*B*); ^{13}C NMR (126 MHz, $CDCl_3$): δ 161.3 (C**5**, *B*), 160.5 (C**4**, *A*), 148.6 (C**4**, *A*+*B*), 143.6 (C**1**, *A*), 143.2 (C**1**, *B*), 115.9 (C**3**, *A*), 115.1 (C**3**, *B*), 111.1 (C**2**, *A*+*B*), 59.6 (C**7**, *B*), 55.6 (C**7**, *A*), 31.8 (C**9**, *B*), 31.1 (C**9**, *A*), 30.3 (C**6**, *A*), 27.4 (C**6**, *B*), 20.0 (C**10**, *A*+*B*), 20.0 (C**11**, *A*+*B*), 17.3 (C**8**, *B*), 15.9 (C**8**, *A*); HRMS: (ESI$^+$) calculated for $C_{11}H_{18}NO_2$ 196.1338. Found $[M+H]^+$ 196.1342.

***N*-Benzyl-*N*-isopropylfuran-2-carboxamide (1u)**

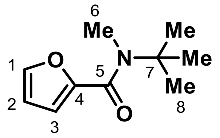

1u

General Procedure A: 2-Furoic acid (1.17 g, 10.5 mmol), *N-tert*-butylmethylamine (1.63 mL, 13.6 mmol), oxalyl chloride (1.15 mL, 13.6 mmol) and Et_3N (2.19 mL, 15.7 mmol) were employed. Purification by FCC (20% EtOAc/Hex) afforded the title compound (1.05 g, 55%) as a colorless oil; ν_{max}/cm^{-1}: 2972 (w), 1638 (s), 1365 (m), 1063 (m); ^1H NMR (500 MHz, $CDCl_3$): δ 7.39 (1H, br, C**1**-H), 6.84 (1H, d, *J* = 3.4 Hz, C**3**-H), 6.39 (1H, dd, *J* = 3.4, 1.8 Hz, C**2**-H), 3.01 (3H, s, C**6**-H), 1.43 (9H, s, C**8**-H); ^{13}C NMR (126 MHz, $CDCl_3$): δ 162.3 (C**5**), 149.8 (C**4**), 143.1 (C**1**), 114.9 (C**3**), 111.0 (C**2**), 56.9 (C**7**), 33.9 (C**6**), 27.5 (C**8**); HRMS: (ESI^+) calculated for $C_{10}H_{16}NO_2$ 182.1181. Found $[M+H]^+$ 182.1181.

Intramolecular N→C Alkyl Transfer Involving Other Alkyl Units: Products (Table 1B)

(*S*)-3-(*sec*-Butyl)-*N*-methylfuran-2-carboxamide (4p)

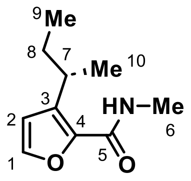

4p

General Procedure B: Compound **1p** (18.1 mg, 0.10 mmol), $[Ir(cod)_2]BARF$ (12.8 mg, 0.005 mmol), **L-4** (10.6 mg, 0.005 mmol) and 1,2-DCB (0.10 mL) were employed at 120 °C for 72 h. Purification by FCC (5–10% EtOAc/PhMe) afforded the title compound (14.7 mg, 81%) as a colorless oil; $[\alpha]_D^{21}$ -76.2 (c = 0.10, $CHCl_3$); ν_{max}/cm^{-1}: 2994 (w), 1668 (m), 1555 (m); ^1H NMR (500 MHz, $CDCl_3$): δ 7.29 (1H, d, *J* = 1.9 Hz, C**1**-H), 6.42 (1H, d, *J* = 1.9 Hz, C**2**-H), 6.32 (1H, br, NH), 3.64 (1H, m, C**7**-H), 2.95 (3H, d, *J* = 5.0 Hz, C**6**-H), 1.57–1.50 (2H, m, C**8**-H), 1.17 (3H, d, *J* = 6.9 Hz, C**10**-H), 0.88 (3H, t, *J* = 7.4 Hz, C**9**-H); ^{13}C NMR (126 MHz, $CDCl_3$): δ 160.4 (C**5**), 142.5 (C**1**), 141.0 (C**4**), 137.2 (C**3**), 111.2 (C**2**), 30.8 (C**7**), 25.5 (C**6**), 30.2 (C**8**), 20.9 (C**10**), 11.9 (C**10**); HRMS: (ESI^+) calculated for $C_{10}H_{16}NO_2$ 182.1181. Found $[M+H]^+$ 182.1178. *The e.r. of this compound was determined to be 88:12 by SFC analysis against a racemic standard (prepared using* ***rac. L-5****), as detailed below.*

SFC conditions: CHIRALART CELLULOSE-SB (25.0 cm), 99:1 CO_2:MeOH, 2.0 mL/min, 254 nm; Retention times: t_{minor} = 12.87 min, t_{major} = 12.25 min.

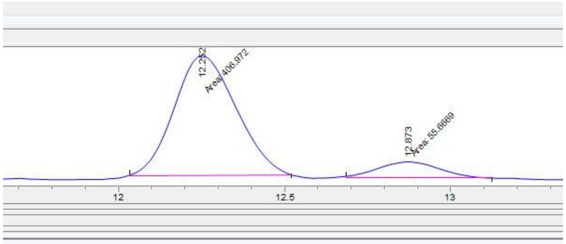

#	Time	Area	Height	Width	Area%	Symmetry
1	12.252	407	31.1	0.2178	87.968	0.812
2	12.873	55.7	4.3	0.2179	12.032	0.907

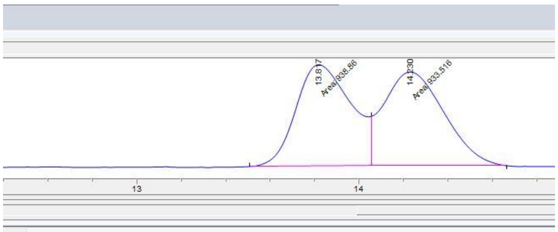

#	Time	Area	Height	Width	Area%	Symmetry
1	13.817	938.9	53.9	0.2905	50.143	0
2	14.23	933.5	49.8	0.3123	49.857	0.764

***4p** was generated in similar e.r. regardless of whether **1p** was enantioenriched (>98:2 e.r.) or racemic.*

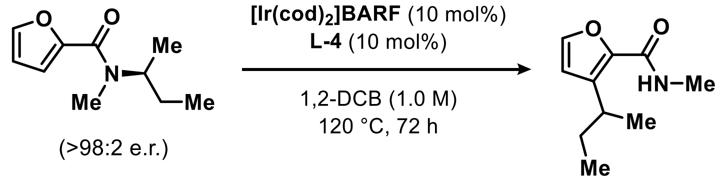

4p

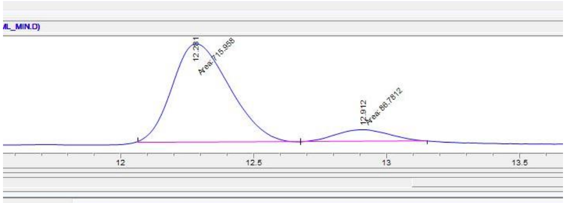

#	Time	Area	Height	Width	Area%	Symmetry
1	12.281	716	47.2	0.2529	89.189	0.69
2	12.912	86.8	5.9	0.2467	10.811	1.018

(*S*)-*N*-Methyl-3-(4-phenylbutan-2-yl)furan-2-carboxamide (4q)

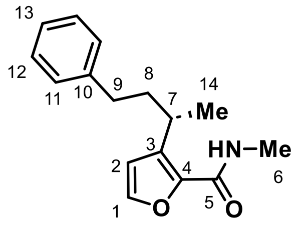

4q

General Procedure B: Compound **1q** (25.7 mg, 0.10 mmol), [Ir(cod)$_2$]BARF (12.8 mg, 0.01 mmol), **L-4** (10.6 mg, 0.01 mmol) and 1,2-DCB (0.10 mL) were employed at 120 °C for 72 h. Purification by FCC (0–40% EtOAc/Hex) afforded the title compound (24.2 mg, 94%) as a colorless oil; $[\alpha]_D^{21}$ +34.6 (c = 0.02, $CHCl_3$); ν_{max}/cm^{-1}: 2969 (m), 1762 (w), 1639 (s); ^{1}H NMR (500 MHz, $CDCl_3$): δ 7.32 (1H, d, *J* = 1.8 Hz, C**1**-H), 7.26–7.22 (2H, m, ArH), 7.16–7.13 (3H, m, ArH), 6.44 (1H, d, *J* = 1.8 Hz, C**2**-H), 6.33 (1H, br, NH), 3.82 (1H, m, C**7**-H), 2.96 (3H, d, *J* = 4.9 Hz, C**6**-H), 2.64 (1H, ddd, *J* = 13.7 Hz, 10.9, 6.2 Hz, C**8**-H), 2.48 (1H, ddd, *J* = 13.7, 10.7, 6.0 Hz, C**8**-H), 1.92–1.73 (2H, m, C**9**-H), 1.23 (3H, d, *J* = 7.0 Hz, C**14**-H); ^{13}C NMR (126 MHz, $CDCl_3$): δ 160.3 (C**5**), 142.8 (C**10**), 142.7 (C**1**), 141.7 (C**4**), 136.9 (C**3**), 128.3 (ArCH), 128.2 (ArCH), 125.6 (ArCH), 111.2 (C**2**), 39.6 (C**9**), 34.1 (C**8**), 29.6 (C**7**), 25.6 (C**6**), 21.5 (C**14**); HRMS: (ESI$^+$) calculated for $C_{16}H_{20}NO_2$ 258.1494. Found $[M+H]^+$ 258.1493. *The e.r. of this compound was determined to be 81:19 by SFC analysis against a racemic standard (prepared using **rac. L-5**), as detailed below.*

SFC conditions: CHIRALART CELLULOSE-SC (25.0 cm), 96:4 CO_2:MeOH, 2.0 mL/min, 254 nm; Retention times: t_{minor} = 11.81 min, t_{major} = 13.31 min.

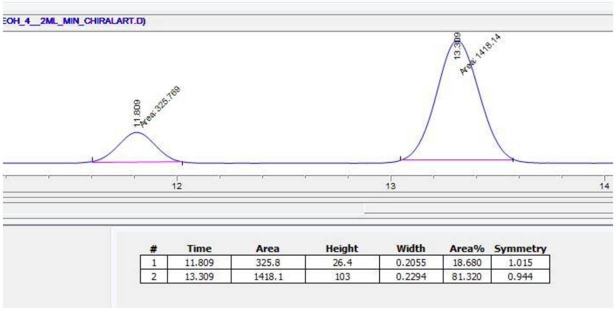

#	Time	Area	Height	Width	Area%	Symmetry
1	11.809	325.8	26.4	0.2055	18.680	1.015
2	13.309	1418.1	103	0.2294	81.320	0.944

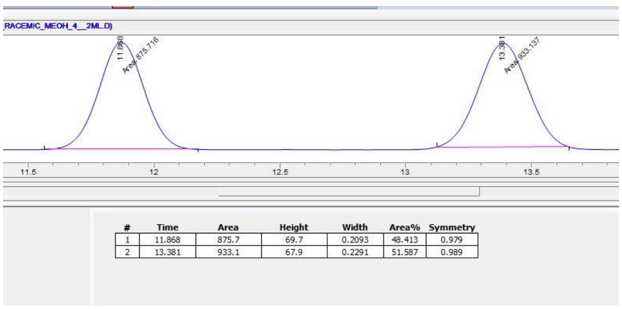

#	Time	Area	Height	Width	Area%	Symmetry
1	11.868	875.7	69.7	0.2093	48.413	0.979
2	13.381	933.1	67.9	0.2291	51.587	0.989

(*S*)-*N*-Methyl-3-(octan-2-yl)furan-2-carboxamide (4r)

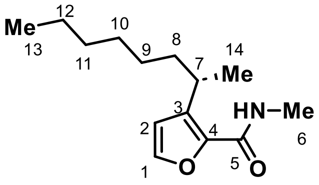

4r

General Procedure B: Compound **1r** (23.7 mg, 0.10 mmol), [Ir(cod)$_2$]BARF (12.8 mg, 0.01 mmol), **L-4** (10.6 mg, 0.01 mmol) and 1,2-DCB (0.10 mL) were employed at 120 °C for 72 h. Purification by FCC (0–40% EtOAc/Hex) afforded the title compound (21.6 mg, 91%) as a colorless oil; $[\alpha]_D^{22}$ +48.5 (c = 0.02, $CHCl_3$); ν_{max}/cm^{-1}: 3379 (br), 2919 (m), 2252 (m), 1770 (s); ^1H NMR (500 MHz, $CDCl_3$): δ 7.28 (1H, d, *J* = 1.8 Hz, C**1**-H), 6.40 (1H, d, *J* = 1.8 Hz, C**2**-H), 6.31 (1H, br, NH), 3.69 (1H, *J* = 7.0 Hz, sext, C**7**-H), 2.95 (3H, d, *J* = 4.9 Hz, C**6**-H), 1.52–1.46 (2H, m, C**8**-H), 1.29–1.19 (8H, m, C**9**-H, C**10**-H, C**11**-H, C**12**-H), 1.15 (3H, d, *J* = 7.0 Hz, C**10**-H), 0.85 (3H, t, *J* = 6.8 Hz, C**14**-H); ^{13}C NMR (126 MHz, $CDCl_3$): δ 160.3 (C**5**), 142.4 (C**1**), 141.5 (C**4**), 137.6 (C**3**), 111.3 (C**2**), 37.5 (C**8**), 31.8 (CH_2), 29.4 (CH_2), 29.2 (C**7**), 27.4 (CH_2), 25.5 (C**6**), 22.6 (CH_2), 21.4 (C**14**), 14.1 (C**13**); HRMS: (ESI^+) calculated for $C_{14}H_{24}NO_2$ 238.1807. Found $[M+H]^+$ 238.1806. *The e.r. of this compound was determined to be 89:11 by SFC analysis against a racemic standard (prepared using* ***rac. L-4****), as detailed below.*

SFC conditions: CHIRALART CELLULOSE-SC (25.0 cm), 95:10 CO_2:MeOH, 2.0 mL/min, 254 nm; Retention times: t_{minor} = 3.22 min, t_{major} = 3.54 min.

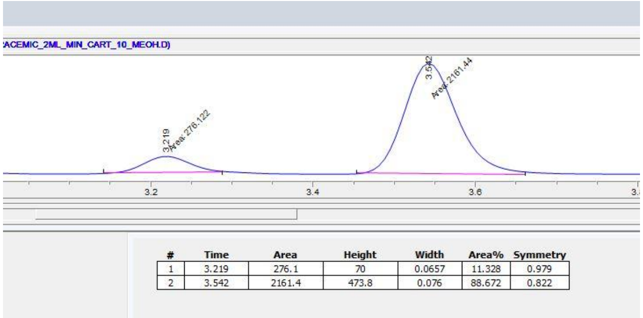

#	Time	Area	Height	Width	Area%	Symmetry
1	3.219	276.1	70	0.0657	11.328	0.979
2	3.542	2161.4	473.8	0.076	88.672	0.822

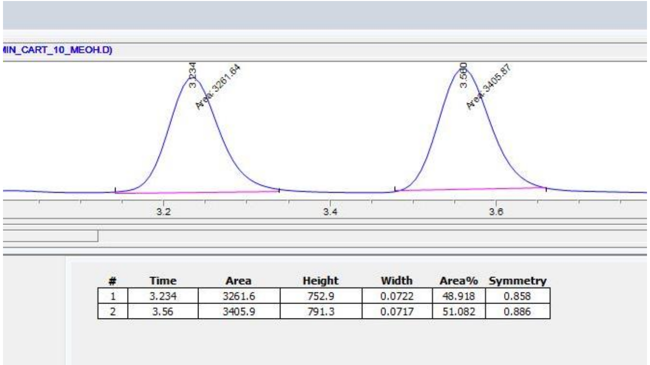

#	Time	Area	Height	Width	Area%	Symmetry
1	3.234	3261.6	752.9	0.0722	48.918	0.858
2	3.56	3405.9	791.3	0.0717	51.082	0.886

(*S*)-3-(5-Benzyloxy)pentan-2-yl)-*N*-methylfuran-2-carboxamide (4s)

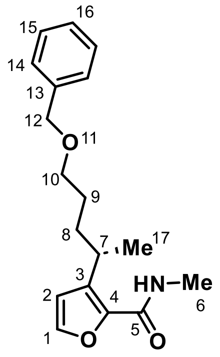

4s

General Procedure B: Compound **1s** (30.1 mg, 0.10 mmol), [Ir(cod)$_2$]BARF (12.8 mg, 0.01 mmol), **L-4** (10.6 mg, 0.01 mmol) and 1,2-DCB (0.10 mL) were employed 120 °C for 72 h. Purification by FCC (0–60% EtOAc/Hex) afforded the title compound (15.9 mg, 53%, 0.50:0.50 mixture of rotamers *A*:*B*) as a colorless oil; $[\alpha]_D^{22}$ +13.9 (c = 0.01, CHCl$_3$); ν_{max}/cm^{-1}: 2936 (br), 1717 (w), 1618 (m); ^{1}H NMR (500 MHz, CDCl$_3$): δ 7.39–7.28 (6H, m, ArH, C**1**-H, *A+B*), 6.48 (1H, br, C**2**-H, *A+B*), 4.77 (0.5H, br, C**7**-H, *A*), 4.52 (2H, s, C**12**-H, *A+B*), 3.83 (0.5H, br, C**7**-H, *B*), 3.48 (2H, br, C**10**-H, *A+B*), 2.80 (1.5H, br, C**6**-H, *A*), 2.75 (1.5H, br, C**6**-H, *B*), 1.61–1.52 (4H, m, C**8**-H, C**9**-H, *A+B*), 1.18 (3H, d, *J* = 6.7 Hz, C**17**-H, *A*), 1.09 (3H, d, *J* = 6.7 Hz, C**17**-H, *B*); ^{13}C NMR (126 MHz, CDCl$_3$): δ 170.7 (C**5**, *A*), 170.4 (C**5**, *B*), 138.6 (C**4**, *A*), 138.4 (C**4**, *B*), 134.8 (C**13**, *A+B*), 130.5 (C**1**, *A+B*), 128.4 (ArCH, *A+B*), 128.4 (ArCH, *A+B*), 127.6 (ArCH, *A+B*), 127.6 (ArCH, *A+B*), 127.5 (ArCH, *A+B*), 123.8 (C**3**, *A+B*), 116.6 (C**2**, *A+B*), 73.0 (C**12**, *A*), 72.9 (C**12**, *B*), 70.0 (C**10**, *A*), 69.8 (C**10**, *B*), 53.3 (C**7**, *A*), 47.5 (C**7**, *B*), 31.1 (C**5**, *A*), 30.3 (C**5**, *B*), 26.8 (CH$_2$), 26.8 (CH$_2$), 22.4 (CH$_2$), 21.7 (CH$_2$), 18.9 (C**17**, *A*), 18.0 (C**17**, *B*); HRMS: (ESI$^+$) calculated for $C_{18}H_{24}NO_3$ 302.1756. Found $[M+H]^+$ 302.1760. *The e.r. of this compound was determined to be 87:13 by SFC analysis against a racemic standard (prepared using* ***rac. L-5****), as detailed below.*

SFC conditions: CHIRALART CELLULOSE-SC (25.0 cm), 95:5 CO$_2$:MeOH, 2.0 mL/min, 254 nm; Retention times: t_{minor} = 14.27 min, t_{major} = 17.29 min.

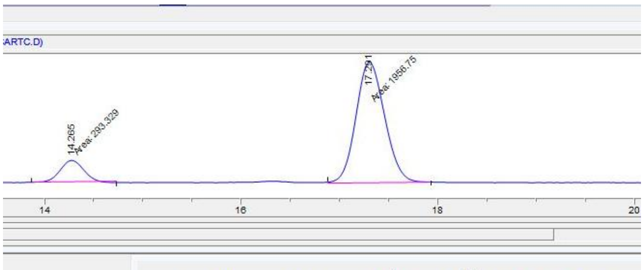

#	Time	Area	Height	Width	Area%	Symmetry
1	14.265	293.3	17.3	0.2829	13.036	0.973
2	17.291	1956.7	95.6	0.3411	86.964	0.85

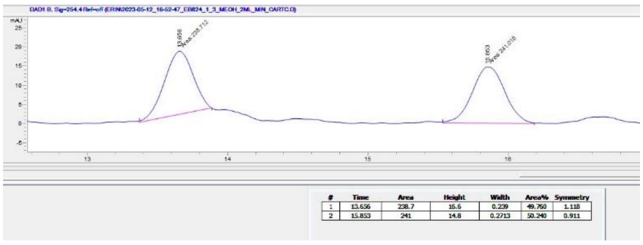

#	Time	Area	Height	Width	Area%	Symmetry
1	13.656	238.7	16.6	0.239	49.760	1.118
2	15.853	241	14.8	0.2713	50.240	0.911

(*S*)-*N*-Methyl-3-(3-methylbutan-2-yl)furan-carboxamide (4t)

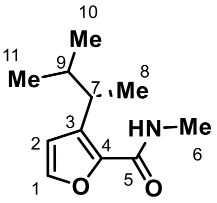

4t

General Procedure B: Compound **1t** (19.5 mg, 0.10 mmol), $[Ir(cod)_2]$BARF (12.8 mg, 0.01 mmol), **L-4** (10.6 mg, 0.01 mmol) and 1,2-DCB (0.10 mL) were employed 120 °C for 72 h. Purification by FCC (20% EtOAc/Hex) afforded the title compound (12.1 mg, 62%) as a colorless oil; $[\alpha]_D^{22}$ -23.4 (c = 0.05, $CHCl_3$); ν_{max}/cm^{-1}: 3332 (br), 2962 (m), 1645 (s), 1533 (s); ^1H NMR (500 MHz, $CDCl_3$): δ 7.29 (1H, d, *J* = 1.8 Hz, C**1**-H), 6.39 (1H, d, *J* = 1.8 Hz, C**2**-H), 6.35 (1H, br, NH), 3.53 (1H, m, C**7**-H), 2.95 (3H, d, *J* = 4.9 Hz, C**6**-H), 1.74 (1H, m, C**9**-H), 1.14 (3H, d, *J* = 7.0 Hz, C**8**-H), 0.94 (3H, d, *J* = 6.7 Hz, C**10**-H), 0.83 (3H, d, *J* = 6.7 Hz, C**11**-H); ^{13}C NMR (126 MHz, $CDCl_3$): δ 160.4 (C**5**), 142.2 (C**4**), 141.8 (C**1**), 136.9 (C**3**), 112.0 (C**2**), 35.5 (C**7**), 33.7 (C**9**), 25.5 (C**6**), 20.9 (C**10**), 19.8 (C**11**), 18.0 (C**8**); HRMS: (ESI^+) calculated for $C_{11}H_{18}NO_2$ 196.1338. Found $[M+H]^+$ 196.1332. *The e.r. of this compound was determined to be 82:18 by SFC analysis against a racemic standard (prepared using* ***rac. L-5****), as detailed below.*

SFC conditions: CHIRALART CELLULOSE-SC (25.0 cm), 97:3 CO_2:MeOH, 2.0 mL/min, 254 nm; Retention times: t_{minor} = 14.61 min, t_{major} = 14.05 min.

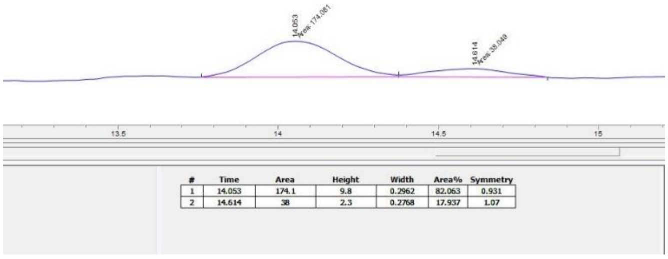

#	Time	Area	Height	Width	Area%	Symmetry
1	14.053	174.1	9.8	0.2962	82.063	0.931
2	14.614	38	2.3	0.2768	17.937	1.07

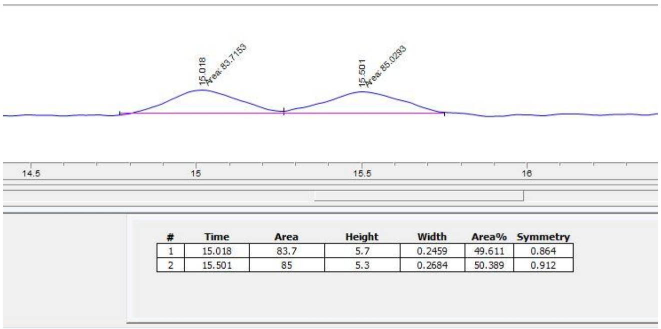

#	Time	Area	Height	Width	Area%	Symmetry
1	15.018	83.7	5.7	0.2459	49.611	0.864
2	15.501	85	5.3	0.2684	50.389	0.912

3-(*tert*-Butyl)-*N*-methylfuran-2-carboxamide (4u)

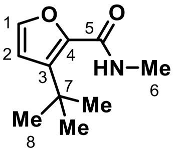

4u

General Procedure B: Compound **1u** (18.1 mg, 0.10 mmol), [Ir(cod)$_2$]BARF (6.40 mg, 0.005 mmol), **L-4** (5.30 mg, 0.005 mmol) and 1,2-DCB (0.10 mL) were employed at 120 °C for 72 h. Purification by FCC (20% EtOAc/Hex) afforded the title compound (17.2 mg, 95%) as a colorless oil; ν_{max}/cm^{-1}: 3348 (br), 2955 (w), 1650 (s), 1529 (s); ^{1}H NMR (500 MHz, CDCl$_3$): δ 7.25 (1H, d, *J* = 1.8 Hz, C**1**-H), 6.47 (1H, br, NH), 6.44 (1H, d, *J* = 1.8 Hz C**2**-H), 2.95 (3H, d, *J* = 4.9 Hz, C**6**-H), 1.41 (9H, s, C**8**-H); ^{13}C NMR (126 MHz, CDCl$_3$): δ 159.9 (C**5**), 141.9 (C**4**), 141.0 (C**1**), 140.9 (C**3**), 112.7 (C**2**), 30.9 (C**7**), 30.0 (C**8**), 25.8 (C**6**); HRMS: (ESI$^+$) calculated for $C_{10}H_{16}NO_2$ 182.1181. Found [M+H]$^+$ 182.1178.

Evaluation of L-5 (Derived from Commercially Available SPINOL)

During the course of this study, parent SPINOL became commercially available and this allowed easy access to ***L-5****. Evaluation of this ligand on a subset of substrates, with General Procedure B, but using Ir(cod)$_2$]BARF (10 mol%) and* ***L-5*** *(10 mol%), revealed that it is effective in certain cases, but does not offer the generality of* ***L-4****.*

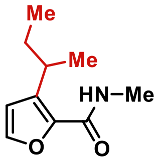

4p, 95% Yield

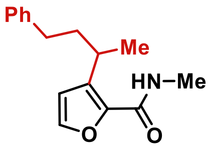

4q, 60% Yield

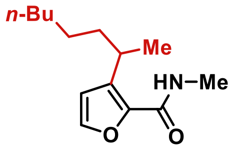

4r, 52% Yield

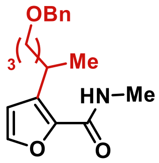

4s, 3% Yield

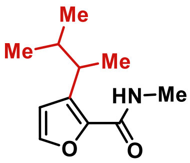

4t, 62% Yield

Unsuccessful Systems (Table 1C)

***N*,*N*-Diethylfuran-2-carboxamide (1v)**

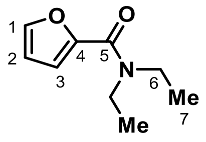

1v

General Procedure A: 2-Furoic acid (1.25 g, 11.2 mmol), diethylamine (1.50 mL, 14.5 mmol), oxalyl chloride (1.25 mL, 14.6 mmol) and Et_3N (2.33 mL, 16.7 mmol) were employed. Purification by FCC (30% EtOAc/Hex) afforded the title compound (0.98 g, 53%) as a pale yellow oil; ^1H NMR (500 MHz, $CDCl_3$): δ 7.46 (1H, m, C**1**-H), 7.00 (1H, dd, *J* = 3.4, 1.0 Hz, C**3**-H), 6.46 (1H, dd, *J* = 3.4, 1.8 Hz, C**2**-H), 3.55 (4H, br, C**6**-H), 1.25 (6H, s, C**7**-H); ^{13}C NMR (126 MHz, $CDCl_3$): δ 159.6 (C**5**), 148.7 (C**4**), 143.5 (C**1**), 115.8 (C**3**), 111.2 (C**2**), 42.5 (br, C**6**), 41.4 (br, C**6**), 14.6 (br, C**7**), 12.9 (br, C**7**).

The spectroscopic properties for this compound were consistent with the data available in the literature.[5]

***N*,*N*-Bis(1-phenylethyl)furan-2-carboxamide (1w)**

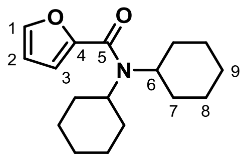

1w

To a flame-dried Young's tube was added 2-furoic acid (560 mg, 5.00 mmol) and the vessel evacuated and refilled with N_2 three times. Thionyl chloride (3.63 mL, 50.0 mmol) was added and the solution heated at 85 °C for 5 h. After cooling to r.t. the reaction mixture was concentrated *in vacuo* and the residue dissolved in CH_2Cl_2 (5.00 mL). Dicyclohexylamine (1.09 mL, 5.50 mmol) was then added dropwise at 0 °C and the resulting solution stirred at r.t. for 18 h. H_2O (10.0 mL) was added and the solution extracted with CH_2Cl_2 (3 × 10.0 mL). The organic layers were combined, washed with saturated aq. $NaHCO_3$ (10.0 mL), then H_2O (10.0 mL), dried (Na_2SO_4), filtered and concentrated *in vacuo*. Purification by FCC (10% EtOAc/Hex) afforded the title compound (0.90 g, 65%) as a colorless solid; m.p. 96–98 °C (pentane); ^1H NMR (500 MHz, $CDCl_3$): δ 7.41 (1H, d, *J* = 1.0 Hz, C**1**-H), 6.78 (1H, dd, *J* = 3.5, 1.0 Hz, C**3**-H), 6.42 (1H, dd, *J* = 3.5, 1.0 Hz, C**2**-H), 4.00–1.46 (16H, m, CH_2), 1.31–1.10 (6H, m, CH_2); ^{13}C NMR (126 MHz, $CDCl_3$): δ 160.6 (C**5**), 149.7 (C**4**), 142.9 (C**1**), 114.0 (C**3**), 111.0 (C**2**), 57.8 (CH), 31.0 (CH_2), 26.3 (CH_2), 25.4 (CH_2).

The spectroscopic properties for this compound were consistent with the data available in the literature.[7]

_N,N_-Bis(1-phenylethyl)furan-2-carboxamide (1x)

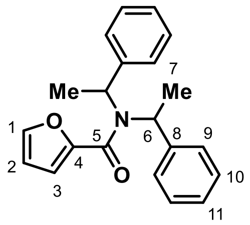

1x

General Procedure A: 2-Furoic acid (192 mg, 1.71 mmol), bis(1-phenylethyl)amine (500 mg, 2.22 mmol), oxalyl chloride (0.19 mL, 2.22 mmol) and Et$_3$N (0.36 mL, 2.57 mmol) were employed. Purification by FCC (20% EtOAc/Hex) afforded the title compound (0.32 g, 60%) as a colorless solid; m.p. 59–61 °C (pentane); ν_{max}/cm^{-1}: 2985 (w), 1620 (s), 1425 (m), 1312 (s); ^{1}H NMR (500 MHz, CDCl$_3$): δ 7.46 (1H, br, C**1**-H), 7.19–7.15 (10H, m, ArH), 6.96 (1H, dd, J = 3.5, 0.8 Hz, C**3**-H), 6.45 (1H, dd, J = 3.5, 1.8 Hz, C**2**-H), 5.20 (2H, br, C**6**-H), 1.85 (6H, d, J = 7.0 Hz, C**7**-H); ^{13}C NMR (126 MHz, CDCl$_3$): δ 160.3 (C**5**), 149.3 (C**4**), 143.1 (C**1**), 140.5 (C**8**), 128.2 (C**11**), 127.9 (C**9** or C**10**), 127.2 (C**9** or C**10**), 115.6 (C**3**), 111.3 (C**2**), 55.2 (C**6**), 18.7 (C**7**); HRMS: (ESI$^+$) calculated for $C_{21}H_{22}NO_2$ 320.1651. Found [M+H]$^+$ 320.1644.

*In addition to **1v**, **1w** and **1x**, unsuccessful systems are outlined below.*

Substrates with directing group at 1-position:

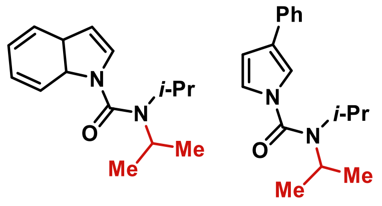

Substrates with directing group at 2-position:

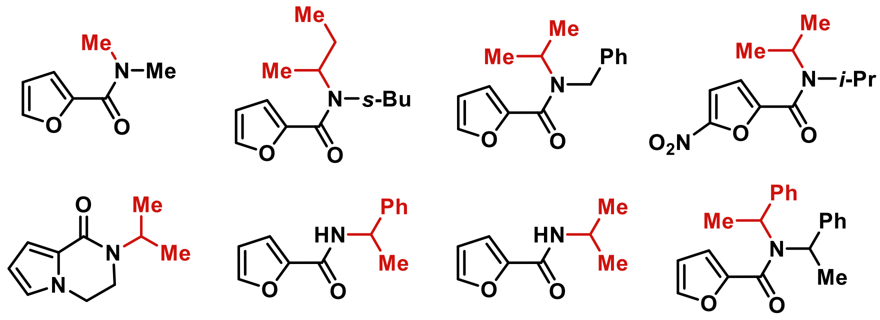

Substrates with directing group at 3-position:

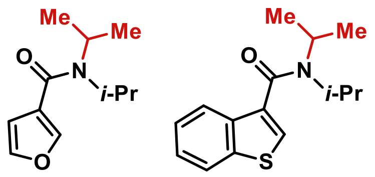

Mechanistic Studies (Scheme 3A)

N-Isopropyl-*N*-(propan-2-yl-1,1,1,3,3,3-d$_6$)furan-2-carboxamide (*deuterio*-1a)

Synthesis of N-isopropylpropan-1,1,1,3,3,3-d$_6$-2-aminium chloride

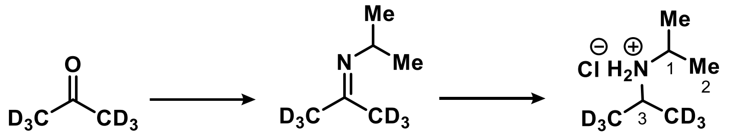

To a mixture of isopropylamine (26.4 mL, 308 mmol) and acetone-d$_6$ (25.0 mL, 308 mmol) under an atmosphere of N_2 was added CaH_2 (5.85 g, 139 mmol) and $CaCl_2$ (0.50 g, 4.51 mmol) at 0 °C. The reaction was monitored and kept at 0 °C to prevent any evaporation of starting materials due to the extreme exotherm. After the evolution of H_2 was observed to have finished, the reaction was then warmed to r.t. and stirred for 24 h. Et_2O (20.0 mL) was added to the resulting mixture and the solution used directly in the next step (kept under an atmosphere of N_2).

To a solution of $LiAlH_4$ (4.00 g, 105 mmol) in Et_2O (100 mL) under an atmosphere of N_2 was added the crude solution of the imine dropwise *via* cannula transfer. The resulting mixture was stirred at r.t. for 24 h then cooled to 0 ºC. Et_2O (50.0 mL), H_2O (4.00 mL) and 5% w/v aq. NaOH (4.00 mL) were added carefully and the solution warmed to r.t. H_2O (15.0 mL) was then added and the resulting mixture stirred at r.t. for 15 min before $MgSO_4$ was added and the mixture stirred for a subsequent 15 min. The insoluble salts were removed *via* filtration and 4 M HCl in dioxane (40.0 mL) added dropwise to the filtrate and the solution left to stir for 30 min. The solution was concentrated *in vacuo* to afford the crude HCl salt in sufficient purity for direct use in the subsequent step; ^{1}H NMR (500 MHz, DMSO): δ 8.92 (1H, br, NH), 4.11 (1H, br, NH), 3.33 (2H, m, C**1**-H, C**3**-H), 1.24 (6H, d, *J* = 6.5 Hz, C**2**-H).

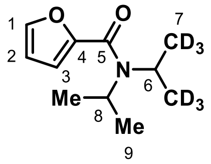

***deuterio*-1a**

General Procedure A: 2-Furoic acid (2.00 g, 17.8 mmol), *N*-isopropylpropan-1,1,1,3,3,3-d$_6$-2-aminium chloride (3.32 g, 23.1 mmol), 2M oxalyl chloride in CH_2Cl_2 (11.6 mL, 23.1 mmol) and Et_3N (9.90 mL, 71.1 mmol) were employed. Purification by FCC (10–20% EtOAc/Hex) afforded the title compound (883 mg, 25%) as a colorless oil; ν_{max}/cm^{-1}: 2971 (w), 2226 (*C-D stretch*), 1621 (s), 1477 (m), 1329 (m); ^{1}H NMR (500 MHz, CDCl$_3$): 7.45 (1H, dd, *J* = 1.8, 0.9 Hz, C**1**-H), 6.85 (1H, dd, *J* = 3.4, 0.9 Hz, C**3**-H), 6.46 (1H, dd, *J* = 3.4, 1.8 Hz, C**2**-H), 3.96 (2H, br, C**6**-H, C**8**-H), 1.38 (6H, br, C**9**-H); ^{2}H NMR (77 MHz, CDCl$_3$): δ 7.47 (br, C**7**-D), 1.36 (br, C**7**-D); ^{13}C NMR (126 MHz, CDCl$_3$): δ 160.3 (C**5**), 149.6 (C**4**), 142.8 (C**1**), 113.9 (C**3**), 110.9 (C**2**), 47.7 (br, C**6**, C**8**), 20.9 (C**9**); HRMS: (CI$^+$) calculated for $C_{11}H_{11}D_6NO_2$ 201.1636. Found [M]$^+$ 201.1651.

The chemical shifts match the data for the non-deuterated analogue (**1a**).

Deuterium Exchange Experiment

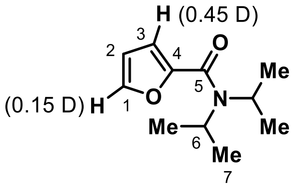

***deuterio*-1a'**

To a flame-dried resealable Schlenk tube was added diisopropylfuran **1a** (75.0 mg, 0.38 mmol), [Ir(cod)$_2$]BARF (24.4 mg, 0.019 mmol), and **L-4** (20.4 mg, 0.019 mmol). The tube was fitted with a rubber septum, evacuated and purged with N_2 three times. 1,2-DCB (0.40 mL) and D_2O (0.21 mL, 11.5 mmol) were added, the rubber septum was replaced with a Young's tap and the reaction sealed under an atmosphere of N_2. The reaction was heated at 120 °C for 3 h before being cooled to r.t. and concentrated *in vacuo*. Purification by FCC (5–20% EtOAc/Hex) afforded the recovered **1a**. Deuterium incorporation was calculated by integration of the ^{1}H NMR spectrum and that exchange occurs at the C1 and C3 positions was confirmed by ^{2}H NMR analysis; ^{1}H NMR (500 MHz): 7.43 (0.85H, d, J = 1.7 Hz, C**1**-H), 6.83 (0.55H, d, J = 3.4, 0.8 Hz, C**3**-H), 6.44−6.42 (1H, m, C**2**-H), 3.98 (2H, br, C**6**-H), 1.40 (12H, br, C**7**-H); ^{2}H NMR (77 MHz, CDCl$_3$): δ 7.47 (br, C**1**-D), 6.88 (br, C**3**-D); ^{13}C NMR (126 MHz, CDCl$_3$): δ 160.3 (C**5**), 149.6 (C**4**), 142.8 (C**1**), 113.9 (C**3**), 110.9 (C**2**), 47.9 (br, C**6**), 20.9 (C**7**).

Catalysis of Mono-Deuterated Amide

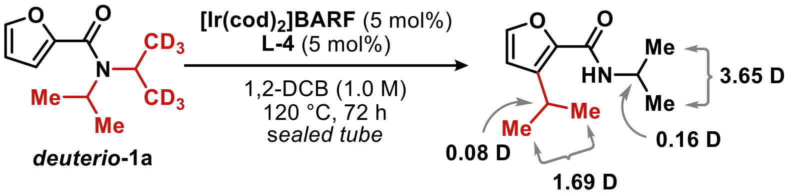

***deuterio*-4a**

Catalysis of Deuterium Exchange Product

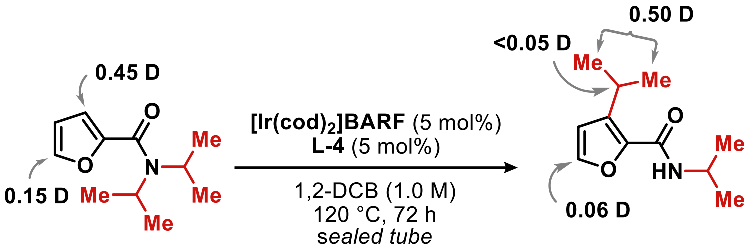

***deuterio*-1a'** ***deuterio*-4a'**

A Key Observation: Intermolecular Variant (Scheme 4A)

Methyl 5-(diisopropylcarbamoyl)furan-2-carboxylate (1y)

Synthesis of 5-(methoxycarbonyl)furan-2-carboxylic acid

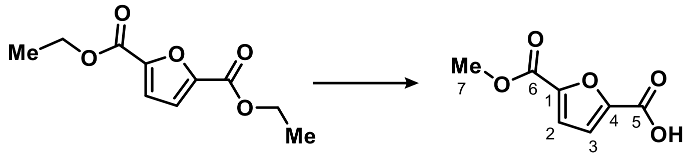

Diethylfuran-2,5-dicarboxylate (2.00 g, 9.42 mmol) was dissolved in MeOH (20.0 mL) and NaOH (0.44 g, 11.0 mmol) added. The reaction mixture was heated at 50 °C for 24 h before being acidified with 1 M aq. HCl to a pH of 1. The precipitate was collected by filtration, washed with H_2O (10.0 mL) and dried at 60 °C under high vacuum for 5 h to afford the title compound (1.36 g, 85%) as a colorless solid; m.p. decomposition at 215 °C (H_2O); ^{1}H NMR (500 MHz, MeOD): δ 7.31 (1H, d, *J* = 3.5 Hz, C**3**-H), 7.28 (1H, d, *J* = 3.5 Hz, C**2**-H), 3.93 (3H, s, C**9**-H); ^{13}C NMR (126 MHz, MeOD): δ 158.4 (C**5**), 157.6 (C**6**), 146.6 (C**4**), 145.5 (C**1**), 117.3 (C**3**), 117.1 (C**2**), 50.4 (C**7**).

The spectroscopic properties for this compound were consistent with the data available in the literature.[11]

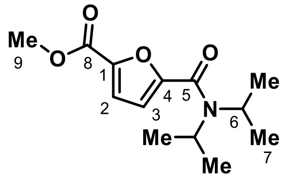

1y

General Procedure A: 5-(Methoxycarbonyl)furan-2-carboxylic acid (1.00 g, 5.43 mmol), diisopropylamine (0.99 mL, 7.06 mmol), oxalyl chloride (0.90 mL, 7.10 mmol) and Et_3N (1.13 mL, 8.15 mmol) were employed. Purification by FCC (0–50% EtOAc/Hex) afforded the title compound (73.6 mg, 6%) as a colorless solid; m.p. decomposition at 209 °C (toluene); ν_{max}/cm^{-1}: 2915 (m), 1703 (m), 1406 (m); ^{1}H NMR (500 MHz, $CDCl_3$): δ 7.17 (1H, d, *J* = 3.6 Hz, C**1**-H), 6.88 (1H, d, *J* = 3.6 Hz, C**3**-H), 3.89 (3H, br, C**10**-H), 3.17 (2H, br, C**6**-H), 1.45–1.24 (14H, br, C**7**-H, C**9**-H); ^{13}C NMR (126 MHz, $CDCl_3$): δ 159.5 (C**5**), 158.9 (C**8**), 152.3 (C**4**), 143.8 (C**1**), 118.4 (C**3**), 114.8 (C**2**), 52.3 (C**10**), 47.8 (C**6**), 20.7 (C**9**), 19.3 (C**7**); HRMS: (ESI$^+$) calculated for $C_{13}H_{20}NO_4$ 254.1392. Found $[M+H]^+$ 254.1390.

***N*,3-Diisopropylthiophene-2-carboxamide (4y)**

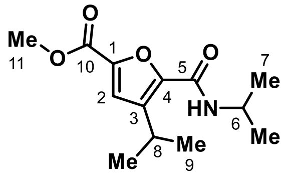

4y

General Procedure B: Compound **1y** (25.3 mg, 0.10 mmol), [Ir(cod)$_2$]BARF (6.40 mg, 0.005 mmol), **L-4** (5.30 mg, 0.005 mmol) and 1,2-DCB (0.10 mL) were employed at 120 °C for 48 h. Purification by FCC (20–40% EtOAc/Hex) afforded the title compound (2.90 mg, 11%) as a colorless oil; ν_{max}/cm^{-1}: 3665 (br), 2969 (s), 1630 (m); ^{1}H NMR (500 MHz, CDCl$_3$): δ 7.16 (1H, s, C**2**-H), 6.40 (1H, br, NH), 4.24 (1H, m, C**6**-H), 3.91 (3H, s, C**11**-H), 3.81 (1H, hept, *J* = 6.9 Hz, C**8**-H), 1.26 (6H, d, *J* = 6.9 Hz, C**7**-H), 1.21 (6H, d, *J* = 6.9 Hz, C**9**-H),^{13}C NMR (126 MHz, CDCl$_3$): δ 158.9 (C**10**), 157.9 (C**5**), 143.5 (C**1**), 142.9 (C**4**) 139.5 (C**3**), 118.4 (C**2**), 52.2 (C**11**), 41.2 (C**6**), 29.7 (C**8**), 23.0 (C**7**), 22.8 (C**9**); HRMS: (ESI$^+$) calculated for $C_{13}H_{20}NO_4$ 254.1392. Found [M+H]$^+$ 254.1392.

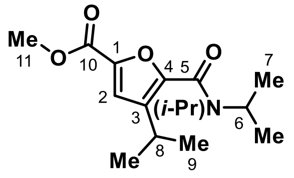

***N-i*-Pr-4y**

In addition to **4y**, ***N-i*-Pr-4y** was afforded (15.5 mg, 19%) as a colorless oil; ν_{max}/cm^{-1}: 2930 (m), 1731 (m), 1633 (s); ^{1}H NMR (500 MHz, CDCl$_3$): δ 7.13 (1H, s, C**2**-H), 3.87 (3H, s, C**9**-H), 3.69 (2H, br, C**6**-H), 3.15 (1H, hept, *J* = 6.9 Hz, C**8**-H), 1.50 (6H, br, C**7**-H), 1.20 (6H, d, *J* = 6.9 Hz, C**9**-H); ^{13}C NMR (126 MHz, CDCl$_3$): δ 160.7 (C**8**), 159.0 (C**5**), 146.8 (C**1**), 136.2 (C**4**), 135.2 (C**3**) 117.8 (C**2**), 52.0 (C**11**), 41.2 (C**6**), 24.4 (C**8**), 22.8 (C**9**), 20.6 (C**7**); HRMS: (ESI$^+$) calculated for $C_{16}H_{26}NO_4$ 296.1862. Found [M+H]$^+$ 296.1862.

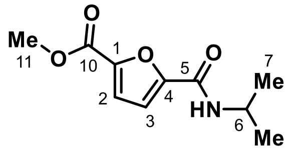

NH-1y

In addition to **4y**, **NH-1y** was afforded (4.90 mg, 23%) as a colorless oil; ν_{max}/cm^{-1}: 2918 (m), 1730 (m), 1663 (s); ^{1}H NMR (500 MHz, CDCl$_3$): δ 7.19 (1H, d, *J* = 3.7 Hz, C**3**-H), 7.15 (1H, d, *J* = 3.5 Hz, C**2**-H), 6.44 (1H, br, NH), 4.27 (1H, dhept, *J* = 8.2, 6.6 Hz, C**6**-H), 1.27 (1H, d, *J* = 6.6 Hz, C**7**-H); ^{13}C NMR (126 MHz, CDCl$_3$): δ 158.8 (C**8**), 156.7 (C**5**), 150.7 (C**1**), 144.5 (C**4**) 119.2 (C**3**), 115.0 (C**6**), 52.3 (C**9**) 41.6 (C**6**), 22.7 (C**7**); HRMS: (ESI$^+$) calculated for $C_{10}H_{14}NO_4$ 212.0923. Found [M+H]$^+$ 212.0920.

Identification of an Effective Alkyl Transfer Reagent (Scheme 4B)

N,*N*-Diethyl-3-isopropylfuran-2-carboxamide (4v)

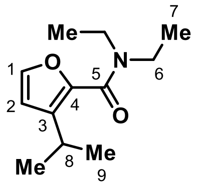

4v

General Procedure C: Compound **1v** (16.7 mg, 0.10 mmol), **6a** (28.6 mg, 0.20 mmol), [Ir(cod)$_2$]BARF (6.40 mg, 0.005 mmol), **L-4** (5.30 mg, 0.005 mmol) and 1,2-DCB (0.10 mL) were employed at 120 °C for 72 h. Purification by FCC (5–20% EtOAc/Hex) afforded the title compound (2.6 mg, 19%) as a colorless oil; ν_{max}/cm^{-1}: 3260 (m), 2970 (m), 1619 (s); ^{1}H NMR (500 MHz, CDCl$_3$): δ 7.30 (1H, d, *J* = 1.8 Hz, C**1**-H), 6.40 (1H, d, *J* = 1.8 Hz, C**2**-H), 3.45 (4H, br, C**6**-H), 3.43 (1H, m, C**8**-H), 1.23 (6H, t, *J* = 7.1 Hz, C**7**-H), 1.19 (6H, d, *J* = 6.9 Hz, C**9**-H); ^{13}C NMR (126 MHz, CDCl$_3$): δ 158.5 (C**5**), 141.3 (C**4**), 139.9 (C**1**), 137.6 (C**3**) 110.1 (C**2**), 32.6 (C**6**), 23.2 (C**8**), 22.1 (C**9**), 14.0 (C**7**); HRMS: (ESI$^+$) calculated for $C_{12}H_{20}NO_2$ 210.1494. Found [M+H]$^+$ 210.1490.

N,*N*-Diisopropylacetamide (6a)

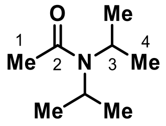

6a

Acetyl chloride (2.60 mL, 36.9 mmol) was added slowly to a solution of diisopropylamine (10.7 mL, 76.1 mmol) in CH_2Cl_2 (20.0 mL) at 0 °C and the mixture stirred at r.t. for 16 h. H_2O (20.0 mL) was added and the resulting solution extracted with Et_2O (3 × 20 mL). The combined organic layers were washed with brine (20.0 mL), dried ($MgSO_4$), filtered and concentrated *in vacuo* to afford the title compound (2.98 g, 57%) as a yellow oil; ^{1}H NMR (500 MHz, CDCl$_3$): δ 3.90 (1H, hept, *J* = 6.8 Hz, C**3**-H), 3.54 (1H, br, C**3**-H), 2.08 (3H, s, C**1**-H), 1.37 (6H, d, *J* = 6.8 Hz, C**4**-H), 1.21 (6H, d, *J* = 6.8 Hz, C**4**-H); ^{13}C NMR (126 MHz, CDCl$_3$): δ 169.5 (C**2**), 49.3 (C**3**), 45.5 (C**3**), 24.0 (C**1**), 21.0 (C**4**), 20.7 (C**4**).

The spectroscopic properties for this compound were consistent with the data available in the literature.[12]

*Dealkylation of a simple amide (**6a**)*

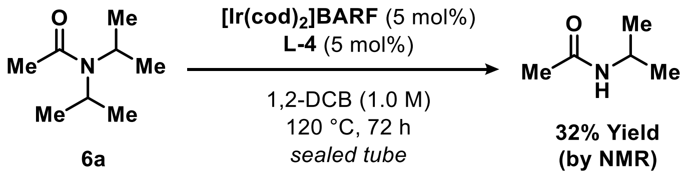

***N*-Isopropylacetamide (S6)**

A pure sample of ***S6*** *was prepared by a known method as a reference for the above experiment.*

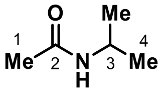

S6

To a solution of acetic anhydride (1.20 mL, 12.7 mmol) in (10.0 mL) at 0 °C was added slowly Et_3N (11.0 mL, 79.0 mmol) and isopropylamine (5.44 mL, 63.5 mmol) and the reaction stirred at r.t. for 16 h. The reaction mixture was concentrated *in vacuo* and then Et_2O (20.0 mL) added along with K_2CO_3 (3.00 g, 21.7 mmol). The resulting mixture was stirred at r.t. for 16 h and then filtered. The filtrate was concentrated *in vacuo* to afford the title compound (3.80 g, 71%) as a colorless oil; ^{1}H NMR (500 MHz, $CDCl_3$): δ 5.46 (1H, br, NH), 4.04 (1H, dhept, *J* = 8.1, 6.6 Hz, C**3**-H), 1.93 (3H, s, C**1**-H), 1.13 (6H, d, *J* = 6.6 Hz, C**4**-H); ^{13}C NMR (126 MHz, $CDCl_3$): δ 169.2 (C**2**), 41.4 (C**3**), 23.5 (C**1**), 22.8 (C**4**).

The spectroscopic properties for this compound were consistent with the data available in the literature.[13]

***N*-Isopropyl-*N*-methylacetamide (6b)**

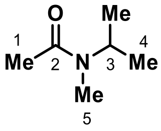

6b

Acetyl chloride (0.51 mL, 7.20 mmol) was added slowly to a solution of *N*-methyl-2-propanamine (1.50 mL, 14.4 mmol) in CH_2Cl_2 (20.0 mL) at 0 °C and the mixture stirred at r.t. for 16 h. H_2O (20.0 mL) was added and the resulting solution extracted with Et_2O (3 × 20.0 mL). The combined organic layers were washed with brine (20.0 mL), dried ($MgSO_4$), filtered and concentrated *in vacuo* to afford the title compound (99.1 mg, 12%, 0.52:0.48 mixture of rotamers *A*:*B*) as a colorless oil; ν_{max}/cm^{-1}: 2924 (w), 1630 (s), 1406 (m); ^{1}H NMR (500 MHz, $CDCl_3$): δ 4.86 (0.52H, hept, *J* = 6.8 Hz, C**1**-H, *A*), 4.01 (0.48H, hept, *J* = 6.6 Hz, C**1**-H, *B*), 2.80 (1.56H, s, C**1**-H, *A*), 2.76 (1.44H, s, C**1**-H, *B*), 2.11 (1.44H, s, C**5**-H, *B*), 2.06 (1.56H, s, C**5**-H, *A*), 1.18 (2.73H, d, *J* = 6.6 Hz, C**4**-H), 1.07 (2.96H, d, *J* = 6.8 Hz, C**4**-H); ^{13}C NMR (126 MHz, $CDCl_3$): δ 170.2 (C**2**, *A*), 170.0 (C**2**, *B*), 48.8 (C**3**, *B*), 43.6 (C**3**, *A*), 28.9 (C**1**, *A*), 25.6 (C**1**, *B*), 22.5 (C**5**, *A*), 21.6 (C**5**, *B*), 20.4 (C**4**, *B*), 19.5 (C**4**, *A*); HRMS: (ESI^+) calculated for $C_6H_{14}NO$ 116.1075. Found $[M+H]^+$ 116.1069.

1,1-Diethyl-3,3-diisopropylurea (6c)

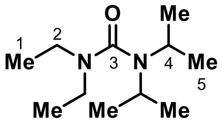

6c

Diethylamine (5.08 mL, 48.8 mmol) was added slowly to a solution of *N*,*N*-diisopropylcarbamoyl chloride (4.00 g, 24.4 mmol) in toluene (20.0 mL) at 0 °C. The mixture was stirred at r.t. for 16 h before being concentrated *in vacuo* to remove excess diethylamine. To the resulting residue was added EtOAc (20.0 mL) and H_2O (20.0 mL) and the layers separated. The organic layer was washed with 1 M aq. HCl (20.0 mL), H_2O (20.0 mL), saturated aq. $NaHCO_3$ (20.0 mL) and brine (20.0 mL) then dried (Na_2SO_4) and concentrated *in vacuo*. Purification by distillation (80 °C at 0.80 mbar) afforded the title compound (3.24 g, 66%) as a colorless oil; ν_{max}/cm^{-1}: 2967 (m), 1643 (s), 1423 (m), 1285 (s); ^{1}H NMR (500 MHz, $CDCl_3$): δ 3.63 (2H, hept, *J* = 6.7 Hz, C**4**-H), 3.05 (4H, q, *J* = 7.1 Hz, C**2**-H), 1.25 (6H, d, *J* = 6.7 Hz, C**5**-H), 1.07 (6H, t, *J* = 7.1 Hz, C**1**-H); ^{13}C NMR (126 MHz, $CDCl_3$): δ 164.4 (C**3**), 47.4 (C**4**), 42.7 (C**2**). 21.6 (C**5**), 13.2 (C**1**); HRMS: (ESI$^+$) calculated for $C_{11}H_{25}N_2O$ 201.1967. Found [M+H]$^+$ 201.1965.

2,6-Difluoro-*N*,*N*-diisopropylbenzamide (6d)

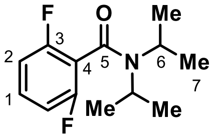

6d

To a solution of diisopropylamine (1.84 mL, 13.0 mmol) and Et_3N (2.09 mL, 15.0 mmol) in CH_2Cl_2 (30.0 mL) was added 2,6-difluorobenzoyl chloride (1.26 mL, 10.0 mmol) at 0 °C and the mixture stirred at r.t. for 16 h. H_2O (30.0 mL) was added and the resulting solution extracted with CH_2Cl_2 (3 × 30 mL). The combined organic layers were washed with saturated aq. $NaHCO_3$ (30.0 mL), H_2O (30.0 mL), dried ($NaSO_4$) and concentrated *in vacuo*. Purification by FCC (20% EtOAc/Hex) afforded the title compound (1.95 g, 75%) as a colorless solid; m.p. 110–112 °C (EtOH); ν_{max}/cm^{-1}: 2964 (br), 1736 (m), 1579 (m); ^{1}H NMR (500 MHz, $CDCl_3$): 7.28 (1H, m, C**1**-H), 6.96-6.87 (2H, m, C**3**-H), 3.73 (1H, hept, *J* = 6.6 Hz, C**6**-H), 3.55 (1H, hept, *J* = 6.7 Hz, C**6**-H), 1.56 (6H, d, *J* = 6.7 Hz, C**7**-H), 1.16 (6H, d, *J* = 6.6 Hz, C**7**-H); ^{19}F NMR (470 MHz, $CDCl_3$): -114.5; ^{13}C NMR (126 MHz, $CDCl_3$): δ 160.5 (C**5**), 158.7 (dd, $^1J_{C\text{-}F}$ = 246.3 Hz, $^3J_{C\text{-}F}$ = 8.8 Hz, C**3**), 129.9 (t, $^3J_{C\text{-}F}$ = 9.6 Hz, C**1**), 116.2 (t, $^2J_{C\text{-}F}$ = 24.5 Hz, C**4**), 111.65 (dd, $^2J_{C\text{-}F}$ = 20.1 Hz, $^4J_{C\text{-}F}$ = 5.0 Hz, C**2**), 51.5 (C**6**), 46.4 (C**6**), 20.8 (C**7**), 20.4 (C**7**); HRMS: (ESI$^+$) calculated for $C_{13}H_{18}F_2NO$ 242.1357. Found [M+H]$^+$ 242.1347.

N,*N*-Diisopropylcyclohexanecarboxamide (6e)

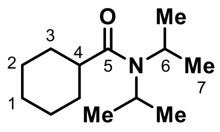

6e

To a solution of cyclohexanecarbonyl chloride (1.00 mL, 7.08 mmol) in CH_2Cl_2 (25.0 mL) was added diisopropylamine (1.25 mL, 9.20 mmol) and Et_3N (1.48 mL, 10.6 mmol) at 0 °C and the reaction mixture stirred at r.t. for 16 h. To the resulting residue was added H_2O (25.0 mL) and the layers separated. The aqueous layer was extracted with CH_2Cl_2 (3 × 25.0 mL) and the organic layers combined then washed with saturated aq. $NaHCO_3$ (25.0 mL) and brine (25.0 mL), dried (Na_2SO_4) and concentrated *in vacuo*. Purification by FCC (0–30% EtOAc/Hex) afforded the title compound (772 mg, 52%) as a colorless solid; m.p. 79–80 °C (toluene); ^{1}H NMR (500 MHz, $CDCl_3$): 3.97 (1H, br, C**6**-H), 3.56 (1H, br, C**6**-H), 2.36 (1H, tt, *J* = 11.6, 3.4 Hz, C**4**-H), 1.81–1.77 (2H, m, CH_2), 1.70–1.62 (4H, m, CH_2), 1.57–1.49 (2H, m, CH_2), 1.33 (6H, d, *J* = 6.7 Hz, C**7**-H), 1.28–1.21 (8H, m, CH_2, C**7**-H); ^{13}C NMR (126 MHz, $CDCl_3$): δ 175.3 (C**5**), 45.4 (C**6**-H), 42.5 (C**6**-H), 29.7 (CH_2), 26.0 (CH_2), 25.9 (CH_2), 21.5 (C**7**-H), 20.8 (C**7**-H).

The spectroscopic properties for this compound were consistent with the data available in the literature.[14]

Alternative Transfer Reagents Screened with 1v

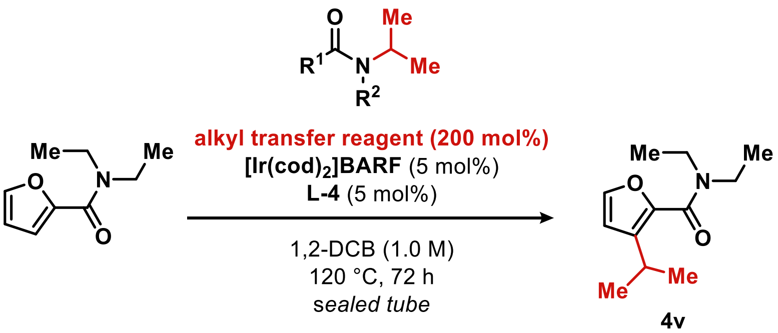

R^1	R^2	Yield
Me	H	0%
Me (C=S)	*i*-Pr	0%
OMe	*i*-Pr	0%
CF_3	*i*-Pr	0%
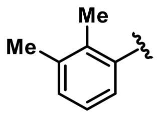	*i*-Pr	0%
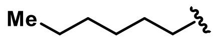	*i*-Pr	14%
Cy	*i*-Pr	0%

Intermolecular N→C Isopropyl Transfer: Substrates (Scheme 4C)

N-Isopropylfuran-2-carboxamide (NH-1a)

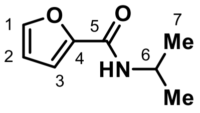

NH-1a

General Procedure A: 2-Furoic acid (2.50 g, 22.3 mmol), isopropylamine (2.50 mL, 29.0 mmol), oxalyl chloride (2.50 mL, 29.0 mmol) and Et_3N (4.70 mL, 33.5 mmol) were employed. Purification by FCC (20% EtOAc/Hex) afforded the title compound (2.63 g, 77%) as a colorless solid; m.p. 119–122 °C (EtOH); ^1H NMR (500 MHz, $CDCl_3$): δ 7.43 (1H, dd, *J* = 1.9, 0.8 Hz, C**1**-H), 7.11 (1H, dd, *J* = 3.5, 0.8 Hz, C**3**-H), 6.50 (1H, dd, *J* = 3.5, 1.9 Hz, C**2**-H), 6.18 (1H, br, NH), 4.27 (1H, dhept, *J* = 8.2, 6.6 Hz, C**7**-H), 1.27 (6H, d, *J* = 6.6 Hz, C**8**-H); ^{13}C NMR (126 MHz, $CDCl_3$): δ 157.6 (C**5**), 148.3 (C**4**), 143.6 (C**1**), 113.9 (C**3**), 112.1 (C**2**), 41.2 (C**6**), 22.9 (C**7**).

The spectroscopic properties for this compound were consistent with the data available in the literature.[15]

N-Methylfuran-2-carboxamide (NH-1b)

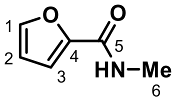

NH-1b

General Procedure A: 2-Furoic acid (1.16 g, 8.92 mmol), 2 M methylamine in THF (5.80 mL,11.6 mmol), 2 M oxalyl chloride in CH_2Cl_2 (5.80 mL, 11.6 mmol) and Et_3N (1.87 mL, 13.4 mmol) were employed. Purification by FCC (0–20% EtOAc/Hex) afforded the title compound (2.45 g, 89%) as a colorless oil which crystallized upon standing; ^1H NMR (500 MHz, $CDCl_3$): δ 7.36 (1H, dd, *J* = 1.8, 0.9 Hz, C**1**-H), 7.04 (1H, dd, *J* = 3.5, 0.9 Hz, C**3**-H), 6.61 (1H, br, NH), 6.42 (1H, dd, *J* = 3.5, 1.8 Hz, C**2**-H), 2.92 (3H, d, *J* = 4.9 Hz, C**6**-H); ^{13}C NMR (126 MHz, $CDCl_3$): δ 159.1 (C**5**), 148.2 (C**4**), 143.9 (C**1**), 113.7 (C**3**), 112.0 (C**2**), 25.9 (C**6**).

The spectroscopic properties for this compound were consistent with the data available in the literature.[16]

5-Methyl-*N*-isopropylfuran-2-carboxamide (NH-1g)

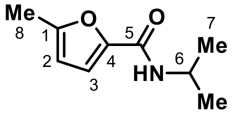

NH-1g

General Procedure A: 5-Methyl-2-furoic acid (0.50 g, 3.96 mmol), isopropylamine (0.44 mL, 5.15 mmol), 2 M oxalyl chloride in CH_2Cl_2 (2.58 mL, 5.15 mmol) and Et_3N (0.83 mL, 5.94 mmol) were employed. Purification by FCC (0–40% EtOAc/Hex) afforded the title compound (372 mg, 56%) as a colorless solid; m.p. 79–81 °C (EtOH); ν_{max}/cm^{-1}: 3260 (br), 2975 (w), 1633 (s), 1541 (s); ^1H NMR (500 MHz, $CDCl_3$): δ 6.99 (1H, d, *J* = 3.4 Hz, C**1**-H), 6.10 (1H, br, NH), 6.09 (1H, m, C**3**-H), 4.26 (1H, m, C**6**-H), 2.35 (3H, s, C**8**-H), 1.26 (6H, d, *J* = 6.6 Hz, C**7**-H);

^{13}C NMR (126 MHz, $CDCl_3$): δ 157.8 (C**5**), 154.1 (C**4**), 146.7 (C**1**), 115.1 (C**3**), 108.4 (C**2**), 41.0 (C**6**), 22.9 (C**7**), 13.8 (C**8**); HRMS: (ESI^+) calculated for $C_9H_{14}NO_2$ 168.1024. Found $[M+H]^+$ 168.1014.

5-Chloro-*N*-isopropylfuran-2-carboxamide (NH-1i)

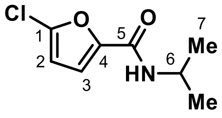

NH-1i

General Procedure A: 5-chloro-2-furoic acid (0.55 g, 3.75 mmol), isopropylamine (0.42 mL, 4.88 mmol), oxalyl chloride (0.40 mL, 4.88 mmol) and Et_3N (0.78 mL, 5.63 mmol) were employed. Purification by FCC (20–40% EtOAc/Hex) afforded the title compound (224 mg, 32%) as a colorless solid; m.p. 78–80 °C (EtOH); ν_{max}/cm^{-1}: 3263 (br), 2974 (m), 1631 (s), 1537 (s); ^{1}H NMR (500 MHz, $CDCl_3$): δ 7.07 (1H, d, J = 3.5 Hz, C**1**-H), 6.28 (1H, d, J = 3.5 Hz, C**3**-H), 6.05 (1H, br, NH), 4.22 (1H, m, C**6**-H), 1.25 (6H, d, J = 6.6 Hz, C**7**-H); ^{13}C NMR (126 MHz, $CDCl_3$): δ 156.5 (C**5**), 147.4 (C**4**), 138.0 (C**1**), 116.1 (C**3**), 109.1 (C**2**), 41.4 (C**6**), 22.9 (C**7**); HRMS: (ESI^+) calculated for $C_8H_{11}{}^{35}ClNO_2$ 188.0478. Found $[M+H]^+$ 188.0480.

***N*-Isopropyl-benzofuran-2-carboxamide (NH-1m)**

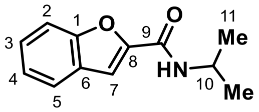

NH-1m

General Procedure A: Benzofuran-2-carboxylic acid (1.00 g, 6.17 mmol), isopropylamine (0.70 mL, 8.02 mmol), oxalyl chloride (0.68 mL, 8.02 mmol) and Et_3N (1.28 mL, 9.23 mmol) were employed. Purification by FCC (0–30% EtOAc/Hex) afforded the title compound (798 mg, 64%) as a colorless solid; m.p. 85–87 °C (EtOH); ^{1}H NMR (500 MHz, $CDCl_3$): δ 7.65 (1H, d, J = 9.6 Hz, C**5**-H), 7.49 (1H, d, J = 8.4 Hz, C**2**-H), 7.45 (1H, s, C**7**-H), 7.39 (1H, t, J = 8.4 Hz, C**3**-H), 7.27 (1H, m, C**4**-H), 7.48 (1H, br, NH), 4.31 (1H, m, C**10**-H), 1.30 (6H, d, J = 7.1 Hz, C**11**-H); ^{13}C NMR (126 MHz, $CDCl_3$): δ 158.1 (C**9**), 154.7 (C**1**), 149.0 (C**8**), 127.7 (C**6**), 126.7 (C**3**), 123.7 (C**4**), 122.7 (C**5**), 111.7 9 (C**2**), 110.2 (C**7**), 41.5 (C**10**), 22.9 (C**11**).

The spectroscopic properties for this compound were consistent with the data available in the literature.[17]

***N*-Isopropylbenzamide (NH-1o)**

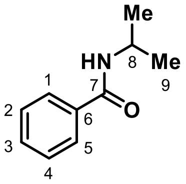

NH-1o

To a solution of isopropylamine (0.43 mL, 5.00 mmol) and Et_3N (0.84 mL, 6.00 mmol) in CH_2Cl_2 (25.0 mL) was added benzoyl chloride (0.58 mL, 5.00 mmol) dropwise at 0 ºC. The resulting mixture was warmed to r.t. and

stirred for 12 h before being quenched by saturated aq. $NaHCO_3$ (25.0 mL) and the layers separated. The aqueous phase was extracted with CH_2Cl_2 (3 × 25.0 mL) and the organic layers combined, washed with brine (25.0 mL), dried (Na_2SO_4), filtered and concentrated *in vacuo*. Purification by FCC (40% EtOAc/Hex) afforded the title compound (810 mg, 99%) as a colorless solid; m.p. 94–96 °C (EtOH); ^{1}H NMR (500 MHz, $CDCl_3$): δ 7.75–7.73 (2H, m, ArH), 7.47 (1H, m, ArH), 7.41 (1H, m, ArH), 6.00 (1H, br, NH), 4.28 (1H, m, C**8**-H), 1.25 (6H, d, *J* = 6.6 Hz, C**9**-H); ^{13}C NMR (126 MHz, $CDCl_3$): δ 166.7 (C**7**), 135.0 (ArC), 131.3 (ArCH), 128.5 (ArCH), 126.8 (ArCH), 41.9 (C**8**), 22.9 (C**9**).

The spectroscopic properties for this compound were consistent with the data available in the literature.[12]

***N*-(1-Phenylethyl)furan-2-carboxamide (1z)**

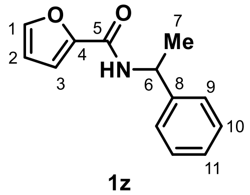

1z

General Procedure A: 2-Furoic acid (1.25 g, 11.2 mmol), 1-phenylethylamine (1.89 mL, 14.5 mmol), oxalyl chloride (1.25 mL, 14.6 mmol) and Et_3N (2.33 mL, 16.7 mmol) were employed. Purification by FCC (20–30% EtOAc/Hex) afforded the title compound (1.22 g, 51%) as a colorless solid; m.p. 82–85 °C (EtOH); ^{1}H NMR (500 MHz, $CDCl_3$): δ 7.42–7.34 (5H, m, ArH), 7.28 (1H, m, *J* = 3.4, 0.9 Hz, C**1**-H), 7.11 (1H, dd, *J* = 1.7, 0.9 Hz, C**3**-H), 6.58 (1H, br, NH), 6.52–6.51 (1H, dd, *J* = 3.4, 1.7 Hz, C**2**-H), 5.30 (qt, *J* = 7.0 Hz, C**6**-H), 1.60 (3H, d, *J* = 7.0 Hz, C**7**-H); ^{13}C NMR (126 MHz, $CDCl_3$): δ 157.5 (C**5**), 148.0 (C**4**), 143.8 (C**8**), 142.9 (ArCH), 128.8 (ArCH), 126.5 (ArCH), 126.3 (ArCH), 114.1 (C**1**), 112.2 (C**3**), 48.4 (C**6**), 21.8 (C**7**).

The spectroscopic properties for this compound were consistent with the data available in the literature.[18]

1-Methyl-*N*-(1-phenylethyl)-1*H*-pyrrole-2-carboxamide (1aa)

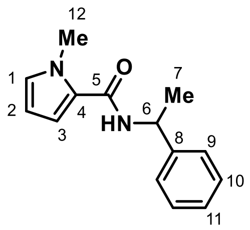

1aa

General Procedure A: *N*-Methylpyrrole-2-carboxylic acid (368 mg, 2.94 mmol), α-methylbenzylamine (0.49 mL, 3.82 mmol), 2 M oxalyl chloride in CH_2Cl_2 (1.91 mL, 3.82 mmol) and Et_3N (0.61 mL, 4.41 mmol) were employed. Purification by FCC (20% EtOAc/Hex) afforded the title compound (436 mg, 65%) as a pale yellow solid; m.p. 98−100 °C (EtOH); ν_{max}/cm^{-1}: 3332 (br), 2979 (w), 1622 (s), 1541 (s); ^{1}H NMR (500 MHz, $CDCl_3$): δ 7.38–7.32 (4H, m, ArH), 7.26 (1H, m, ArH), 6.71 (1H, t, *J* = 1.7 Hz, C**1**-H), 6.54 (1H, dd, *J* = 3.9, 1.7 Hz, C**2**-H), 6.08–6.04 (2H, m, C**3**-H, NH), 5.24 (1H, qt, *J* = 7.1 Hz, C**6**-H), 3.93 (3H, s, C**12**-H), 1.56 (3H, d, *J* = 7.1 Hz, C**7**-H); ^{13}C NMR (126 MHz, $CDCl_3$): δ 161.1 (C**5**), 143.6 (C**8**), 128.7 (ArCH), 128.0 (C**1**), 127.3 (ArCH), 126.1

(Ar<u>CH</u>), 125.7 (C**4**), 111.3 (C**3**), 107.2 (C**2**), 48.5 (C**6**), 36.8 (C**7**), 22.1 (C**12**); HRMS: (ESI$^+$) calculated for $C_{14}H_{17}NO_2$ 229.1341. Found $[M+H]^+$ 229.1335.

***N*-Isopropylthiophene-2-carboxamide (1bb)**

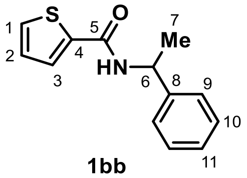

1bb

General Procedure A: Thiophene-2-carboxylic acid (1.00 g, 7.80 mmol), α-methylbenzylamine (1.29 mL, 10.1 mmol), 2 M oxalyl chloride in CH_2Cl_2 (5.10 mL, 10.1 mmol) and Et_3N (1.63 mL, 11.7 mmol) were employed. Purification by FCC (0−40% EtOAc/Hex) afforded the title compound (1.28 g, 71%) as a pale yellow solid; m.p. 128–130 °C (EtOH); ν_{max}/cm^{-1}: 3260 (br), 2978 (w), 1620 (s), 1640 (s); ^{1}H NMR (500 MHz, $CDCl_3$): δ 7.49 (1H, dd, *J* = 3.7, 1.2 Hz, C**1**-<u>H</u>), 7.46 (1H, dd, *J* = 5.0, 1.2 Hz, C**3**-<u>H</u>), 7.40–7.33 (4H, m, Ar<u>H</u>), 7.28 (1H, m, Ar<u>H</u>), 7.05 (1H, dd, *J* = 5.0 Hz, 3.7 Hz, C**2**-<u>H</u>), 6.21 (1H, br, N<u>H</u>), 5.31 (1H, qt, *J* = 7.1 Hz, C**6**-<u>H</u>), 1.60 (1H, d, *J* = 7.1 Hz, C**7**-<u>H</u>); ^{13}C NMR (126 MHz, $CDCl_3$): δ 161.0 (C**5**), 142.9 (C**8**), 139.0 (C**4**), 129.9 (C**1**), 128.8 (Ar<u>CH</u>), 128.0 (C**3**), 127.6 (Ar<u>CH</u>), 127.6 (C**2**), 126.3 (Ar<u>CH</u>), 49.2 (C**6**), 21.7 (C**7**); HRMS: (ESI$^+$) calculated for $C_{13}H_{14}NOS$ 232.0796. Found $[M+H]^+$ 232.0792.

<u>Intermolecular N→C Isopropyl Transfer: Products (Scheme 4C)</u>

3-Isopropyl-*N*-(1-phenylethyl)furan-2-carboxamide (4z)

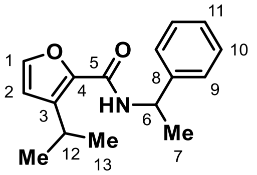

4z

General Procedure C: Compound **1z** (21.5 mg, 0.10 mmol), **6a** (28.6 mg, 0.20 mmol), $[Ir(cod)_2]$BARF (12.6 mg, 0.01 mmol), **L-4** (10.6 mg, 0.01 mmol) and 1,2-DCB (0.10 mL) were employed at 120 °C for 72 h. Purification by FCC (0–40% EtOAc/Hex) afforded the title compound (18.6 mg,72%) as a colorless oil; ν_{max}/cm^{-1}: 3315 (br), 2968 (w), 1639 (m), 1521 (m); ^{1}H NMR (500 MHz, $CDCl_3$): δ 7.40–7.27 (6H, m, Ar<u>H</u>, C**1**-<u>H</u>), 6.55 (1H, br, N<u>H</u>), 6.44 (1H, d, *J* = 1.8 Hz, C**2**-<u>H</u>), 5.28 (1H, quint, *J* = 6.9 Hz, C**6**-<u>H</u>), 3.79 (1H, hept, *J* = 6.9 Hz, C**12**-<u>H</u>), 1.58 (3H, d, *J* = 6.9 Hz, C**7**-<u>H</u>), 1.19 (3H, d, *J* = 6.9 Hz, C**13**-<u>H</u>), 1.17 (3H, d, *J* = 6.9 Hz, C**9**-<u>H</u>); ^{13}C NMR (126 MHz, $CDCl_3$): δ 158.7 (C**5**), 143.4 (C**8**), 142.4 (C**1**), 140.7 (C**4**), 139.0 (C**3**), 128.7 (Ar<u>CH</u>), 127.3 (Ar<u>CH</u>), 126.2 (Ar<u>CH</u>), 111.2 (C**2**), 48.1 (C**6**), 24.2 (C**12**), 23.2 (C**13**), 22.1 (C**7**); HRMS: (ESI$^+$) calculated for $C_{16}H_{20}NO_2$ 258.1494. Found $[M+H]^+$ 258.1495.

3-Isopropyl-1-methyl-*N*-(1-phenylethyl)-1*H*-pyrrole--2-carboxamide (4aa)

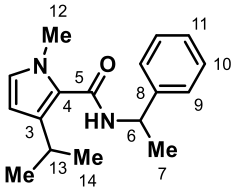

4aa

General Procedure C: Compound **1aa** (22.8 mg, 0.10 mmol), **6a** (28.6 mg, 0.20 mmol), [Ir(cod)$_2$]BARF (12.6 mg, 0.01 mmol), **L-4** (10.6 mg, 0.01 mmol) and 1,2-DCB (0.10 mL) were employed at 120 °C for 72 h. Purification by FCC (0–30% EtOAc/Hex) afforded the title compound (17.6 mg, 63%) as a brown oil; ν_{max}/cm^{-1}: 3315 (br), 2930 (w), 1629 (s), 1490 (m); ^{1}H NMR (500 MHz, CDCl$_3$): δ 7.38–7.34 (5H, m, ArH), 6.59 (1H, d, *J* = 2.7 Hz, C**1**-H), 6.00 (1H, d, *J* = 2.7 Hz, C**2**-H), 5.28 (1H, m, C**6**-H), 3.76 (3H, s, C**12**-H), 3.07 (1H, hept, *J* = 6.8 Hz, C**13**-H), 1.58 (3H, d, *J* = 6.9 Hz, C**7**-H), 1.24 (3H, d, *J* = 6.8 Hz, C**14**-H), 1.20 (3H, d, *J* = 6.8 Hz, C**14**-H); ^{13}C NMR (126 MHz, CDCl$_3$): δ 162.0 (C**5**), 143.4 (C**4**), 133.8 (C**8**), 128.7 (ArCH), 127.4 (ArCH), 126.1 (ArCH), 125.8 (ArCH), 122.9 (C**1**), 111.3 (C**3**), 104.9 (C**2**), 49.0 (C**6**). 36.3 (C**12**), 26.4 (C**13**), 24.5 (C**14**), 22.2 (C**7**); HRMS: (ESI$^+$) calculated for $C_{17}H_{23}NO_2$ 271.1810. Found [M+H]$^+$ 271.1815.

3-Isopropyl-*N*-(1-phenylethyl)thiophene-2-carboxamide (4bb)

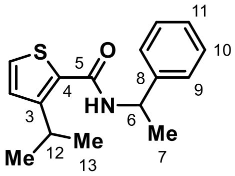

4bb

General Procedure C: Compound **1bb** (28.6 mg, 0.10 mmol), **6a** (28.6 mg, 0.20 mmol), [Ir(cod)$_2$]BARF (12.6 mg, 0.01 mmol), **L-4** (10.6 mg, 0.01 mmol) and 1,2-DCB (0.10 mL) were employed at 120 °C for 72 h. Purification by FCC (10–40% EtOAc/Hex) afforded the title compound (21.2 mg, 78%) as a colorless oil; ν_{max}/cm^{-1}: 3302 (br), 2967 (w), 1636 (s), 1537 (m); ^{1}H NMR (500 MHz, CDCl$_3$): δ 7.43–7.35 (4H, m, ArH), 7.34–7.24 (2H, m, C**1**-H, ArH), 7.07 (1H, d, *J* = 5.0 Hz, C**2**-H), 6.05 (1H, br, NH), 5.29 (1H, quint, *J* = 6.9 Hz, C**6**-H), 3.80 (1H, hept, *J* = 7.0 Hz, C**12**-H), 1.62 (3H, d, *J* = 6.9 Hz, C**7**-H), 1.27 (6H, d, *J* = 7.0, 5.3 Hz, C**13**-H); ^{13}C NMR (126 MHz, CDCl$_3$): δ 162.3 (C**5**), 153.5 (C**8**), 143.2 (C**4**), 129.3 (C**1**), 128.8 (ArC), 127.7 (C**3**), 127.4 (C**2**), 126.2 (ArCH), 126.2 (ArCH), 49.3 (C**6**), 27.9 (C**12**), 23.7 (C**13**), 22.0 (C**7**); HRMS: (ESI$^+$) calculated for $C_{16}H_{20}NOS$ 274.1266. Found [M+H]$^+$ 274.1266.

Intermolecular N→C *sec*-Butyl Transfer (Scheme 4D)

***N,N*-Di-*sec*-butylacetamide (6f)**

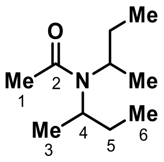

6f

Acetyl chloride (3.00 mL, 42.0 mmol) was added slowly to a solution of di-*sec*-butylamine (10.80 mL, 63.0 mmol) in Et_2O (20.0 mL) at 0 °C and the mixture stirred at r.t. for 16 h. H_2O (20.0 mL) was added and the resulting solution extracted with Et_2O (3 × 20.0 mL). The combined organic layers were washed with brine (20.0 mL), dried ($NaSO_4$) and concentrated *in vacuo*. Purification by distillation (60 ºC at 0.15 mbar) afforded the title compound as a colorless oil (3.72 g, 52%, 1:1 mixture of diastereoisomers *A*:*B*); ν_{max}/cm^{-1}: 2967 (w), 1635 (s), 1431 (m); ^1H NMR (500 MHz, $CDCl_3$): δ 3.56 (2H, br, C**4**-H, *A*), 2.96 (2H, br, C**4**-H, *B*), 2.05 (6H, s, C**1**-H, *A*+*B*), 2.03–1.41 (8H, m, C**5**-H, *A*+*B*), 1.33–1.30 (6H, m, C**3**-H, *B*), 1.19–1.15 (6H, m, C**3**-H, *A*), 0.92 (6H, br, C**6**-H, *B*), 0.84 (6H, br, C**6**-H, *A*); ^{13}C NMR (126 MHz, $CDCl_3$)*: δ 170.1 (C**2**, *A*+*B*), 56.1 (C**4**, *A*), 52.0 (C**4**, *B*), 28.1 (C**5**, *A*), 27.7 (C**5**, *B*), 24.0 (C**1**, *A*+*B*), 19.3 (C**3**, *A*), 17.9 (C**3**, *B*), 12.2 (C**6**, *A*), 11.8 (C**6**, *B*); HRMS: (ESI$^+$) calculated for $C_{10}H_{22}NO$ 172.1701 Found $[M+H]^+$ 172.1697.

**additional peaks can be seen in the ^{13}C NMR accounting for rotamers due to hindered rotation around the amide*

3-(*sec*-Butyl)-*N*-(1-phenylethyl)furan-2-carboxamide (4z')

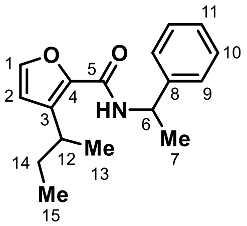

4z'

General Procedure C: Compound **4z** (21.5 mg, 0.10 mmol), **6f** (34.3 mg, 0.20 mmol), $[Ir(cod)_2]BARF$ (12.6 mg, 0.01 mmol), **L-4** (10.6 mg, 0.01 mmol) and 1,2-DCB (0.10 mL) were employed at 120 °C for 72 h. Purification by FCC (10–30 EtOAc/Hex) afforded the title compound (24.7 mg, 91%) as a colorless oil; The d.r. of this compound was determined to be 5:1 by ^1H NMR analysis of the crude reaction mixture. An analytically pure sample of the major diastereoisomer was isolated by FCC. Characteristic signals for minor diastereoisomer: ^1H NMR (500 MHz, $CDCl^3$): δ 6.52 (1H, br, NH), 6.38 (1H, d, J = 1.8 Hz, C**2**-H), 5.13 (1H, m, C**6**-H). Data for the major diastereoisomer: ν_{max}/cm^{-1}: 2994 (m), 1668 (m), 1555 (m); ^1H NMR (500 MHz, $CDCl_3$): δ 7.39–7.26 (6H, m, ArH, C**1**-H), 6.55 (1H, br, NH), 6.40 (1H, d, J = 1.9 Hz, C**2**-H), 5.28 (1H, quint, J = 6.9 Hz, C**6**-H), 3.60 (1H, m, C**12**-H), 1.58 (3H, d, J = 6.9 Hz, C**7**-H), 1.55–1.49 (2H, m, C**14**-H), 1.16 (3H, dd, J = 10.5, 7.0 Hz, C**13**-H), 0.85 (3H, dt, J = 10.3, 7.4 Hz, C**15**-H); 13C NMR (126 MHz, $CDCl_3$): δ 158.7 (C**5**), 151.1 (C**4**), 142.4 (C**1**), 128.7

(C**8**), 127.4 (ArCH), 126.3 (ArCH), 126.2 (ArCH), 124.9 (C**3**), 111.4 (C**2**), 48.1 (C**6**), 30.8 (C**7**), 30.1 (C**14**), 22.1 (C**12**), 20.8 (C**15**), 11.9 (C**13**); HRMS: (ESI$^+$) calculated for $C_{17}H_{22}NO_2$ 272.1651. Found $[M+H]^+$ 272.1651.

Application to the Synthesis of an Antiparasitic Compound (Scheme 4E)

Synthesis of Heterocyclic Core

Methyl benzofuran-2-carboxylate (8)

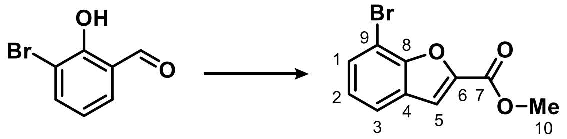

8

To a mixture of 3-bromo-2-hydroxybenzaldehyde (5.00 g, 24.9 mmol) and methyl chloroacetate (2.40 mL, 27.4 mmol) in acetone (50.0 mL) was added tetra-*n*-butylammonium iodide (1.01 g, 2.74 mmol) and potassium carbonate (13.8 g, 99.6 mmol) and the mixture heated to 130 °C for 16 h. The reaction mixture was filtered through celite and the celite washed with acetone (30.0 mL). Purification by FCC (20% EtOAc/Hex) afforded the title compound (1.77 g, 30%) as a colorless solid; m.p. 80−82 °C (hexane); ^{1}H NMR (500 MHz, $CDCl_3$): 7.64–7.59 (2H, m, C**1**-H, C**3**-H), 7.58 (1H, s, C**5**-H), 7.19 (1H, t, J = 7.5 Hz, C**2**-H), 3.99 (3H, s, C**10**-H); ^{13}C NMR (126 MHz, $CDCl_3$): δ 159.6 (C**7**), 153.0 (C**8**), 146.3 (C**6**), 130.6 (C**4**), 128.2 (C**1**), 125.2 (C**2**), 122.1 (C**3**), 114.5 (C**5**), 105.0 (C**9**), 52.5 (C**10**).

The spectroscopic properties for this compound were consistent with the data available in the literature.[19]

Methyl-7-(3,5-dichlorophenyl)benzofuran-2-carboxylate (9)

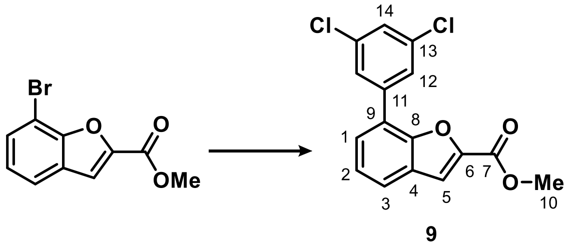

9

To a mixture of **8** (1.50 g, 5.53 mmol) and 3,5-dichlorophenylboronic acid (1.23 g, 6.47 mmol) in THF (8.00 mL) and H_2O (2.00 mL) was added Pd(dtbpf)Cl_2 (180 mg, 0.28 mmol) and then K_3PO_4 (2.30 g, 11.0 mmol). The resulting reaction mixture was stirred at r.t. for 16 h under an atmosphere of N_2 then concentrated *in vacuo*. Purification by FCC (0–20% EtOAc/Hex) afforded the title compound (1.40 g, 74%) as a colorless solid; m.p.

149−153 °C (toluene); ν_{max}/cm^{-1}: 2980 (w), 1735 (m), 1545 (m); ^{1}H NMR (500 MHz, $CDCl_3$): 7.76 (2H, d, *J* = 2.0 Hz, C**12**-H), 7.71 (1H, dd, *J* = 7.8, 1.3 Hz, C**3**-H), 7.60 (1H, s, C**5**-H), 7.56 (1H, dd, *J* = 7.6, 1.3 Hz, C**1**-H), 7.44−7.37 (2H, m, C**2**-H, C**14**-H), 3.98 (3H, s, C**10**-H); ^{13}C NMR (126 MHz, $CDCl_3$): δ 160.0 (C**7**), 152.6 (C**4**), 146.0 (C**6**), 138.5 (C**8**), 135.3 (C**11**), 128.0 (C**13**), 128.0 (C**2**), 127.0 (C**12**), 126.9 (C**1**), 124.4 (C**14**), 123.8 (C**9**), 123.2 (C**3**), 114.1 (C**5**), 52.5 (C**10**); HRMS: (ESI$^+$) calculated for $C_{16}H_{11}{}^{35}Cl_2O_3$ 321.0085. Found $[M+H]^+$ 321.0079.

7-(3,5-Dichlorophenyl)benzofuran-2-carboxylic acid (10)

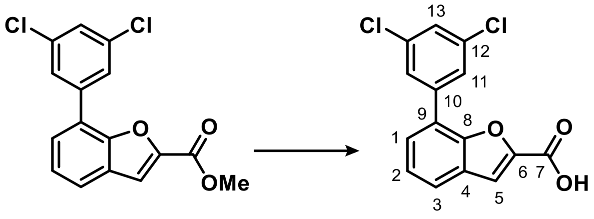

10

To a solution **9** in MeOH (6.00 mL) was added KOH (0.35 g, 6.22 mmol) and the reaction mixture stirred at r.t. for 24 h. The reaction was quenched with 1 M aq. HCl and adjusted to a pH ~ 3, then CH_2Cl_2 (10.0 ml) was added and the layers separated. The aqueous layer was extracted with CH_2Cl_2 (3 × 10.0 mL), the organic layers combined, dried (Na_2SO_4), filtered and concentrated *in vacuo* to afford the target compound as a colorless solid which was used directly in the subsequent step; ^{1}H NMR (500 MHz, MeOH): δ 10.0–9.98 (2H, m, C**11**-H), 9.91 (1H, m, C**3**-H), 9.81–9.75 (2H, m, C**1**-H, C**5**-H), 9.62–9.53 (2H, m, C**2**-H, C**13**-H).

7-(3,5-Dichlorophenyl)-*N*,*N*-diisopropylbenzofuran-2-carboxamide (12)

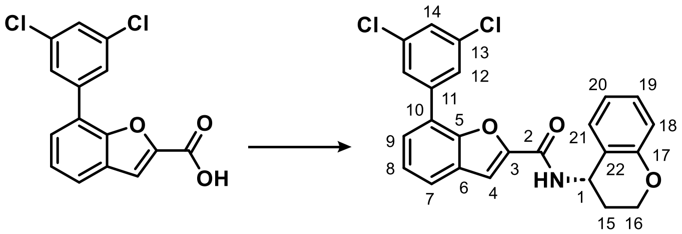

12

To a solution of **10** (132 mg, 0.43 mmol) in CH_2Cl_2 (3.00 mL) was added DMF (0.10 mL, 1.20 mmol) and the reaction cooled to 0 °C and stirred for 5 min. Oxalyl chloride (0.05 mL, 0.56 mmol) was added dropwise over 5 min and the reaction mixture stirred at r.t. for 16 h under an atmosphere of N_2. The resulting mixture was concentrated *in vacuo* to afford the acid chloride which was used directly without further purification.

To a solution of **11** (83.5 mg, 0.56 mmol) and Et_3N (0.09 mL, 0.65 mmol) in CH_2Cl_2 (3.00 mL) was added the acid chloride (assuming 0.43 mmol) in CH_2Cl_2 (3.00 mL) dropwise at 0 °C. The reaction mixture was then stirred at 30 ºC for 16 h under an atmosphere of N_2. H_2O (10.0 mL) was added and the solution extracted with CH_2Cl_2 (3 × 10.0 mL). The organic layers were combined, washed with saturated aq. $NaHCO_3$ (10.0 mL) then H_2O (10.0

mL), dried (Na_2SO_4), filtered and concentrated *in vacuo*. Purification by FCC (0–40% EtOAc/Hex) afforded the title compound (147 mg, 78%) as a colorless solid; $[\alpha]_D^{20}$ +3.4 (c = 0.30, $CHCl_3$); m.p. decomposition at 205 °C (EtOH); ν_{max}/cm^{-1}: 3260 (br), 2982 (m), 1638 (m); ^{1}H NMR (500 MHz, $CDCl_3$): 7.71 (1H, dd, *J* = 7.8, 1.2 Hz, C**7**-H), 7.65 (2H, d, *J* = 1.8 Hz, C**12**-H), 7.59 (1H, s, C**4**-H), 7.51 (1H, dd, *J* = 7.8, 1.2 Hz, C**9**-H), 7.43 (1H, t, *J* = 7.8 Hz, C**8**-H), 7.39 (1H, t, *J* = 1.8 Hz, C**14**-H), 7.32 (1H, m, C**18**-H), 7.21 (1H, m, C**20**-H), 6.94 (1H, td, *J* = 7.5, 1.4 Hz, C**19**-H), 6.87 (1H, dd, *J* = 8.3, 1.3 Hz, C**21**-H), 6.75 (1H, d, *J* = 7.9 Hz, NH), 5.39 (1H, dt, *J* = 8.2, 7.9 Hz, C**1**-H), 4.34 (1H, ddd, *J* = 11.6, 6.9, 3.1 Hz, C**16**-H), 4.24 (1H, ddd, *J* = 11.6, 8.7, 2.8 Hz, C**16**-H), 2.39 (1H, dddd, *J* = 14.0, 8.7, 6.9, 3.4 Hz, C**15**-H), 2.18 (1H, m, C**15**-H); ^{13}C NMR (126 MHz, $CDCl_3$): δ 158.0 (C**2**), 155.1 (C**17**), 151.6 (C**5**), 148.8 (C**3**), 138.6 (C**10**), 135.4 (C**13**), 129.5 (C**19**), 129.0 (C**18**), 128.6 (C**6**), 128.0 (C**14**), 126.8 (C**12**), 126.4 (C**9**), 124.5 (C**8**), 123.4 (C**11**), 123.2 (C**7**), 121.6 (C**22**), 121.1 (C**20**), 117.4 (C**21**), 111.0 (C**4**), 63.2 (C**15**), 43.8 2(C**1**), 29.1 (C**16**); HRMS: (CI$^+$) calculated for $C_{24}H_{18}{}^{35}Cl_2NO_3$ 438.0658. Found $[M+H]^+$ 438.0654.

Synthesis of Amine Component

(*R*)-*N*-(Chroman-4-ylidene)-2-methylpropane-2-sulfinamide (S7)

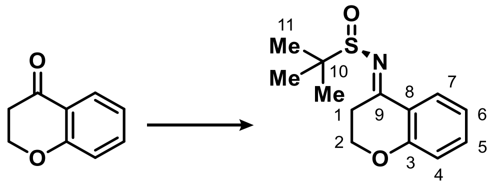

S7

To a mixture of (*R*)-(+)-*t*-butylsulfinamide (4.01 g, 18.9 mmol) and $Ti(OEt)_4$ (8.62 g, 37.8 mmol) in THF (100 mL) was added 4-chromanone (3.36 g, 22.7 mmol) and the resulting mixture stirred at 60 ºC for 16 h. H_2O (100 mL) was added and the aqueous layer extracted with Et_2O (3 × 100 mL). The organic extracts were combined, dried ($MgSO_4$), filtered and concentrated *in vacuo*. Purification by FCC (20% EtOAc/Hex) afforded the title compound (3.54 g, 75%) as a yellow oil; ^{1}H NMR (500 MHz, $CDCl_3$): 7.99 (1H, d, *J* = 7.9, C**4**-H), 7.38 (1H, t, *J* = 7.9 Hz, C**5**-H), 6.97 (1H, t, *J* = 7.9 Hz, C**6**-H), 7.91 (1H, d, *J* = 7.9 Hz, C**7**-H), 4.40–4.28 (2H, m, C**2**-H), 3.50 (1H, m, C**1**-H), 3.27 (1H, m, C**1**-H), 1.32 (9H, s, C**11**-H); ^{13}C NMR (126 MHz, $CDCl_3$): δ 169.6 (C**9**), 159.2 (C**3**), 134.2 (C**5**), 126.9 (C**4**), 121.3 (C**6**), 121.1 (C**8**), 118.0 (C**7**), 65.6 (C**2**), 58.0 (C**10**), 30.7 (C**1**), 22.6 (C**11**).

The spectroscopic properties for this compound were consistent with the data available in the literature.[20]

***(S)*-Chroman-4-aminium chloride (11)**

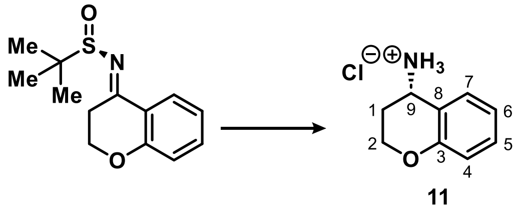

11

To a solution of **S5** (500 mg, 1.99 mmol) in THF (50.0 mL) at -78 °C was added a solution of 1 M L-Selectride® in THF (6.00 mL, 6.00 mmol). The reaction mixture was warmed to r.t. over 3 h, stirred for 16 h and then

concentrated *in vacuo*. $CHCl_3$ (50.0 mL) and MeOH (50.0 mL) were added and resulting mixture filtered through celite. Brine (50.0 mL) was added and the organic layer separated, dried ($MgSO_4$) and concentrated *in vacuo* until approximately 30.0 mL of solvent remained. The solution was cooled to 0 °C and 1 M HCl in Et_2O (3.00 mL) was added dropwise over 5 min. The resulting precipitate was collected by filtration to afford the title compound as a brown solid which was used directly without further purification; ^{1}H NMR (500 MHz, D_2O): δ 7.32–7.27 (2H, m, ArH), 7.00 (1H, m, ArH), 6.88 (1H, dd, J = 8.3, 1.3 Hz, ArH), 4.59 (1H, t, J = 5.5 Hz, C**9**-H), 4.30–4.19 (2H, m, C**2**-H), 2.37 (1H, m, C**1**-H), 2.16 (1H, m, C**1**-H).

To confirm the absolute stereochemistry of ***11****, and hence the absolute stereochemistry of the final target product (****13****), a small sample of compound* ***11*** *was dissolved in* CH_2Cl_2 *and stirred with 4 M aq. NaOH for 30 min. The organic layer was separated, dried (*$MgSO_4$*), filtered and concentrated in vacuo to yield a small quantity of the free-based amine sufficient for optical rotation measurement;* $[\alpha]_D^{20}$ -95.6 (c = 0.20, $CHCl_3$). *By comparison of the specific rotation value to literature data for the antipode the absolute stereochemistry was determined as (S).*[20]

(*S*)-*N*-(Chroman-4-yl)-7-(3,5-dichlorophenyl)-3-isopropylbenzofuran-2-carboxamide (13)

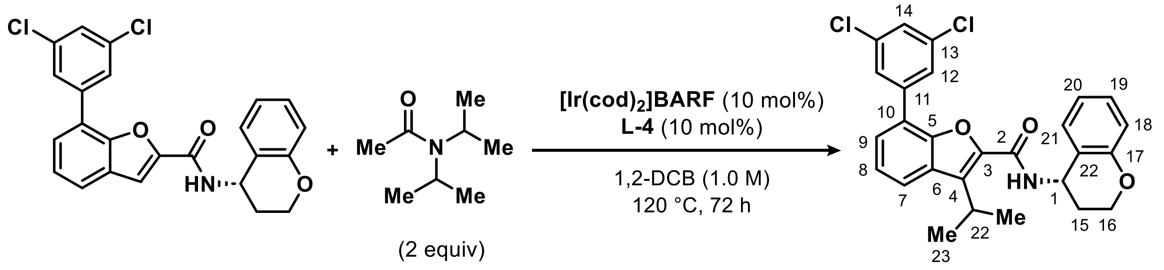

13

General Procedure C: Compound **12** (17.4 mg, 0.05 mmol), [Ir(cod)$_2$]BARF (6.40 mg, 0.005 mmol), **L-4** (5.30 mg, 0.01 mmol) and 1,2-DCB (0.05 mL) were employed at 120 °C for 72 h. Purification by FCC (0–20% EtOAc/Hex) afforded the title compound (15.8 mg, 81%) as a colorless oil; $[\alpha]_D^{21}$ +25.66 (c = 0.34, $CHCl_3$); ν_{max}/cm^{-1}: 3433 (br), 2932 (w), 1663 (s), 1586 (m); ^{1}H NMR (500 MHz, $CDCl_3$): δ 7.85 (1H, dd, J = 8.0, 1.4 Hz, C**7**-H), 7.63 (2H, d, J = 1.8 Hz, C**12**-H), 7.49 (1H, dd, J = 7.5, 1.4 Hz, C**9**-H), 7.43–7.30 (3H, m, C**8**-H, C**14**-H, C**18**-H), 7.21 (1H, m, C**20**-H), 6.94 (1H, td, J = 7.5, 1.4 Hz, C**19**-H), 6.86 (1H, dd, J = 8.2, 1.4 Hz, C**21**-H), 6.78 (1H, d, J = 7.8 Hz, NH), 5.35 (1H, m, C**1**-H), 4.36–4.29 (2H, m, C**16**-H), 4.25 (1H, m, C**22**-H), 2.38 (1H, m, C**15**-H), 2.18 (1H, m, C**15**-H), 1.51 (6H, dd, J = 7.1, 3.6 Hz, C**23**-H); ^{13}C NMR (126 MHz, $CDCl_3$): δ 159.3 (C**2**), 155.1 (C**17**), 150.5 (C**5**), 141.4 (C**3**), 138.8 (C**10**), 135.3 (C**13**), 132.9 (C**4**), 129.4 (C**20**), 129.2 (C**8**-H, C**14**-H or C**18**-H), 128.7 (C**6**), 127.8 (C**8**-H, C**14**-H or C**18**-H), 126.8 (C**12**), 126.0 (C**9**), 123.6 (C**8**-H, C**14**-H or C**18**-H), 123.3 (C**7**), 121.8 (C**11**), 121.1 (C**19**), 117.3 (C**21**), 63.2 (C**22**), 43.4 (C**1**), 29.2 (C**15**), 24.7 (C**16**), 22.1 (C**23**); HRMS: (ESI$^+$) calculated for $C_{27}H_{24}{}^{35}Cl_2NO_3$ 480.1133 Found $[M+H]^+$ 480.1136.

7-(3,5-Dichlorophenyl)-*N*,3-diisopropylbenzofuran-2-carboxamide (S8)

*To investigate the potential for the intermolecular transfer event to access the final target compound (**13**), a model substrate was tested using General Procedure B in the first instance to access **S8**.*

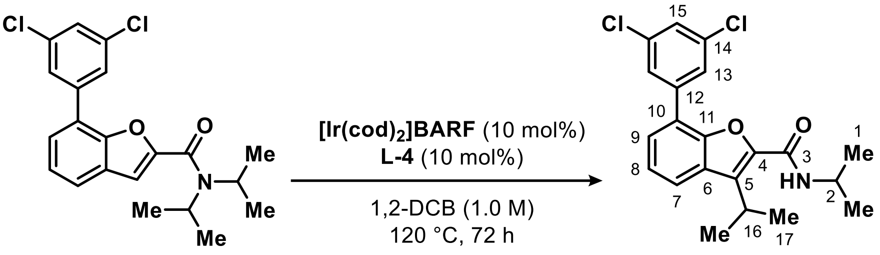

S8

General Procedure B: 7-(3,5-dichlorophenyl)-*N*,*N*-diisopropylbenzofuran-2-carboxamide (19.5 mg, 0.05 mmol), $[Ir(cod)_2]$BARF (6.40 mg, 0.05 mmol), **L-4** (5.30 mg, 0.05 mmol) and 1,2-DCB (0.05 mL) were employed at 120 °C for 72 h. Purification by FCC (0–40% EtOAc/Hex) afforded the title compound (12.1 mg, 62%) as a colorless oil; ν_{max}/cm^{-1}: 3440 (br), 2968 (w), 1666 (s), 1602 (m); ^{1}H NMR (500 MHz, $CDCl_3$): δ 7.84 (1H, dd, *J* = 7.7, 1.2 Hz, C**9**-H), 7.72 (2H, d, *J* = 1.8 Hz, C**13**-H), 7.50 (1H, dd, *J* = 7.7, 1.2 Hz, C**7**-H), 7.42 (1H, t, *J* = 1.8 Hz, C**15**-H), 7.35 (1H, t, *J* = 7.7 Hz, C**8**-H), 6.35 (1H, d, *J* = 8.1 Hz, NH), 4.35–4.22 (2H, m, C**2**-H, C**16**-H), 1.46 (6H, d, *J* = 7.2 Hz, C**1**-H), 1.29 (6H, d, *J* = 6.6 Hz, C**17**-H); ^{13}C NMR (126 MHz, $CDCl_3$): δ 159.1 (C**3**), 150.4 (C**11**), 141.8 (C**4**), 139.0 (C**5**), 135.3 (C**14**), 132.1 (C**6**), 128.8 (C**12**), 127.7 (C**15**), 126.9 (C**13**), 125.6 (C**7**), 123.5 (C**8**), 123.3 (C**9**), 114.0 (C**10**), 41.3 (C**16**), 24.6 (C**2**), 22.8 (C**17**), 22.0 (C**1**); HRMS: (ESI$^+$) calculated for $C_{21}H_{22}{}^{35}Cl_2NO_2$ 390.1028. Found $[M+H]^+$ 390.1031.

7-(3,5-Dichlorophenyl)-*N*-isopropylbenzofuran-2-carboxamide (S9)

*Following on from the testing of the model substrate, the target substrate was subsequently screened using General Procedure B. Unfortunately, intramolecular N⟶C alkyl transfer did not occur but rather dealkylation of the chromanyl unit as 2H-chromene to give **S9**. Note: 2H-chromene was also observed in the crude reaction mixture of **S9**.*

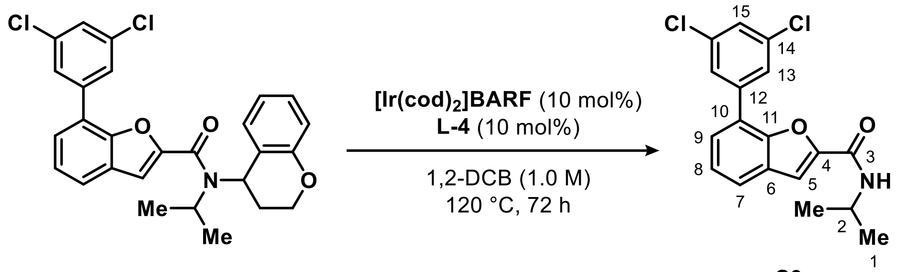

General Procedure B: *N*-(Chroman-4-yl)-7-(3,5-dichlorophenyl)-*N*-isopropylbenzofuran-2-carboxamide (48.0 mg, 0.10 mmol), $[Ir(cod)_2]BARF$ (12.6 mg, 0.01 mmol), **L-4** (10.6 mg, 0.01 mmol) and 1,2-DCB (0.10 mL) were employed at 120 °C for 72 h. Purification by FCC (20–30% EtOAc/Hex) afforded the title compound (41.0 mg, 85%) as a colorless oil; ν_{max}/cm^{-1}: 2936 (w), 1629 (s), 1585 (s), 1488 (m); ^{1}H NMR (500 MHz, $CDCl_3$): δ 7.72 (2H, d, *J* = 1.8 Hz, C**13**-H), 7.70 (1H, dd, *J* = 7.7, 1.2 Hz, C**9**-H), 7.54–7.50 (2H, m, C**7**-H, C**5**-H), 7.43 (1H, t, *J* = 1.8 Hz, C**15**-H), 7.39 (1H, t, *J* = 7.7 Hz, C**8**-H), 6.30 (1H, d, *J* = 8.1 Hz, NH), 4.30 (1H, dhept, *J* = 8.1, 6.6 Hz, C**2**-H), 1.30 (6H, d, *J* = 6.6 Hz, C**1**-H); ^{13}C NMR (126 MHz, $CDCl_3$): δ 157.7 (C**3**), 151.5 (C**11**), 149.3 (C**4**), 138.9 (C**6**), 135.4 (C**14**), 128.7 (C**15**), 127.9 (C**10**), 126.9 (C**13**), 126.0 (C**5**), 124.4 (C**8**), 123.2 (C**9**), 123.1 (C**12**), 110.4 (C**7**), 41.7 (C**2**), 22.8 (C**1**); HRMS: (ESI^{+}) calculated for $C_{18}H_{15}{}^{35}Cl_2NO_2$ 348.0558. Found $[M+H]^{+}$ 348.0561.

Computational Chemistry

This work made use of the Barkla High Performance Computing facilities at the University of Liverpool, using Gaussian 16.[21] Geometry and transition state optimisations were performed at the 6-311G(d,p)/m06L level, with Ir described by the Stuttgart RSC effective core potential (ECP).[22–24] Full geometry optimisations were carried out in chlorobenzene using an implicit polarisable continuum model (PCM).[25] Vibrational frequencies were calculated at the same level of theory as the optimisations to assign stationary points as either minima or transition structures, as characterised by the presence of a zero or a single imaginary frequency respectively. The connectivity of the transition states was confirmed by the relaxation of each transition state towards both the reactant and the product or, by IRC calculations.[26]

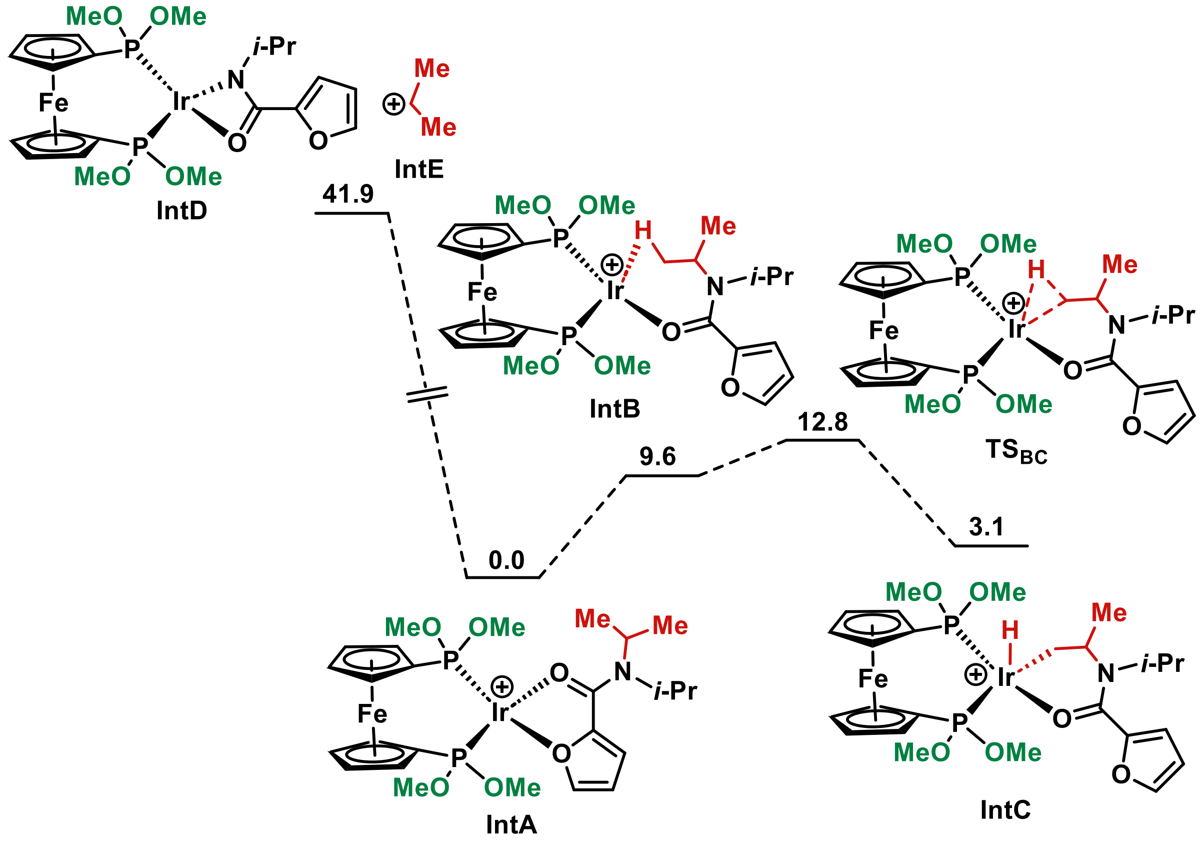

Figure S1. Intermediates and transition states for C–H oxidative addition and Ir-mediated elimination of a propane cation. Energies are Gibbs energies at 298.15 K in kcal mol^{-1}.

Key points:

- The ionization energy for the direct release of the propane cation from **IntA** is high in energy.
- Oxidative addition of the methyl unit is low in energy.
- Because a simplified ligand system has been used, no conclusions can be drawn regarding reversibility etc. of key steps in the "real" catalytic system.

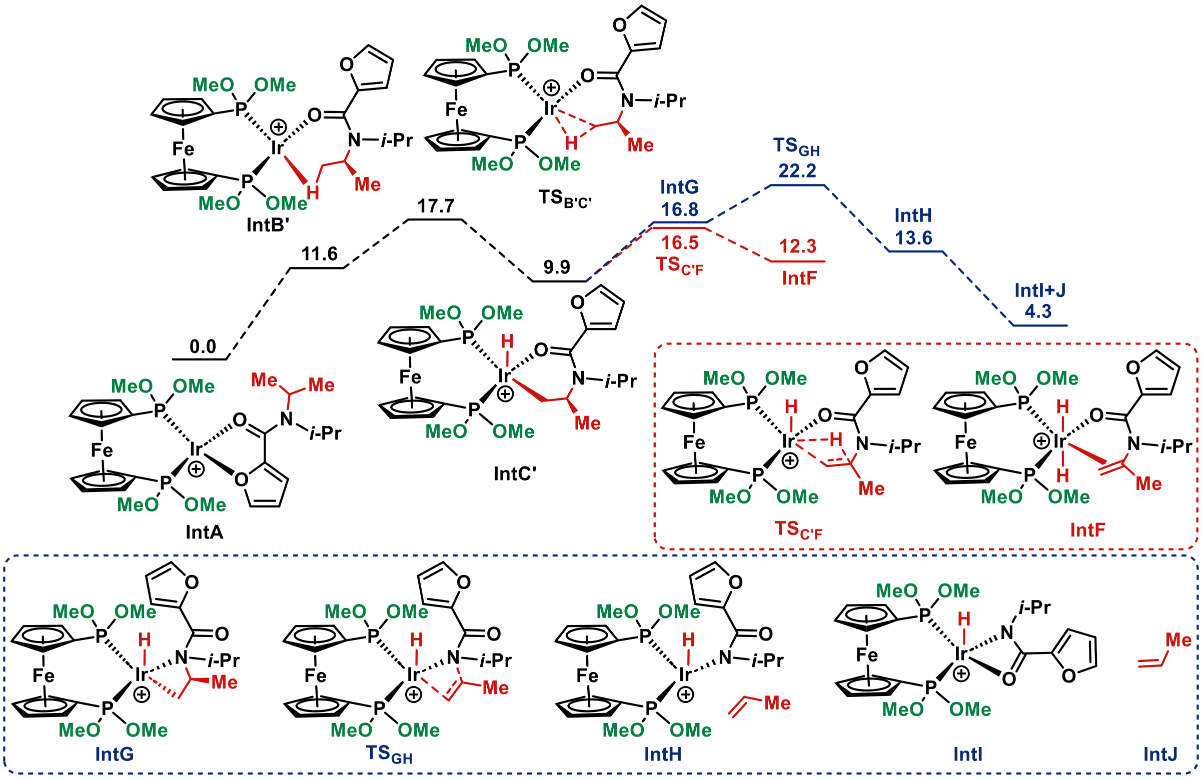

Figure S2. Intermediates and transition states for C–H oxidative addition, followed by β-hydride (red) or β-*N* elimination (blue). Energies are Gibbs energies at 298.15 K in kcal mol^{-1}.

Key points:

- A different orientation of the β-hydrogen is required for β-hydride elimination (**IntC** vs **IntC'**), and this requires a higher energy (but easily accessible) C-H oxidative addition pathway versus Figure S1.
- β-Hydride elimination from **IntC'** to **IntF** is viable and reversible.
- β-Nitrogen elimination from **IntG** to **IntH** is energetically accessible and is driven through elimination of propene.
- Because a simplified ligand system has been used, no conclusions can be drawn regarding reversibility etc. of key steps in the "real" catalytic system.

Cartesian Coordinates and Energies

All energies are quoted in electronic Hartree's.

IntA

Gibbs Energy (298.15 K): −3531.52151

C	1.868719000	-3.347889000	-0.218438000	C	3.862660000	-2.629356000	-0.908516000
C	3.009507000	-3.759993000	-0.821530000	C	3.207593000	-1.571385000	-0.342604000

O	1.963202000	-2.020704000	0.080632000	H	6.364206000	0.353951000	1.620729000
C	3.337596000	-0.120501000	-0.182444000	H	7.063933000	1.445234000	0.410873000
N	4.495643000	0.530371000	-0.339056000	H	5.653182000	-0.500705000	-2.557807000
O	2.287610000	0.531951000	0.078286000	H	7.290072000	-0.660941000	-1.900404000
C	5.810457000	-0.147340000	-0.419144000	H	6.589152000	0.939381000	-2.131414000
C	4.483723000	2.019927000	-0.440976000	H	4.769761000	2.308464000	1.694920000
C	6.780030000	0.409605000	0.612363000	H	4.342608000	3.767307000	0.796656000
C	6.361501000	-0.088701000	-1.834528000	H	3.804941000	3.571599000	-1.750140000
C	4.150901000	2.694100000	0.881326000	H	3.845145000	1.981806000	-2.516430000
C	3.610456000	2.508069000	-1.587294000	H	2.548215000	2.388090000	-1.371897000
Ir	0.358375000	-0.381078000	0.392066000	H	-1.830273000	-2.104640000	3.688402000
P	-1.486494000	-1.511761000	0.668820000	H	-0.906653000	-0.649684000	3.213370000
P	-0.753446000	1.473898000	0.587666000	H	-2.565573000	-0.487948000	3.846096000
O	-2.472834000	-1.186779000	1.932495000	H	-2.684861000	-4.158606000	-0.070668000
C	-1.900543000	-1.107173000	3.243039000	H	-1.698494000	-5.030988000	1.128941000
O	-1.119346000	-3.101830000	0.828515000	H	-2.894555000	-3.830731000	1.672531000
C	-2.170109000	-4.077954000	0.891226000	H	-1.208023000	4.208482000	-0.203884000
O	0.183056000	2.638575000	-0.048389000	H	0.438962000	4.509737000	-0.802108000
C	-0.144407000	4.031701000	-0.014388000	H	0.135428000	4.466526000	0.948994000
O	-1.042840000	1.837469000	2.144965000	H	-2.322539000	3.488986000	1.826938000
C	-1.742607000	3.000136000	2.613488000	H	-1.021668000	3.707966000	3.028339000
H	0.939759000	-3.811483000	0.073422000	H	-2.420817000	2.676899000	3.405647000
H	3.219972000	-4.759374000	-1.168124000	C	-4.166426000	-1.411616000	-0.461490000
H	4.842039000	-2.614413000	-1.358878000	C	-4.732400000	-1.114724000	-1.731827000
H	5.628840000	-1.185492000	-0.144285000	C	-3.672078000	-0.940378000	-2.667065000
H	5.517578000	2.265659000	-0.695817000	C	-2.439675000	-1.125436000	-1.983024000
H	7.696390000	-0.184837000	0.594179000	C	-2.739311000	-1.418301000	-0.608020000

C	-2.356822000	1.758255000	-0.194517000	H	-3.783171000	-0.664446000	-3.706896000
C	-2.551909000	2.100744000	-1.574698000	H	-1.444959000	-1.021239000	-2.397541000
C	-3.950344000	2.216410000	-1.806292000	H	-1.765129000	2.224541000	-2.307785000
C	-4.629722000	1.939208000	-0.585030000	H	-4.417378000	2.431011000	-2.757955000
C	-3.656070000	1.647325000	0.411296000	H	-5.702178000	1.904835000	-0.449003000
Fe	-3.478017000	0.364419000	-1.122647000	H	-3.849261000	1.357771000	1.436235000
H	-4.701118000	-1.552144000	0.467547000	H	3.101443000	2.555305000	1.144719000
H	-5.786892000	-0.990231000	-1.939167000				

IntB

Gibbs Energy (298.15 K): −3531.506164

C	4.300938000	3.597774000	-1.151620000	C	0.031231000	3.833094000	-0.239276000
C	5.575293000	3.256564000	-0.813599000	O	-0.244553000	1.933790000	1.999925000
C	5.518621000	1.901878000	-0.406350000	C	-0.928258000	2.971899000	2.719814000
C	4.209647000	1.508529000	-0.523819000	H	3.850454000	4.502758000	-1.528536000
O	3.462041000	2.560240000	-0.973279000	H	6.444192000	3.895213000	-0.848125000
C	3.415300000	0.339640000	-0.135891000	H	6.342137000	1.302598000	-0.050025000
N	3.968348000	-0.888166000	-0.075679000	H	5.544112000	-0.365588000	-1.306173000
O	2.221586000	0.581346000	0.174382000	H	4.007620000	-2.782987000	0.696865000
C	5.262873000	-1.236576000	-0.714740000	H	6.020404000	-2.565723000	-2.218728000
C	3.271224000	-1.977881000	0.654097000	H	4.301963000	-2.198120000	-2.409573000
C	5.086246000	-2.401565000	-1.676631000	H	4.845880000	-3.334969000	-1.160163000
C	6.337285000	-1.505037000	0.326765000	H	6.435902000	-0.674813000	1.030441000
C	2.055012000	-2.516233000	-0.104380000	H	7.302863000	-1.653281000	-0.162824000
C	2.966381000	-1.593396000	2.095768000	H	6.124073000	-2.410203000	0.903295000
Ir	0.319651000	-0.524291000	0.163231000	H	2.283157000	-3.379245000	-0.734372000
P	-1.502580000	-1.722495000	0.010191000	H	1.265106000	-2.840510000	0.584275000
P	-0.686431000	1.372065000	0.536250000	H	2.628921000	-2.480836000	2.637645000
O	-2.349070000	-2.091194000	1.367914000	H	3.862689000	-1.213926000	2.593001000
C	-1.609592000	-2.453799000	2.535855000	H	2.179663000	-0.838608000	2.175132000
O	-1.066387000	-3.190746000	-0.573708000	H	-1.081186000	-3.403462000	2.394176000
C	-2.063679000	-4.196552000	-0.794995000	H	-0.884170000	-1.674076000	2.797858000
O	-0.138807000	2.448606000	-0.554014000	H	-2.331385000	-2.567946000	3.344593000

H	-2.731664000	-3.911808000	-1.613518000	C	-3.160943000	2.486314000	-0.448816000
H	-1.532683000	-5.108042000	-1.067384000	C	-4.551240000	2.389927000	-0.168393000
H	-2.652267000	-4.377429000	0.109353000	C	-4.725620000	1.505271000	0.933459000
H	-0.920579000	4.310169000	0.020797000	C	-3.445037000	1.045169000	1.345415000
H	0.431825000	4.306499000	-1.135481000	Fe	-3.749554000	0.566034000	-0.583668000
H	0.743079000	3.963898000	0.580558000	H	-4.702342000	-1.941172000	0.119104000
H	-1.682770000	3.479729000	2.114561000	H	-6.099601000	-0.500794000	-1.684391000
H	-0.182691000	3.697261000	3.049737000	H	-4.345812000	0.727713000	-3.315624000
H	-1.414434000	2.531791000	3.593503000	H	-1.851554000	0.053675000	-2.524079000
C	-4.293538000	-1.370801000	-0.703126000	H	-2.703993000	3.044340000	-1.254745000
C	-5.023770000	-0.609339000	-1.655504000	H	-5.342401000	2.867010000	-0.730737000
C	-4.096251000	0.040804000	-2.518505000	H	-5.672448000	1.191570000	1.351602000
C	-2.782262000	-0.310529000	-2.107073000	H	-3.240768000	0.330288000	2.132448000
C	-2.895450000	-1.189205000	-0.975026000	H	1.655836000	-1.793789000	-0.855235000
C	-2.461674000	1.651318000	0.490020000				

TS$_{BC}$

Gibbs Energy (298.15 K): −3531.501053

C	4.237787000	3.699087000	-0.687189000	O	-2.325270000	-1.953071000	1.553169000
C	5.521975000	3.244404000	-0.708559000	C	-1.632633000	-2.123477000	2.793243000
C	5.442740000	1.849179000	-0.485989000	O	-1.077496000	-3.336325000	-0.236642000
C	4.111550000	1.547106000	-0.344605000	C	-2.130634000	-4.297351000	-0.389017000
O	3.374453000	2.692559000	-0.459410000	O	0.046519000	2.420769000	-0.437522000
C	3.329589000	0.370559000	0.026135000	C	0.052463000	3.838912000	-0.257085000
N	3.783624000	-0.874323000	-0.190826000	O	-0.406712000	1.989560000	2.126435000
O	2.230739000	0.595504000	0.604096000	C	-1.061977000	3.137277000	2.689498000
C	4.881343000	-1.206913000	-1.131368000	H	3.794191000	4.674135000	-0.818025000
C	3.155103000	-1.999855000	0.549119000	H	6.412219000	3.834250000	-0.861593000
C	4.404100000	-2.209270000	-2.171255000	H	6.270752000	1.160726000	-0.417989000
C	6.117674000	-1.687533000	-0.388864000	H	5.113215000	-0.278661000	-1.654308000
C	1.754551000	-2.345510000	0.055464000	H	3.798929000	-2.853825000	0.318437000
C	3.221369000	-1.791850000	2.056091000	H	5.189148000	-2.357466000	-2.916401000
Ir	0.414641000	-0.622063000	0.368333000	H	3.511037000	-1.850971000	-2.688969000
P	-1.426967000	-1.800298000	0.190886000	H	4.184265000	-3.187872000	-1.735663000
P	-0.656684000	1.411428000	0.623763000	H	6.441515000	-0.967711000	0.367005000

H	6.940868000	-1.835854000	-1.091974000
H	5.941811000	-2.644090000	0.112474000
H	1.807593000	-2.934019000	-0.867699000
H	1.249613000	-3.000697000	0.772332000
H	2.948493000	-2.724065000	2.557021000
H	4.233657000	-1.525221000	2.370847000
H	2.536729000	-1.015803000	2.403347000
H	-1.067094000	-3.061462000	2.810714000
H	-0.948594000	-1.286491000	2.979415000
H	-2.390005000	-2.150261000	3.576359000
H	-2.768336000	-4.048043000	-1.242291000
H	-1.650509000	-5.258494000	-0.568750000
H	-2.740392000	-4.362273000	0.516810000
H	-0.953776000	4.235172000	-0.082100000
H	0.448288000	4.268199000	-1.177654000
H	0.705865000	4.117730000	0.574201000
H	-1.847184000	3.529922000	2.038635000
H	-0.317434000	3.915068000	2.870215000
H	-1.507986000	2.837137000	3.639516000
C	-4.158893000	-1.416058000	-0.708847000
C	-4.808841000	-0.745865000	-1.780845000
C	-3.812984000	-0.196864000	-2.637826000
C	-2.536783000	-0.520643000	-2.103235000
C	-2.743020000	-1.280746000	-0.901391000
C	-2.409327000	1.722880000	0.340131000
C	-2.986864000	2.442076000	-0.760741000
C	-4.401442000	2.382277000	-0.630100000
C	-4.711147000	1.629009000	0.537807000
C	-3.490608000	1.214878000	1.138607000
Fe	-3.595617000	0.519868000	-0.747369000
H	-4.634252000	-1.898047000	0.134277000
H	-5.878004000	-0.626528000	-1.894023000
H	-3.995619000	0.410171000	-3.514032000
H	-1.573688000	-0.215508000	-2.492758000
H	-2.435804000	2.905673000	-1.568034000
H	-5.117419000	2.792418000	-1.329475000
H	-5.703613000	1.367826000	0.879401000
H	-3.386764000	0.597144000	2.021874000
H	1.039431000	-1.396146000	-0.897166000

IntC

Gibbs Energy (298.15 K): −3531.516603

C	4.257813000	3.628570000	-0.951208000
C	5.546105000	3.203723000	-0.828565000
C	5.470787000	1.832590000	-0.484531000
C	4.138196000	1.514704000	-0.420658000
O	3.394409000	2.627448000	-0.697295000
C	3.344695000	0.356087000	-0.011691000
N	3.801113000	-0.894908000	-0.137332000
O	2.220463000	0.623068000	0.505107000
C	4.983590000	-1.267643000	-0.952274000
C	3.084019000	-1.996764000	0.563552000
C	4.612193000	-2.310888000	-1.994785000
C	6.132985000	-1.717321000	-0.064938000
C	1.697600000	-2.254208000	-0.012266000
C	3.073500000	-1.787477000	2.072727000
Ir	0.409846000	-0.585861000	0.261413000
P	-1.417614000	-1.813098000	0.068310000
P	-0.671891000	1.492413000	0.526945000
O	-2.239704000	-2.056897000	1.464276000

C	-1.472994000	-2.328373000	2.640972000	H	-1.667260000	-5.211331000	-0.896613000
O	-1.087755000	-3.310006000	-0.473246000	H	-2.695632000	-4.400445000	0.309364000
C	-2.143490000	-4.270046000	-0.625732000	H	-1.053077000	4.308855000	-0.114764000
O	-0.082259000	2.506767000	-0.601712000	H	0.175283000	4.357868000	-1.400458000
C	-0.077061000	3.925864000	-0.431543000	H	0.684624000	4.219073000	0.296493000
O	-0.301806000	2.089755000	1.998380000	H	-1.798804000	3.574905000	2.046356000
C	-0.918735000	3.238110000	2.600577000	H	-0.189881000	4.049624000	2.653830000
H	3.810707000	4.575612000	-1.210837000	H	-1.222925000	2.961518000	3.611602000
H	6.436394000	3.797374000	-0.965972000	C	-4.182319000	-1.395655000	-0.680457000
H	6.299047000	1.170737000	-0.284725000	C	-4.889627000	-0.684896000	-1.687633000
H	5.273891000	-0.363853000	-1.487691000	C	-3.941839000	-0.069965000	-2.553744000
H	3.710531000	-2.873973000	0.369970000	C	-2.637808000	-0.393062000	-2.090445000
H	5.475270000	-2.496436000	-2.638555000	C	-2.778169000	-1.220681000	-0.924314000
H	3.788679000	-1.967959000	-2.625137000	C	-2.450650000	1.773624000	0.405680000
H	4.330600000	-3.267801000	-1.548100000	C	-3.160175000	2.504383000	-0.605749000
H	6.392151000	-0.956016000	0.675319000	C	-4.553153000	2.355205000	-0.359717000
H	7.019152000	-1.916757000	-0.672342000	C	-4.718571000	1.533117000	0.791076000
H	5.889192000	-2.639783000	0.470375000	C	-3.429253000	1.166252000	1.264852000
H	1.769437000	-2.526455000	-1.071374000	Fe	-3.660161000	0.551827000	-0.638633000
H	1.292695000	-3.137638000	0.496900000	H	-4.612123000	-1.933552000	0.153231000
H	2.685848000	-2.688500000	2.555870000	H	-5.964632000	-0.582240000	-1.749060000
H	4.078302000	-1.604515000	2.462407000	H	-4.172468000	0.579162000	-3.387404000
H	2.440606000	-0.948627000	2.375459000	H	-1.700510000	-0.047824000	-2.508098000
H	-0.864811000	-3.232223000	2.527004000	H	-2.708778000	3.032044000	-1.435443000
H	-0.823302000	-1.480046000	2.893397000	H	-5.349019000	2.752281000	-0.975314000
H	-2.182768000	-2.478266000	3.453570000	H	-5.661870000	1.197315000	1.199943000
H	-2.832850000	-3.972400000	-1.421087000	H	-3.213195000	0.511651000	2.100658000

H	0.349763000	-0.375495000	-1.269867000

IntD

Gibbs Energy (298.15 K): −3413.203443

C	6.292532000	1.975760000	-1.156456000
C	6.895499000	0.864841000	-0.653063000
C	5.844795000	0.003717000	-0.245240000
C	4.669686000	0.646692000	-0.530000000
O	4.950607000	1.867430000	-1.081644000
C	3.247437000	0.403834000	-0.286621000
N	2.613037000	-0.743016000	-0.138100000
O	2.494376000	1.446884000	-0.172562000
C	3.050711000	-2.127315000	-0.310039000
C	3.671005000	-2.678734000	0.967198000
C	3.896498000	-2.382644000	-1.550894000
Ir	0.735452000	0.182991000	0.281035000
P	-0.671375000	-1.418086000	0.688117000
P	-0.901288000	1.635034000	0.538900000
O	-1.667469000	-1.343118000	1.999507000
C	-1.078196000	-1.044581000	3.265081000
O	0.096688000	-2.858621000	0.888516000
C	-0.658754000	-4.053050000	1.090067000
O	-0.440438000	3.088771000	-0.053197000
C	-1.284699000	4.229286000	-0.204168000
O	-1.336745000	1.878894000	2.104430000
C	-2.243158000	2.892978000	2.536046000
H	6.663966000	2.889703000	-1.592917000
H	7.957198000	0.685936000	-0.578993000
H	5.948533000	-0.963520000	0.219033000
H	2.113462000	-2.673938000	-0.454294000
H	3.868931000	-3.750695000	0.868326000
H	2.991177000	-2.536634000	1.811211000
H	4.615632000	-2.186712000	1.214878000
H	3.440926000	-1.924581000	-2.433171000
H	3.958012000	-3.459976000	-1.730856000
H	4.918052000	-2.009857000	-1.470190000
H	-0.657909000	-1.949825000	3.718267000
H	-0.291980000	-0.289015000	3.167587000
H	-1.869837000	-0.655827000	3.907759000
H	-1.192804000	-4.342707000	0.178848000
H	0.053144000	-4.837791000	1.348687000
H	-1.379325000	-3.940582000	1.906731000
H	-2.340430000	3.960482000	-0.306083000
H	-0.969554000	4.749598000	-1.110890000
H	-1.164461000	4.903804000	0.648279000
H	-3.019191000	3.110937000	1.795943000
H	-1.698173000	3.812931000	2.768012000
H	-2.724175000	2.531920000	3.447943000
C	-3.334416000	-2.142520000	-0.273370000
C	-4.020904000	-2.107089000	-1.519625000
C	-3.097164000	-1.703501000	-2.527539000
C	-1.834124000	-1.484727000	-1.910433000
C	-1.973631000	-1.753428000	-0.507162000
C	-2.532628000	1.414236000	-0.242273000

C	-2.807804000	1.595372000	-1.638136000	H	-3.327865000	-1.543761000	-3.572279000
C	-4.182589000	1.301462000	-1.864729000	H	-0.930341000	-1.128441000	-2.388646000
C	-4.766557000	0.926651000	-0.619150000	H	-2.080120000	1.882685000	-2.386903000
C	-3.753780000	0.989127000	0.381278000	H	-4.684008000	1.314468000	-2.823321000
Fe	-3.223726000	-0.295772000	-1.073064000	H	-5.788216000	0.603822000	-0.468273000
H	-3.762838000	-2.366385000	0.693721000	H	-3.862315000	0.725567000	1.425698000
H	-5.074475000	-2.305021000	-1.667731000				

IntE

Gibbs Energy (298.15 K): −118.251231

C	0.001872000	0.453854000	-0.008188000	H	2.045169000	0.366602000	-0.551524000
C	1.270439000	-0.194389000	-0.025299000	H	1.246855000	-1.260482000	-0.243120000
C	-1.266443000	-0.199104000	0.019386000	H	-1.669264000	0.027350000	-0.991517000
H	-0.001533000	1.547386000	0.003151000	H	-1.982795000	0.312998000	0.669695000
H	1.559497000	-0.078031000	1.046077000	H	-1.233136000	-1.277990000	0.151845000

IntB’

Gibbs Energy (298.15 K): −3531.503065

C	3.969261000	3.770847000	-1.090495000	O	-2.341893000	-2.205552000	1.286185000
C	5.288653000	3.436724000	-1.030053000	C	-1.657936000	-2.610879000	2.473611000
C	5.326382000	2.080433000	-0.630571000	O	-0.904765000	-3.161746000	-0.629665000
C	4.023376000	1.677157000	-0.473085000	C	-1.842954000	-4.196612000	-0.953569000
O	3.191812000	2.727399000	-0.750208000	O	-0.178726000	2.447979000	-0.477013000
C	3.328325000	0.494391000	0.030952000	C	-0.017454000	3.834859000	-0.169424000
N	3.910000000	-0.727194000	0.019777000	O	-0.356423000	1.975575000	2.080650000
O	2.187567000	0.696202000	0.525898000	C	-1.087814000	3.007649000	2.760092000
C	5.043089000	-1.049368000	-0.888689000	H	3.447420000	4.677236000	-1.355633000
C	3.239541000	-1.761962000	0.845924000	H	6.126919000	4.081594000	-1.243005000
C	4.726095000	-2.230385000	-1.793095000	H	6.210508000	1.485887000	-0.461369000
C	6.376052000	-1.211630000	-0.166554000	H	5.118090000	-0.177541000	-1.539195000
C	2.129300000	-2.493733000	0.094976000	H	5.526884000	-2.329326000	-2.530316000
Ir	0.331379000	-0.497531000	0.300434000	H	3.790325000	-2.081015000	-2.336302000
P	-1.439296000	-1.744308000	-0.004310000	H	4.669667000	-3.177701000	-1.253215000
P	-0.731548000	1.376505000	0.615462000	H	6.499427000	-0.471911000	0.627874000

H	7.191208000	-1.075150000	-0.882643000	C	-2.510951000	1.595661000	0.503292000
H	6.496751000	-2.202221000	0.274082000	C	-3.204898000	2.430611000	-0.439514000
H	2.480728000	-3.334963000	-0.507896000	C	-4.599899000	2.281756000	-0.210375000
H	1.397193000	-2.893539000	0.805170000	C	-4.782298000	1.364688000	0.863261000
H	-1.132281000	-3.560488000	2.323972000	C	-3.502081000	0.936792000	1.309300000
H	-0.938857000	-1.846316000	2.790645000	Fe	-3.722541000	0.495875000	-0.640352000
H	-2.415505000	-2.743020000	3.246038000	H	-4.629105000	-2.053704000	-0.035530000
H	-2.478393000	-3.898994000	-1.793223000	H	-5.994603000	-0.610091000	-1.860771000
H	-1.258111000	-5.070383000	-1.239029000	H	-4.212997000	0.706423000	-3.390405000
H	-2.471319000	-4.447597000	-0.093787000	H	-1.734882000	0.083786000	-2.515463000
H	-0.967325000	4.302214000	0.113362000	H	-2.739536000	3.023172000	-1.215484000
H	0.350674000	4.311404000	-1.077693000	H	-5.386868000	2.746178000	-0.788984000
H	0.715478000	3.973900000	0.629968000	H	-5.731972000	1.009983000	1.240088000
H	-1.870822000	3.449530000	2.139413000	H	-3.300751000	0.210017000	2.086294000
H	-0.380471000	3.784757000	3.054975000	H	1.625284000	-1.852351000	-0.668707000
H	-1.546582000	2.578287000	3.653497000	C	4.195027000	-2.728014000	1.520025000
C	-4.204759000	-1.452219000	-0.827108000	H	4.956394000	-2.203916000	2.099817000
C	-4.918058000	-0.688703000	-1.790375000	H	3.617625000	-3.345859000	2.212476000
C	-3.975953000	0.007924000	-2.599556000	H	4.688052000	-3.405415000	0.820774000
C	-2.669793000	-0.316243000	-2.143266000	H	2.758176000	-1.186878000	1.642814000
C	-2.802654000	-1.224792000	-1.037261000				

$TS_{B'C'}$

Gibbs Energy (298.15 K): −3531.493381

C	4.101765000	3.696425000	-0.493132000	C	5.965786000	-1.607752000	-1.118914000
C	5.340003000	3.232166000	-0.823937000	C	1.827352000	-2.417084000	0.375468000
C	5.286442000	1.830304000	-0.649183000	Ir	0.473148000	-0.703358000	0.601187000
C	4.014708000	1.532785000	-0.225189000	P	-1.356603000	-1.838818000	0.210458000
O	3.292084000	2.688239000	-0.122591000	P	-0.575216000	1.345017000	0.750439000
C	3.321434000	0.336916000	0.234006000	O	-2.391546000	-2.017370000	1.466273000
N	3.779139000	-0.896074000	-0.074669000	C	-1.844750000	-2.202355000	2.774168000
O	2.322150000	0.501042000	0.989346000	O	-0.974454000	-3.365075000	-0.220524000
C	4.526908000	-1.157463000	-1.334453000	C	-2.012567000	-4.315780000	-0.494569000
C	3.230970000	-1.981584000	0.780658000	O	0.198486000	2.316220000	-0.302062000
C	3.752746000	-2.088251000	-2.256331000	C	0.234936000	3.736001000	-0.133747000

O	-0.408458000	1.971545000	2.246091000	C	-3.981034000	-1.408789000	-0.935200000
C	-1.078976000	3.142621000	2.736457000	C	-4.526430000	-0.701224000	-2.040585000
H	3.661569000	4.681429000	-0.473872000	C	-3.453446000	-0.133958000	-2.785166000
H	6.183385000	3.821822000	-1.147870000	C	-2.232699000	-0.483892000	-2.147164000
H	6.092186000	1.129283000	-0.802960000	C	-2.552145000	-1.278438000	-0.993342000
H	4.552945000	-0.187041000	-1.831518000	C	-2.300912000	1.678768000	0.348886000
H	4.262065000	-2.131579000	-3.222538000	C	-2.794883000	2.438320000	-0.766434000
H	2.737567000	-1.721661000	-2.428902000	C	-4.215322000	2.381937000	-0.741002000
H	3.692004000	-3.109885000	-1.876805000	C	-4.611428000	1.592777000	0.375892000
H	6.448959000	-1.051376000	-0.312357000	C	-3.439565000	1.153889000	1.050546000
H	6.535876000	-1.432490000	-2.035469000	Fe	-3.404658000	0.521209000	-0.860460000
H	6.043790000	-2.671336000	-0.889637000	H	-4.533604000	-1.911533000	-0.153576000
H	1.836333000	-3.106983000	-0.474148000	H	-5.580212000	-0.570606000	-2.247046000
H	1.359758000	-2.977117000	1.195962000	H	-3.551319000	0.501692000	-3.654560000
H	-1.260708000	-3.126511000	2.839078000	H	-1.236811000	-0.175086000	-2.439549000
H	-1.211934000	-1.352947000	3.057356000	H	-2.185134000	2.921107000	-1.518375000
H	-2.687780000	-2.265627000	3.461824000	H	-4.877827000	2.818287000	-1.476216000
H	-2.565022000	-4.043245000	-1.398564000	H	-5.626787000	1.325654000	0.635690000
H	-1.521154000	-5.275401000	-0.649746000	H	-3.403480000	0.506863000	1.917931000
H	-2.705814000	-4.396938000	0.347634000	H	1.155523000	-1.458389000	-0.644201000
H	-0.759883000	4.155133000	0.052396000	C	4.172262000	-3.163801000	0.935401000
H	0.623829000	4.151274000	-1.063739000	H	5.159791000	-2.851539000	1.281170000
H	0.905530000	4.006288000	0.686372000	H	3.753216000	-3.830919000	1.692568000
H	-1.887490000	3.465317000	2.075562000	H	4.287619000	-3.749012000	0.021676000
H	-0.354600000	3.951942000	2.848969000	H	3.147644000	-1.513246000	1.766133000
H	-1.498852000	2.897634000	3.713451000				

IntC’

Gibbs Energy (298.15 K): −3531.505728

C	3.972307000	3.805079000	-0.763978000	N	3.673145000	-0.756095000	-0.072710000
C	5.207635000	3.327212000	-1.086585000	O	2.245601000	0.701294000	0.948531000
C	5.163486000	1.937259000	-0.830247000	C	4.359486000	-1.085705000	-1.351800000
C	3.899877000	1.660466000	-0.370455000	C	3.178168000	-1.787740000	0.885335000
O	3.173154000	2.816981000	-0.323216000	C	3.538311000	-2.055395000	-2.189643000
C	3.225143000	0.490099000	0.174214000	C	5.806997000	-1.531912000	-1.190540000

C	1.738312000	-2.228954000	0.622996000
Ir	0.436993000	-0.562079000	0.602426000
P	-1.313852000	-1.827487000	0.144987000
P	-0.694471000	1.513462000	0.663308000
O	-2.325198000	-2.164198000	1.384814000
C	-1.752906000	-2.643063000	2.605974000
O	-0.855977000	-3.287055000	-0.407993000
C	-1.845225000	-4.256988000	-0.783088000
O	-0.000870000	2.508373000	-0.422599000
C	-0.025890000	3.930398000	-0.282302000
O	-0.511944000	2.165068000	2.148750000
C	-1.224574000	3.307223000	2.646327000
H	3.527423000	4.787846000	-0.793707000
H	6.042940000	3.901166000	-1.456462000
H	5.969081000	1.229710000	-0.953176000
H	4.366163000	-0.140808000	-1.897026000
H	4.007585000	-2.157199000	-3.171955000
H	2.520175000	-1.687971000	-2.339615000
H	3.481592000	-3.053631000	-1.751837000
H	6.329153000	-0.950678000	-0.426968000
H	6.331999000	-1.386266000	-2.138765000
H	5.896566000	-2.587765000	-0.932101000
H	1.670741000	-2.854190000	-0.271552000
H	1.442847000	-2.875536000	1.464107000
H	-1.231372000	-3.593576000	2.456672000
H	-1.058463000	-1.909572000	3.031249000
H	-2.576662000	-2.793332000	3.302341000
H	-2.392291000	-3.933694000	-1.673268000
H	-1.305526000	-5.176172000	-1.007056000
H	-2.550158000	-4.441886000	0.032677000
H	-1.038605000	4.311104000	-0.110418000
H	0.354517000	4.345159000	-1.216160000
H	0.626197000	4.244823000	0.537424000
H	-2.031500000	3.617964000	1.977609000
H	-0.523778000	4.134071000	2.779227000
H	-1.651976000	3.039244000	3.614369000
C	-3.953577000	-1.448293000	-0.976052000
C	-4.528721000	-0.747035000	-2.070168000
C	-3.483619000	-0.102254000	-2.790237000
C	-2.250408000	-0.396222000	-2.147763000
C	-2.533823000	-1.235601000	-1.016099000
C	-2.453589000	1.755325000	0.333680000
C	-3.055293000	2.474355000	-0.753938000
C	-4.463334000	2.286289000	-0.679841000
C	-4.745081000	1.453600000	0.439780000
C	-3.513820000	1.119446000	1.066776000
Fe	-3.490019000	0.510746000	-0.852028000
H	-4.482787000	-2.004335000	-0.214629000
H	-5.586469000	-0.671114000	-2.283368000
H	-3.609433000	0.548557000	-3.644677000
H	-1.273315000	-0.022256000	-2.427057000
H	-2.523109000	3.017571000	-1.523431000
H	-5.188651000	2.665732000	-1.386953000
H	-5.721182000	1.089739000	0.730864000
H	-3.387532000	0.466023000	1.921185000
H	0.603726000	-0.406180000	-0.928764000
C	4.136090000	-2.960158000	1.027032000
H	5.150634000	-2.637929000	1.272054000
H	3.781690000	-3.584160000	1.850906000
H	4.173496000	-3.595385000	0.140242000
H	3.187278000	-1.259636000	1.845931000

TS$_{C'F}$

Gibbs Energy (298.15 K): −3531.495204

C	5.683703000	2.198651000	-1.470677000
C	6.485064000	1.643431000	-0.519741000

C	5.794834000	0.501306000	-0.047826000
C	4.616185000	0.440996000	-0.746355000
O	4.544601000	1.493117000	-1.611315000
C	3.393394000	-0.350153000	-0.654759000
N	3.402658000	-1.604644000	-0.148917000
O	2.330067000	0.224863000	-1.003970000
C	4.641002000	-2.448183000	-0.175965000
C	2.095900000	-2.289625000	-0.076332000
C	4.514966000	-3.606985000	-1.152751000
C	5.103105000	-2.863622000	1.210859000
C	1.451187000	-2.479750000	-1.356905000
C	1.960176000	-3.349840000	0.988322000
Ir	0.456284000	-0.781263000	-0.486267000
P	-1.418250000	-1.850502000	0.039477000
P	-0.405212000	1.153541000	0.454820000
O	-1.931641000	-1.731074000	1.580312000
C	-1.045703000	-2.041066000	2.661189000
O	-1.253196000	-3.447223000	-0.221968000
C	-2.370959000	-4.323688000	0.001110000
O	0.539866000	2.403894000	0.057740000
C	0.166344000	3.787118000	0.154184000
O	-0.498582000	1.086829000	2.077466000
C	-0.742576000	2.253212000	2.877578000
H	5.783395000	3.056489000	-2.117026000
H	7.447592000	2.007634000	-0.196402000
H	6.116393000	-0.175273000	0.729422000
H	5.398653000	-1.785700000	-0.590930000
H	5.477116000	-4.122486000	-1.202569000
H	4.273688000	-3.253928000	-2.157669000
H	3.766805000	-4.345748000	-0.861291000
H	5.078784000	-2.021642000	1.907026000
H	6.135214000	-3.218639000	1.149814000
H	4.507582000	-3.673821000	1.633185000
H	2.011922000	-2.197969000	-2.242598000
H	0.816280000	-3.351725000	-1.467214000
H	0.915072000	-3.658930000	1.030874000
H	2.544632000	-4.238600000	0.742550000
H	2.259554000	-2.990874000	1.974700000
H	-0.965226000	-3.124849000	2.791977000
H	-0.053714000	-1.611252000	2.497606000
H	-1.480857000	-1.601151000	3.557961000
H	-3.133701000	-4.175998000	-0.768016000
H	-1.984117000	-5.339578000	-0.063134000
H	-2.811374000	-4.163669000	0.988945000
H	-0.902444000	3.917942000	0.340444000
H	0.423213000	4.264312000	-0.792713000
H	0.737150000	4.252757000	0.959991000
H	-1.484823000	2.919380000	2.428398000
H	0.190957000	2.799669000	3.035071000
H	-1.117054000	1.902610000	3.839379000
C	-4.219283000	-1.195141000	-0.087977000
C	-5.130057000	-0.675674000	-1.046833000
C	-4.437180000	-0.509159000	-2.280065000
C	-3.089699000	-0.922554000	-2.097710000
C	-2.946229000	-1.350619000	-0.734960000
C	-2.059358000	1.698598000	-0.025342000
C	-2.412978000	2.153250000	-1.339272000
C	-3.791925000	2.502626000	-1.326640000
C	-4.302962000	2.256461000	-0.019313000
C	-3.242420000	1.754720000	0.787015000
Fe	-3.534560000	0.560667000	-0.809147000
H	-4.422049000	-1.402515000	0.952984000
H	-6.160016000	-0.405857000	-0.856254000
H	-4.850668000	-0.094980000	-3.189392000
H	-2.301702000	-0.896452000	-2.837418000
H	-1.742849000	2.201997000	-2.188276000
H	-4.363789000	2.848761000	-2.176874000
H	-5.330280000	2.382745000	0.294663000

H	-3.312805000	1.439968000	1.820002000
H	-0.352480000	-0.278295000	-1.822056000
H	1.413946000	-1.206407000	0.854169000

IntF

Gibbs Energy (298.15 K): −3531.501908

C	5.281117000	3.335162000	-0.910392000
C	6.275951000	2.659126000	-0.272954000
C	5.746048000	1.382153000	0.030336000
C	4.458845000	1.367944000	-0.444623000
O	4.173098000	2.576456000	-1.012219000
C	3.310558000	0.469385000	-0.357586000
N	3.443534000	-0.855803000	-0.170075000
O	2.176013000	1.014379000	-0.425262000
C	4.737704000	-1.567300000	-0.417135000
C	2.214886000	-1.666426000	-0.123800000
C	4.595293000	-2.639945000	-1.484429000
C	5.381893000	-2.069862000	0.865873000
C	1.491248000	-1.734029000	-1.335607000
C	2.238231000	-2.775457000	0.889727000
Ir	0.487358000	-0.283800000	-0.017559000
P	-1.239025000	-1.640284000	0.398906000
P	-0.780084000	1.514763000	0.713231000
O	-1.877171000	-1.640062000	1.889692000
C	-1.056325000	-1.864129000	3.042999000
O	-0.836760000	-3.186936000	0.095225000
C	-1.836376000	-4.217496000	0.188847000
O	0.032969000	2.863824000	0.383001000
C	-0.522959000	4.189264000	0.394521000
O	-1.043105000	1.440558000	2.308929000
C	-1.514115000	2.577338000	3.052322000
H	5.212305000	4.321744000	-1.341069000
H	7.263984000	3.028480000	-0.047050000
H	6.249101000	0.585909000	0.556289000
H	5.385768000	-0.801880000	-0.842546000
H	5.586493000	-3.044589000	-1.702133000
H	4.192816000	-2.228404000	-2.412460000
H	3.964480000	-3.476206000	-1.177165000
H	5.357099000	-1.308882000	1.649681000
H	6.428758000	-2.316876000	0.669602000
H	4.902482000	-2.969957000	1.253196000
H	1.875572000	-1.229329000	-2.214475000
H	0.843241000	-2.582746000	-1.519923000
H	1.252411000	-3.229624000	0.965949000
H	2.932140000	-3.569399000	0.596671000
H	2.534252000	-2.408649000	1.873895000
H	-0.771323000	-2.918604000	3.111349000
H	-0.159855000	-1.239829000	3.017405000
H	-1.661281000	-1.596884000	3.908397000
H	-2.552579000	-4.133767000	-0.632872000
H	-1.307204000	-5.166157000	0.114165000
H	-2.366524000	-4.171010000	1.143860000
H	-1.615555000	4.181357000	0.374341000
H	-0.153617000	4.701474000	-0.494346000
H	-0.176070000	4.713960000	1.286592000
H	-2.244434000	3.168666000	2.492614000
H	-0.668424000	3.208998000	3.334533000
H	-1.987744000	2.188631000	3.953615000
C	-4.095047000	-1.385272000	0.019802000
C	-4.980381000	-1.083994000	-1.050365000
C	-4.208132000	-0.894432000	-2.232269000
C	-2.836231000	-1.072621000	-1.905180000
C	-2.758522000	-1.374581000	-0.503757000
C	-2.421642000	1.794135000	0.031153000
C	-2.693802000	2.105439000	-1.343013000
C	-4.101503000	2.246220000	-1.487650000

C	-4.708079000	2.015666000	-0.219020000	H	-1.951798000	2.194081000	-2.126412000
C	-3.680412000	1.730638000	0.723243000	H	-4.623916000	2.446494000	-2.413203000
Fe	-3.623971000	0.404349000	-0.786976000	H	-5.770298000	2.009111000	-0.015431000
H	-4.361726000	-1.554216000	1.053314000	H	-3.816205000	1.476660000	1.766284000
H	-6.052825000	-0.969572000	-0.968412000	H	-0.219820000	0.094736000	-1.485560000
H	-4.593075000	-0.615007000	-3.203476000	H	1.035786000	-0.314246000	1.550938000
H	-1.993726000	-0.968840000	-2.575442000				

IntG

Gibbs Energy (298.15 K): −3531.494791

C	-2.396698000	-2.899669000	-3.190621000	H	-1.497541000	-1.586438000	-4.690277000
C	-1.935122000	-1.729085000	-3.714212000	H	-1.911236000	0.296821000	-2.790454000
C	-2.154608000	-0.749621000	-2.718237000	H	-3.110990000	-0.674320000	2.136566000
C	-2.735850000	-1.385906000	-1.649538000	H	-5.238643000	0.313201000	2.870523000
O	-2.886660000	-2.712014000	-1.954154000	H	-5.436904000	-0.421019000	1.279803000
C	-3.184108000	-1.082101000	-0.287183000	H	-5.312407000	1.342329000	1.445527000
N	-2.961957000	0.232376000	0.274948000	H	-1.642040000	1.287229000	2.472327000
O	-3.663636000	-1.964259000	0.395414000	H	-3.023176000	1.320860000	3.551323000
C	-3.428446000	0.283490000	1.719064000	H	-3.004372000	2.397500000	2.156688000
C	-3.323550000	1.530081000	-0.505396000	H	-1.833280000	2.142794000	-2.006759000
C	-4.940539000	0.384589000	1.821436000	H	-1.905110000	3.180233000	-0.584133000
C	-2.737805000	1.393407000	2.499467000	H	0.482490000	3.466053000	2.056625000
C	-1.995450000	2.143546000	-0.925262000	H	0.745562000	1.838051000	2.757653000
Ir	-0.727993000	0.813909000	0.134462000	H	1.936910000	3.132838000	3.022046000
P	1.187836000	1.867292000	-0.188648000	H	2.740864000	3.636185000	-2.010228000
P	0.236405000	-1.082755000	1.132463000	H	1.642032000	5.012738000	-1.749550000
O	1.998371000	2.320873000	1.157548000	H	2.603805000	4.380749000	-0.390644000
C	1.234419000	2.708933000	2.305368000	H	0.439232000	-4.009562000	1.216213000
O	0.946489000	3.254062000	-1.002330000	H	-1.012783000	-4.297398000	0.224729000
C	2.059169000	4.112853000	-1.300174000	H	-1.189910000	-3.693592000	1.885469000
O	-0.560915000	-2.329056000	0.445292000	H	1.352474000	-2.799901000	3.070451000
C	-0.570189000	-3.653991000	0.985031000	H	-0.155467000	-2.795450000	4.023003000
O	0.033954000	-1.181120000	2.748410000	H	1.150240000	-1.648406000	4.409913000
C	0.634428000	-2.170572000	3.601991000	C	3.927142000	1.140987000	-0.720419000
H	-2.448115000	-3.909628000	-3.566686000	C	4.624000000	0.221511000	-1.549815000

C	3.673710000	-0.464415000	-2.357541000	H	1.443344000	-0.318353000	-2.462298000
C	2.378150000	0.023751000	-2.036812000	H	1.883924000	-3.139331000	-0.684018000
C	2.527229000	1.025561000	-1.017058000	H	4.568606000	-3.193239000	-0.441452000
C	1.964498000	-1.572154000	0.919733000	H	5.278898000	-1.378688000	1.416914000
C	2.476166000	-2.553924000	0.007051000	H	3.037197000	-0.194600000	2.335731000
C	3.891562000	-2.583920000	0.141809000	H	-0.386017000	0.132665000	-1.206920000
C	4.266867000	-1.624839000	1.125173000	C	-4.407048000	1.365425000	-1.549026000
C	3.086418000	-0.991370000	1.603472000	H	-5.270828000	0.822393000	-1.152720000
Fe	3.253477000	-0.730204000	-0.386264000	H	-4.749268000	2.362951000	-1.838667000
H	4.356207000	1.781499000	0.037553000	H	-4.074400000	0.863614000	-2.456877000
H	5.689282000	0.035303000	-1.527488000	H	-3.752655000	2.149690000	0.283477000
H	3.892306000	-1.259494000	-3.057184000				

TS$_{GH}$

Gibbs Energy (298.15 K): −3531.486081

C	-2.290048000	-2.684772000	-3.468492000	O	-0.561650000	-2.383338000	0.308052000
C	-1.890796000	-1.440450000	-3.847577000	C	-0.544716000	-3.726514000	0.805822000
C	-2.103534000	-0.606825000	-2.718973000	O	0.123179000	-1.248639000	2.590077000
C	-2.618057000	-1.398367000	-1.724168000	C	0.732522000	-2.249876000	3.425677000
O	-2.739046000	-2.674964000	-2.197796000	H	-2.320369000	-3.640136000	-3.968501000
C	-3.082007000	-1.267296000	-0.324066000	H	-1.500250000	-1.153711000	-4.812147000
N	-2.749168000	-0.126815000	0.393050000	H	-1.895839000	0.449247000	-2.660530000
O	-3.725200000	-2.193368000	0.161956000	H	-2.945733000	-1.197431000	2.167045000
C	-3.222132000	-0.195765000	1.808510000	H	-5.040494000	-0.240095000	2.963709000
C	-3.261780000	1.828706000	-0.373527000	H	-5.257382000	-0.767063000	1.293762000
C	-4.734584000	-0.054432000	1.929882000	H	-5.068853000	0.956567000	1.675373000
C	-2.528640000	0.826745000	2.698554000	H	-1.432160000	0.757135000	2.638305000
C	-1.985101000	2.379250000	-0.663111000	H	-2.784052000	0.639551000	3.744605000
Ir	-0.699597000	0.752143000	0.160785000	H	-2.828486000	1.856632000	2.476806000
P	1.231664000	1.873147000	-0.021146000	H	-1.758208000	2.552472000	-1.714722000
P	0.269858000	-1.163886000	0.971789000	H	-1.703769000	3.216435000	-0.020840000
O	2.053442000	2.136801000	1.366351000	H	0.547058000	3.175147000	2.403136000
C	1.306935000	2.401442000	2.559833000	H	0.831430000	1.486136000	2.928546000
O	0.981928000	3.355125000	-0.641881000	H	2.019652000	2.756504000	3.303305000
C	2.095840000	4.229851000	-0.886886000	H	2.736461000	3.830125000	-1.677885000

H	1.675583000	5.181266000	-1.208951000	C	3.137028000	-1.174496000	1.391138000
H	2.684317000	4.381912000	0.021859000	Fe	3.261767000	-0.687349000	-0.556950000
H	0.476512000	-4.085504000	0.969510000	H	4.410763000	1.735470000	0.155803000
H	-1.028689000	-4.342214000	0.048582000	H	5.701889000	0.170613000	-1.620634000
H	-1.117942000	-3.791920000	1.734785000	H	3.871979000	-0.900720000	-3.279285000
H	1.400469000	-2.907738000	2.865295000	H	1.444734000	0.002892000	-2.554766000
H	-0.059394000	-2.840878000	3.888821000	H	1.807887000	-3.010379000	-1.093179000
H	1.302964000	-1.737035000	4.202052000	H	4.496705000	-3.167456000	-0.935134000
C	3.964759000	1.199575000	-0.670555000	H	5.309317000	-1.603261000	1.099838000
C	4.639884000	0.375414000	-1.610722000	H	3.131166000	-0.467382000	2.211243000
C	3.672501000	-0.192953000	-2.486405000	H	-0.310582000	0.197559000	-1.225150000
C	2.387994000	0.273086000	-2.097197000	C	-4.299672000	1.529039000	-1.380301000
C	2.561215000	1.142299000	-0.964604000	H	-5.038258000	0.808277000	-1.025522000
C	1.981318000	-1.643011000	0.676936000	H	-4.830956000	2.476084000	-1.552407000
C	2.437925000	-2.524572000	-0.359316000	H	-3.899219000	1.210697000	-2.341571000
C	3.853725000	-2.609636000	-0.268045000	H	-3.656018000	2.035826000	0.616932000
C	4.283213000	-1.783264000	0.809181000				

IntH

Gibbs Energy (298.15 K): −3531.499826

C	-1.303064000	-3.825004000	-2.193477000	P	0.032631000	-0.705361000	1.353629000
C	-1.083183000	-2.795478000	-3.053576000	O	1.897559000	2.147141000	1.319920000
C	-1.661418000	-1.648808000	-2.441526000	C	1.173736000	2.884721000	2.312765000
C	-2.190094000	-2.056096000	-1.245749000	O	1.117411000	3.345760000	-0.818629000
O	-1.982921000	-3.397853000	-1.102458000	C	2.325159000	4.110597000	-0.979261000
C	-3.013130000	-1.440599000	-0.168932000	O	-1.002865000	-1.902736000	1.609295000
N	-2.760753000	-0.139070000	0.133808000	C	-0.702156000	-3.206715000	2.136854000
O	-3.881041000	-2.133722000	0.368790000	O	0.325591000	0.004213000	2.779195000
C	-3.592151000	0.373954000	1.247107000	C	0.545125000	-0.768183000	3.973856000
C	-2.831364000	2.298655000	-1.672132000	H	-1.055752000	-4.874914000	-2.208473000
C	-5.028060000	0.632458000	0.810779000	H	-0.582577000	-2.851804000	-4.008574000
C	-2.979191000	1.627865000	1.861339000	H	-1.700999000	-0.649040000	-2.844635000
C	-1.604850000	2.849951000	-1.628280000	H	-3.613530000	-0.412794000	2.016385000
Ir	-0.851276000	0.794939000	-0.036091000	H	-5.636668000	0.948071000	1.664019000
P	1.175112000	1.878590000	-0.118812000	H	-5.473647000	-0.271570000	0.395306000

H	-5.081310000	1.423293000	0.056062000
H	-1.928498000	1.472846000	2.161920000
H	-3.510595000	1.910470000	2.773622000
H	-3.026391000	2.489867000	1.187075000
H	-0.897764000	2.729107000	-2.444346000
H	-1.337452000	3.565448000	-0.858451000
H	1.183921000	3.954271000	2.080961000
H	0.140153000	2.534071000	2.396305000
H	1.676867000	2.714845000	3.263806000
H	2.962632000	3.664319000	-1.747073000
H	2.018908000	5.106423000	-1.296039000
H	2.878690000	4.182496000	-0.039245000
H	0.348640000	-3.472659000	1.999866000
H	-1.332589000	-3.910850000	1.594991000
H	-0.954726000	-3.231663000	3.198361000
H	1.172782000	-1.643102000	3.784199000
H	-0.412372000	-1.081418000	4.395809000
H	1.052840000	-0.109386000	4.677777000
C	3.881059000	0.921196000	-0.438291000
C	4.566899000	0.004953000	-1.280134000
C	3.652679000	-0.478893000	-2.258768000
C	2.389240000	0.132451000	-2.032744000
C	2.523752000	1.002710000	-0.896664000
C	1.568682000	-1.510973000	0.909287000
C	1.714063000	-2.444077000	-0.172906000
C	3.063006000	-2.893268000	-0.177104000
C	3.757378000	-2.240653000	0.882070000
C	2.845914000	-1.380229000	1.555329000
Fe	2.967284000	-0.877773000	-0.389455000
H	4.282226000	1.431923000	0.425742000
H	5.595156000	-0.309577000	-1.163907000
H	3.867973000	-1.218727000	-3.017293000
H	1.482985000	-0.044940000	-2.597699000
H	0.932701000	-2.740814000	-0.863009000
H	3.497294000	-3.577143000	-0.893376000
H	4.810526000	-2.341474000	1.106489000
H	3.069734000	-0.719361000	2.382654000
H	-0.273312000	-0.088106000	-1.162027000
C	-3.379601000	1.458359000	-2.757805000
H	-3.738355000	0.501580000	-2.362549000
H	-4.253861000	1.953248000	-3.195284000
H	-2.655479000	1.276261000	-3.554652000
H	-3.520584000	2.521863000	-0.859874000

IntI

Gibbs Energy (298.15 K): −3413.648616

C	6.409616000	-1.998173000	-0.728079000
C	6.980385000	-0.777360000	-0.534022000
C	5.909077000	0.127646000	-0.342470000
C	4.750917000	-0.605747000	-0.435454000
O	5.067567000	-1.916447000	-0.667497000
C	3.326946000	-0.348232000	-0.312249000
N	2.715217000	0.819448000	-0.232072000
O	2.529445000	-1.370587000	-0.277584000
C	3.383076000	2.117045000	-0.154051000
C	3.820118000	2.407088000	1.276946000
C	2.469920000	3.210092000	-0.670348000
Ir	0.785126000	-0.118871000	0.056239000
P	-0.747604000	-1.730388000	0.422283000
P	-0.723480000	1.559331000	0.112641000
O	-1.373355000	-1.789895000	1.927869000
C	-0.502346000	-1.530544000	3.034976000
O	0.010790000	-3.144274000	0.217497000
C	-0.701305000	-4.377978000	0.414687000
O	-0.519701000	2.398790000	-1.257632000
C	-0.931174000	3.766758000	-1.397557000

O	-0.408604000	2.508818000	1.390625000
C	-1.240683000	3.594768000	1.840453000
H	6.809608000	-2.982467000	-0.914961000
H	8.035891000	-0.554364000	-0.525574000
H	5.996215000	1.183332000	-0.144619000
H	4.264351000	2.078718000	-0.807745000
H	4.374174000	3.348458000	1.332907000
H	4.456936000	1.615011000	1.679548000
H	2.940958000	2.497837000	1.923824000
H	2.097106000	2.985118000	-1.671453000
H	3.006358000	4.161885000	-0.704685000
H	1.612520000	3.334957000	-0.003018000
H	0.284747000	-2.287298000	3.108680000
H	-0.046290000	-0.535606000	2.952426000
H	-1.116831000	-1.562951000	3.933445000
H	-1.491729000	-4.494028000	-0.332115000
H	0.029582000	-5.175667000	0.295171000
H	-1.132637000	-4.426491000	1.418409000
H	-1.916118000	3.948892000	-0.958473000
H	-0.974243000	3.970310000	-2.467117000
H	-0.193667000	4.430009000	-0.937450000
H	-2.259587000	3.516199000	1.454073000
H	-0.796917000	4.541004000	1.524234000
H	-1.266099000	3.551207000	2.929366000
C	-3.558163000	-2.218072000	-0.015116000
C	-4.506184000	-1.940812000	-1.035892000
C	-3.822531000	-1.364097000	-2.143033000
C	-2.442253000	-1.274997000	-1.820626000
C	-2.269383000	-1.805814000	-0.495598000
C	-2.511256000	1.365001000	0.152224000
C	-3.425390000	1.705830000	-0.906126000
C	-4.737145000	1.372263000	-0.474509000
C	-4.651759000	0.821685000	0.835047000
C	-3.286437000	0.810307000	1.228701000
Fe	-3.513454000	-0.253208000	-0.465145000
H	-3.764017000	-2.617752000	0.968064000
H	-5.574274000	-2.092991000	-0.960307000
H	-4.281519000	-1.002328000	-3.052800000
H	-1.660474000	-0.846831000	-2.435182000
H	-3.151581000	2.102172000	-1.874376000
H	-5.637726000	1.468039000	-1.065524000
H	-5.476599000	0.425898000	1.411617000
H	-2.889522000	0.422298000	2.157881000
H	0.442780000	-0.267593000	-1.443877000

IntJ

Gibbs Energy (298.15 K): −117.866019

C	-0.129057000	0.452833000	0.000013000
C	-1.277414000	-0.218323000	0.000005000
C	1.225171000	-0.163409000	0.000007000
H	-0.158856000	1.542949000	-0.000100000
H	-1.300653000	-1.305877000	-0.000100000

Control experiments to probe the possibility of "hidden" acid promoted N-dealkylation

General procedure E: probing the effects of additivea on N-dealkylation in 1,2-DCB.

To a flame-dried resealable Schlenk tube under a nitrogen atmosphere was added the specified (hetero)aromatic amide (0.1 mmol), additive (10 mol%) and solvent (1 M). The reaction was sealed under an atmosphere of N_2 and transferred to a heating block pre-heated at 120 °C and stirred for 72 h. The reaction was cooled to r.t. and concentrated *in vacuo*. The reaction crude was analyzed by ^{1}H NMR using trichloroethylene as an internal standard and purified by FCC under the conditions noted.

General procedure F: neat acid promoted N-dealkylation.

Adapted from a procedure by Reeves et al.[27] To a flame-dried resealable Schlenk tube under a nitrogen atmosphere was added the specified (hetero)aromatic amide (0.1 mmol) and a methane sulfonic acid (10 volumes). The reaction was sealed under an atmosphere of N_2 and transferred to a heating block pre-heated at 90 °C and stirred for 4 h. The reaction was cooled to r.t., diluted with DCM, washed sat. aq. with $NaHCO_3$, dried over Na_2SO_4 and concentrated *in vacuo*. The reaction crude was analyzed by ^{1}H NMR using trichloroethylene as an internal standard and purified by FCC under the conditions noted.

Probing acid promoted N-dealkylation of 1a:

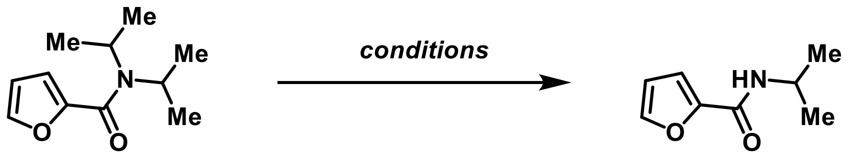

1a **NH-1a**

General procedure E: 1a (19.5 mg, 0.1 mmol), TfOH (0.1 mL of a 0.1M solution in 1,2-DCB). ^{1}H NMR analysis of crude reaction showed only **1a** in a 99% analytical yield.

General procedure F: 1a (19.5 mg, 0.1 mmol), MsOH (200 µL). ^{1}H NMR analysis of crude reaction showed **1a** (46%) and **NH-1a** (51%). Purification was carried out by FCC (0-25% EtOAc:Pet. Ether), and the data was in accordance with that previously reported in this document.

These results show that a highly acidic conditions are required for N-dealkylation. Under the catalysis conditions, catalytic quantities of TfOH do not promote N-dealkylation.

Probing acid promoted N-dealkylation of *deuterio*-1a:

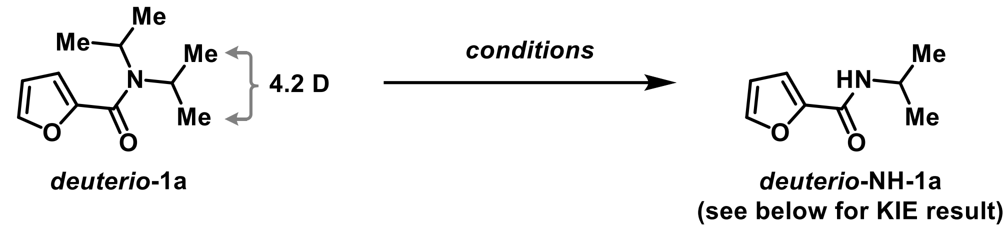

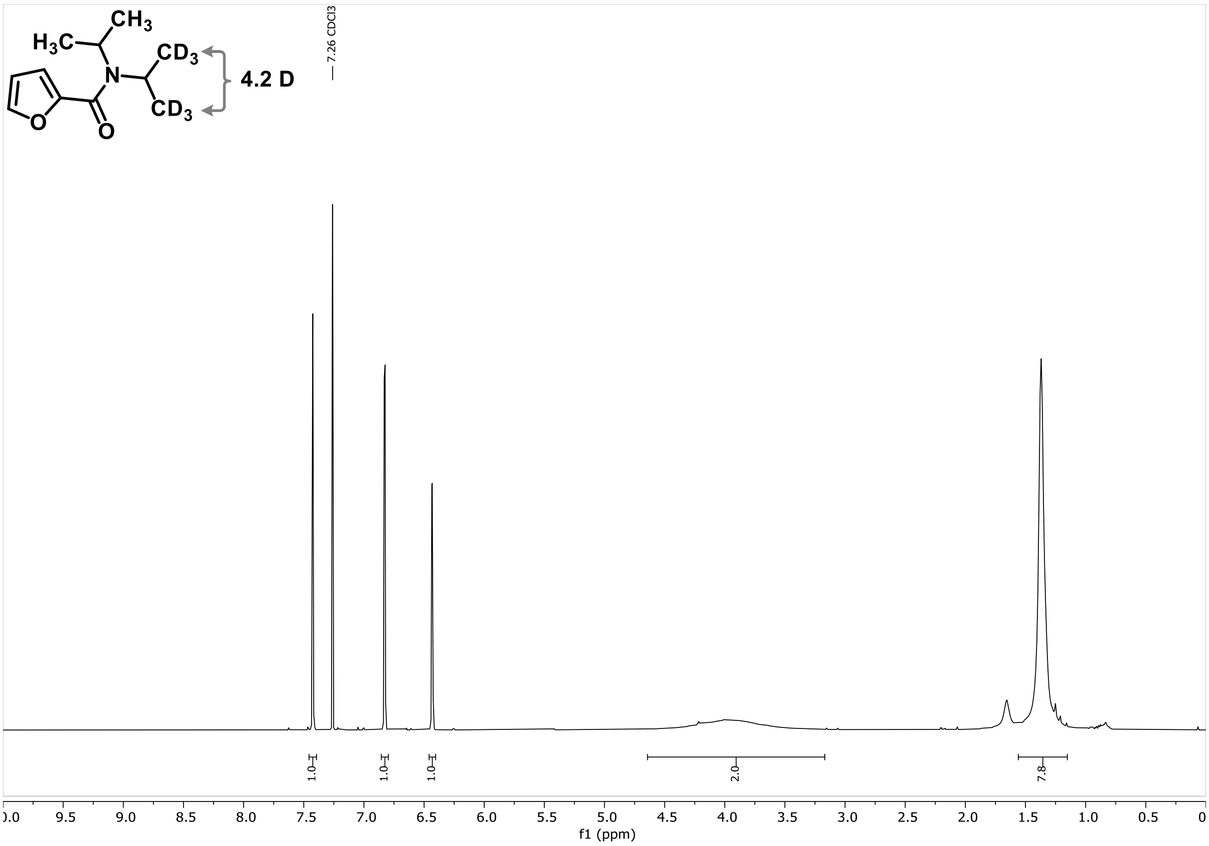

General procedure E: *deuterio*-1a (20.1 mg, 0.1 mmol), NaBArF (8.9 mg, 0.01 mmol) and 1,2-DCB (0.1 mL). ^{1}H NMR analysis of crude reaction showed only ***deuterio*-1a (4.2D)** in 91% analytical yield.

General procedure E: *deuterio*-1a (20.1 mg, 0.1 mmol), MsOH (0.1 mL of a 0.1M solution in 1,2-DCB). ^{1}H NMR analysis of crude reaction showed only ***deuterio*-1a (4.2D)** in 92% analytical yield.

General procedure F: *deuterio*-1a (4.2D) (20.1 mg, 0.1 mmol), MsOH (200 µL). ^{1}H NMR analysis of crude reaction showed ***deuterio*-1a (4.2D)** (41%) and ***deuterio*-NH-1a (2.1D)** (50%). Purification was carried out by FCC (0-25% EtOAc:Pet. Ether), data was in accordance with that previously reported in this document.

These results show that a highly acidic conditions are required for N-dealkylation. Under the catalysis conditions, catalytic quantities of MsOH or NaBARF do not promote N-dealkylation. The results from General Procedure F give an acid promoted secondary KIE of ~1, which is inconsistent with the isotope effect observed under catalytic conditions (~2; see Scheme 3A).

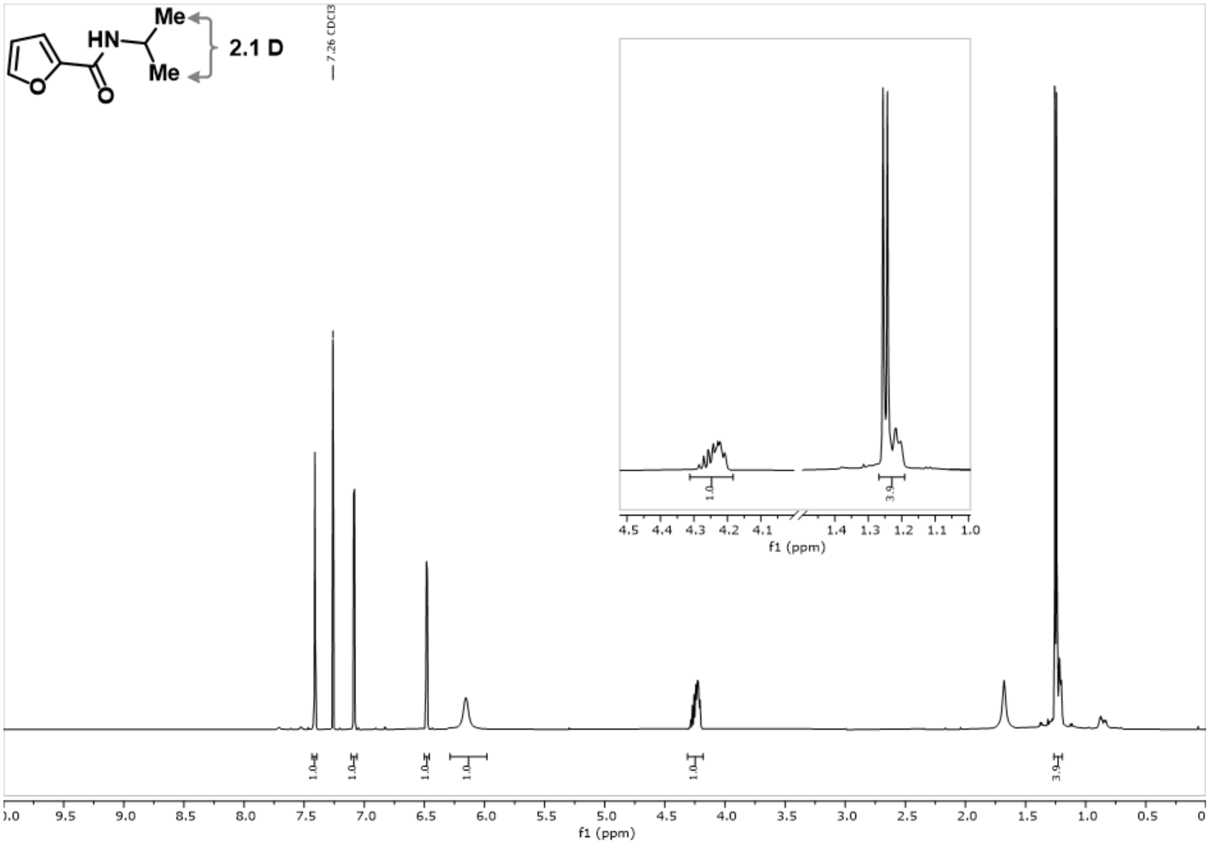

Probing acid promoted N-dealkylation of 1d

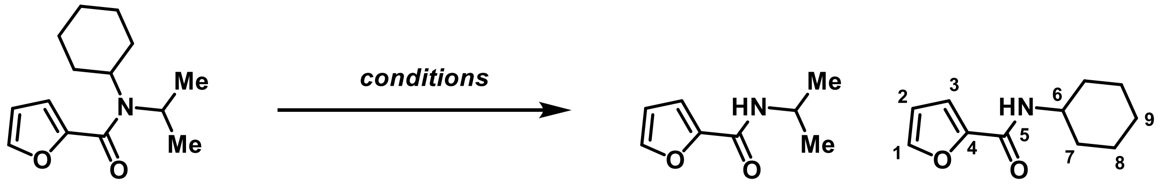

1d **NH-1a** **NH-1w**

General procedure E: 1d (23.5 mg, 0.1 mmol), NaBArF (8.9 mg, 0.01 mmol) and 1,2-DCB (0.1 mL). ^{1}H NMR analysis of crude reaction showed only **1d** in 97% analytical yield.

General procedure E: 1d (23.5 mg, 0.1 mmol), MsOH (0.1 mL of a 0.1M solution in 1,2-DCB). ^{1}H NMR analysis of crude reaction showed only **1d** in 99% analytical yield.

General procedure F: 1d (23.5 mg, 0.1 mmol), MsOH (235 μL). ^{1}H NMR analysis of crude reaction showed **1d** (trace), **NH-1a** (73%) and **NH-1w** (17%). Purification was carried out by FCC (0-25% EtOAc:Pet. Ether), and the data for **NH-1a** was in accordance with that previously reported in this document.

NH-1w: ^{1}H NMR (500 MHz, $CDCl_3$): δ 7.41 (1H, dd, *J* = 1.8, 0.8 Hz, C**1**-H), 7.08 (1H, dd, *J* = 3.4, 0.8 Hz, C**3**-H), 6.47 (1H, dd, *J* = 3.5, 1.8 Hz, C**2**-H), 6.22 (1H, br-s, **NH**), 3.98 – 3.87 (1H, m, C**6**-H), 2.04 – 1.94 (2H, m, C**7**-H’), 1.79 – 1.69 (2H, m, C**8**-H’), 1.68 – 1.58 (1H, m, C**9**-H’), 1.47 – 1.34 (2H, m, C**8**-H’’), 1.32 – 1.12 (3H,

m, C**7**-H'' & C**9**-H''). ^{13}C NMR (126 MHz, $CDCl_3$): δ 157.6 (C**5**), 148.4 (C**4**), 143.7 (C**1**), 114.0 (C**3**), 112.2 (C**2**), 48.0 (C**6**), 33.3 (C**7**), 25.6 (C**9**), 25.0 (C**8**).

Data in accordance with literature.[28]

*These results show that a highly acidic conditions are required for N-dealkylation. Under the catalysis conditions, catalytic quantities of MsOH or NaBARF do not promote N-dealkylation. The results from General Procedure F show that the N-cyclohexyl unit dealkylates in preference to the N-isopropyl unit (~4:1 selectivity) which contrasts with the selectivity observed under iridium catalyzed conditions, where only isopropyl cleavage was observed (see **4d** in Table 1A).*

Probing acid promoted N-dealkylation of 1w

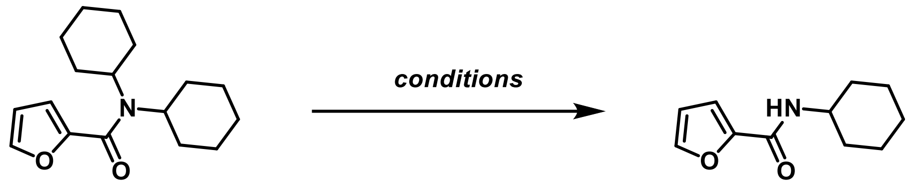

General procedure E: 1w (27.5 mg, 0.1 mmol), NaBArF (8.9 mg, 0.01 mmol) and 1,2-DCB (0.1 mL). ^{1}H NMR Analysis of the crude reaction showed only **1w** in 95% analytical yield.

General procedure E: 1w (27.5 mg, 0.1 mmol), MsOH (0.1 mL of a 0.1M solution in 1,2-DCB). ^{1}H NMR Analysis of the crude reaction showed only **1w** in 99% analytical yield.

General procedure F: 1d (27.5 mg, 0.1 mmol), MsOH (275 µL). ^{1}H NMR Analysis of crude reaction showed **NH-1w** (94%). Purification was carried out by FCC (0-25% EtOAc:Pet. Ether), and data were in accordance with that previously reported in this document.

*These results show that a highly acidic conditions are required for N-dealkylation. Under the catalysis conditions, catalytic quantities of MsOH or NaBARF do not promote N-dealkylation. The results from General Procedure F show that the N-cyclohexyl unit dealkylates faster than the N-isopropyl unit of **1a** (~ 2 × faster, see above). This contrasts the results in the main paper where **1a** reacts but **1w** is recovered intact.*

References

1. Race, N. J.; Faulkner, A.; Fumagalli, G.; Yamauchi, T.; Scott, J. S.; Rydén-Landergren, M.; Sparkes, H. A.; Bower, J. F. *Chem. Sci.* **2017**, *8*, 1981–1985.

2. Birman, V. B.; Rheingold A. L.; Lam, K-C. *Tetrahedron: Asymmetry* **1999**, *10*, 125–131.

3. Li, Z.; Liang, X.; Wu, F.; Wan, B. *Tetrahedron: Asymmetry* **2004**, *15*, 665–669.

4. Grélaud, S.; Cooper, P.; Feron, L. J.; Bower, J. F. *J. Am. Chem. Soc.* **2018**, *140*, 9351–9356.

5. Schröder, N.; Lied, F.; Glorius, F. *J. Am. Chem. Soc*. **2015**, *137*, 1448–1451.

6. Sasaki, I.; Taguchi, J.; Hiraki, S.; Ito, H.; Ishiyama, T. *Chem. Eur. J.* **2015**, *21*, 9236–9241.

7. Jiang, H.; Jiajun, L.; Xie, Y.; Biaolin, Y.; Bin, Y. *Chin. J. Chem*. **2021**, *39*, 62–68.

8. Bisht, R.; Hoque, M. E.; Chattopadhyay, B. *Angew. Chem. Int. Ed.* **2018**, *57*, 15762-15766.

9. Chen, L.; Chen, H-N.; Xiao, T-F.; Hu, X-Q.; Xu, P-F.; Xu, G-Q. *Chem. Commun*. **2023**, *59*, 2003–2006.

10. Hironori, M.; Hideki, N.; Hideo, S.; *Org. Process Res. Dev.* **2020**, *24*, 2772–2779.

11. Mattia, A.; Fabio, A.; Dalla Torre, D.; Trapasso, G. *Green Chem.* **2022**, *24,* 2766–2771.

12. Henry, C.; Bolien, D.; Ibanescu, B.; Bloodworth, S.; Harrowven, D. C.; Zhang, X.; Craven, A.; Sneddon, H. F.; Whitby, R. J. *Eur. J. Org. Chem.* **2015**, *7*, 1491.

13. Pelagalli, R.; Chiarotto, I.; Feroci, M.; Vecchio, S. *Green. Chem*. **2012**, *14*, 2251–2255.

14. Connell, T. U.; Forni, J. A.; Nenad, M.; Polyzos, A.; Weragoda, G. *Angew. Chem. Int. Ed.* **2020**, *59*, 18646–18654.

15. Chirila, P. G.; Skibinski, L.; Miller, K.; Hamilton, A.; Whiteoak, C. J.; *Adv. Synth. Catal*. **2018**, *12*, 2324–2332

16. Xia, Q.; Liu, X.; Zhang, Y.; Chen, C.; Chen, W. *Org. Lett*. **2013**, *15*, 3326–3329.

17. Mistry, S. N.; Shonberg, J.; Draper-Joyce, C. J.; Klein-Herenbrink, C.; Michino, M.; Lei, S.; Christopoulos, A.; Capuano, B.; Scammels, P. J.; Lane, J. R. *J. Med. Chem.* **2015**, *58*, 6819–6843.

18. Ye, Y-H.; Zhang, J.; Wang, G.; Chen, S-Y.; Yu, X-Q. *Tetrahedron*, **2011**, *67*, 4649–4654.

19. Huanfeng, J.; Jiang, K.; Yingwei, L.; Wenkun, L.; Biaolin, Y. *Org. Lett*. **2020**, *22*, 2093–2098.

20. Guijarro, D.; Oscar, P.; Yus, M. *J. Org. Chem.* **2010**, *75*, 5265-5270.

21. Gaussian 16, Revision A.03, M. J. Frisch, G. W. Trucks, H. B. Schlegel, G. E. Scuseria, M. A. Robb, J. R. Cheeseman, G. Scalmani, V. Barone, G. A. Petersson, H. Nakatsuji, X. Li, M. Caricato, A. V. Marenich, J. Bloino, B. G. Janesko, R. Gomperts, B. Mennucci, H. P. Hratchian, J. V. Ortiz, A. F. Izmaylov, J. L. Sonnenberg, D. Williams-Young, F. Ding, F. Lipparini, F. Egidi, J. Goings, B. Peng, A. Petrone, T. Henderson, D. Ranasinghe, V. G. Zakrzewski, J. Gao, N. Rega, G. Zheng, W. Liang, M. Hada, M. Ehara, K. Toyota, R. Fukuda, J. Hasegawa, M. Ishida, T. Nakajima, Y. Honda, O. Kitao, H. Nakai, T. Vreven, K. Throssell, J. A. Montgomery, Jr., J. E. Peralta, F. Ogliaro, M. J. Bearpark, J. J. Heyd, E. N. Brothers, K. N. Kudin, V. N. Staroverov, T. A. Keith, R. Kobayashi, J. Normand, K. Raghavachari, A. P. Rendell, J. C. Burant, S. S. Iyengar, J. Tomasi, M. Cossi, J. M.

Millam, M. Klene, C. Adamo, R. Cammi, J. W. Ochterski, R. L. Martin, K. Morokuma, O. Farkas, J. B. Foresman, and D. J. Fox, Gaussian, Inc., Wallingford CT, **2016**.

22. Zhao, Y.; Truhlar, D. G. *Chem. Phys. Lett.* **2011**, *502*, 1–13.

23. Krishnan, R.; Binkley, J. S.; Seeger, R.; Pople, J. A. *J. Chem. Phys.* **1980**, *72*, 650–654.

24. McLean, A. D.; Chandler, G. S. *J. Chem. Phys.* **1980**, *72*, 5639–5648.

25. Cancès, M. T.; Mennucci, B. B.; Tomasi, J. A new integral equation formalism for the polarizable continuum model: Theoretical background and applications to isotropic and anisotropic dielectrics. *J. Chem. Phys.* **1997**, *107*, 3032−3041.

26. Hehre, W. J.; Ditchfield, R.; Pople, J. A. Self—Consistent Molecular Orbital Methods. XII. Further Extensions of Gaussian—Type Basis Sets for Use in Molecular Orbital Studies of Organic Molecules. *J. Chem. Phys.* **1972**, *56*, 2257–2261.

27. Lorenc, C.; Reeves, J. T.; Busacca, C. A.; Senanayake, C. H. *Tetrahedron Lett.* **2015**, *56*, 1280-1282.

28. Katritzky, A. R.; Chunming, C.; Sandeep. K. S. *J. Org. Chem.* **2006**, *71*, 3375-3380.

NMR Spectra for Novel Compounds

<u>Discovery of the Alkyl Transfer Process (Scheme 2A)</u>

2

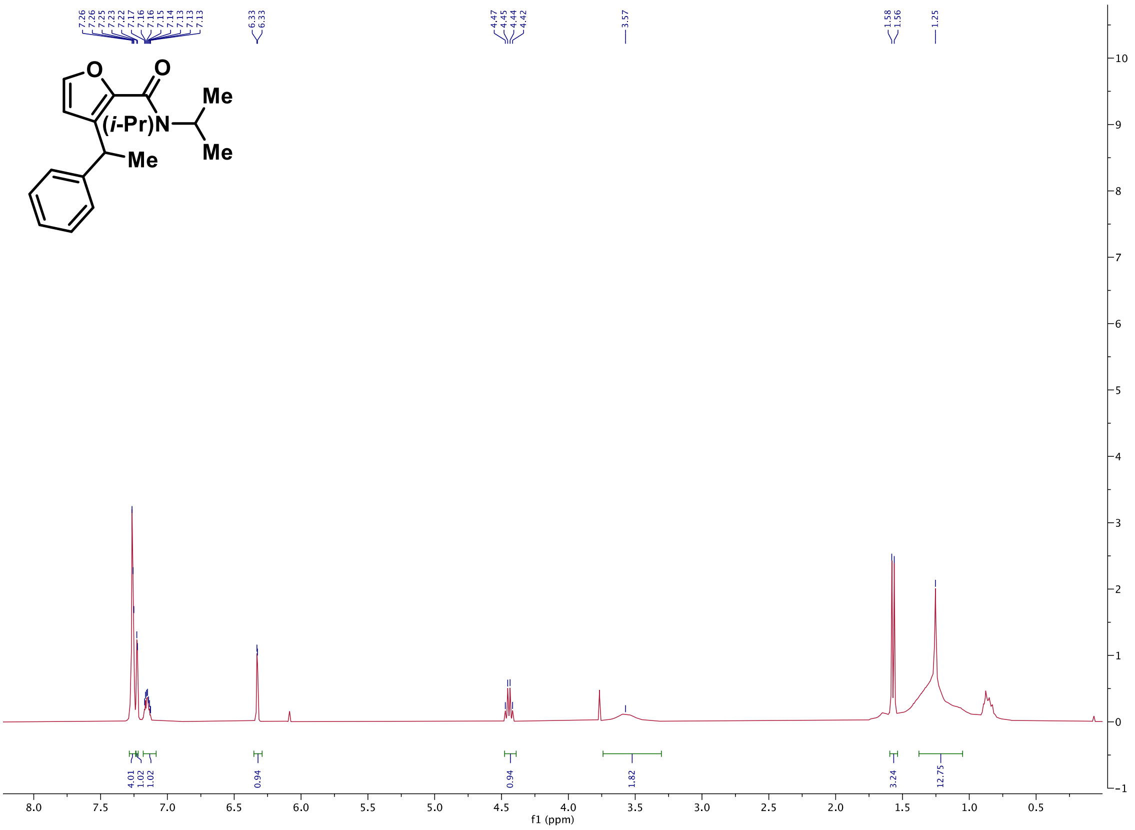

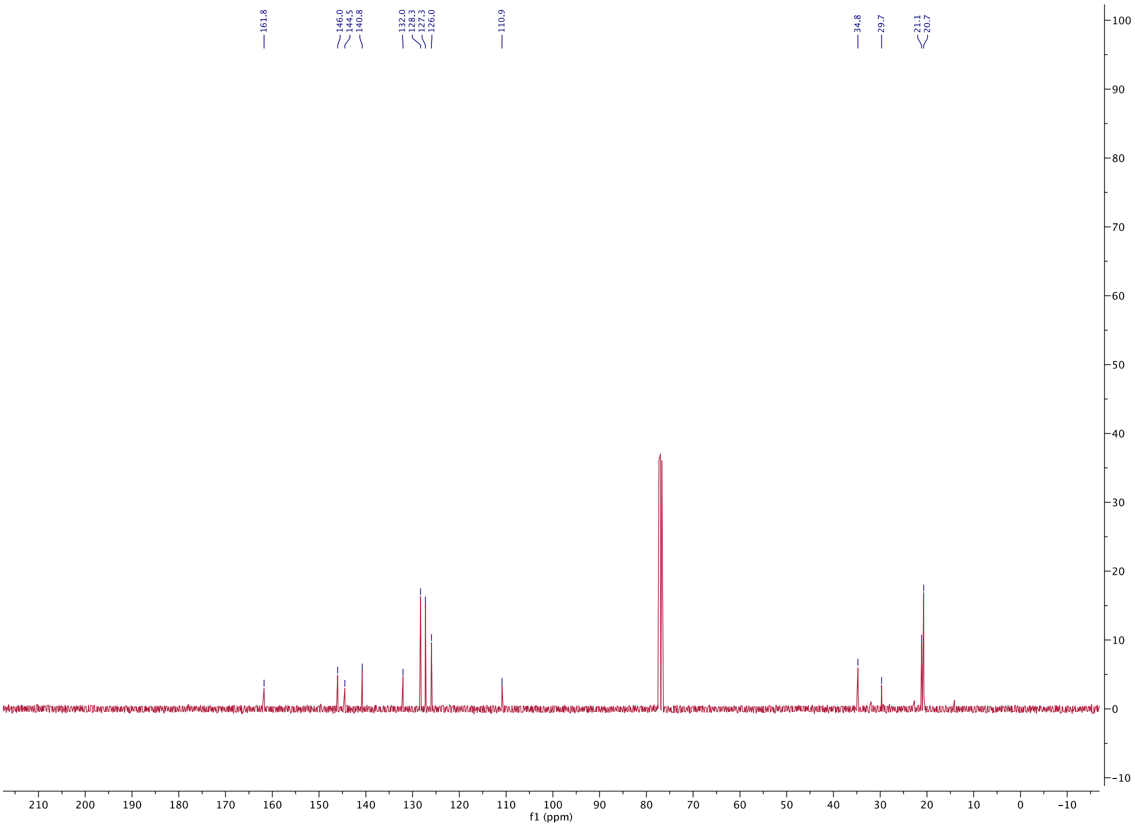

3

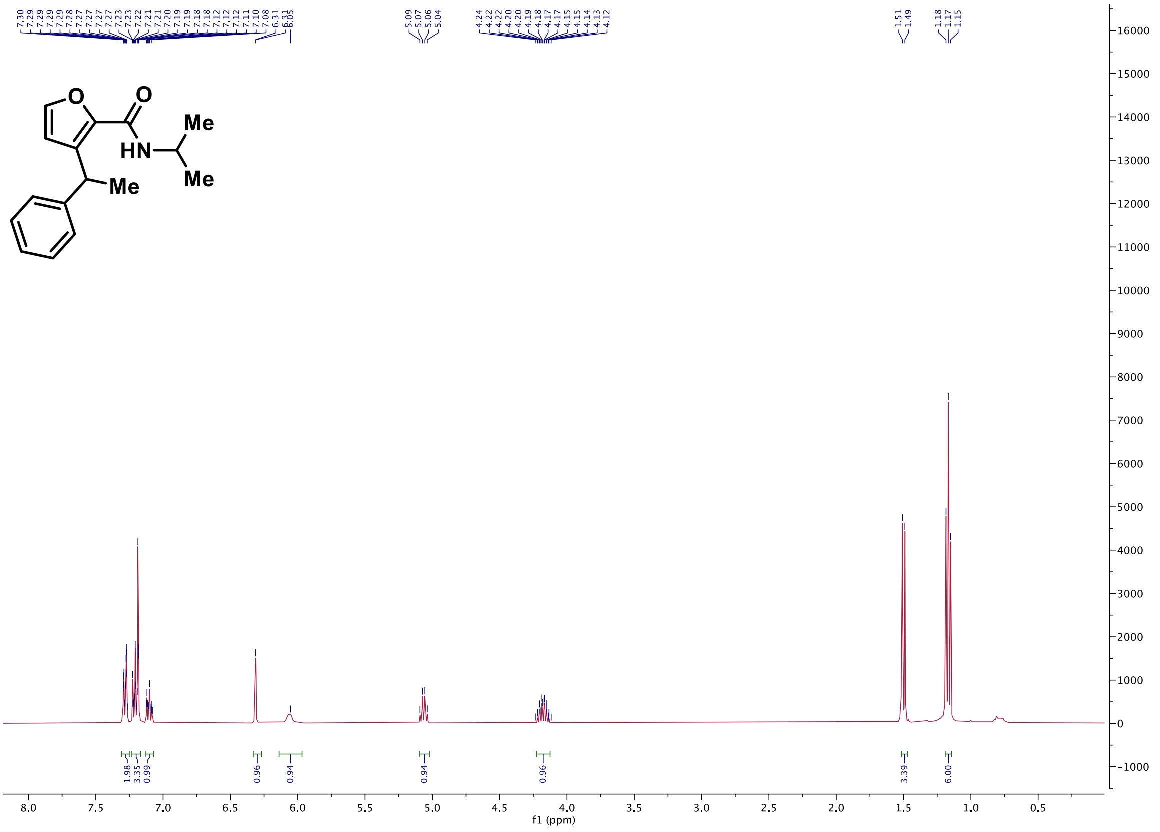
O
HN
Me
Me
Me
1.98
3.35
0.99
0.96
0.94
0.94
0.96
3.39
6.00
f1 (ppm)

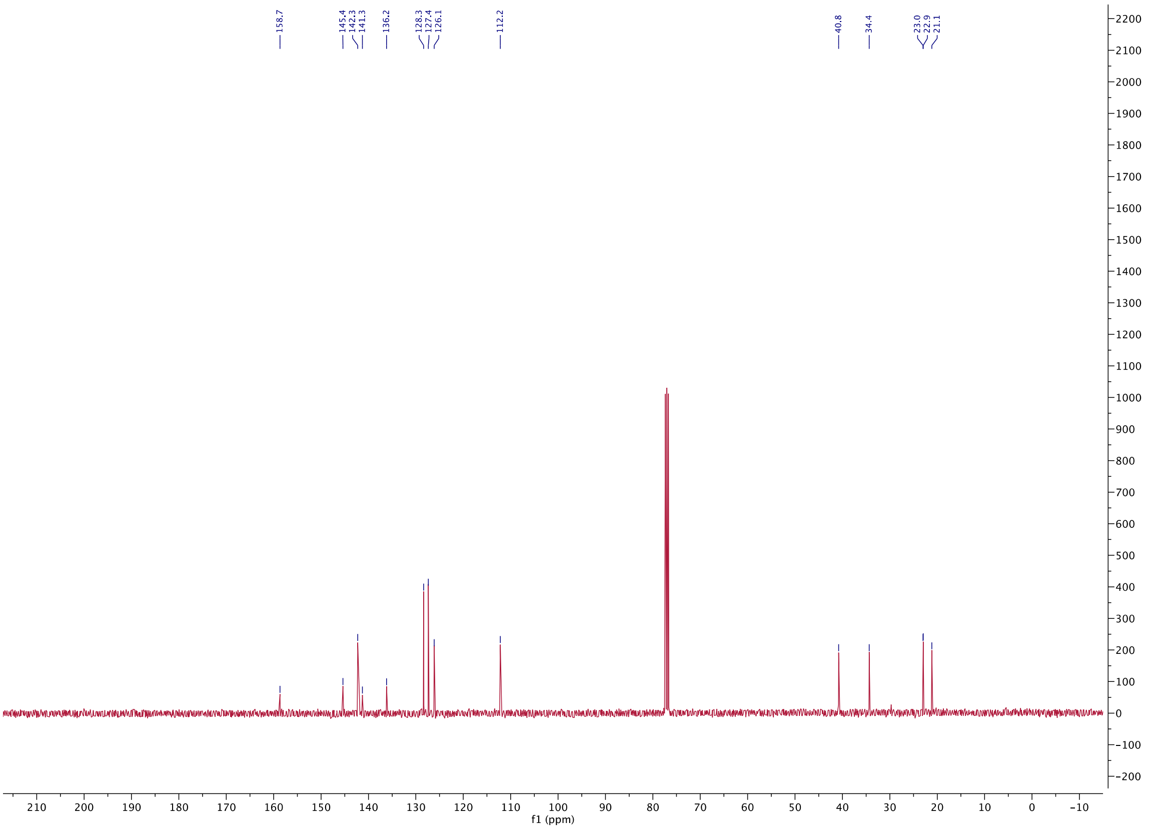
158.7
145.4
142.3
141.3
136.2
128.3
127.4
126.1
112.2
40.8
34.4
23.0
22.9
21.1
f1 (ppm)

Intramolecular Isopropyl N→C Alkyl Transfer Reactions: Substrates (Table 1A)

1b

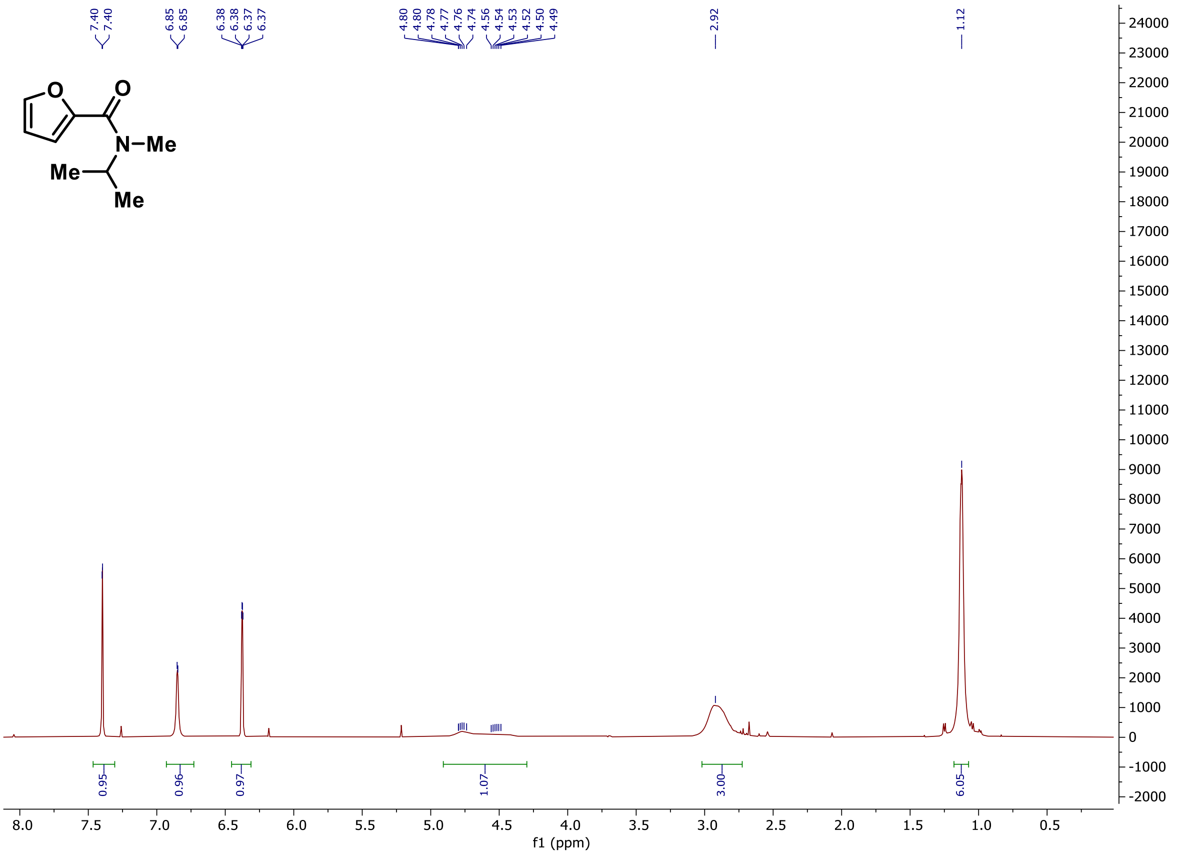

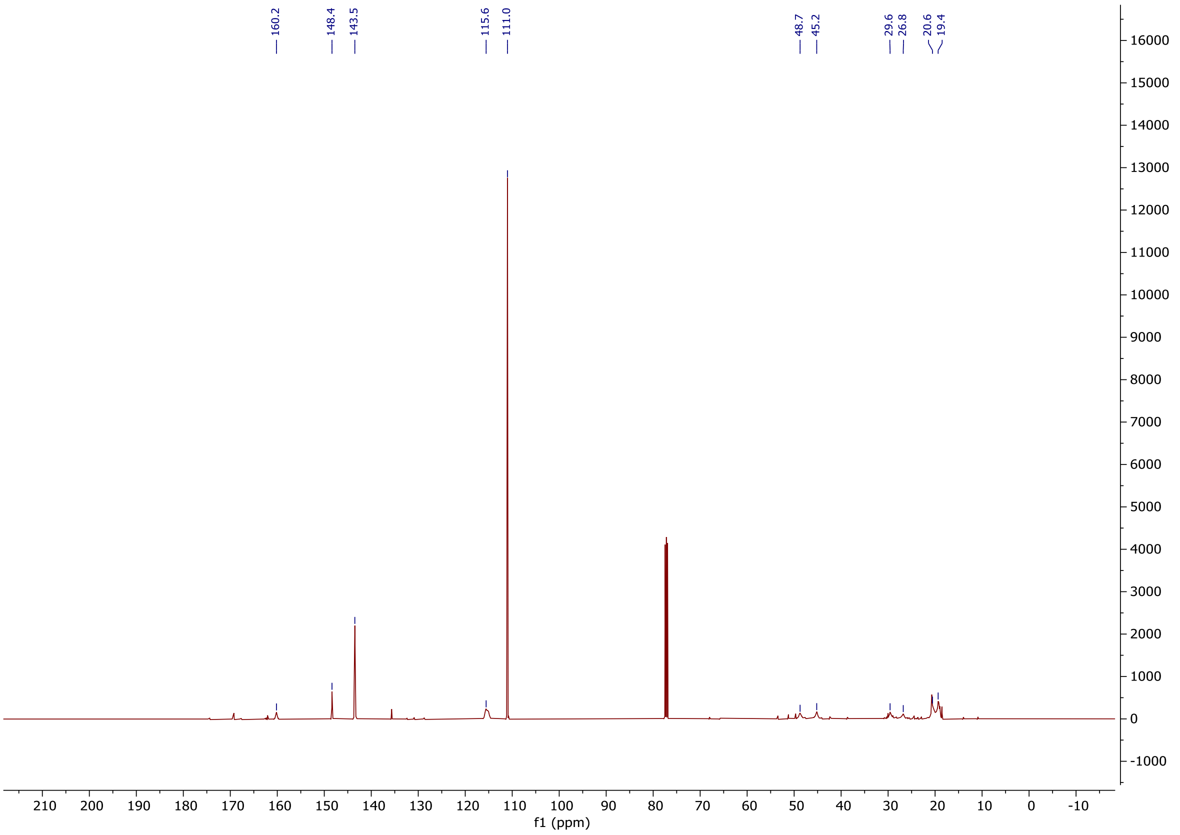

1c

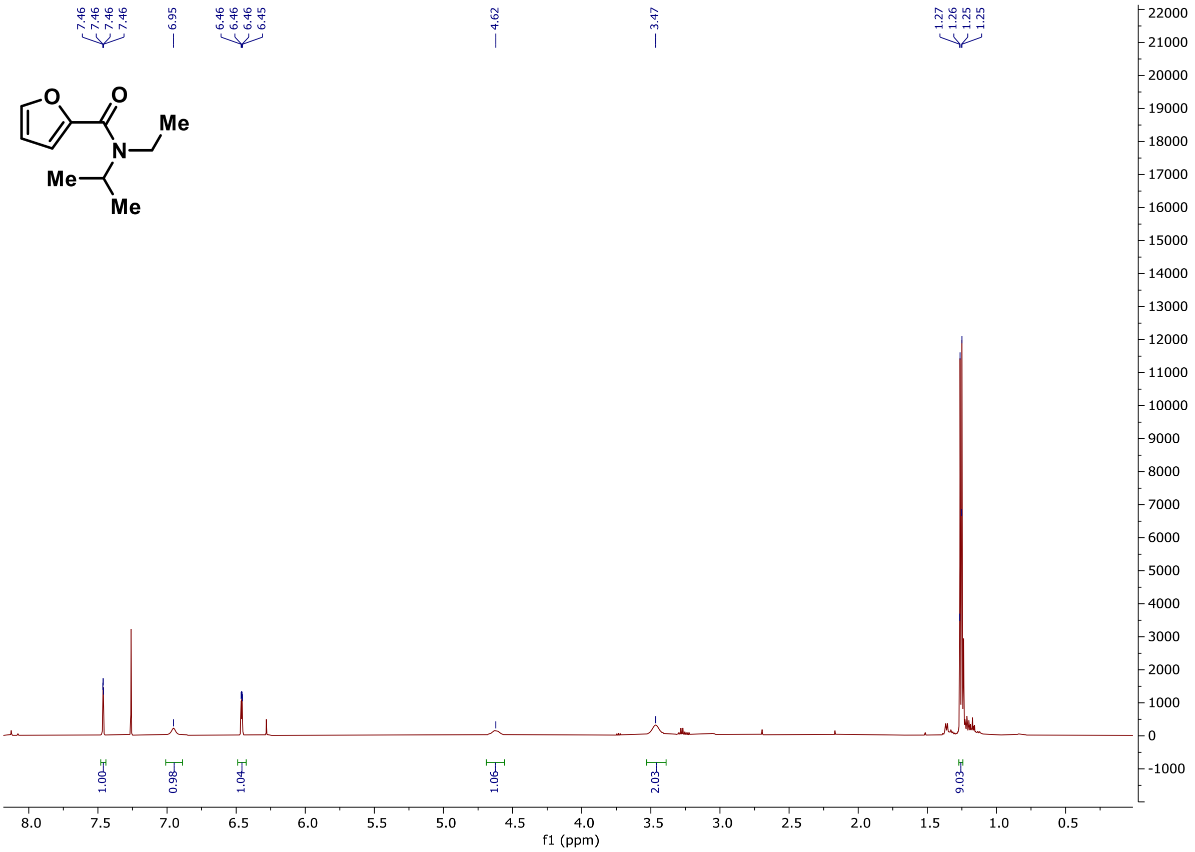
O
O
Me
N
Me
Me
7.46
7.46
7.46
7.46
6.95
6.46
6.46
6.46
6.45
4.62
3.47
1.27
1.26
1.25
1.25
1.00
0.98
1.04
1.06
2.03
9.03
f1 (ppm)

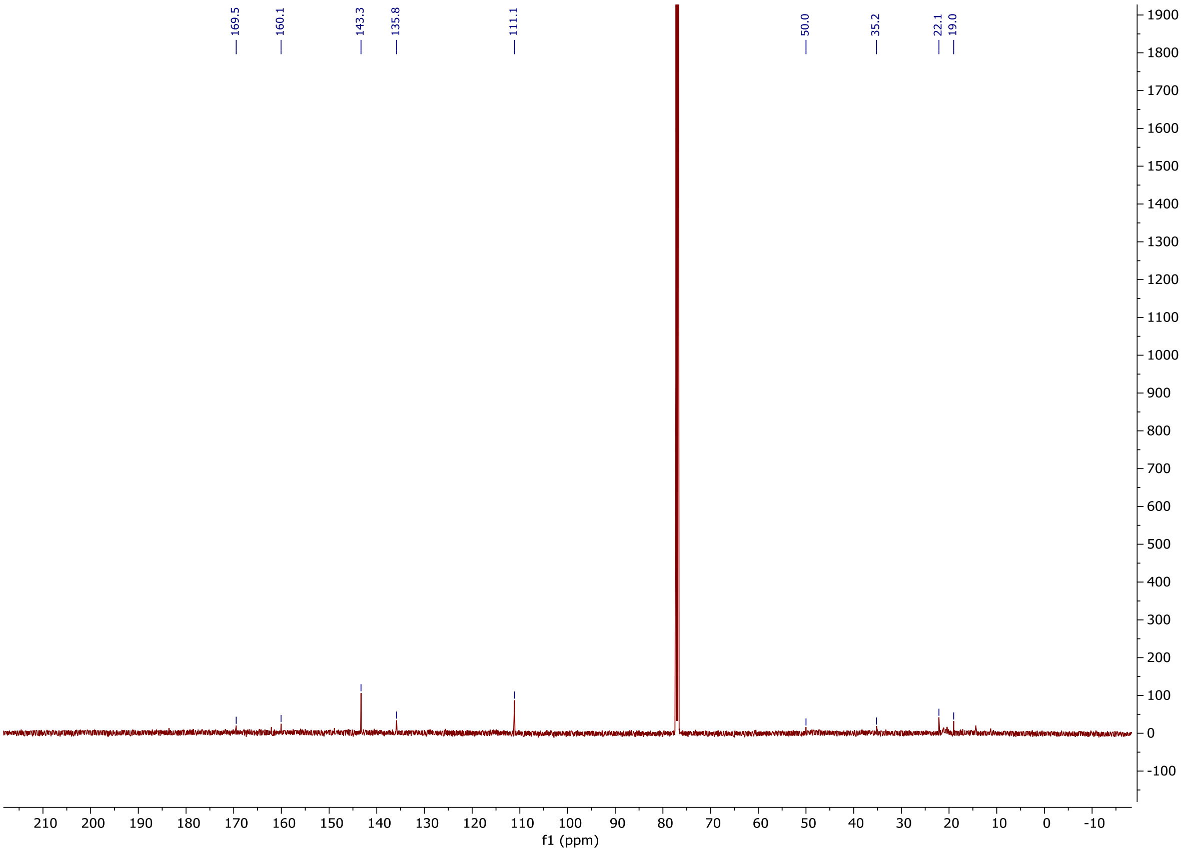
169.5
160.1
143.3
135.8
111.1
50.0
35.2
22.1
19.0
f1 (ppm)

1d

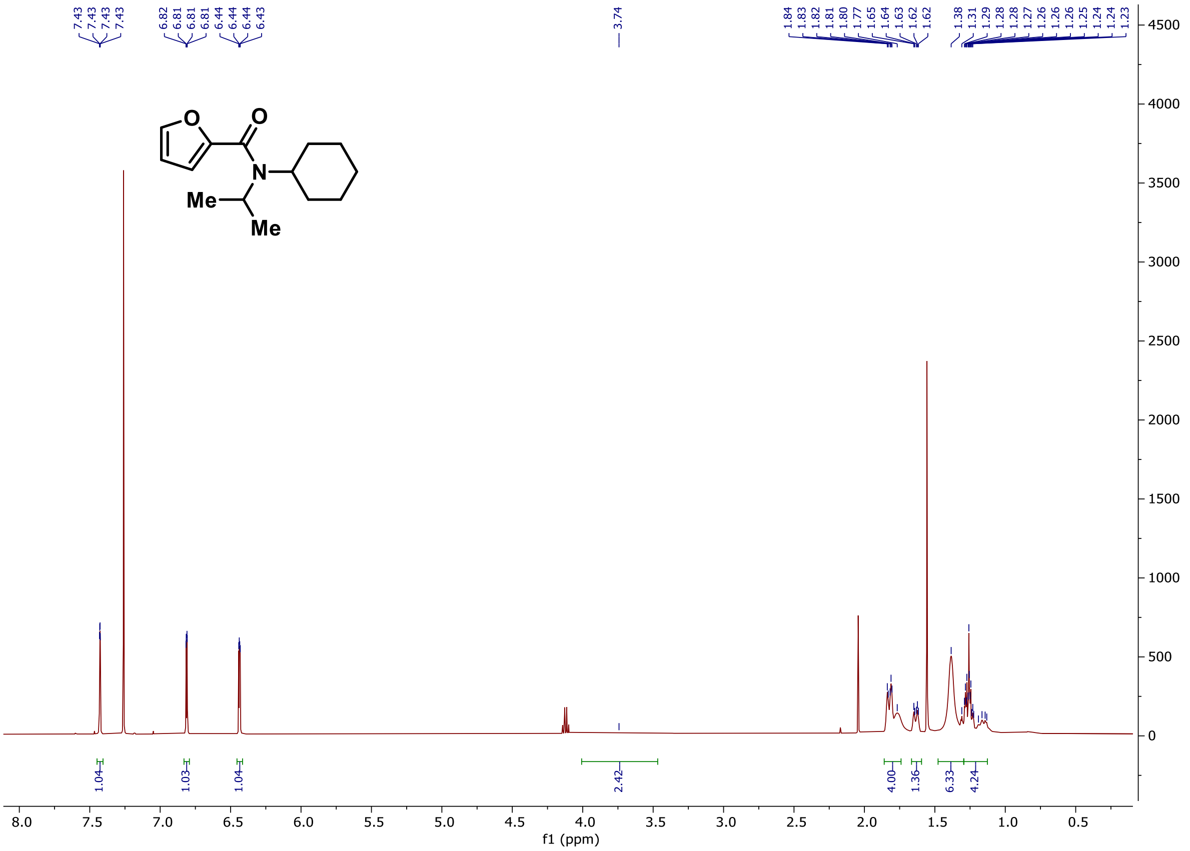
7.43 7.43 7.43 7.43
6.82 6.81 6.81 6.81 6.44 6.44 6.44 6.43
3.74
1.84 1.83 1.82 1.81 1.80 1.77 1.65 1.64 1.63 1.62 1.62
1.38 1.31 1.29 1.28 1.28 1.27 1.26 1.26 1.25 1.24 1.24 1.23
O
O
N
Me
Me
1.04
1.03
1.04
2.42
4.00
1.36
6.33
4.24
f1 (ppm)

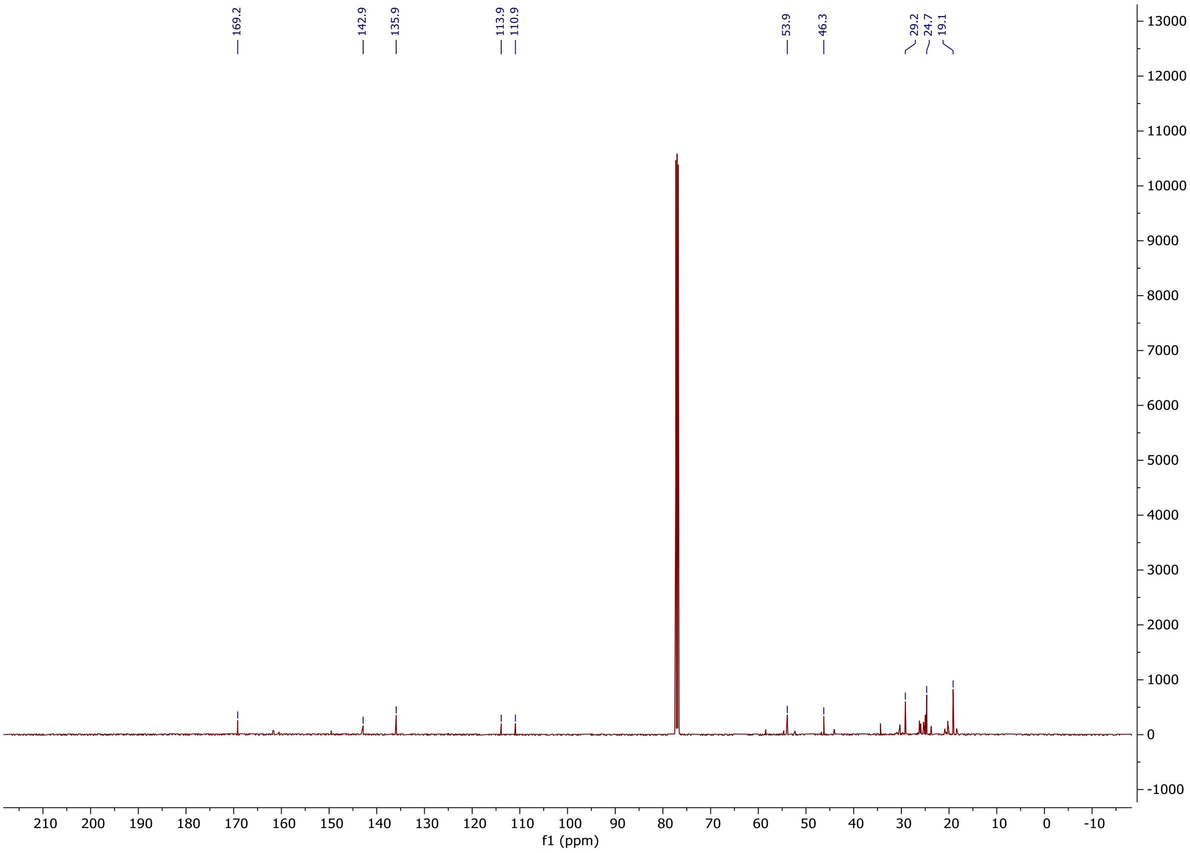
169.2
142.9
135.9
113.9
110.9
53.9
46.3
29.2
24.7
19.1
f1 (ppm)

1e

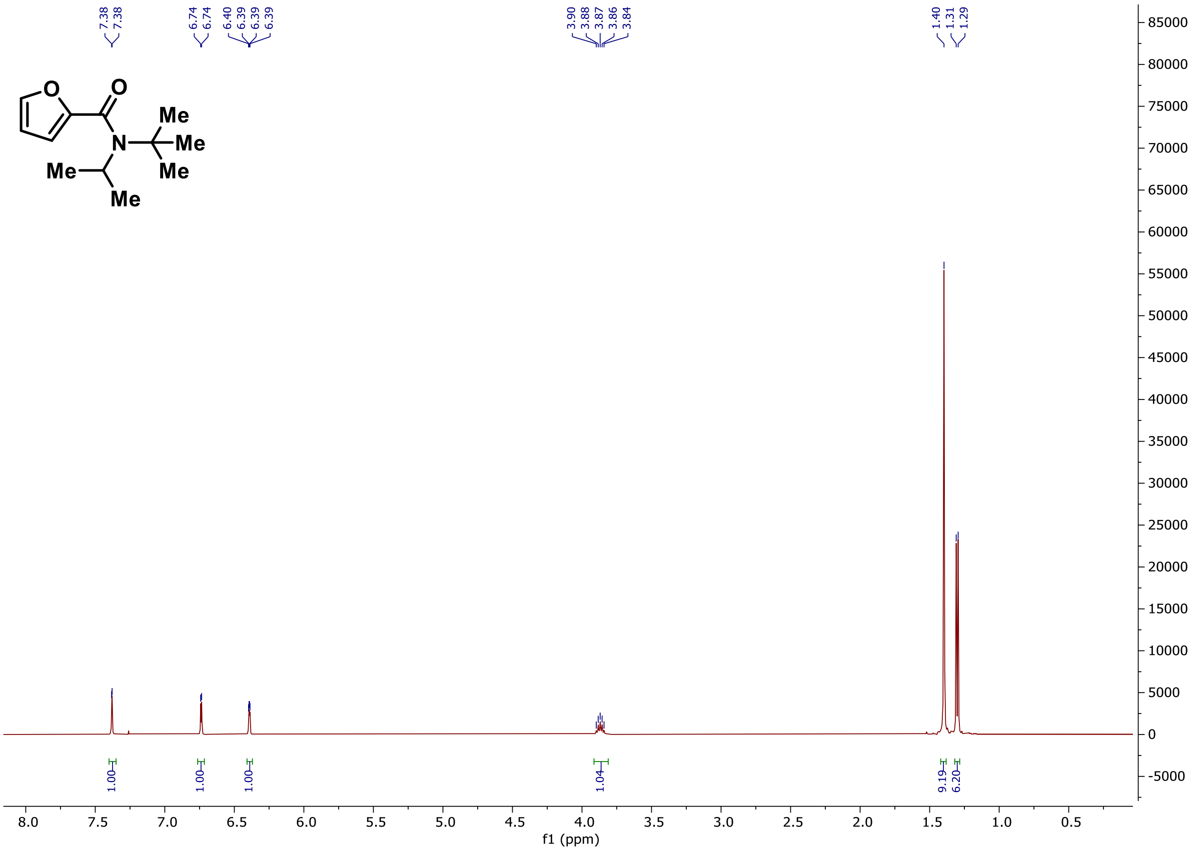

O
O
N
Me
Me
Me
Me
Me
7.38
7.38
6.74
6.74
6.40
6.39
6.39
6.39
3.90
3.88
3.87
3.86
3.84
1.40
1.31
1.29
1.00
1.00
1.00
1.04
9.19
6.20
f1 (ppm)

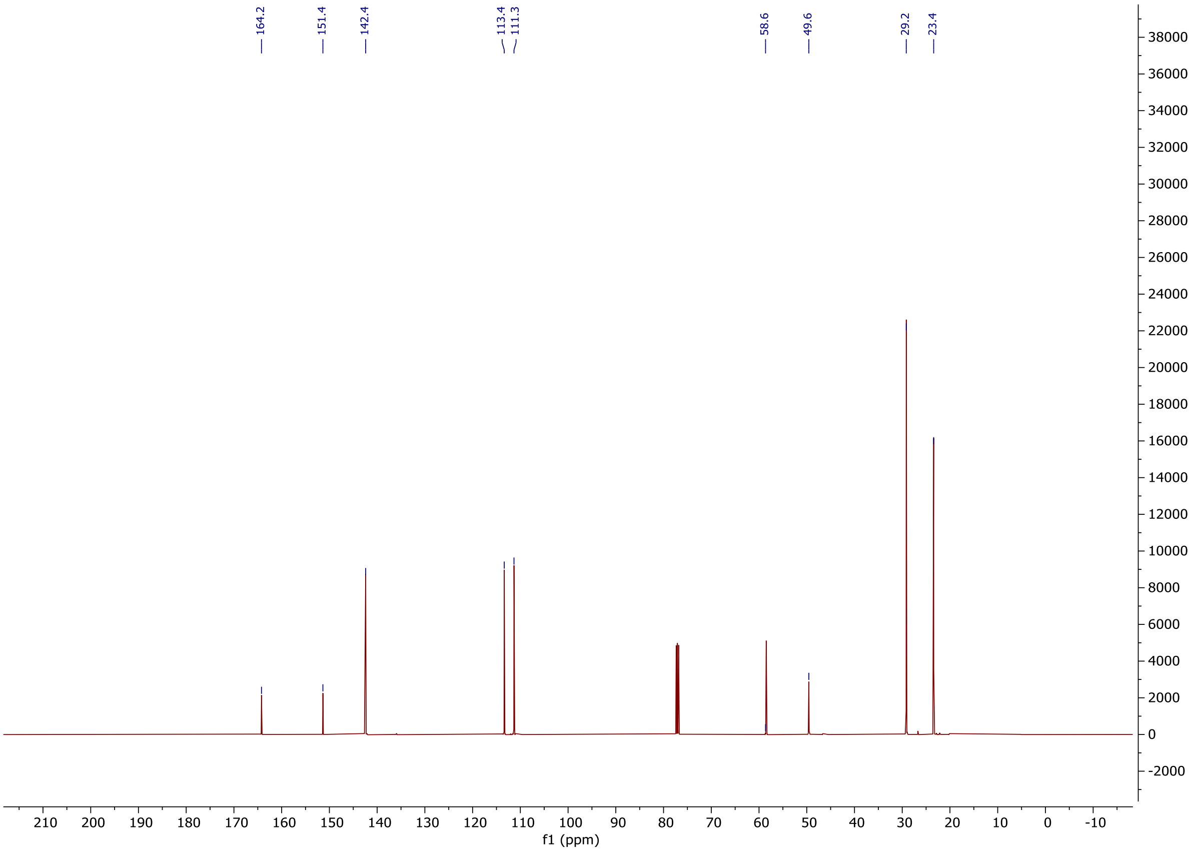

164.2
151.4
142.4
113.4
111.3
58.6
49.6
29.2
23.4
f1 (ppm)

1f

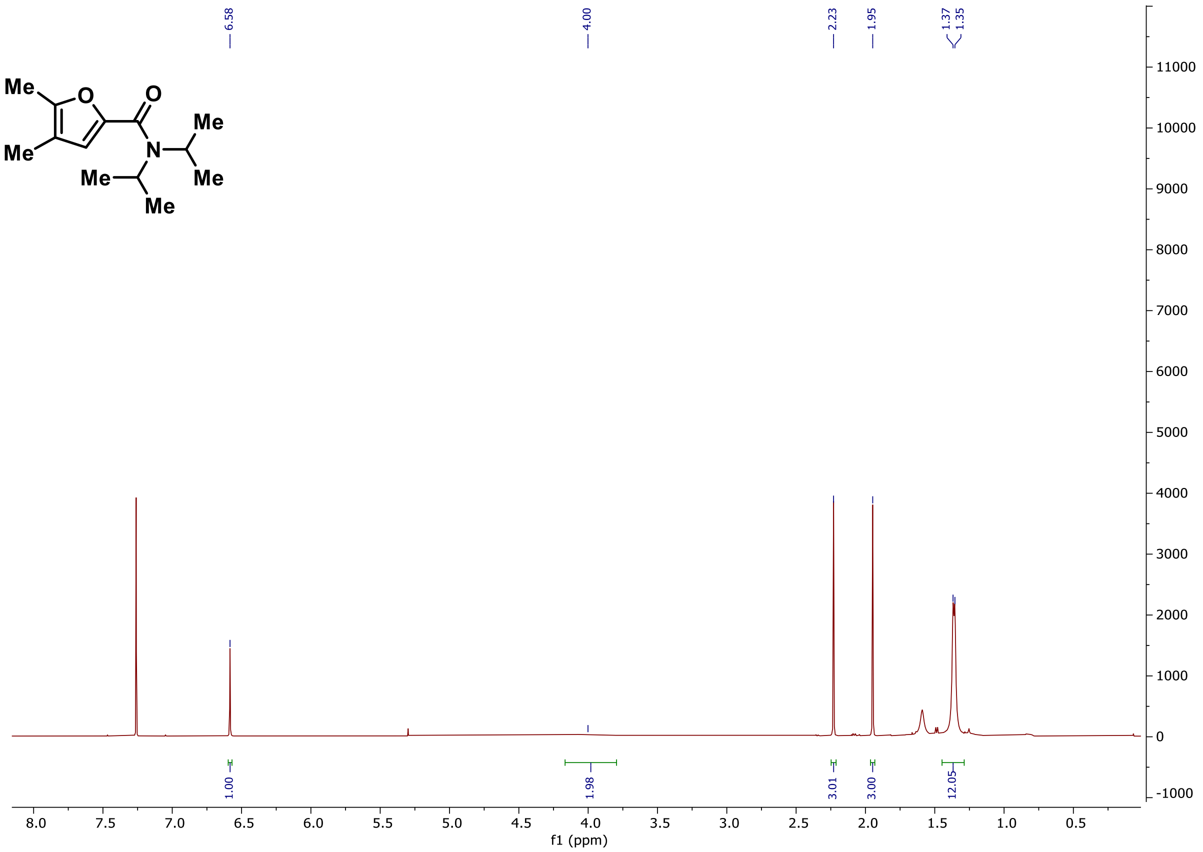

6.58
4.00
2.23
1.95
1.37
1.35
1.00
1.98
3.01
3.00
12.05
f1 (ppm)
Me
O
Me
N
Me
Me
Me

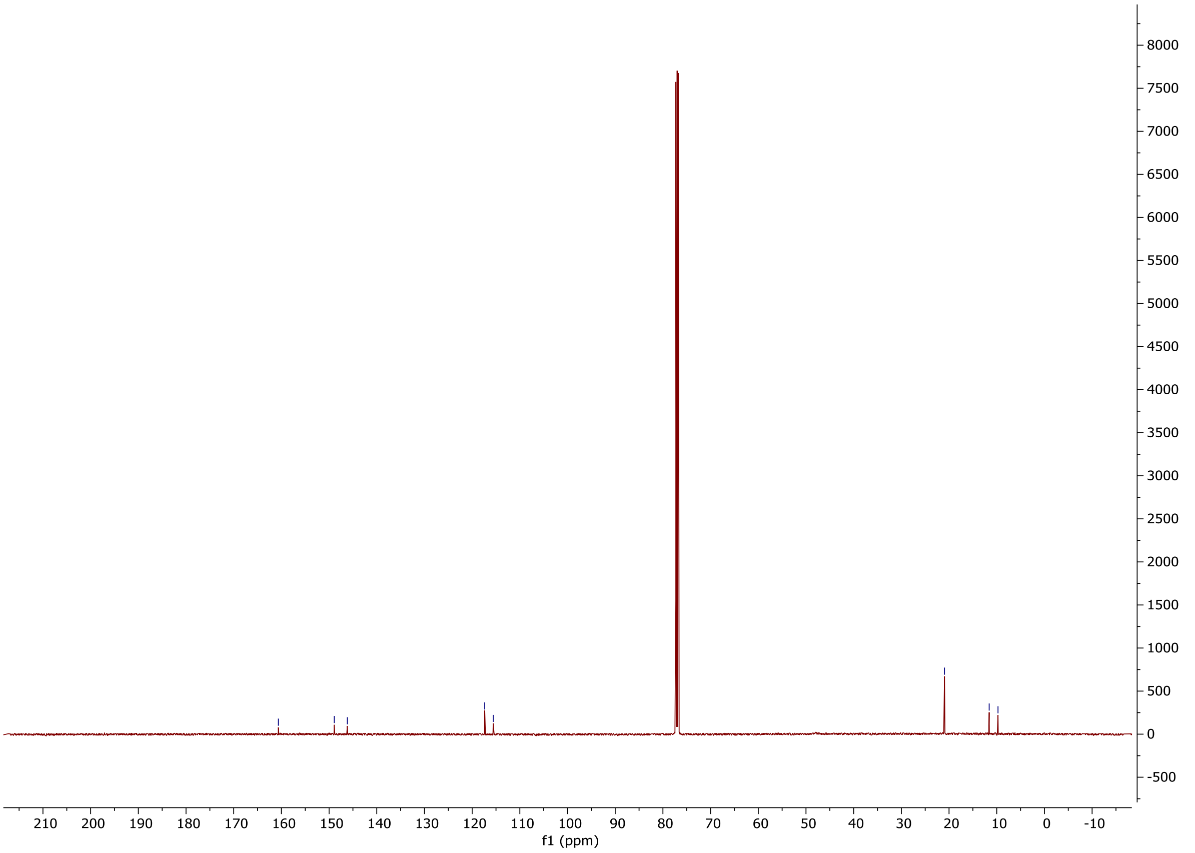

f1 (ppm)

1h

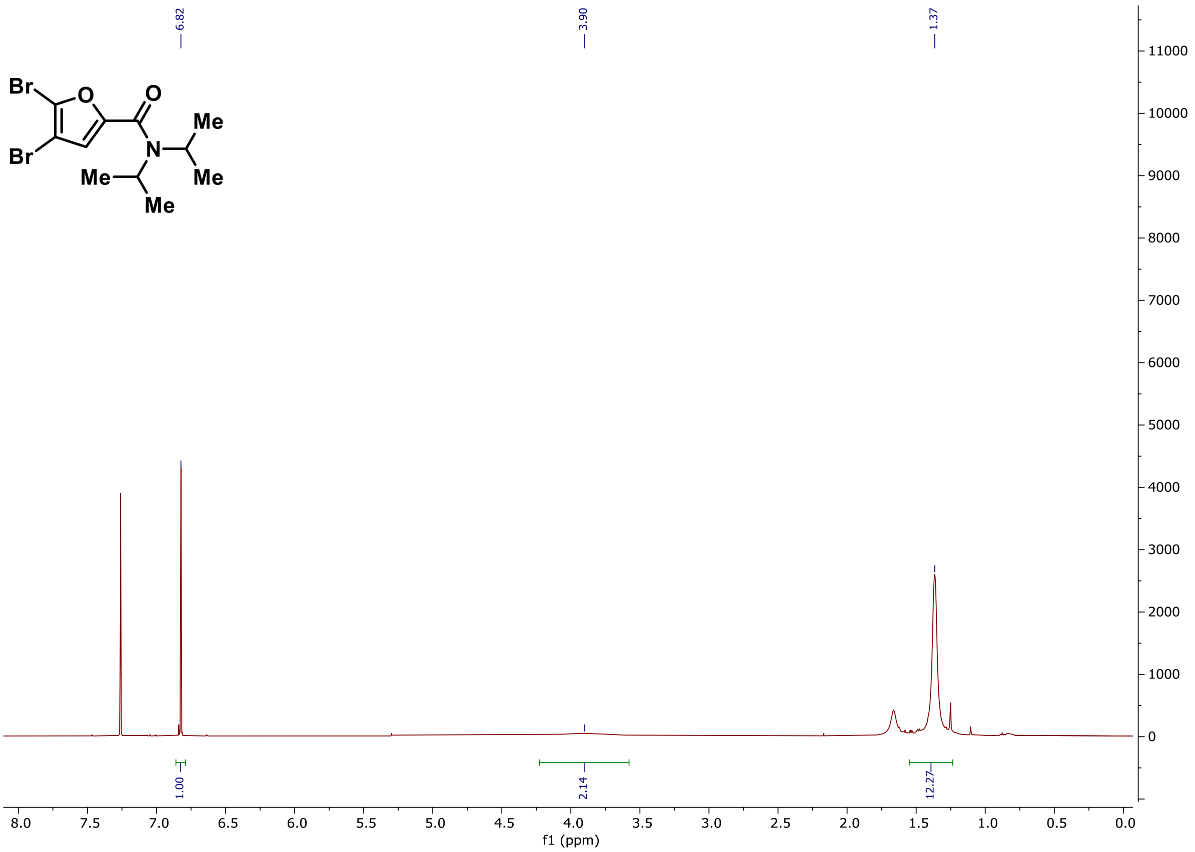

Br
O
O
Me
N
Br
Me
Me
Me
6.82
3.90
1.37
1.00
2.14
12.27
f1 (ppm)

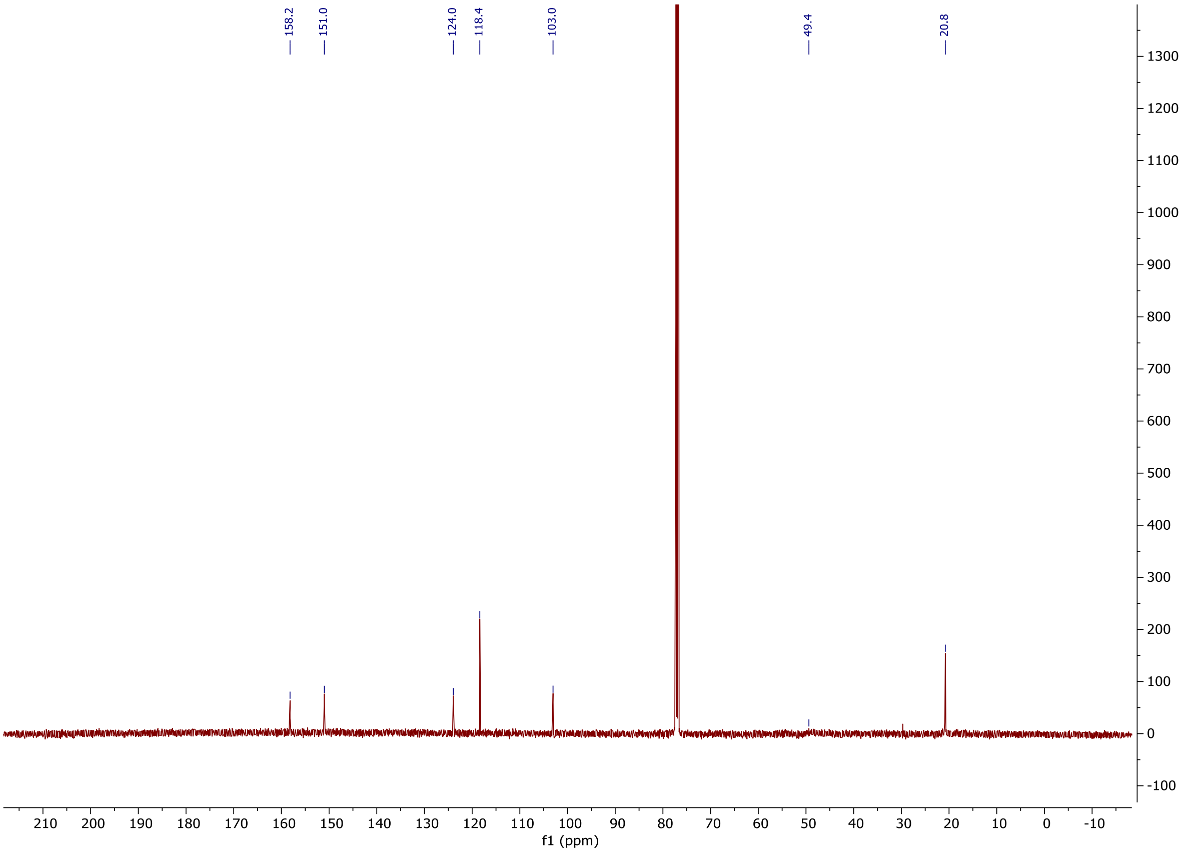

158.2
151.0
124.0
118.4
103.0
49.4
20.8
f1 (ppm)

1i

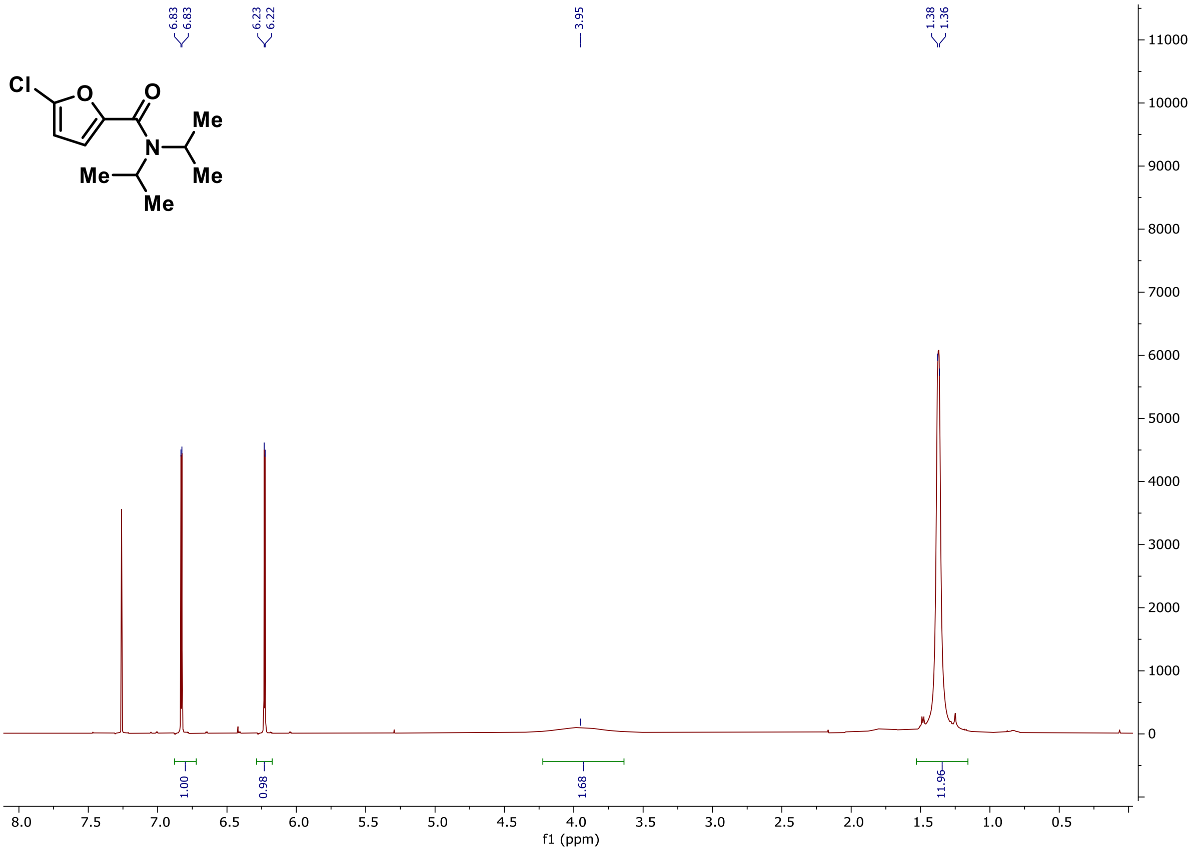
Cl
O
O
N
Me
Me
Me
Me
6.83
6.83
6.23
6.22
3.95
1.38
1.36
1.00
0.98
1.68
11.96
f1 (ppm)

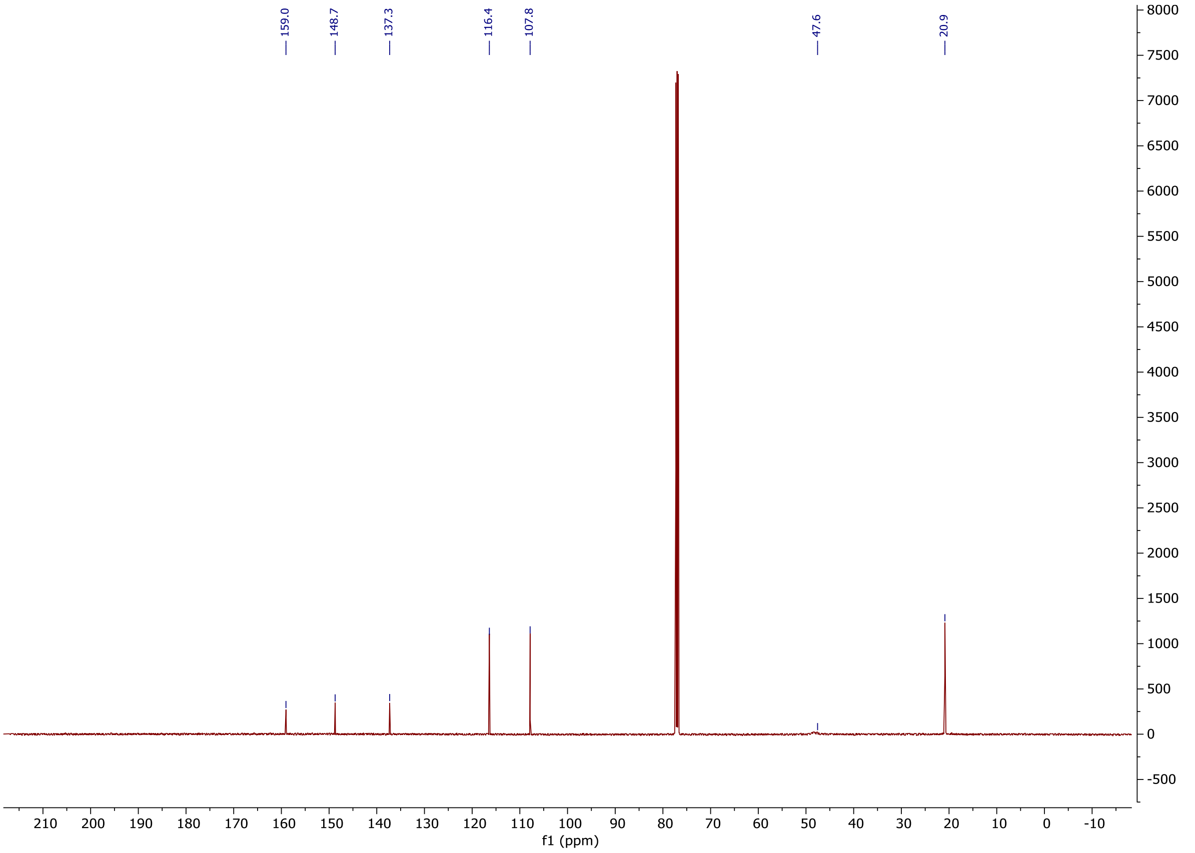
159.0
148.7
137.3
116.4
107.8
47.6
20.9
f1 (ppm)

1j

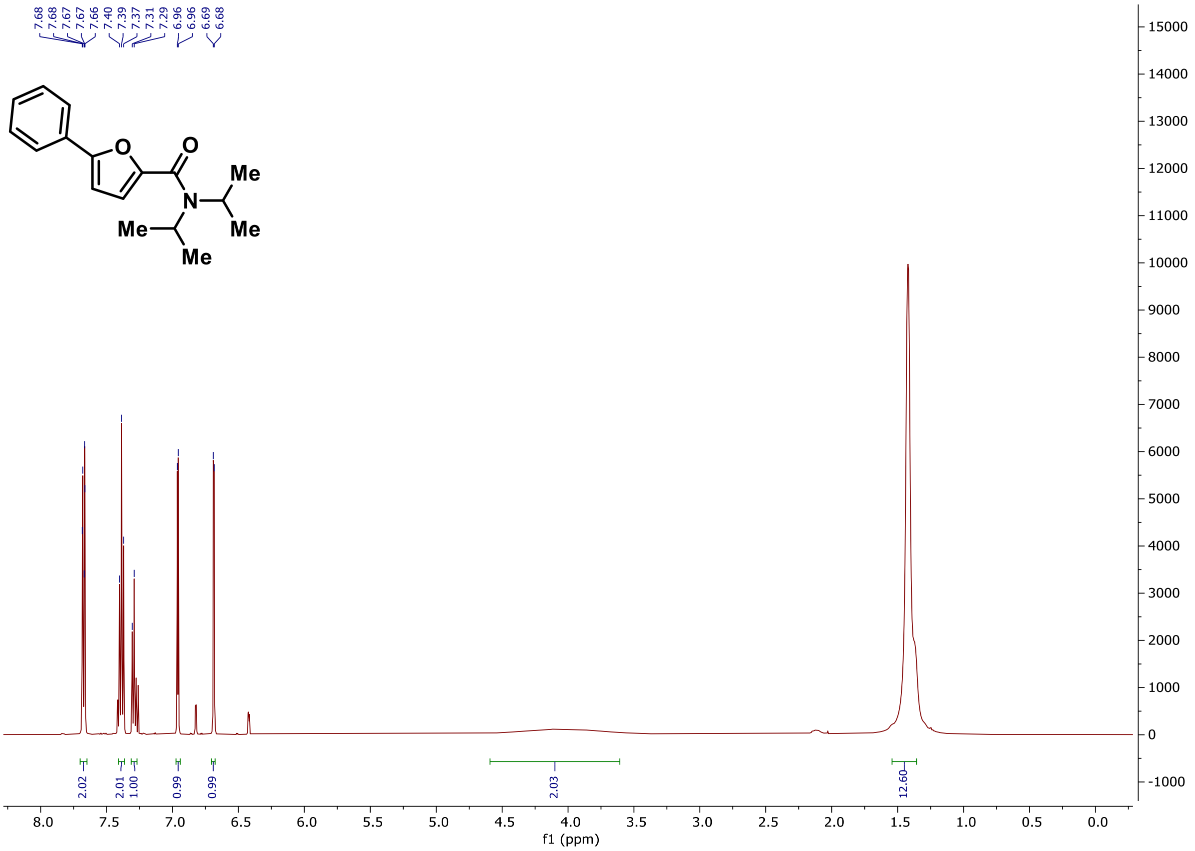

7.68 7.68 7.67 7.66 7.40 7.39 7.37 7.31 7.29 6.96 6.96 6.69 6.68
O
N
Me
Me
Me
Me
2.02
2.01
1.00
0.99
0.99
2.03
12.60
f1 (ppm)

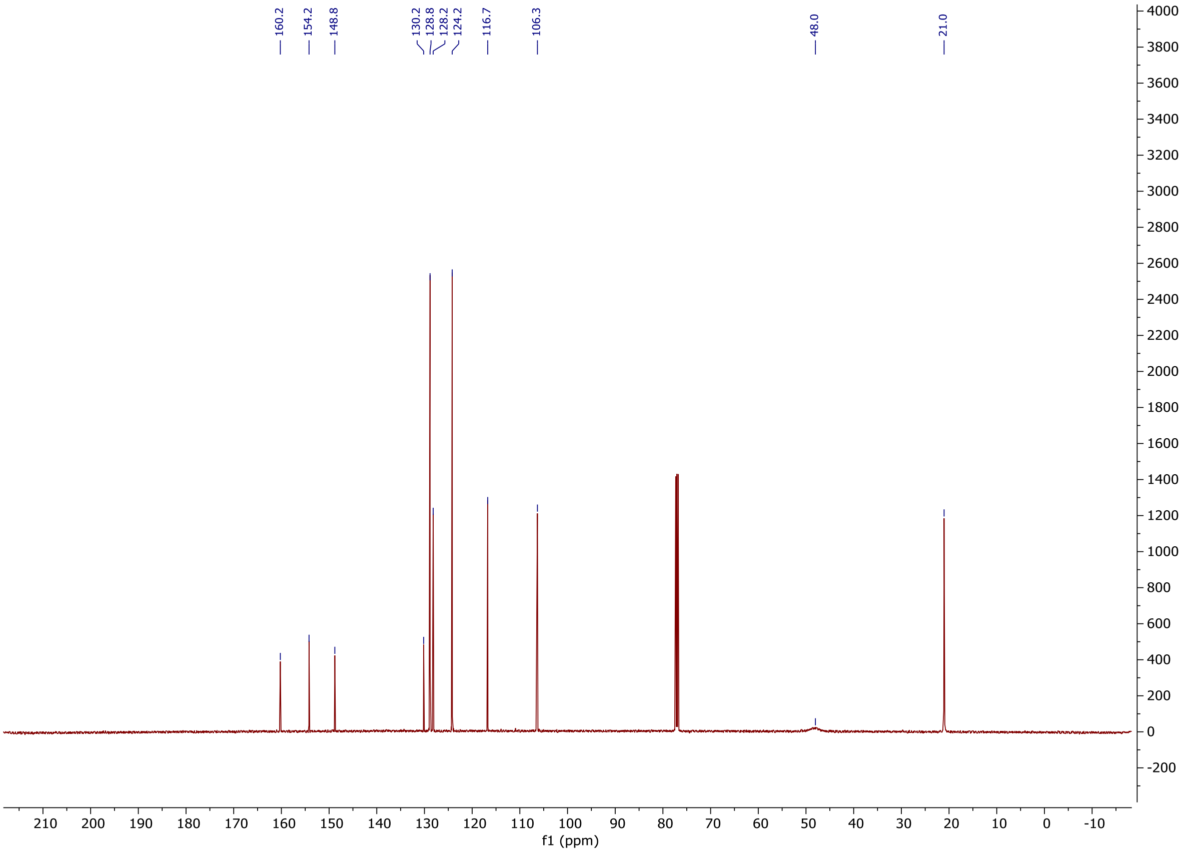

160.2
154.2
148.8
130.2
128.8
128.2
124.2
116.7
106.3
48.0
21.0
f1 (ppm)

1m

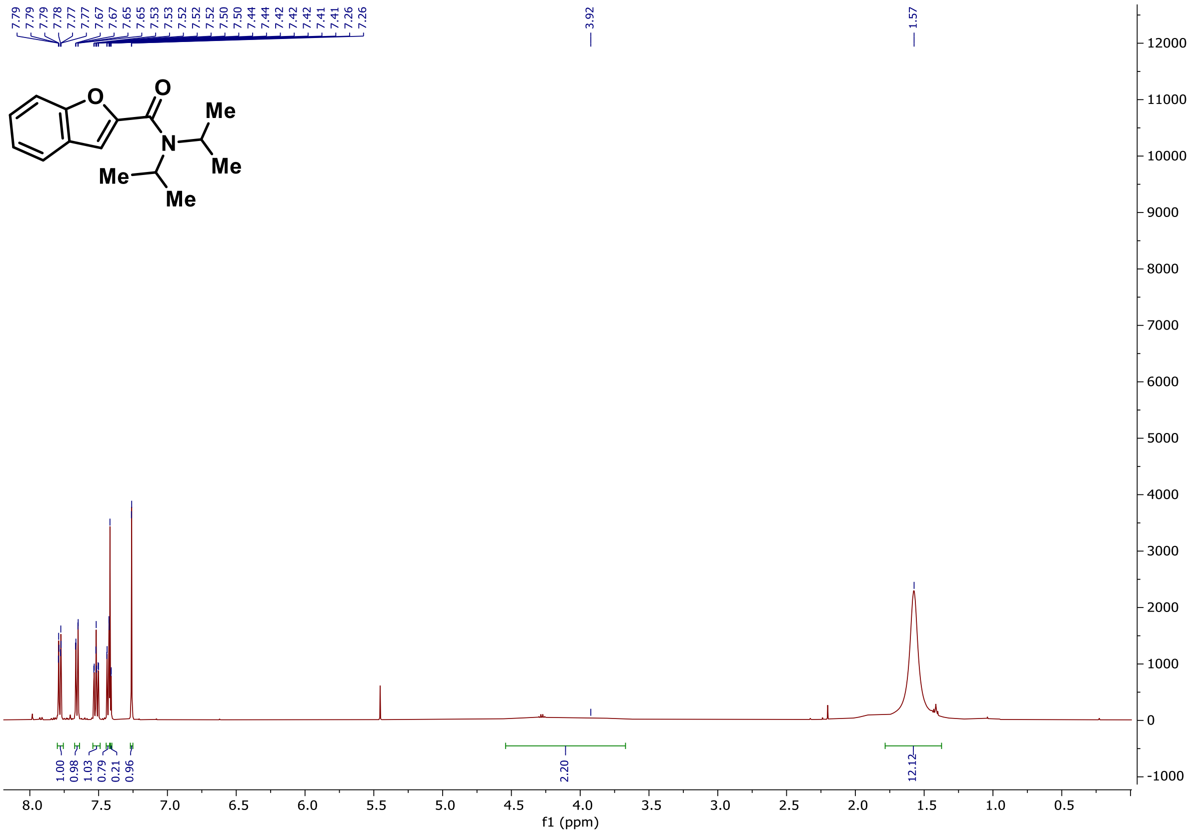

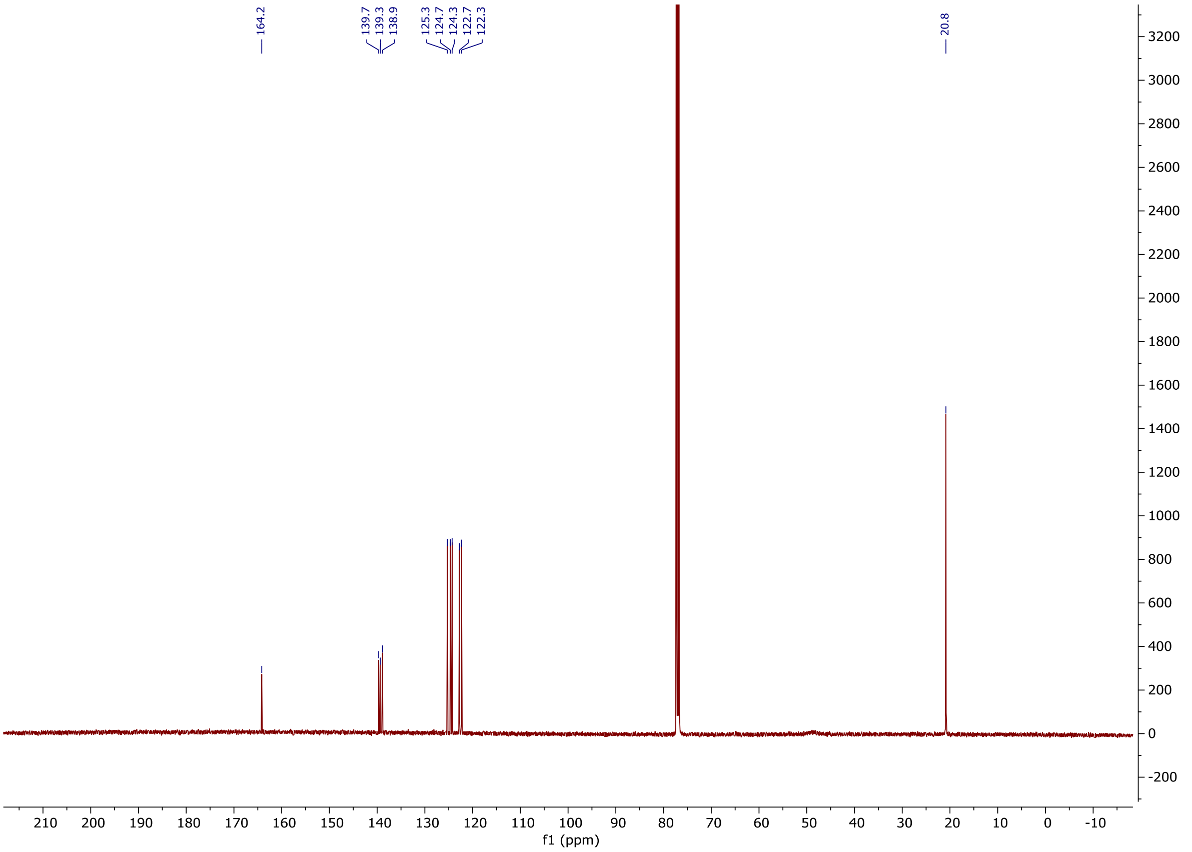

1n

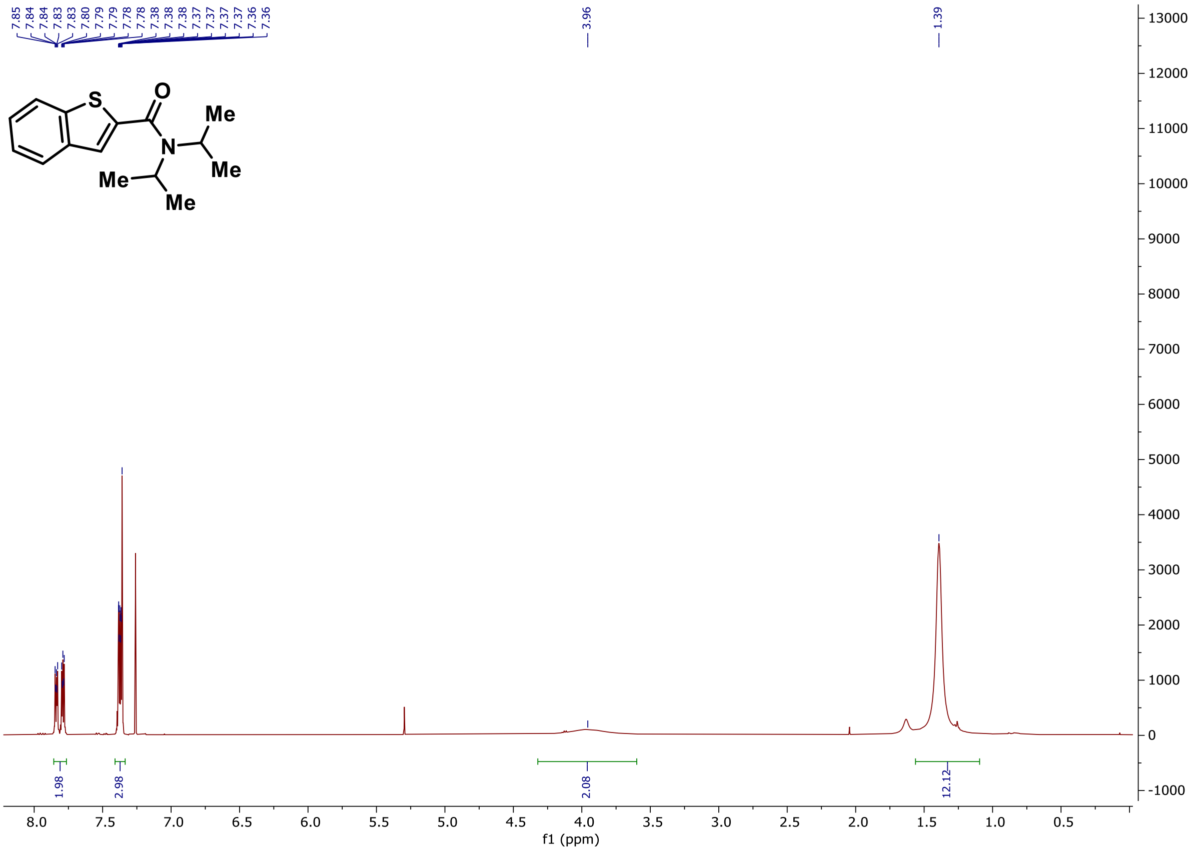

7.85
7.84
7.84
7.83
7.83
7.80
7.79
7.79
7.78
7.78
7.38
7.38
7.38
7.38
7.37
7.37
7.37
7.36
7.36
3.96
1.39
1.98
2.98
2.08
12.12
f1 (ppm)

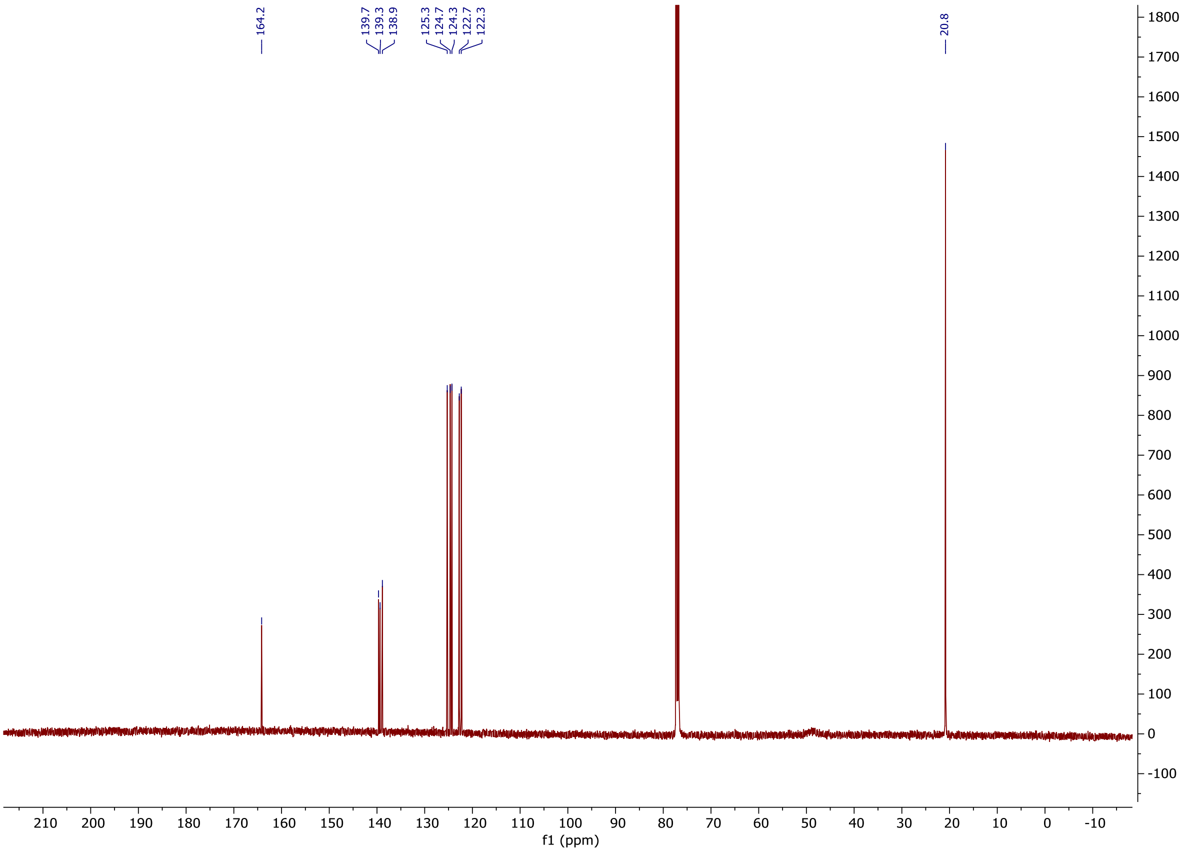

164.2
139.7
139.3
138.9
125.3
124.7
124.3
122.7
122.3
20.8
f1 (ppm)

Intramolecular Isopropyl N→C Alkyl Transfer Reactions: Products (Table 1A)

4a

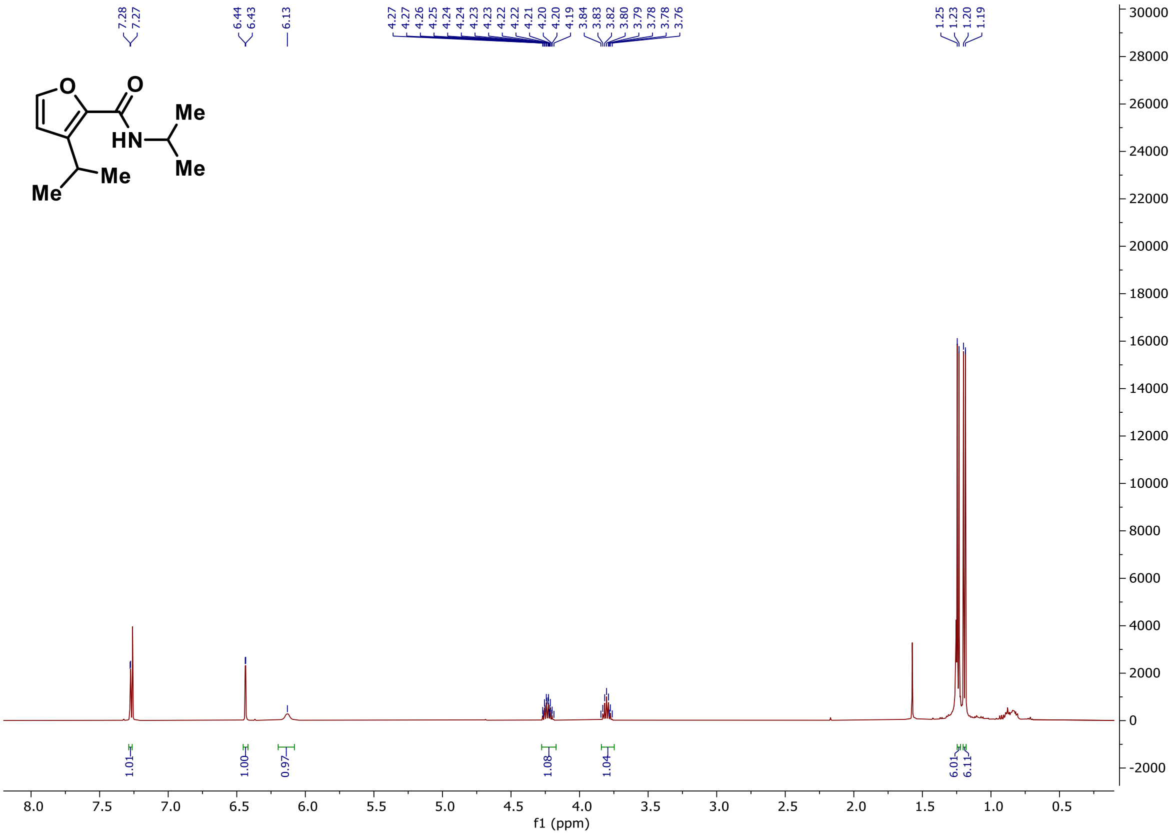

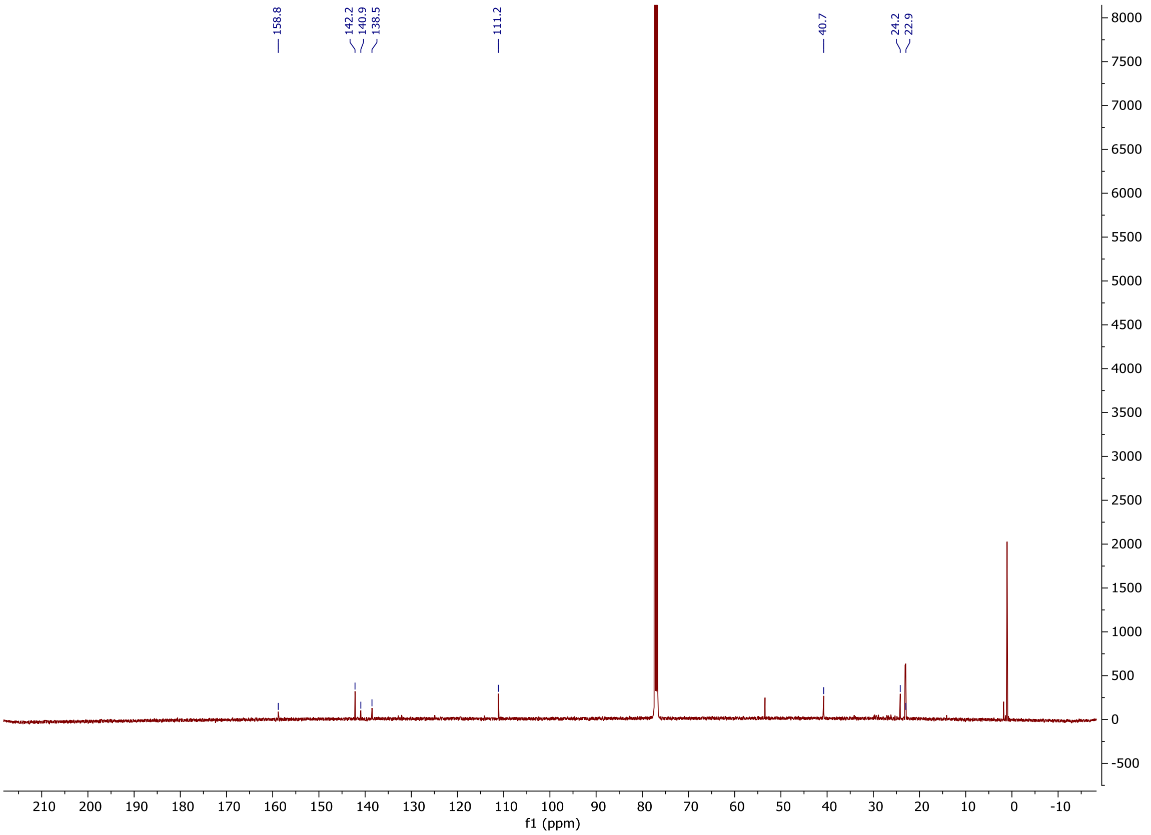

4b

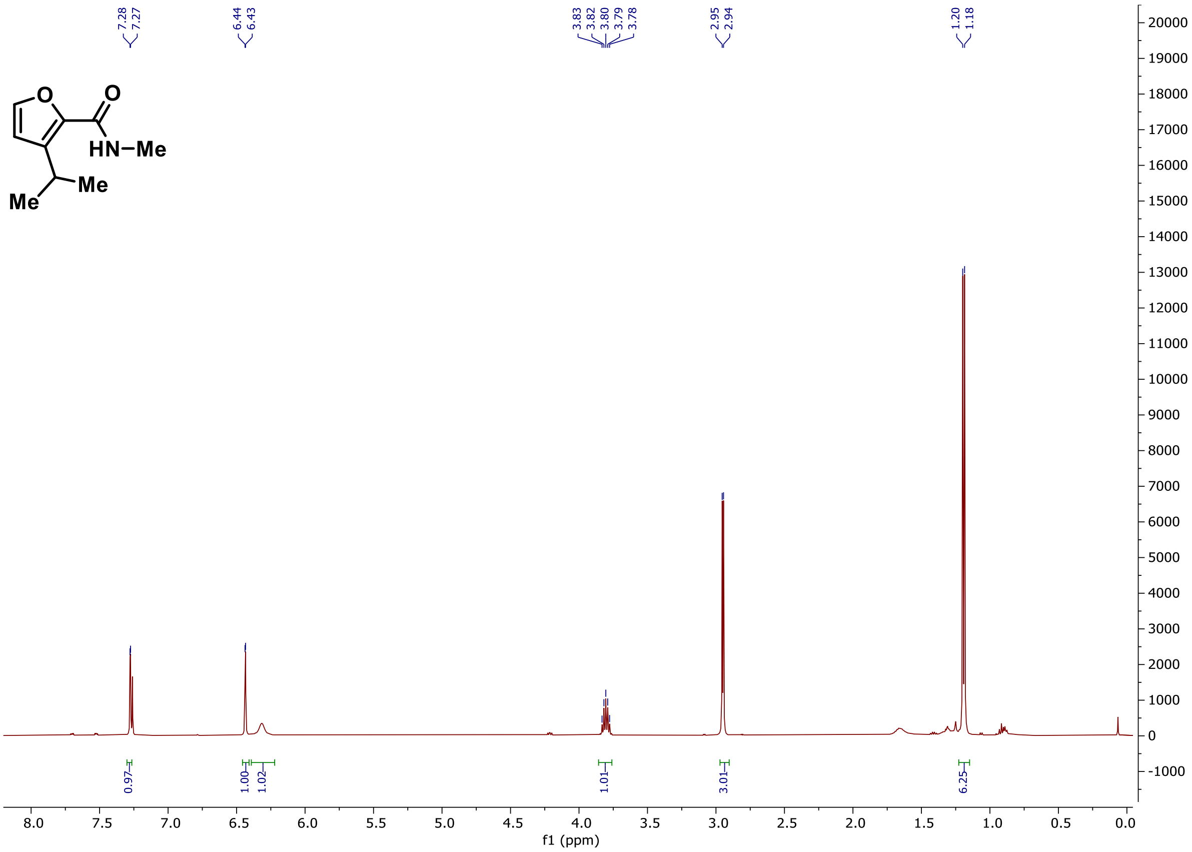
O
HN−Me
Me
Me
7.28
7.27
6.44
6.43
3.83
3.82
3.80
3.79
3.78
2.95
2.94
1.20
1.18
0.97
1.00
1.02
1.01
3.01
6.25
f1 (ppm)

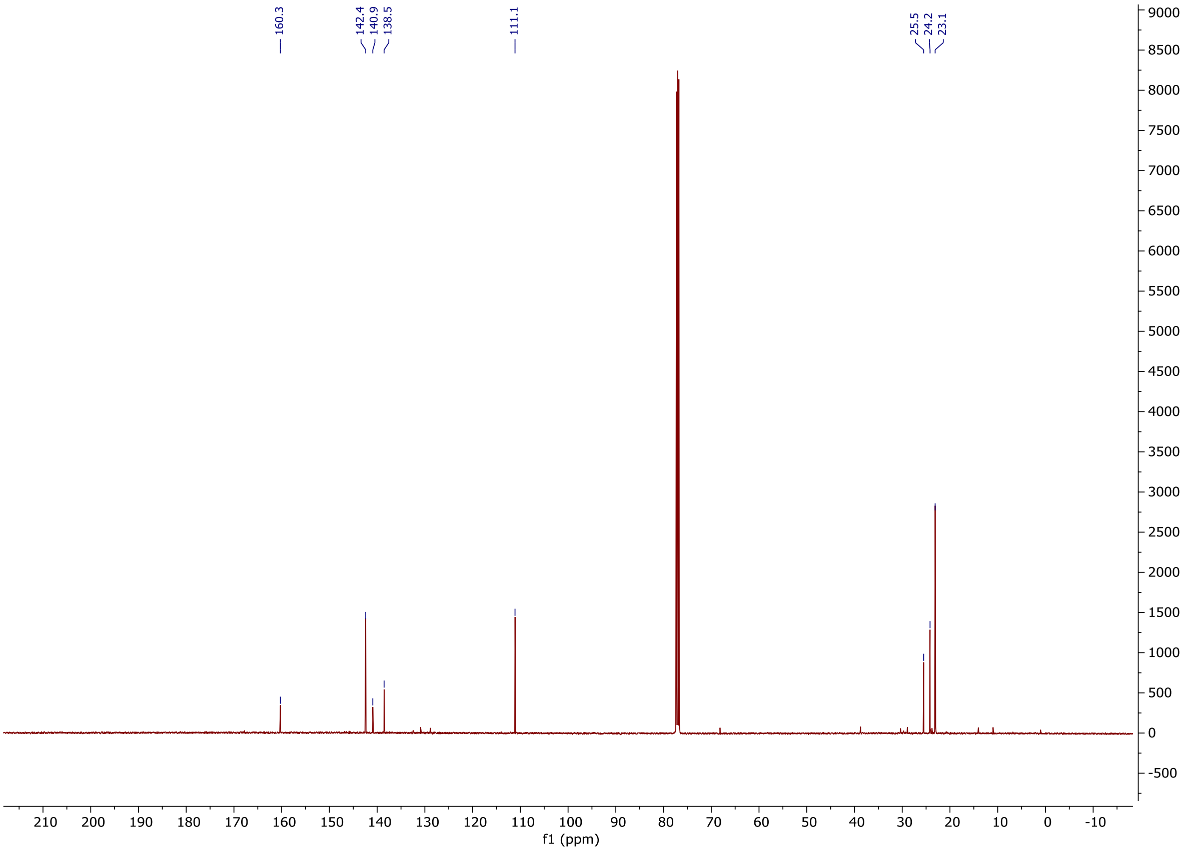
160.3
142.4
140.9
138.5
111.1
25.5
24.2
23.1
f1 (ppm)

4c

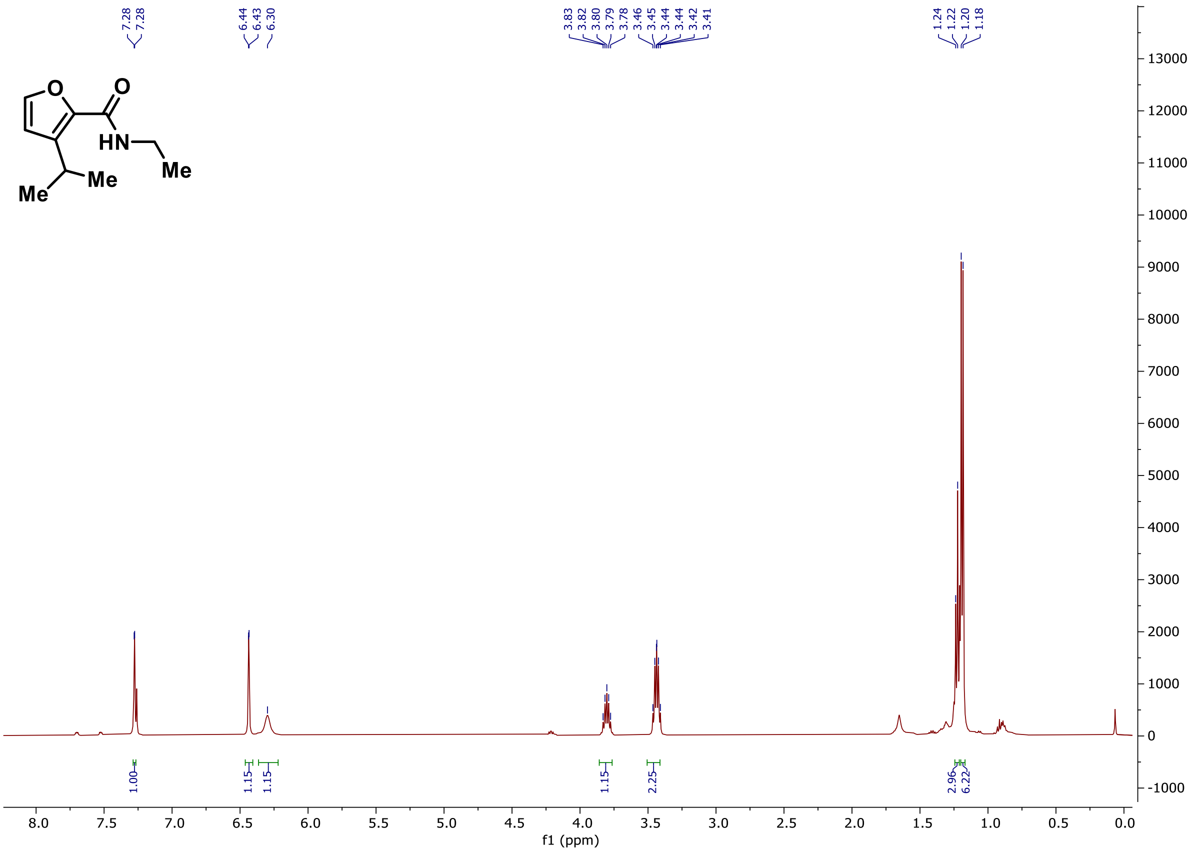
7.28
7.28
6.44
6.43
6.30
3.83
3.82
3.80
3.79
3.78
3.46
3.45
3.44
3.44
3.42
3.41
1.24
1.22
1.20
1.18
O
O
HN
Me
Me
Me
1.00
1.15
1.15
1.15
2.25
2.96
6.22
f1 (ppm)

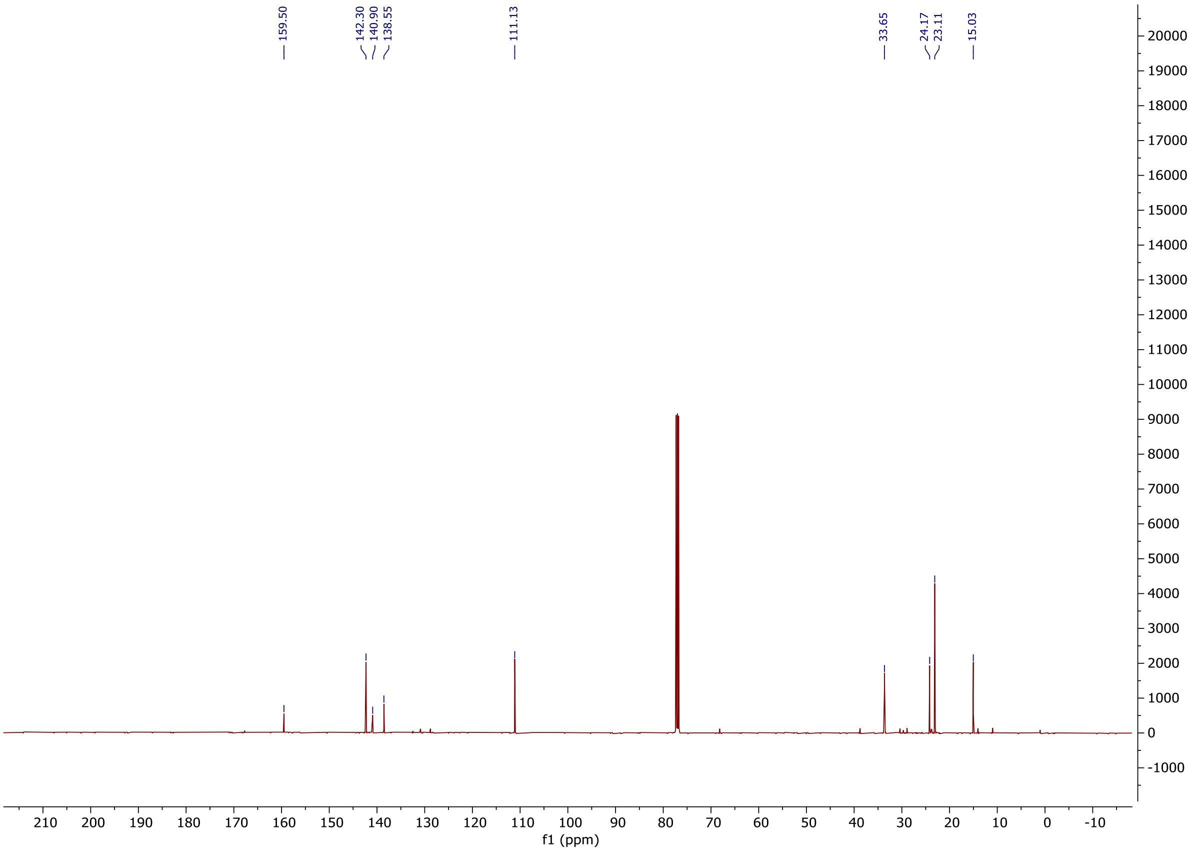
159.50
142.30
140.90
138.55
111.13
33.65
24.17
23.11
15.03
f1 (ppm)

4d

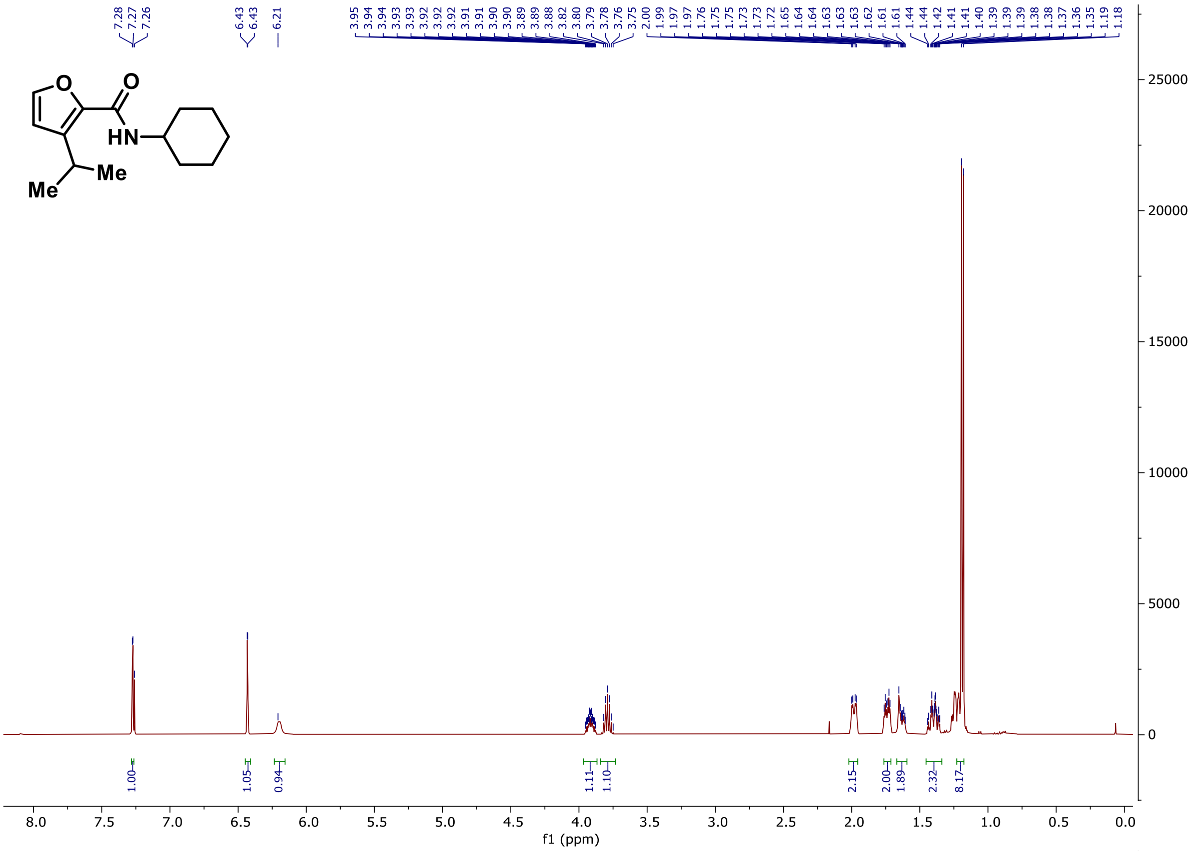

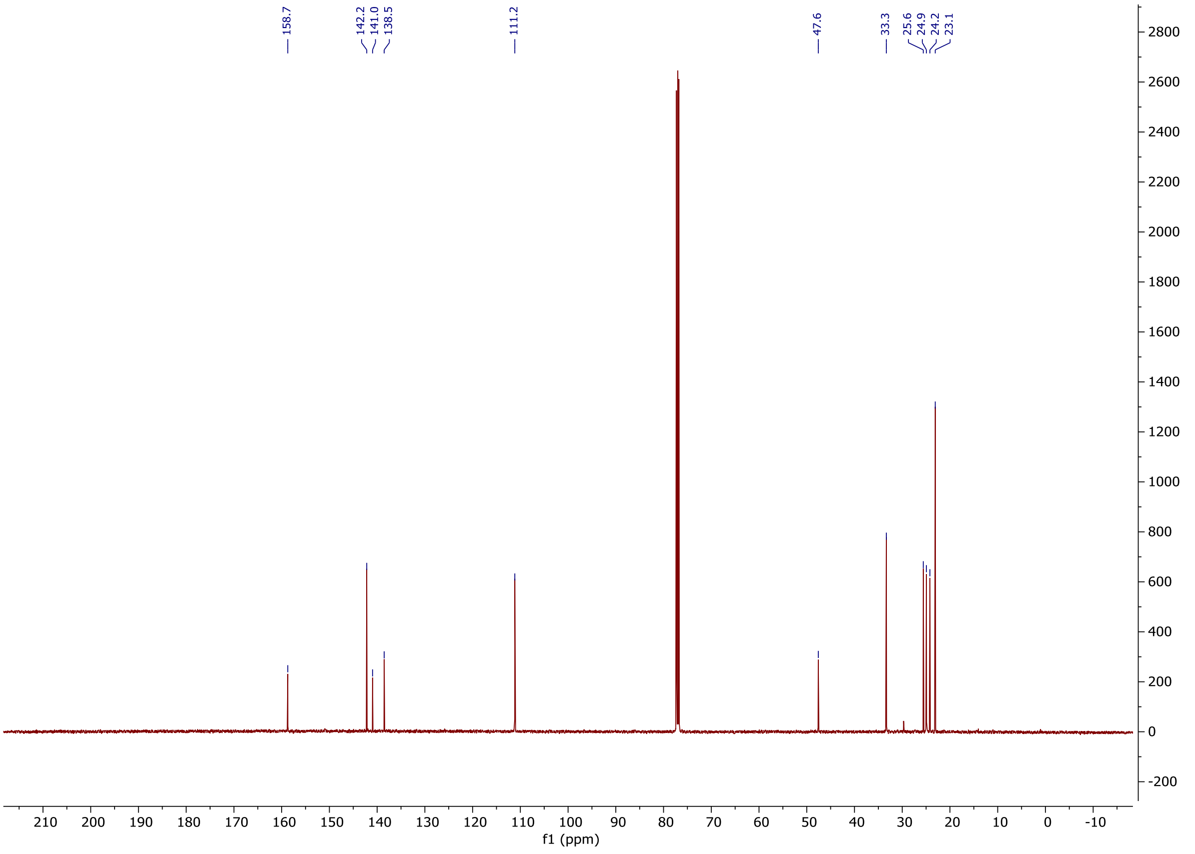

4e'

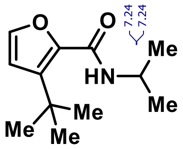

O
O
HN
Me
Me
Me
Me
Me

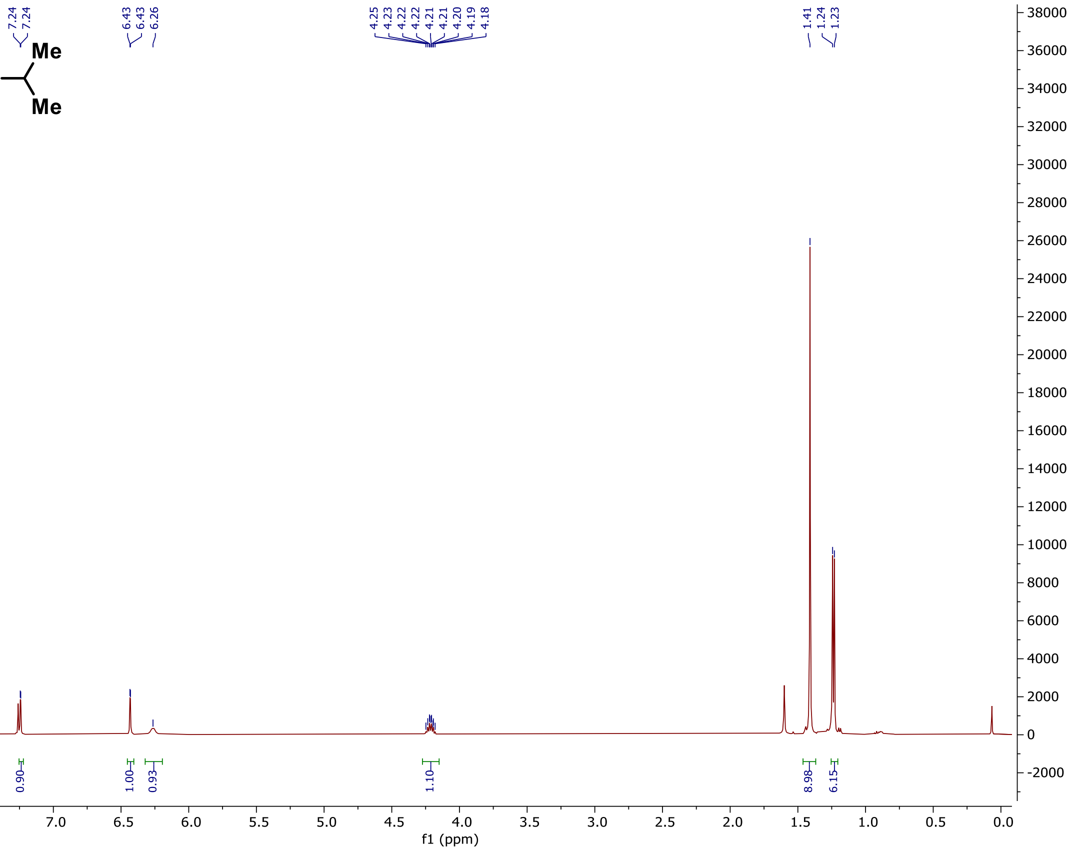

7.24
7.24
6.43
6.43
6.26
4.25
4.23
4.22
4.22
4.21
4.21
4.20
4.19
4.18
1.41
1.24
1.23
0.90
1.00
0.93
1.10
8.98
6.15
f1 (ppm)

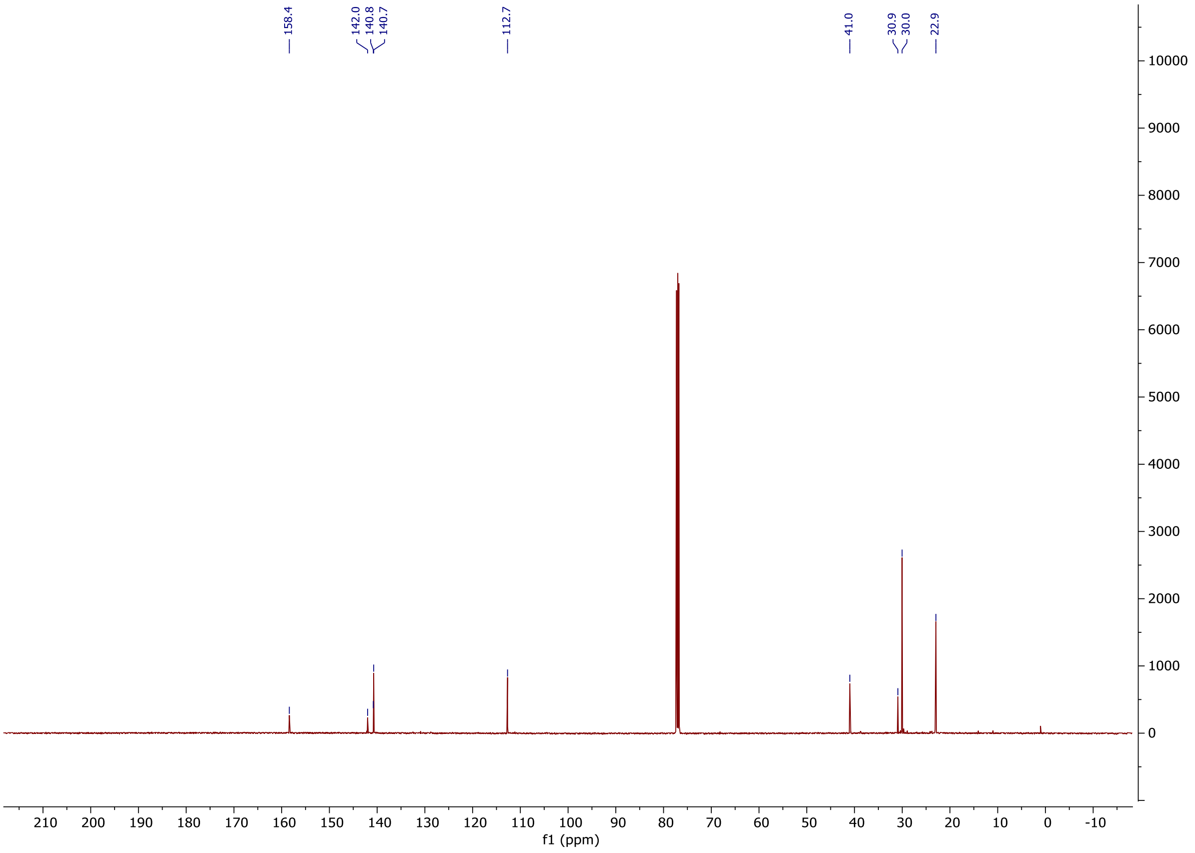

158.4
142.0
140.8
140.7
112.7
41.0
30.9
30.0
22.9
f1 (ppm)

4f

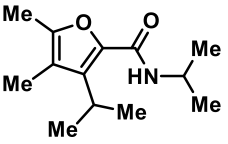
Me
O
O
Me
HN
Me
Me
Me
Me

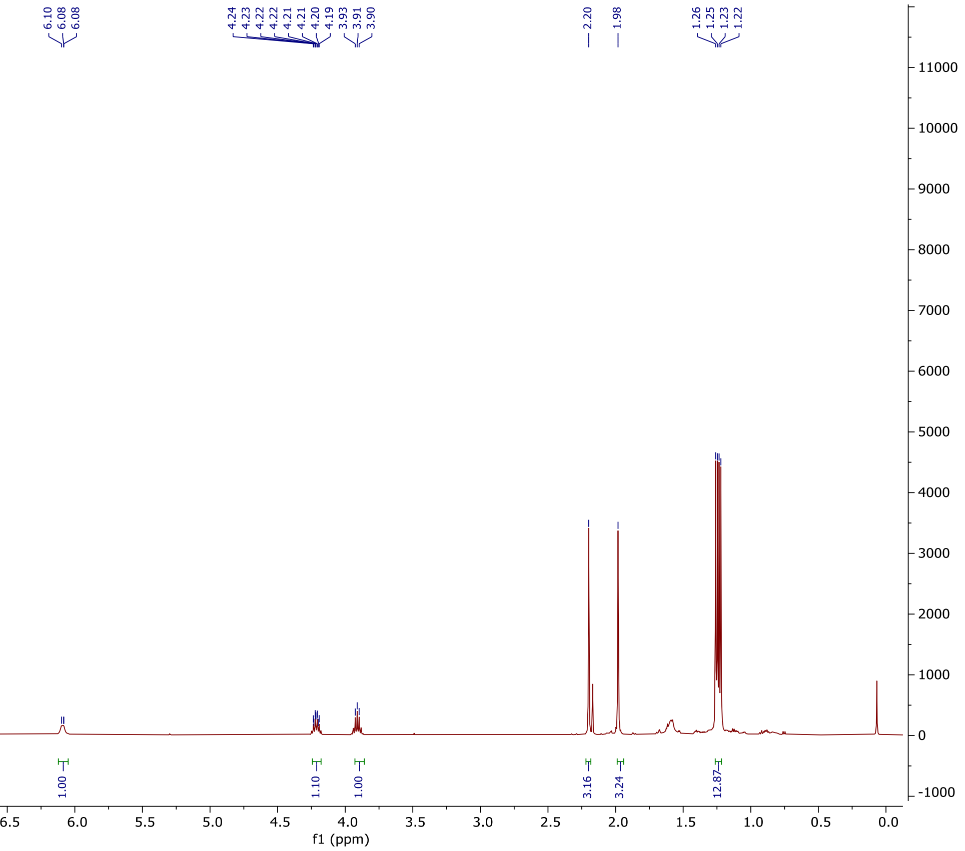
6.10
6.08
6.08
4.24
4.23
4.22
4.22
4.21
4.21
4.20
4.19
3.93
3.91
3.90
2.20
1.98
1.26
1.25
1.23
1.22
1.00
1.10
1.00
3.16
3.24
12.87
f1 (ppm)

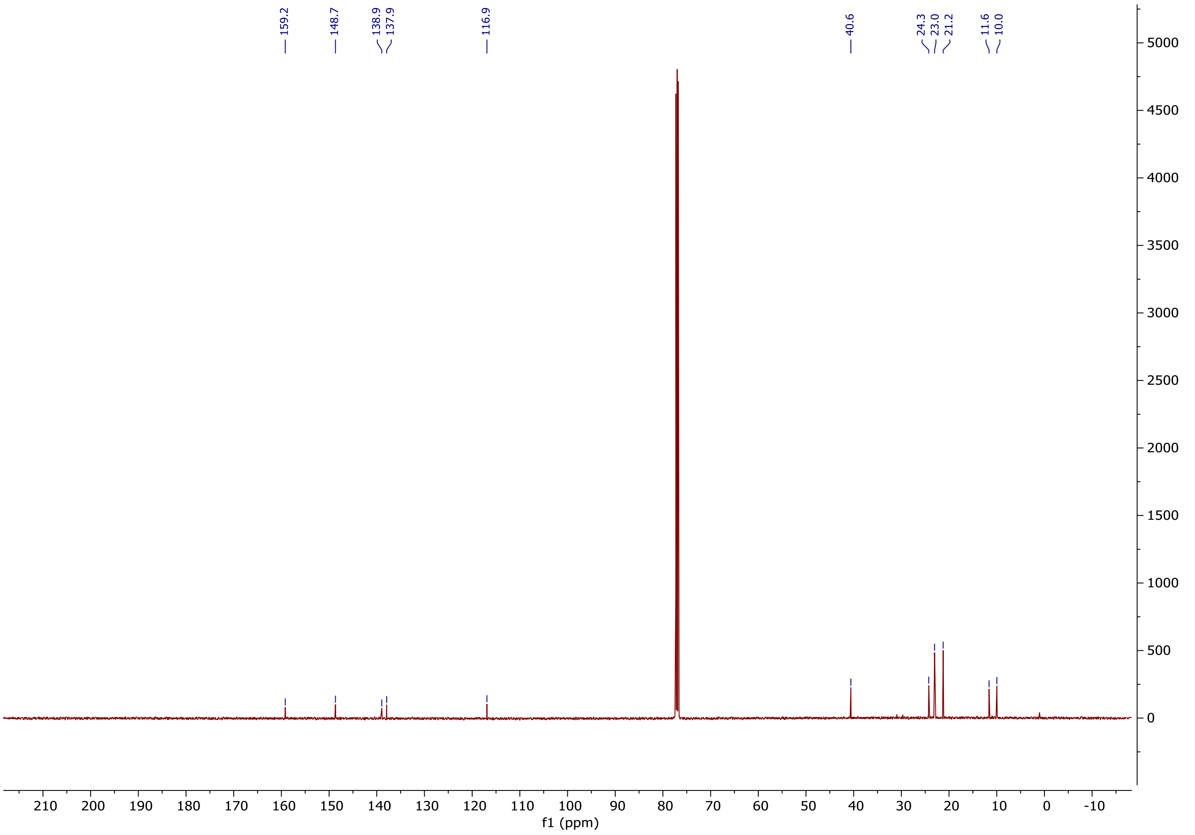
159.2
148.7
138.9
137.9
116.9
40.6
24.3
23.0
21.2
11.6
10.0
f1 (ppm)

4g

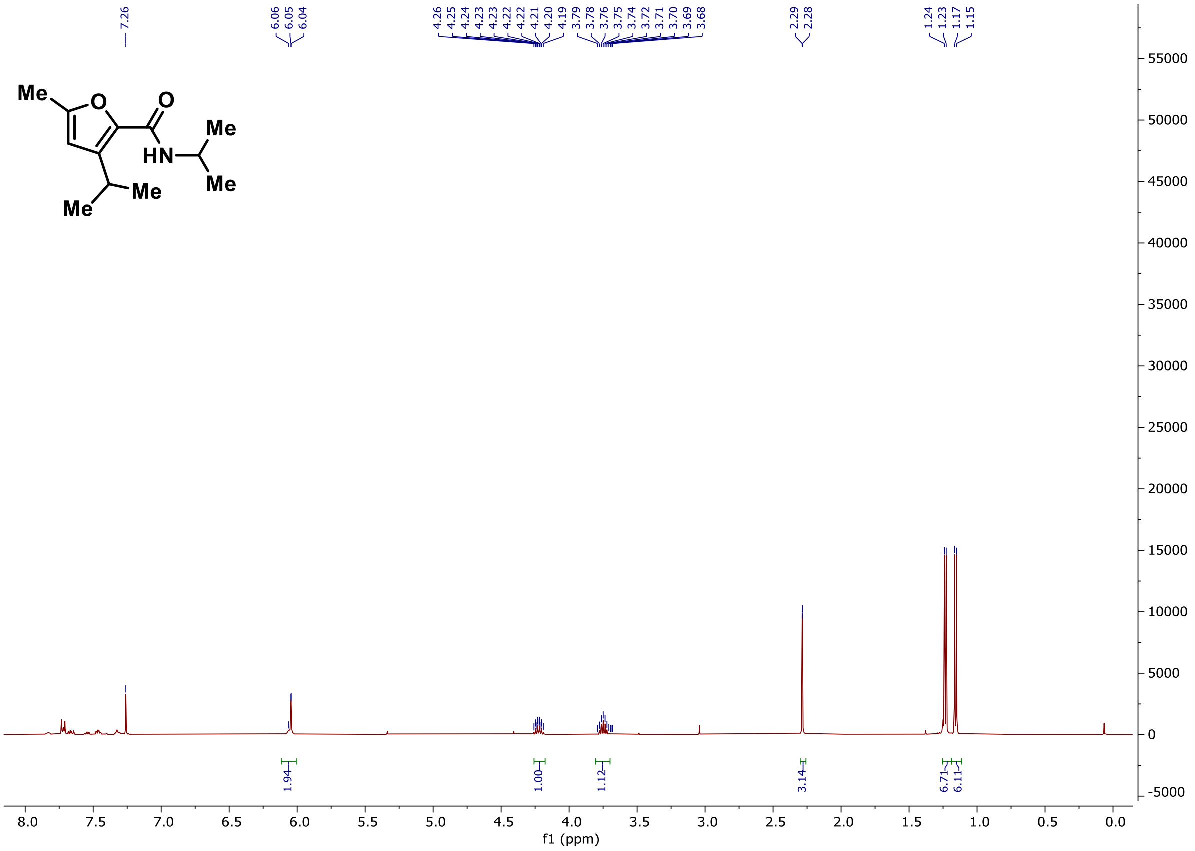
7.26
6.06 6.05 6.04
4.26 4.25 4.24 4.23 4.23 4.22 4.22 4.21 4.20 4.19 3.79 3.78 3.76 3.75 3.74 3.72 3.71 3.70 3.69 3.68
2.29 2.28
1.24 1.23 1.17 1.15
Me
O
O
Me
HN
Me
Me
Me
1.94
1.00
1.12
3.14
6.71
6.11
f1 (ppm)

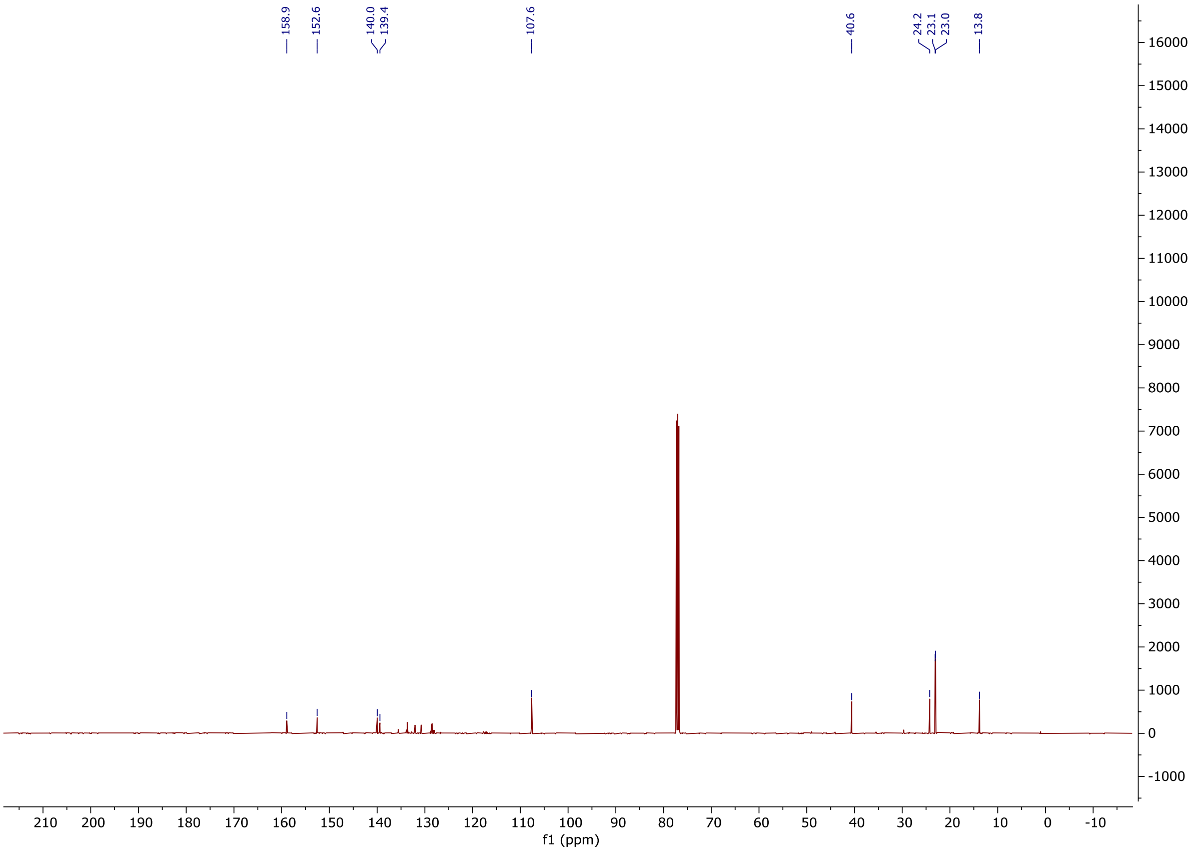
158.9
152.6
140.0 139.4
107.6
40.6
24.2 23.1 23.0
13.8
f1 (ppm)

4h

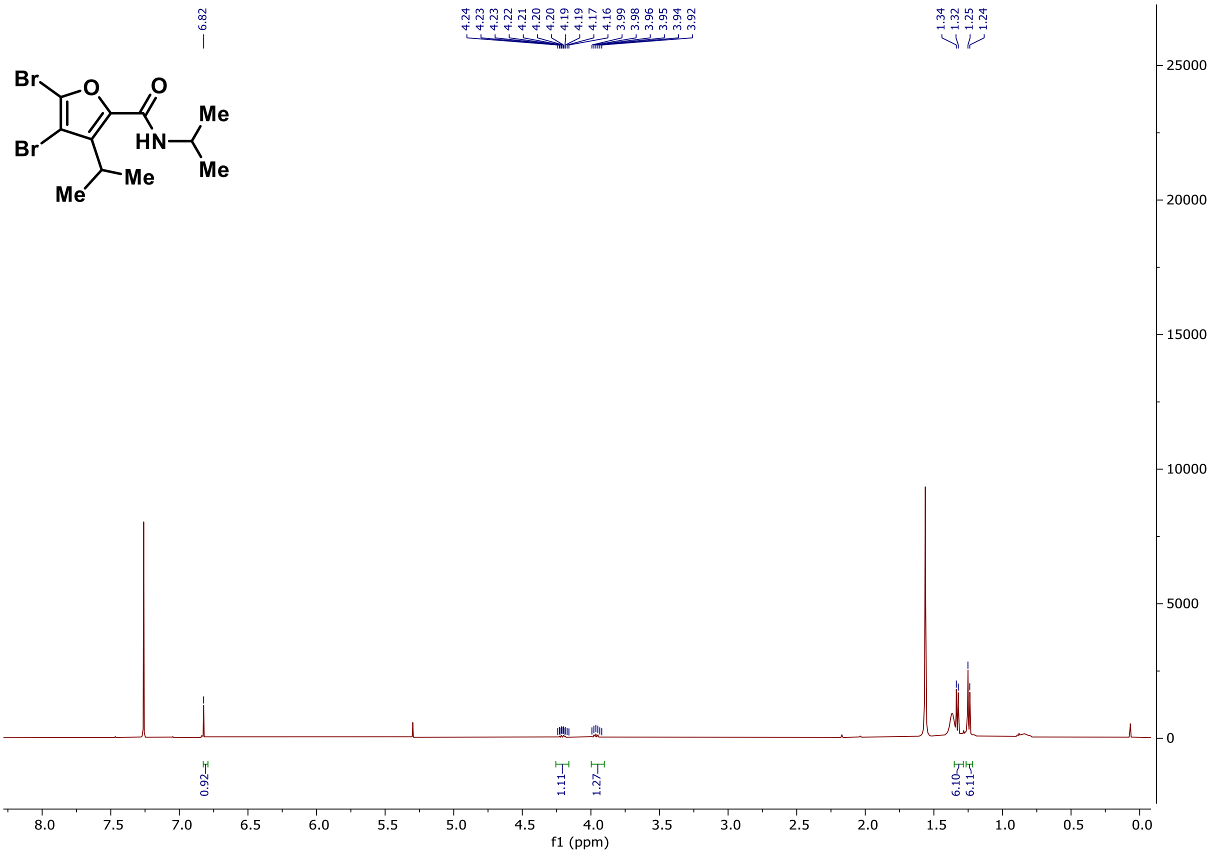
6.82
4.24
4.23
4.23
4.22
4.21
4.20
4.20
4.19
4.19
4.17
4.16
3.99
3.98
3.96
3.95
3.94
3.92
1.34
1.32
1.25
1.24
0.92
1.11
1.27
6.10
6.11
f1 (ppm)

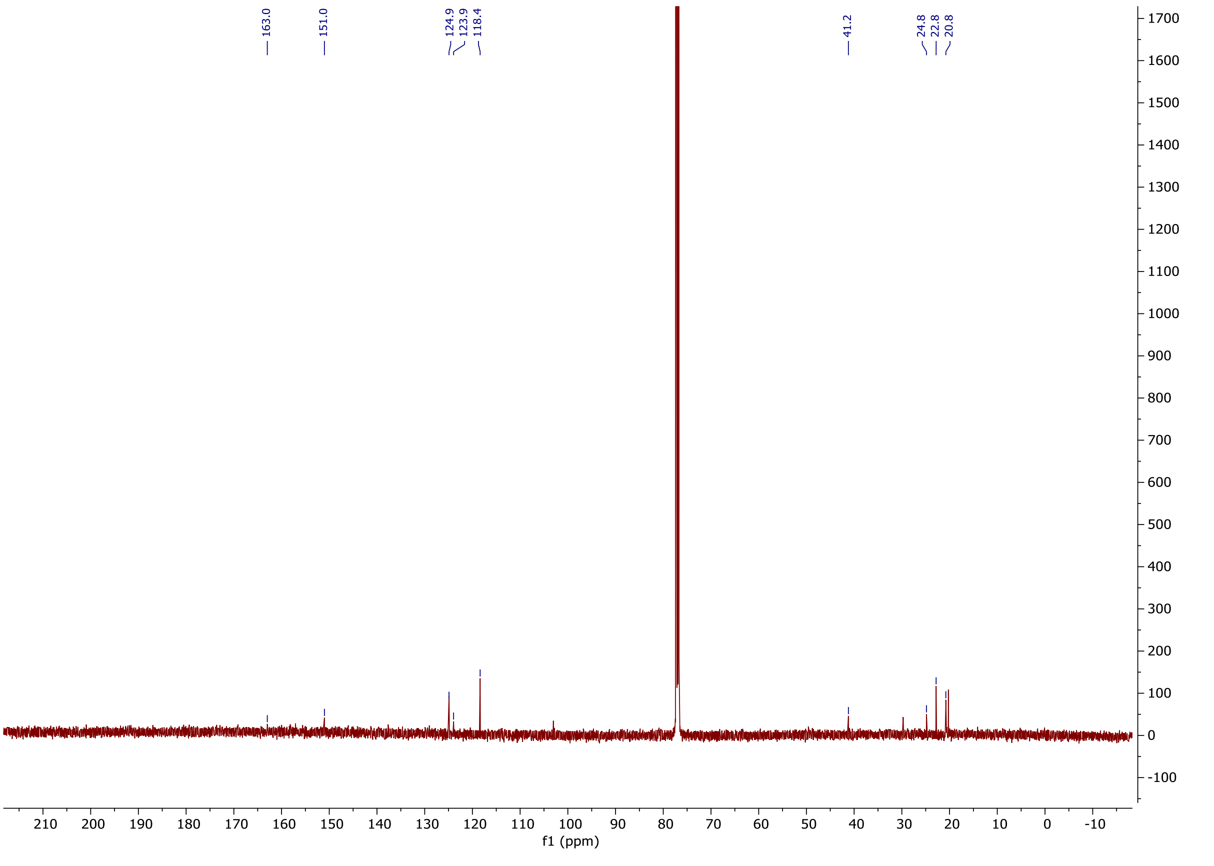
163.0
151.0
124.9
123.9
118.4
41.2
24.8
22.8
20.8
f1 (ppm)

4i

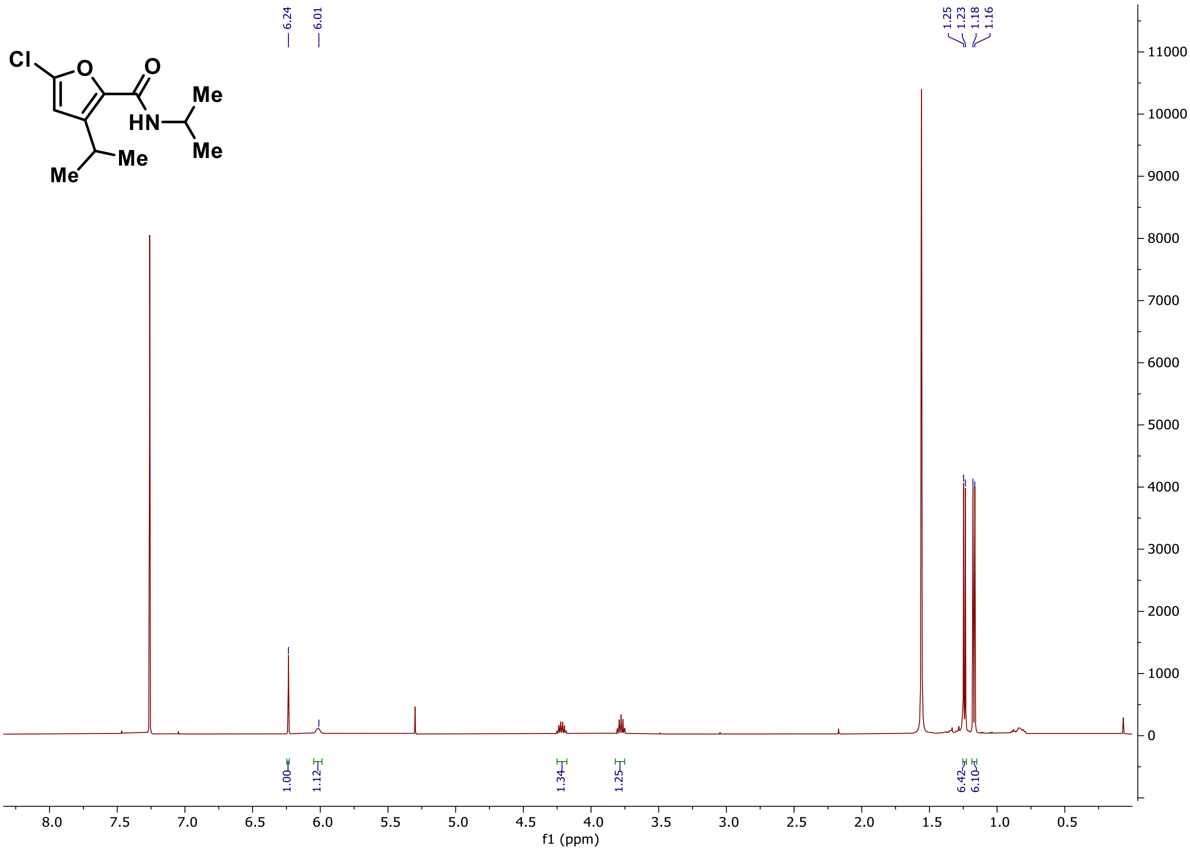
Cl
O
O
Me
HN
Me
Me
Me
6.24
6.01
1.25
1.23
1.18
1.16
1.00
1.12
1.34
1.25
6.42
6.10
f1 (ppm)

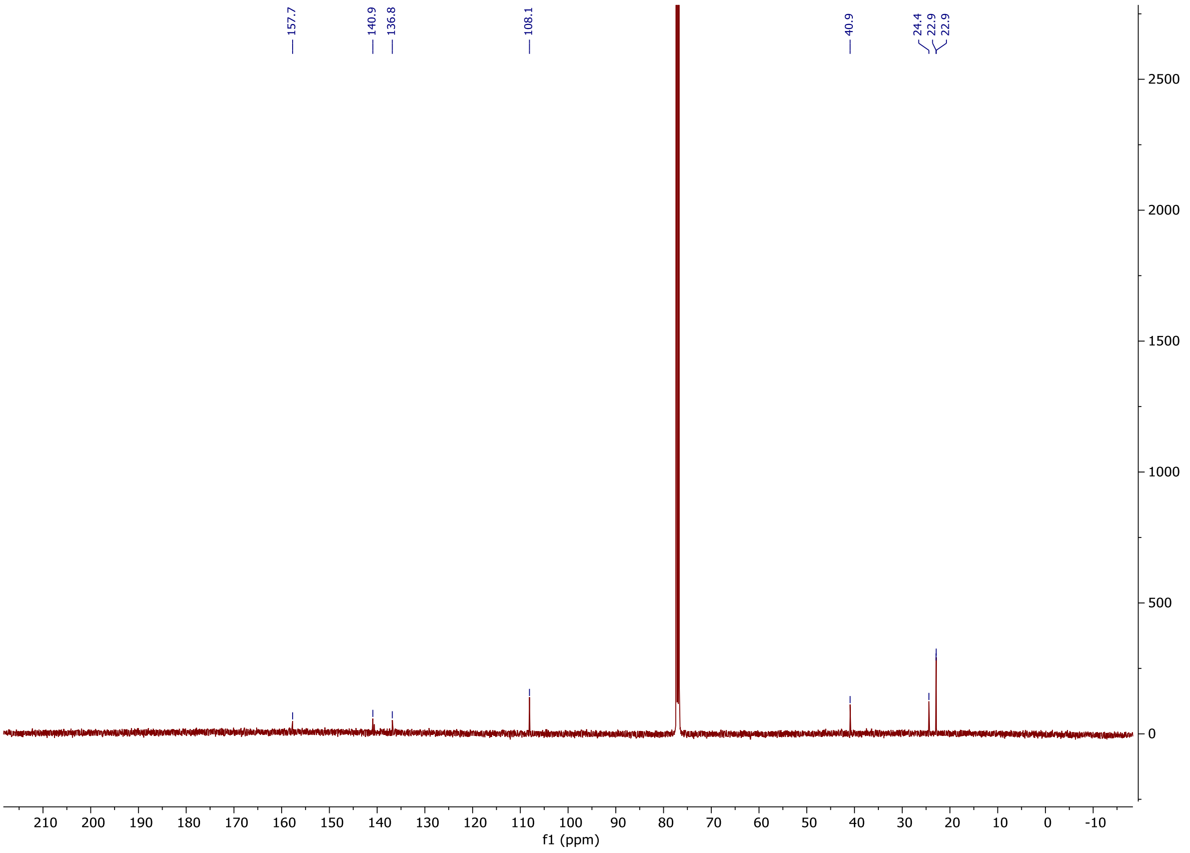
157.7
140.9
136.8
108.1
40.9
24.4
22.9
22.9
f1 (ppm)

4j

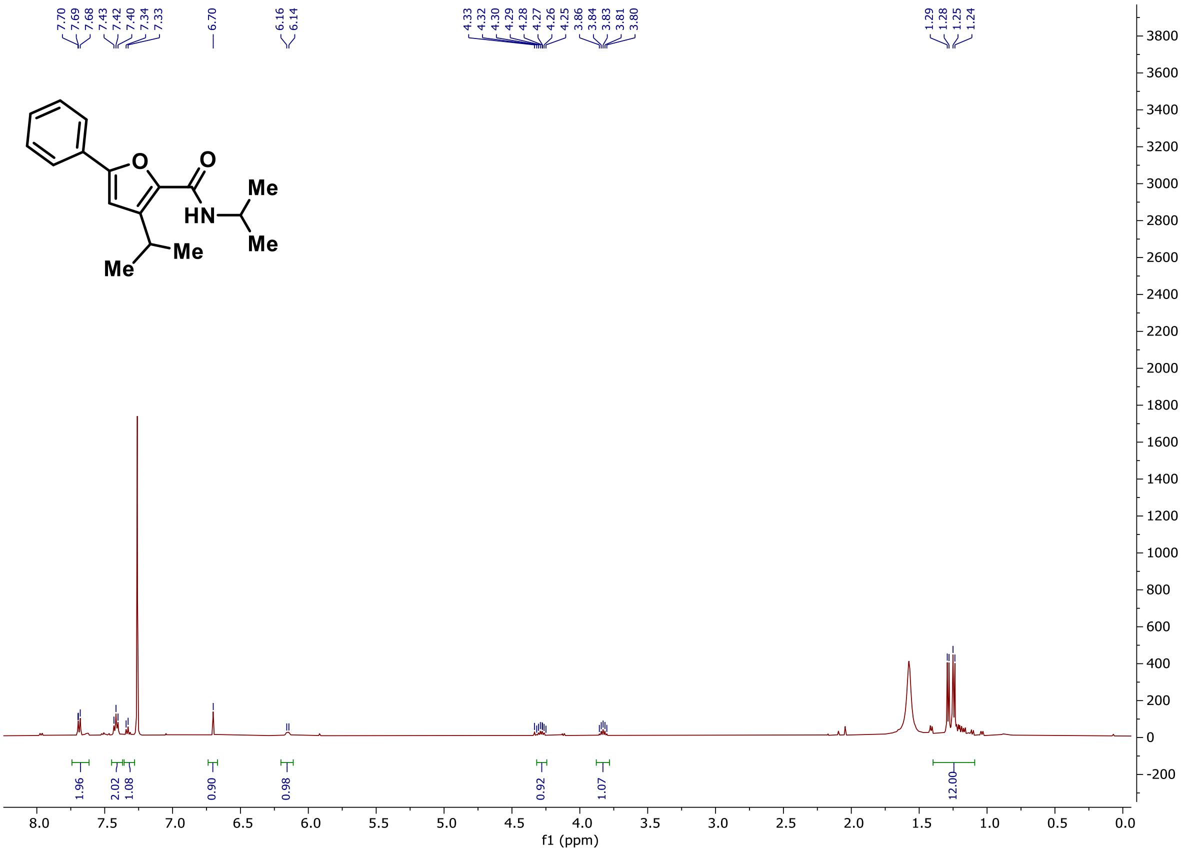
7.70 7.69 7.68 7.43 7.42 7.40 7.34 7.33
6.70
6.16 6.14
4.33 4.32 4.30 4.29 4.28 4.27 4.26 4.25 3.86 3.84 3.83 3.81 3.80
1.29 1.28 1.25 1.24
O
Me
HN
Me
Me
Me
1.96
2.02
1.08
0.90
0.98
0.92
1.07
12.00
f1 (ppm)

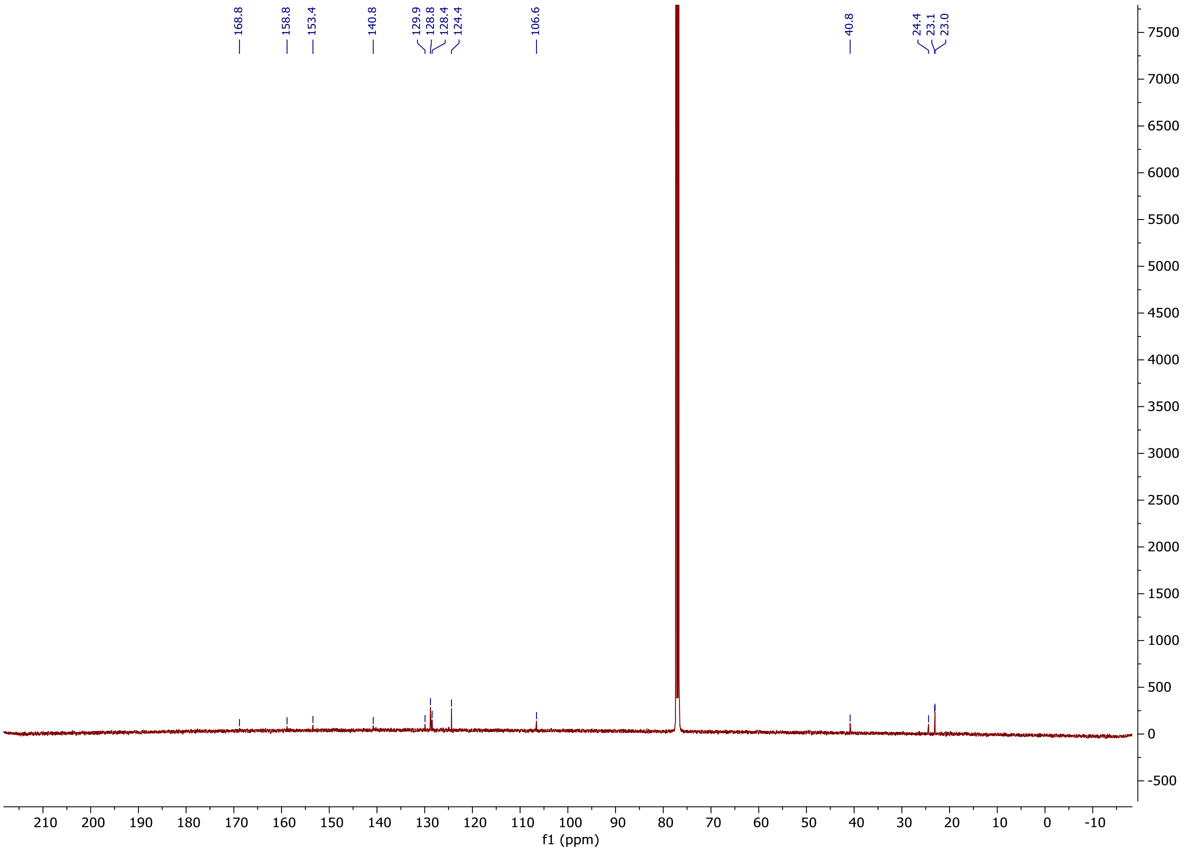
168.8
158.8
153.4
140.8
129.9 128.8 128.4 124.4
106.6
40.8
24.4 23.1 23.0
f1 (ppm)

4k

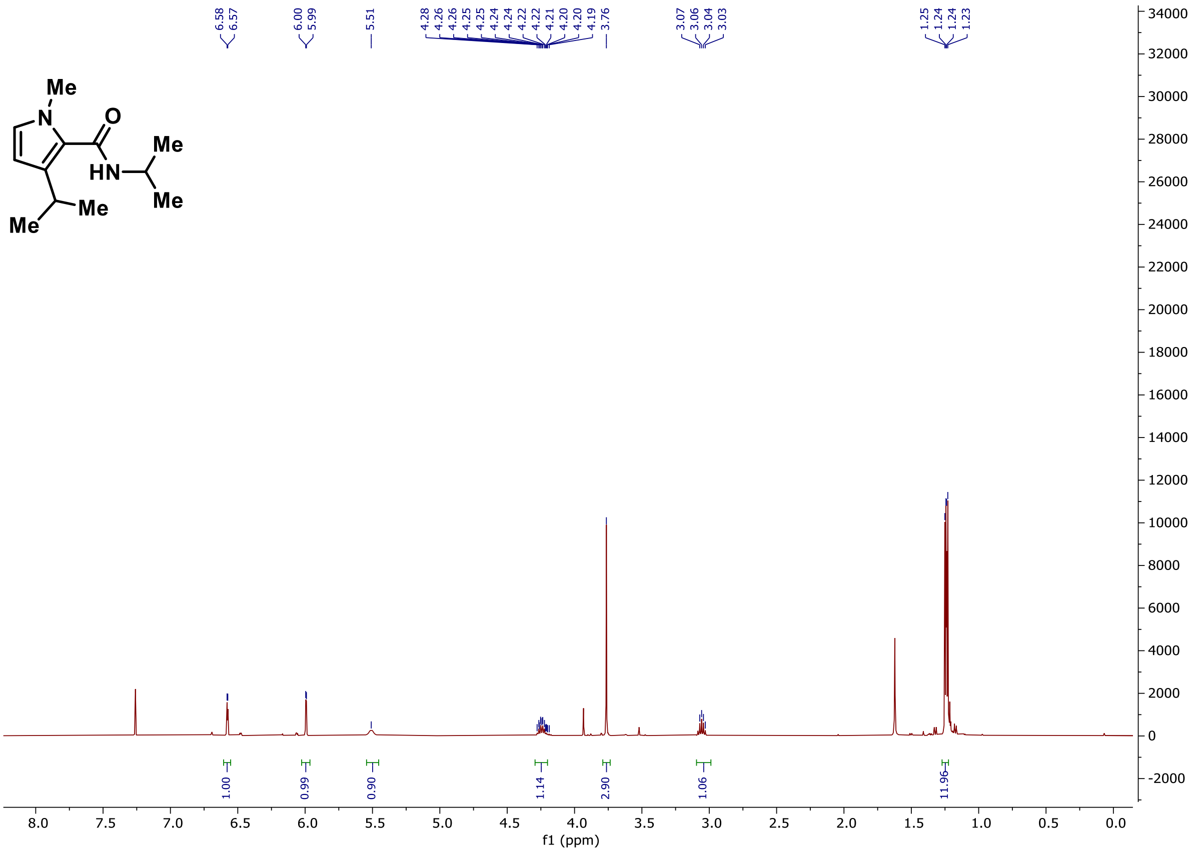
Me
N
O
HN
Me
Me
Me
Me
6.58
6.57
6.00
5.99
5.51
4.28
4.26
4.26
4.25
4.25
4.24
4.24
4.22
4.22
4.21
4.20
4.20
4.19
3.76
3.07
3.06
3.04
3.03
1.25
1.24
1.24
1.23
1.00
0.99
0.90
1.14
2.90
1.06
11.96
f1 (ppm)

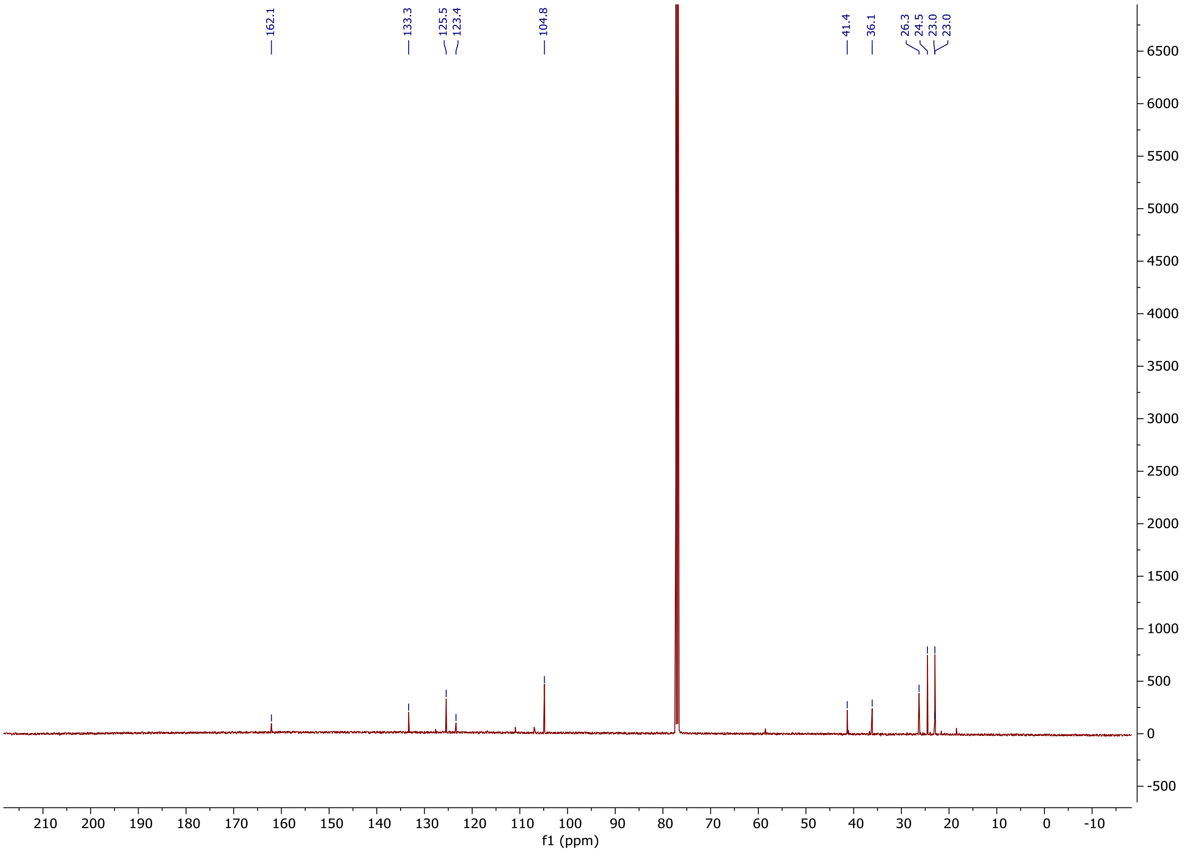
162.1
133.3
125.5
123.4
104.8
41.4
36.1
26.3
24.5
23.0
23.0
f1 (ppm)

4l

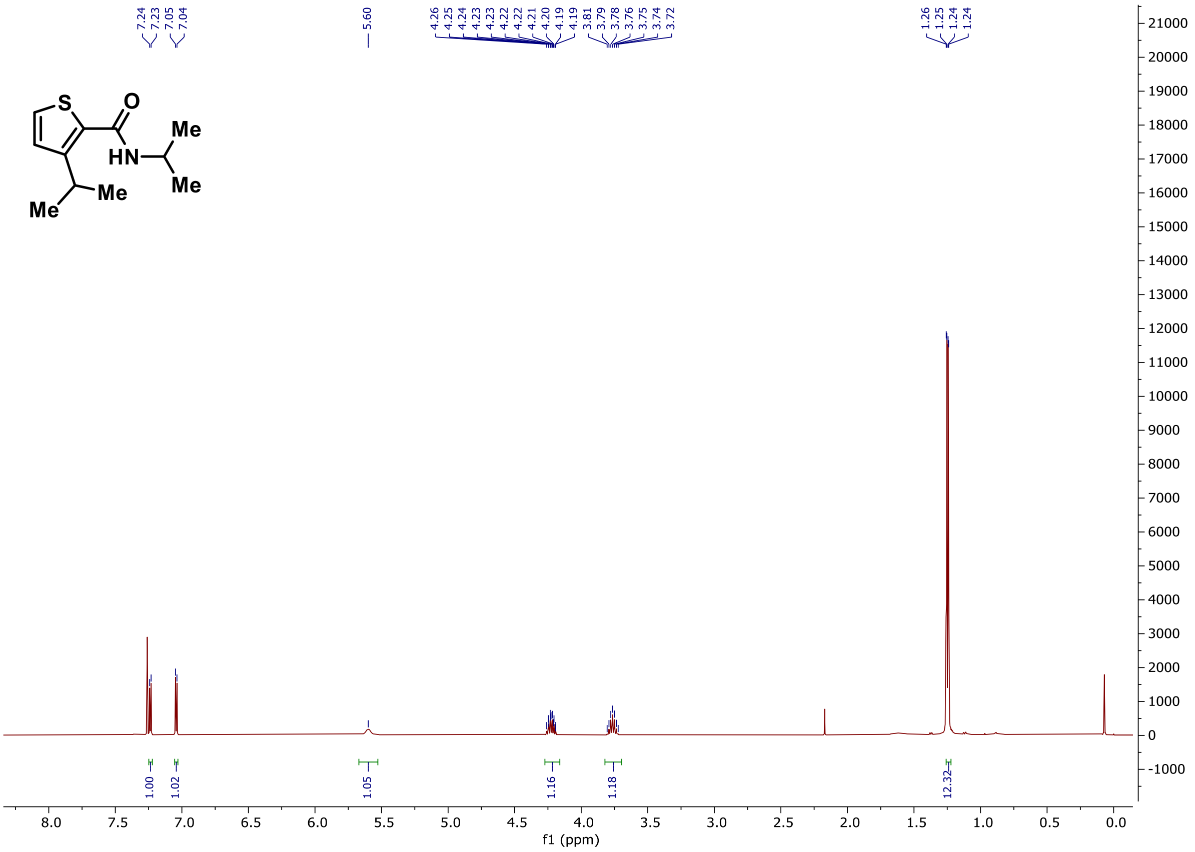

7.24
7.23
7.05
7.04
5.60
4.26
4.25
4.24
4.23
4.23
4.22
4.22
4.21
4.20
4.19
4.19
3.81
3.79
3.78
3.76
3.75
3.74
3.72
1.26
1.25
1.24
1.24
S
O
Me
HN
Me
Me
Me
1.00
1.02
1.05
1.16
1.18
12.32
f1 (ppm)

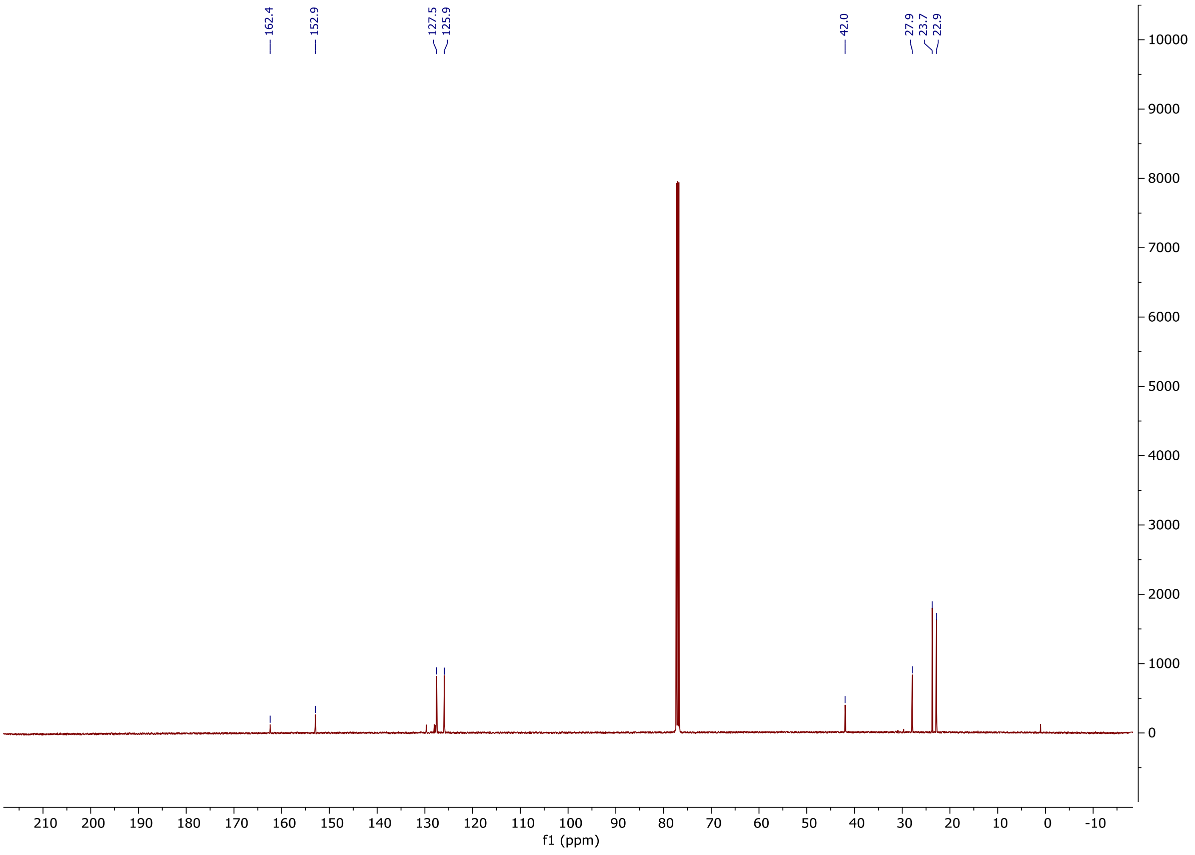

162.4
152.9
127.5
125.9
42.0
27.9
23.7
22.9
f1 (ppm)

4m

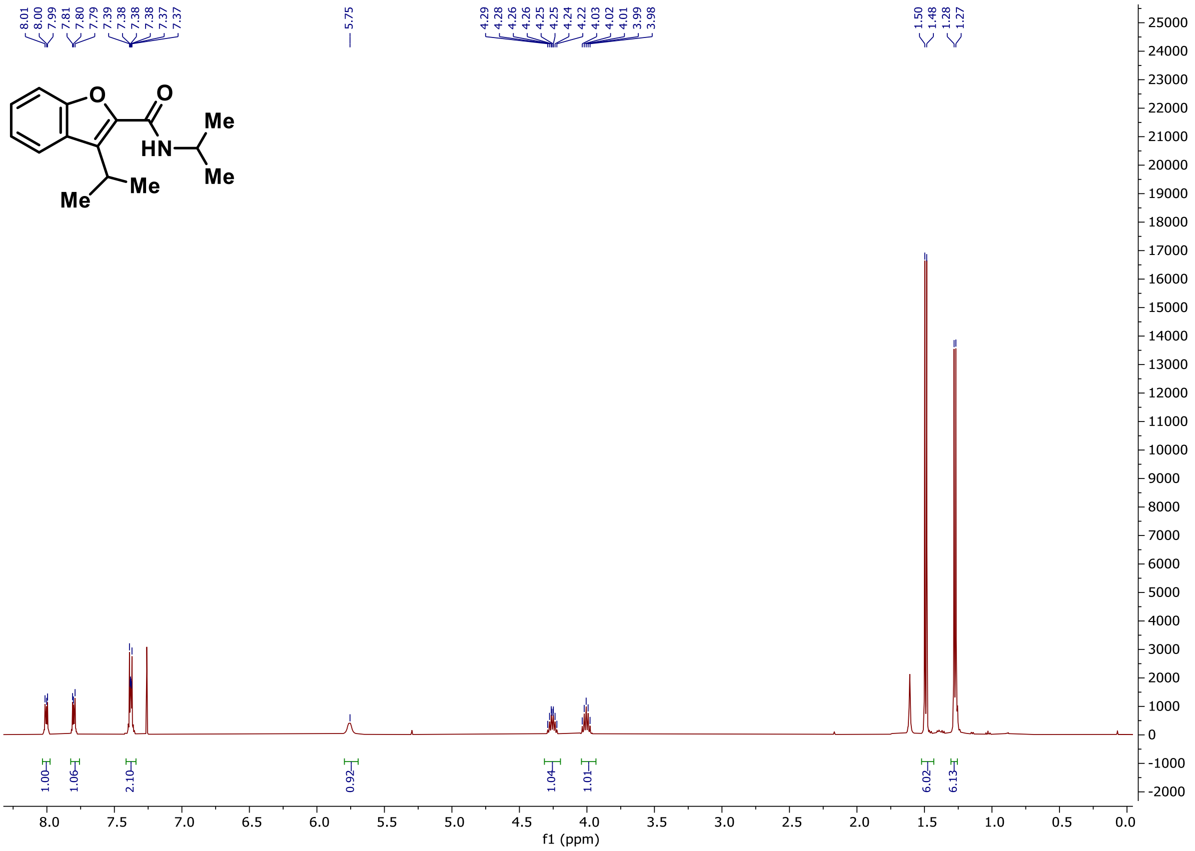
8.01
8.00
7.99
7.81
7.80
7.79
7.39
7.38
7.38
7.38
7.37
7.37
5.75
4.29
4.28
4.26
4.26
4.25
4.25
4.24
4.22
4.03
4.02
4.01
3.99
3.98
1.50
1.48
1.28
1.27
O
O
HN
Me
Me
Me
Me
1.00
1.06
2.10
0.92
1.04
1.01
6.02
6.13
f1 (ppm)

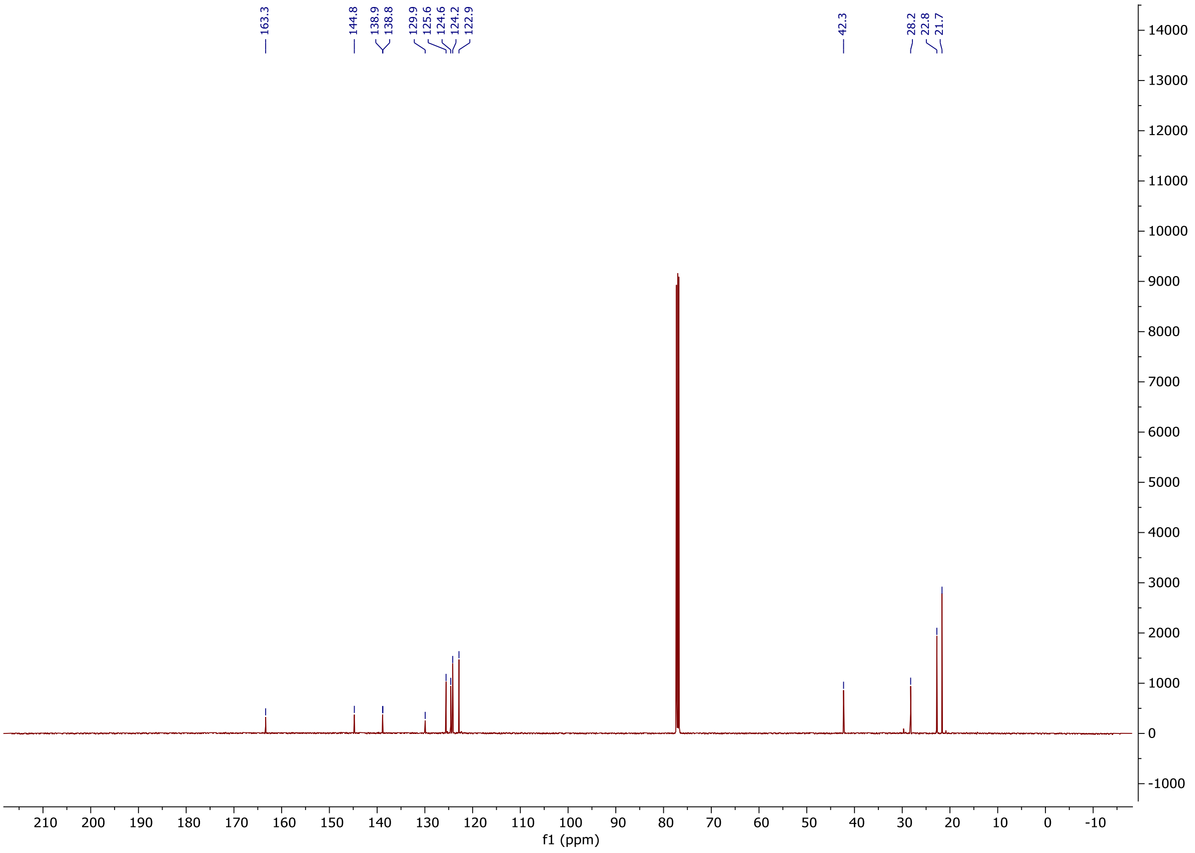
163.3
144.8
138.9
138.8
129.9
125.6
124.6
124.2
122.9
42.3
28.2
22.8
21.7
f1 (ppm)

4n

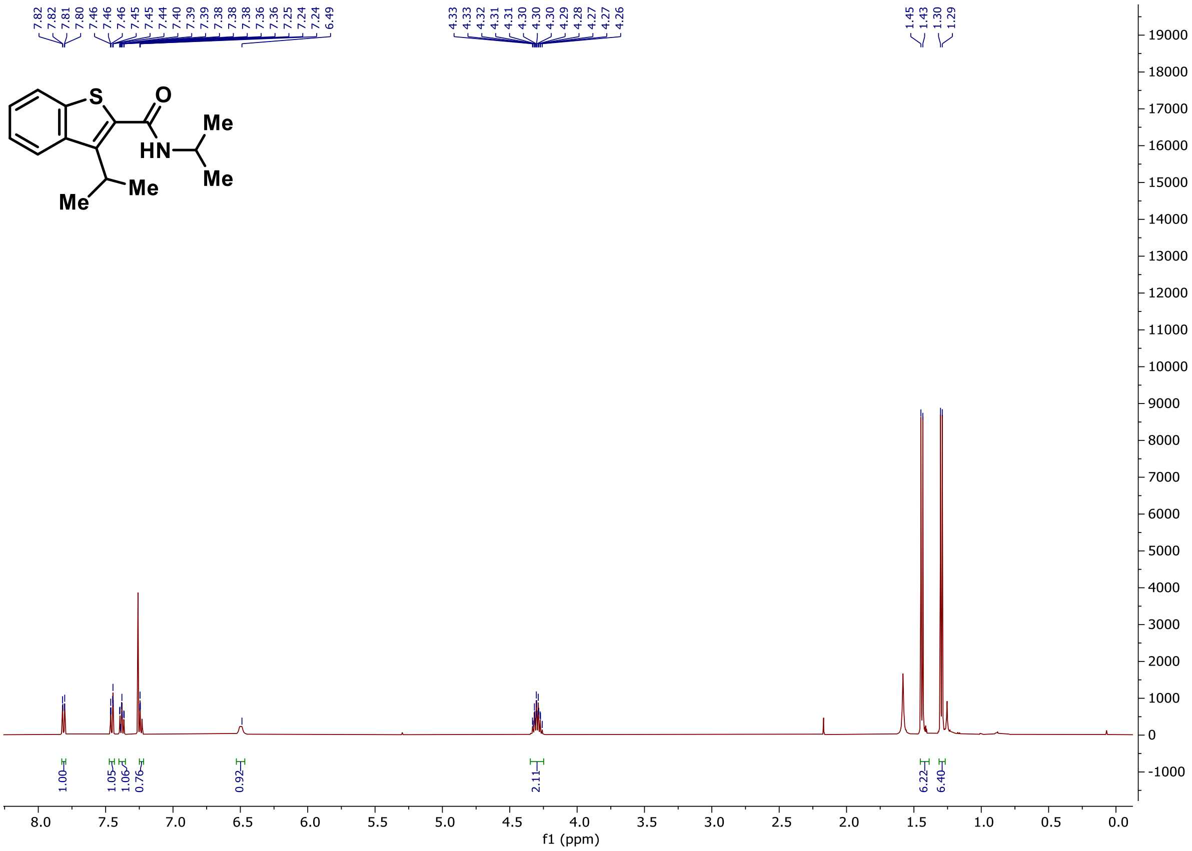
7.82
7.82
7.81
7.80
7.46
7.46
7.45
7.44
7.40
7.39
7.39
7.38
7.38
7.38
7.36
7.36
7.25
7.24
7.24
6.49
4.33
4.33
4.32
4.31
4.31
4.30
4.30
4.30
4.29
4.28
4.27
4.27
4.26
1.45
1.43
1.30
1.29
S
O
HN
Me
Me
Me
Me
1.00
1.05
1.06
0.76
0.92
2.11
6.22
6.40
f1 (ppm)

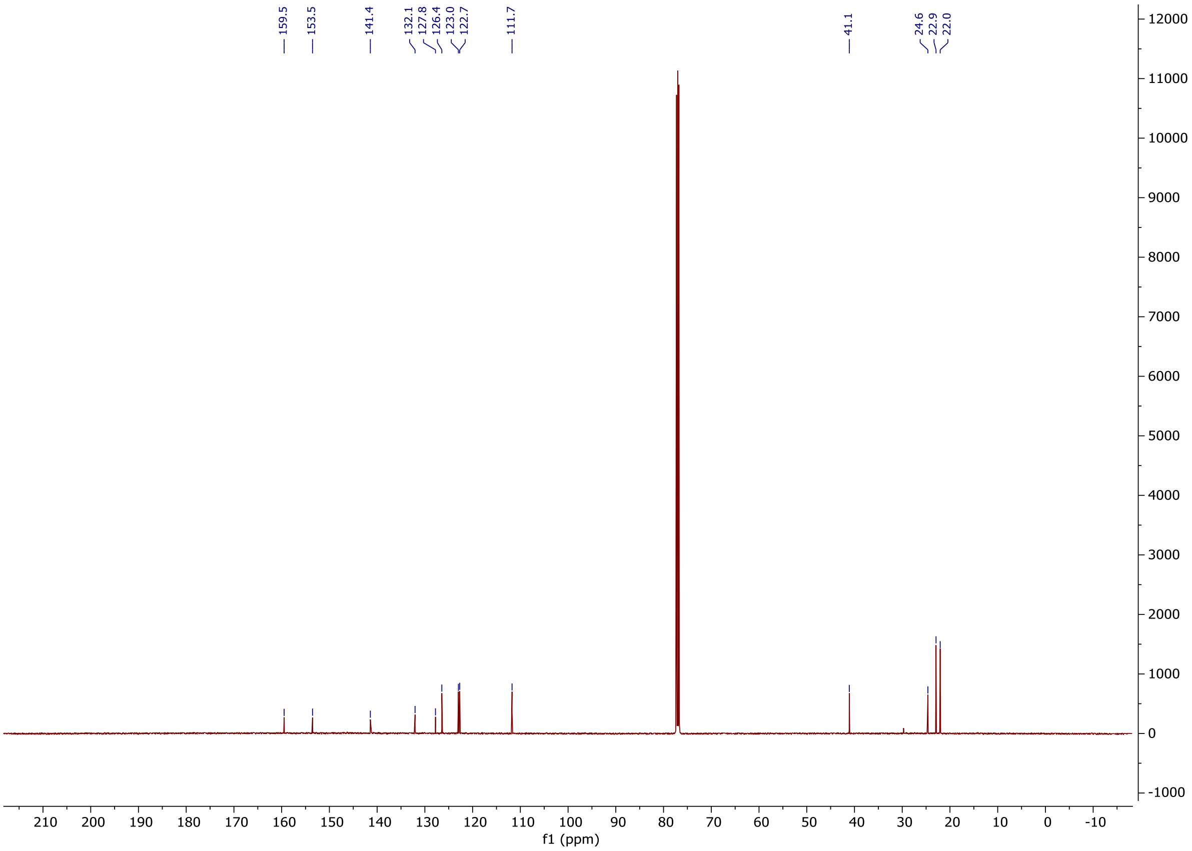
159.5
153.5
141.4
132.1
127.8
126.4
123.0
122.7
111.7
41.1
24.6
22.9
22.0
f1 (ppm)

4o

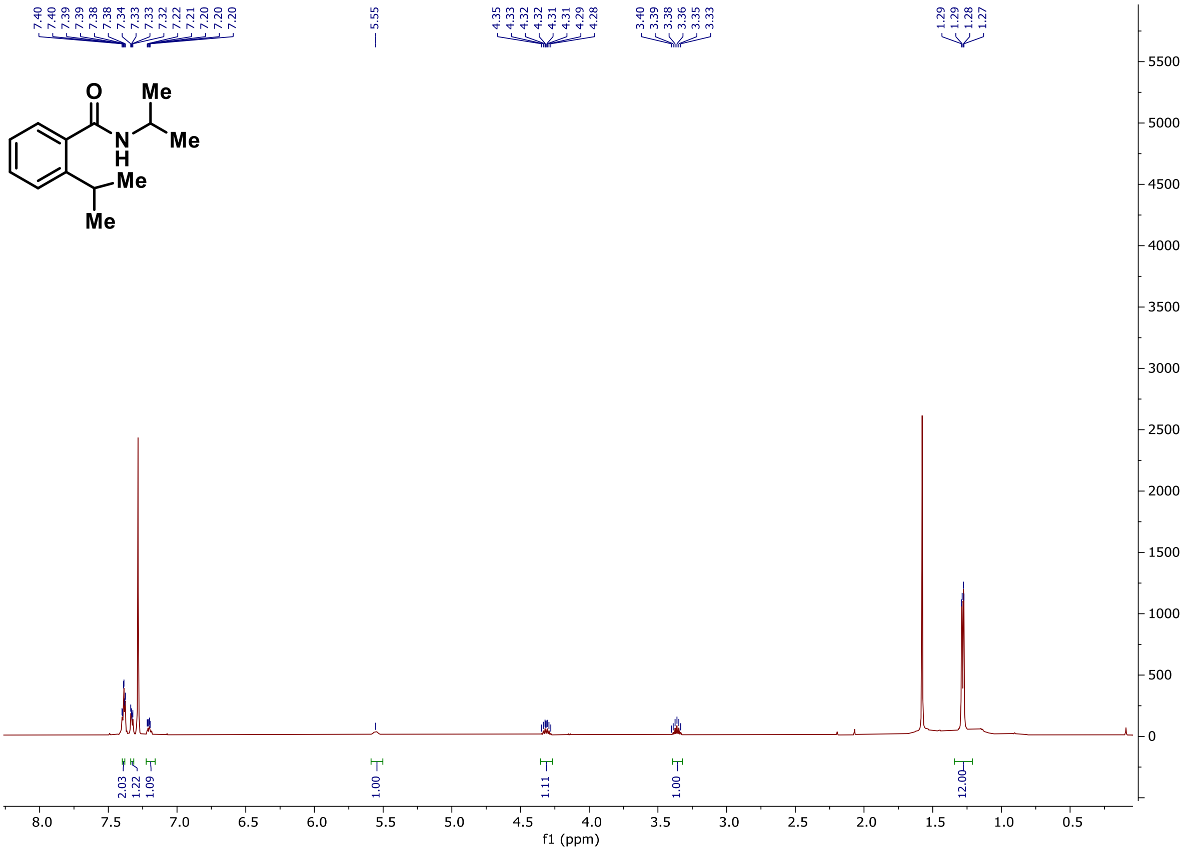
O
Me
N
H
Me
Me
Me
7.40
7.40
7.39
7.39
7.38
7.38
7.34
7.33
7.33
7.32
7.22
7.21
7.20
7.20
5.55
4.35
4.33
4.32
4.32
4.31
4.31
4.29
4.28
3.40
3.39
3.38
3.36
3.35
3.33
1.29
1.29
1.28
1.27
2.03
1.22
1.09
1.00
1.11
1.00
12.00
f1 (ppm)

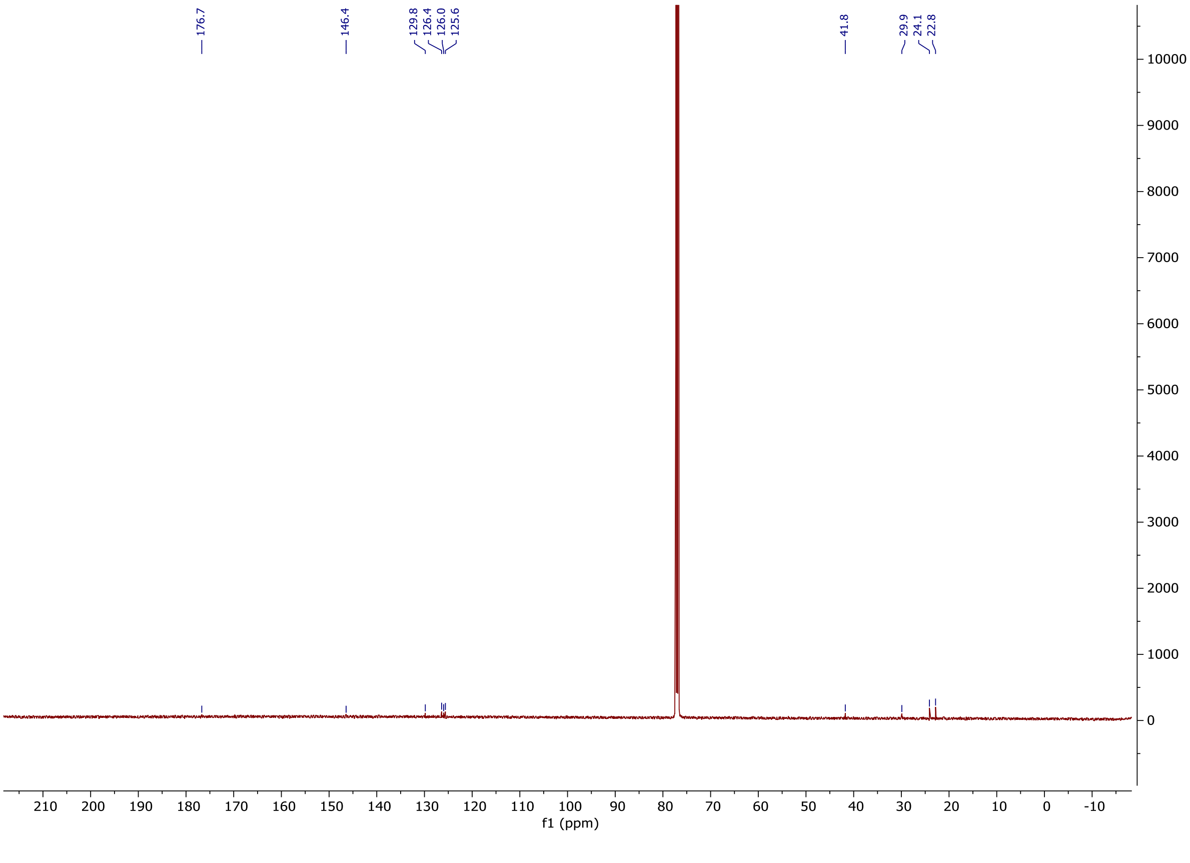
176.7
146.4
129.8
126.4
126.0
125.6
41.8
29.9
24.1
22.8
f1 (ppm)

Intramolecular N→C Alkyl Transfer Involving Other Alkyl Units: Substrates (Table 1B)

1p

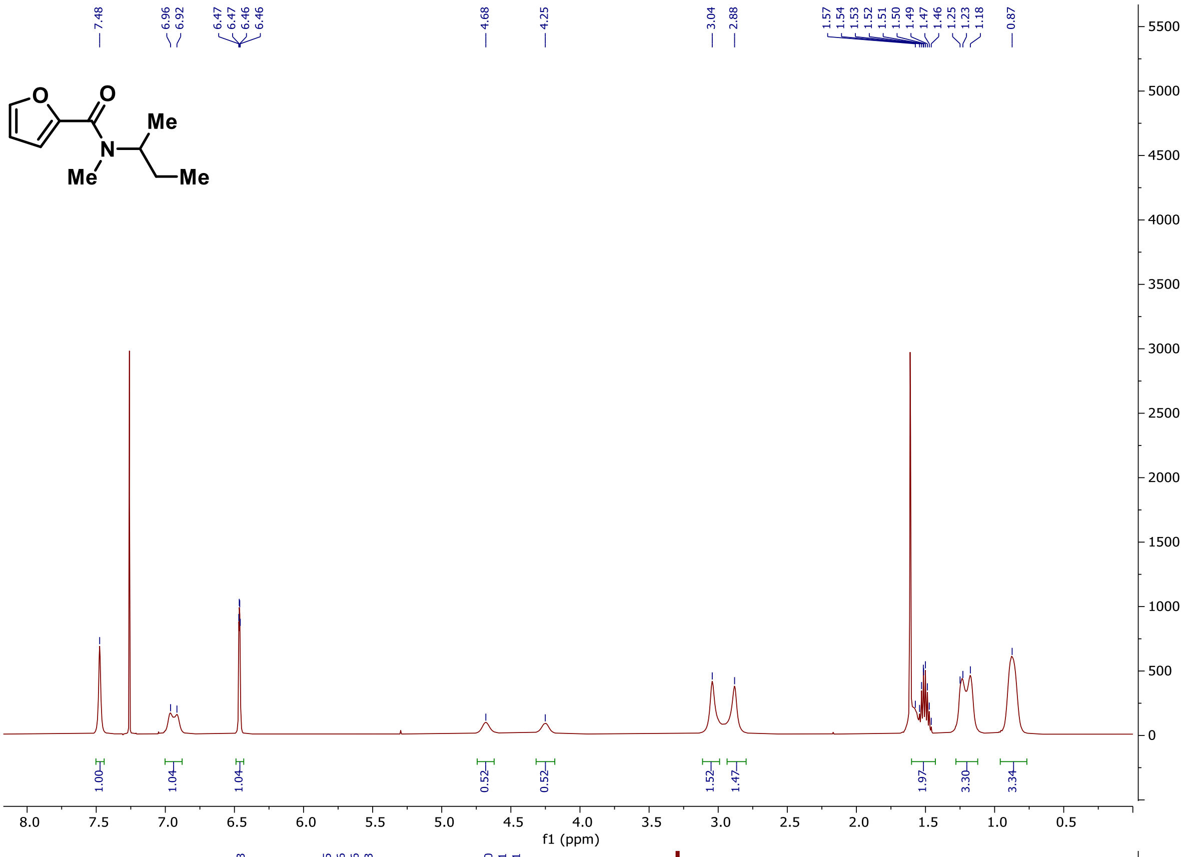

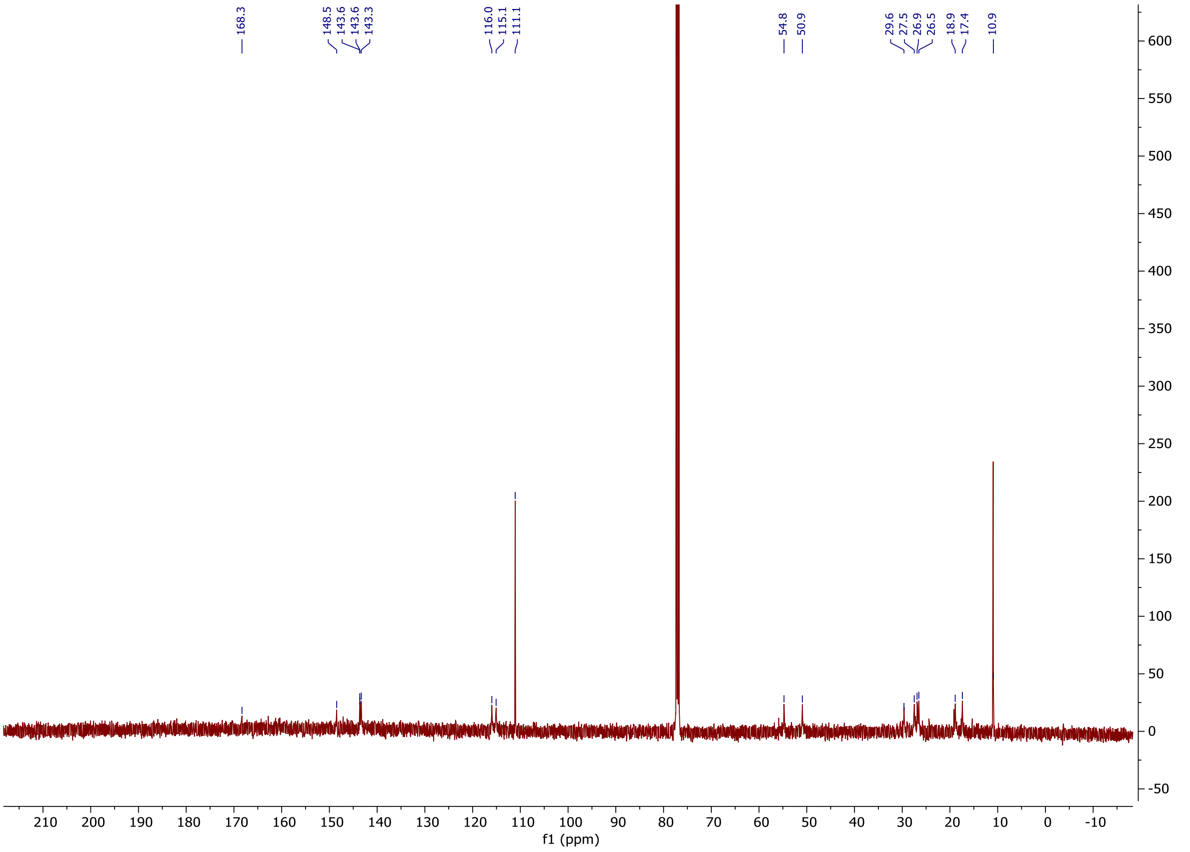

1q

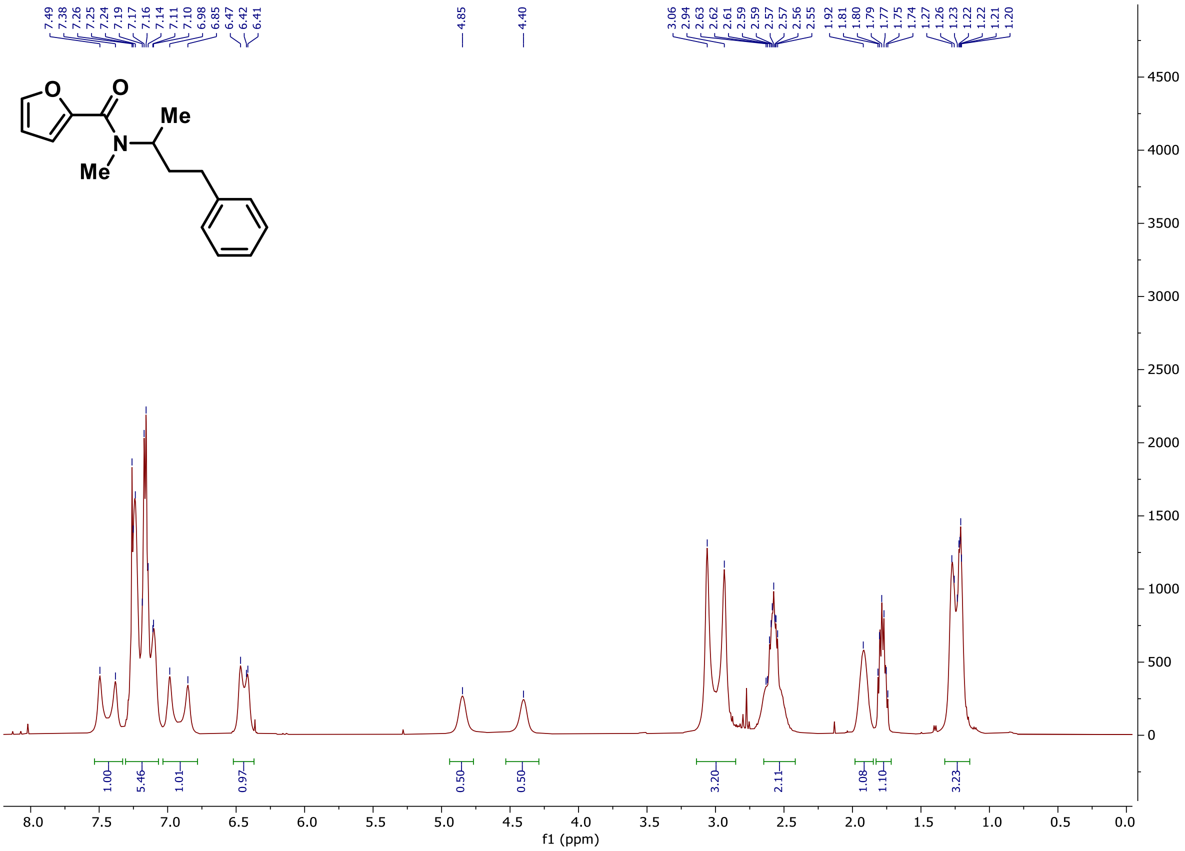
7.49 7.38 7.26 7.25 7.24 7.19 7.17 7.16 7.14 7.11 7.10 6.98 6.85 6.47 6.42 6.41
4.85
4.40
3.06 2.94 2.63 2.62 2.61 2.59 2.59 2.57 2.57 2.56 2.55 1.92 1.81 1.80 1.79 1.77 1.75 1.74 1.27 1.26 1.23 1.22 1.22 1.21 1.20
O
O
Me
N
Me
1.00 5.46 1.01 0.97 0.50 0.50 3.20 2.11 1.08 1.10 3.23
f1 (ppm)

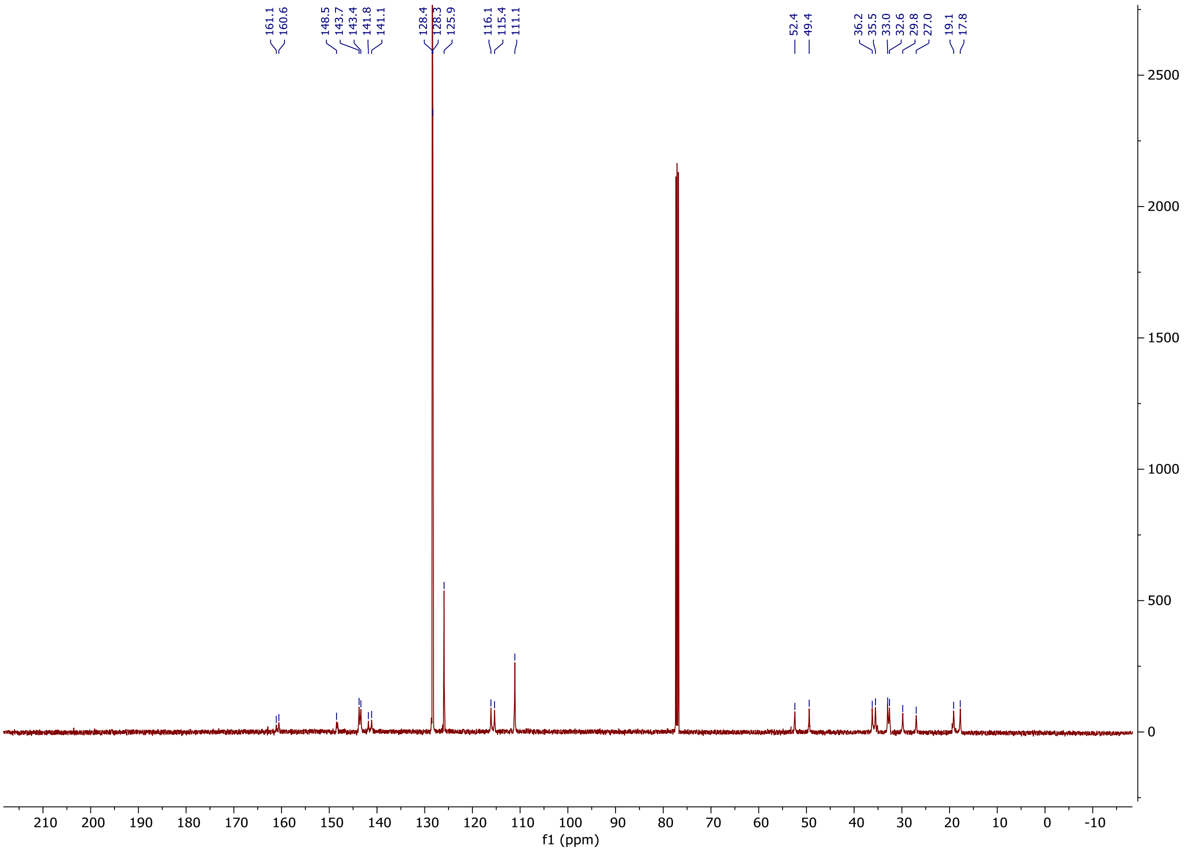
161.1 160.6
148.5 143.7 143.4 141.8 141.1
128.4 128.3 125.9
116.1 115.4 111.1
52.4 49.4
36.2 35.5 33.0 32.6 29.8 27.0
19.1 17.8
f1 (ppm)

1r

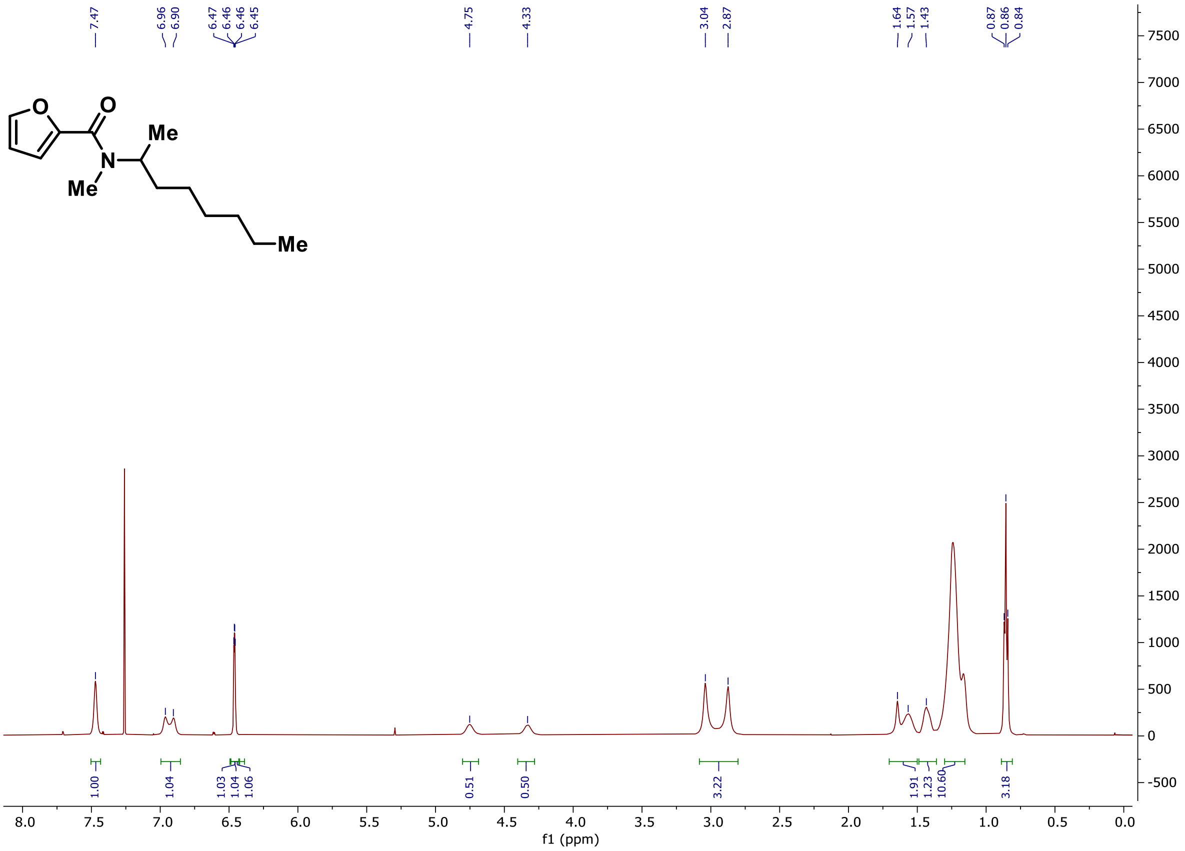
O
O
N
Me
Me
Me
7.47
6.96
6.90
6.47
6.46
6.46
6.45
4.75
4.33
3.04
2.87
1.64
1.57
1.43
0.87
0.86
0.84
1.00
1.04
1.03
1.04
1.06
0.51
0.50
3.22
1.91
1.23
10.60
3.18
f1 (ppm)

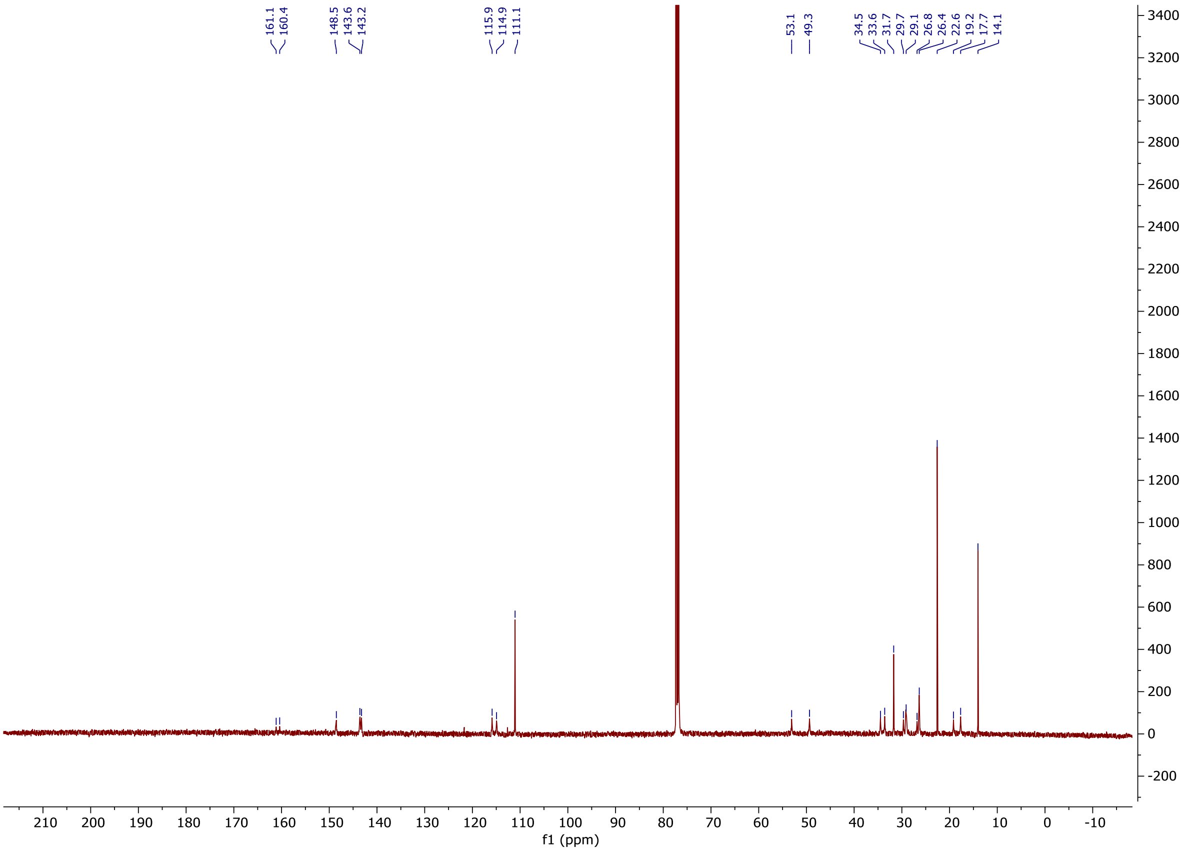
161.1
160.4
148.5
143.6
143.2
115.9
114.9
111.1
53.1
49.3
34.5
33.6
31.7
29.7
29.1
26.8
26.4
22.6
19.2
17.7
14.1
f1 (ppm)

1s

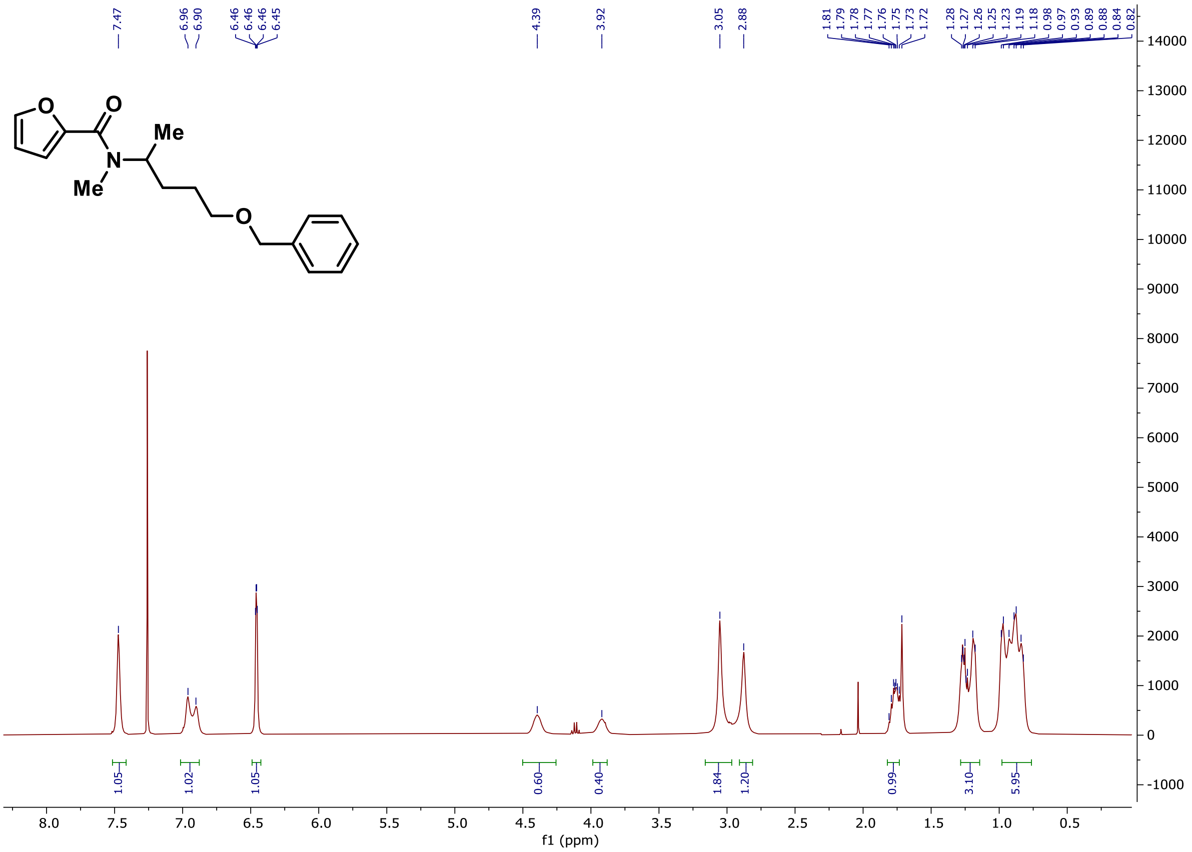
O
O
N
Me
Me
O
7.47
6.96
6.90
6.46
6.46
6.46
6.45
4.39
3.92
3.05
2.88
1.81
1.79
1.78
1.77
1.76
1.75
1.73
1.72
1.28
1.27
1.26
1.25
1.23
1.19
1.18
0.98
0.97
0.93
0.89
0.88
0.84
0.82
1.05
1.02
1.05
0.60
0.40
1.84
1.20
0.99
3.10
5.95
f1 (ppm)

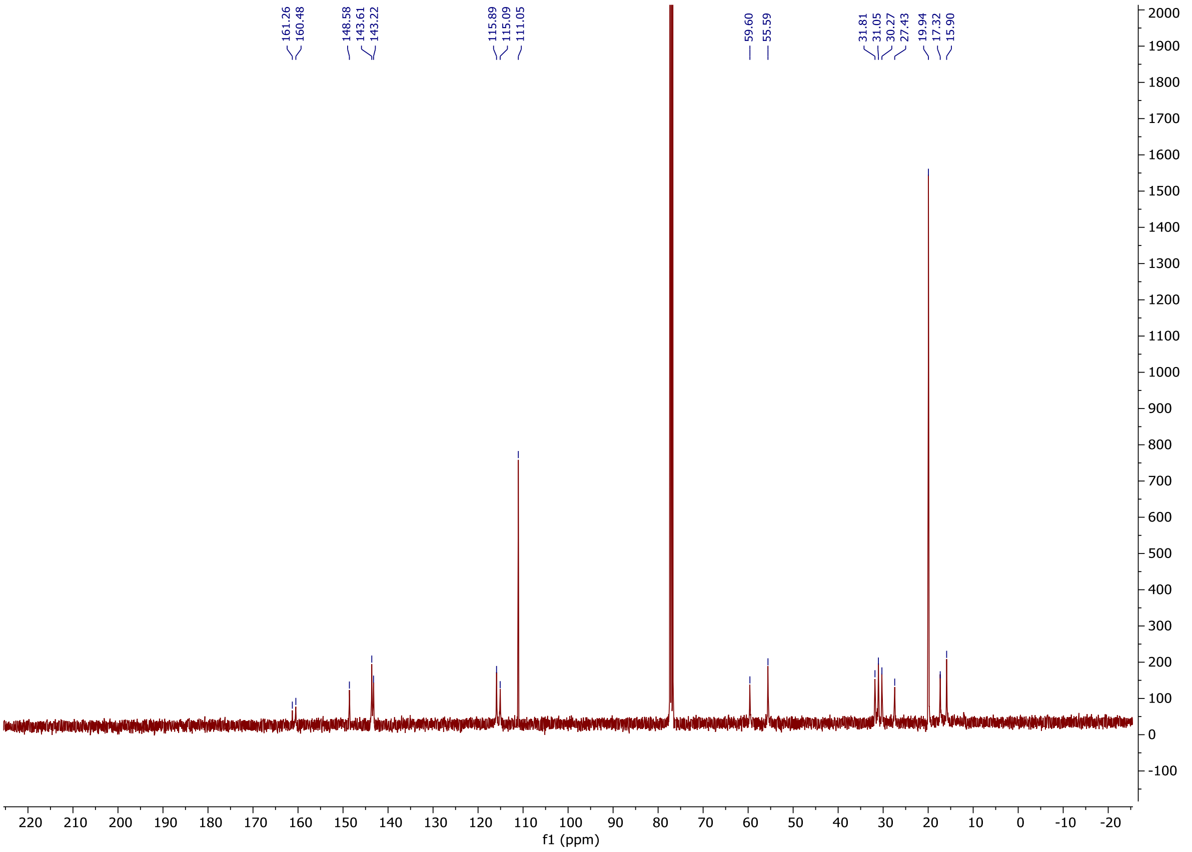
161.26
160.48
148.58
143.61
143.22
115.89
115.09
111.05
59.60
55.59
31.81
31.05
30.27
27.43
19.94
17.32
15.90
f1 (ppm)

1t

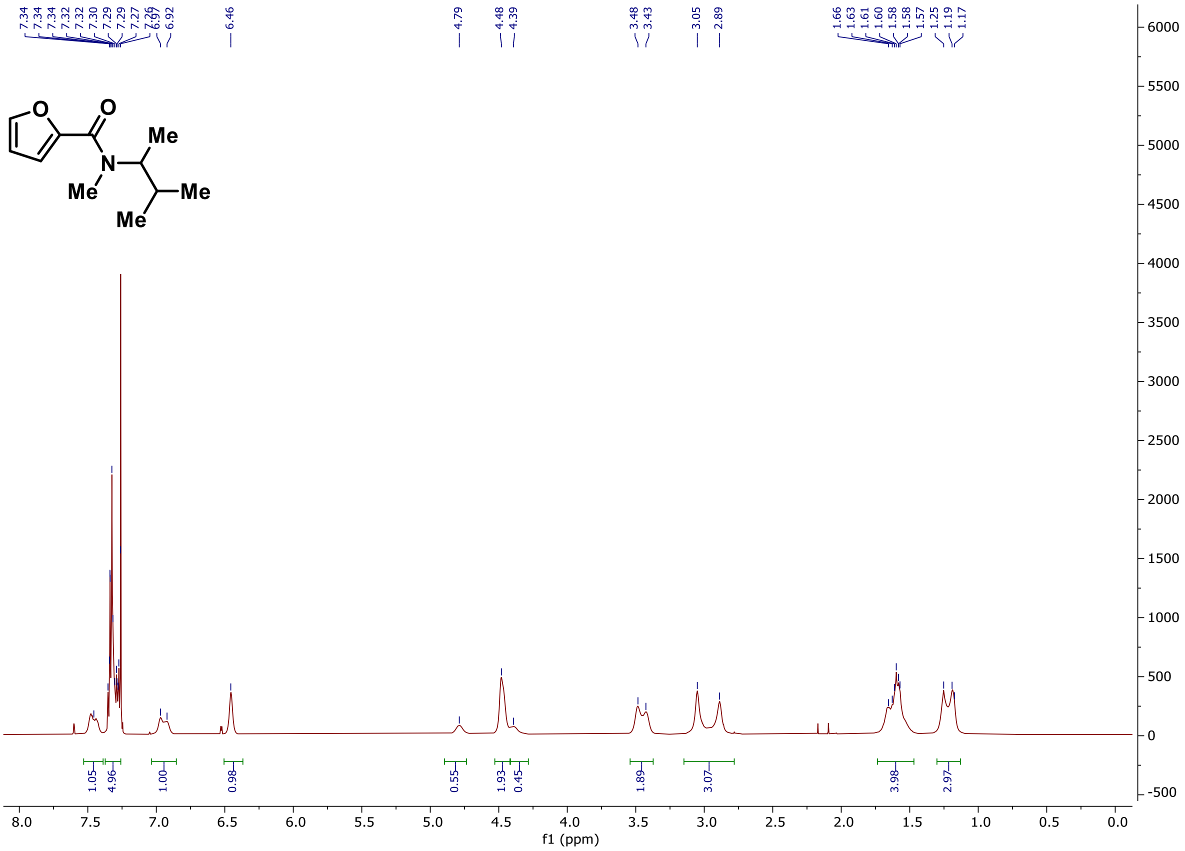

7.34
7.34
7.34
7.32
7.32
7.30
7.29
7.29
7.27
7.26
6.97
6.92
6.46
4.79
4.48
4.39
3.48
3.43
3.05
2.89
1.66
1.63
1.61
1.60
1.58
1.58
1.57
1.25
1.19
1.17
1.05
4.96
1.00
0.98
0.55
1.93
0.45
1.89
3.07
3.98
2.97
f1 (ppm)

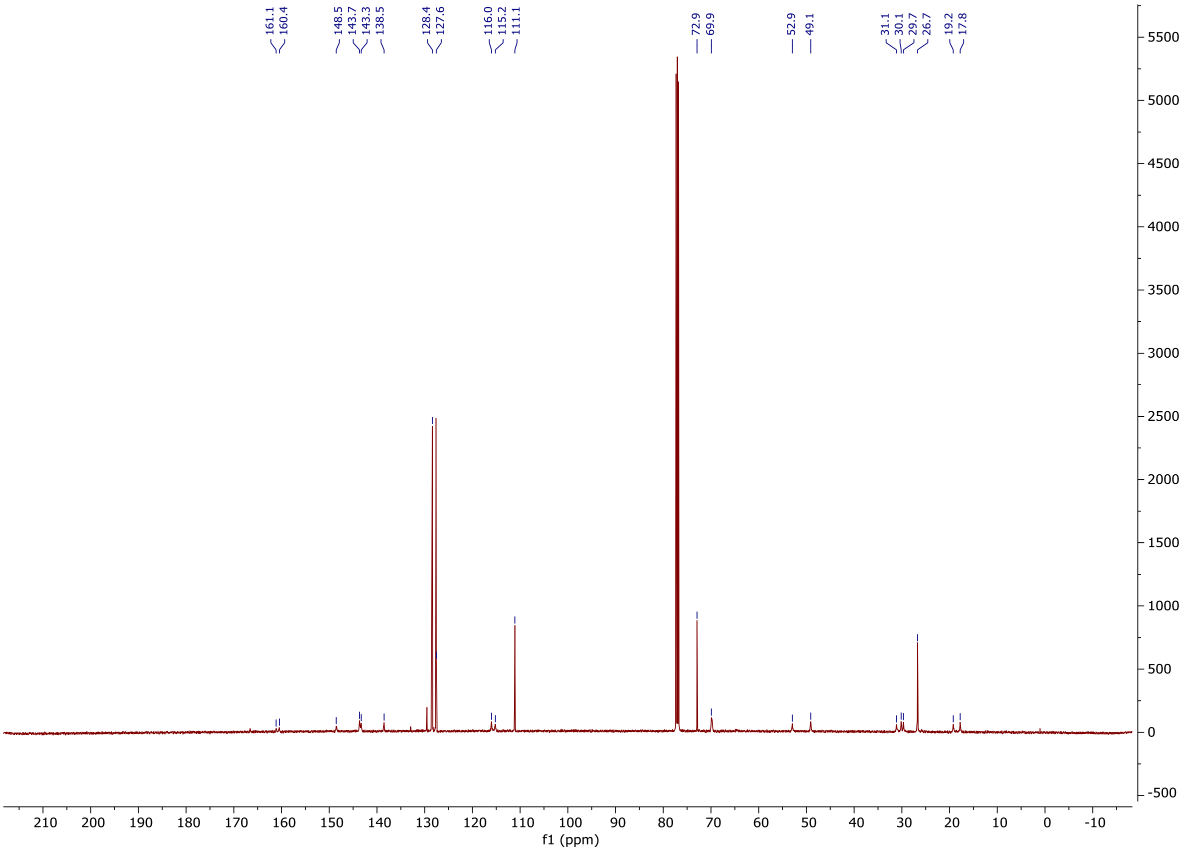

161.1
160.4
148.5
143.7
143.3
138.5
128.4
127.6
116.0
115.2
111.1
72.9
69.9
52.9
49.1
31.1
30.1
29.7
26.7
19.2
17.8
f1 (ppm)

1u

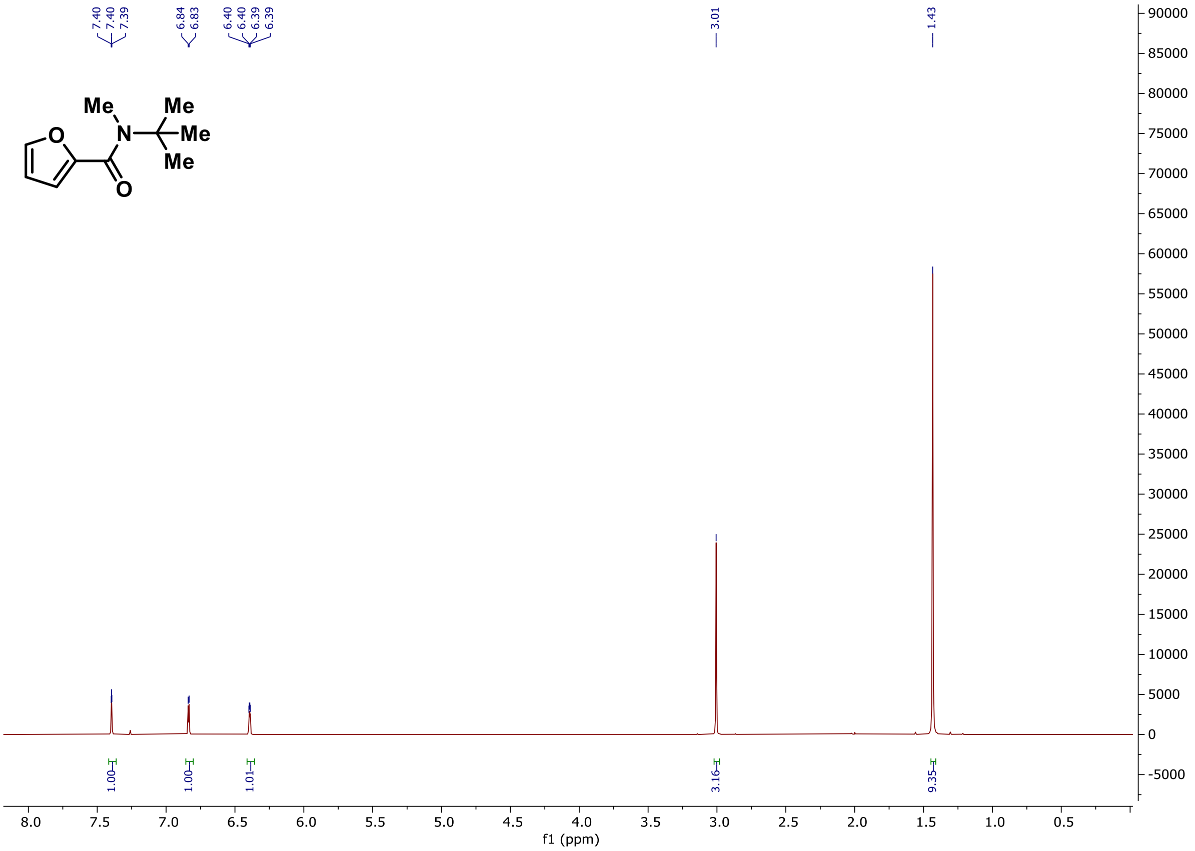
7.40
7.40
7.39
6.84
6.83
6.40
6.40
6.39
6.39
3.01
1.43
Me
N
Me
Me
Me
O
O
1.00
1.00
1.01
3.16
9.35
f1 (ppm)

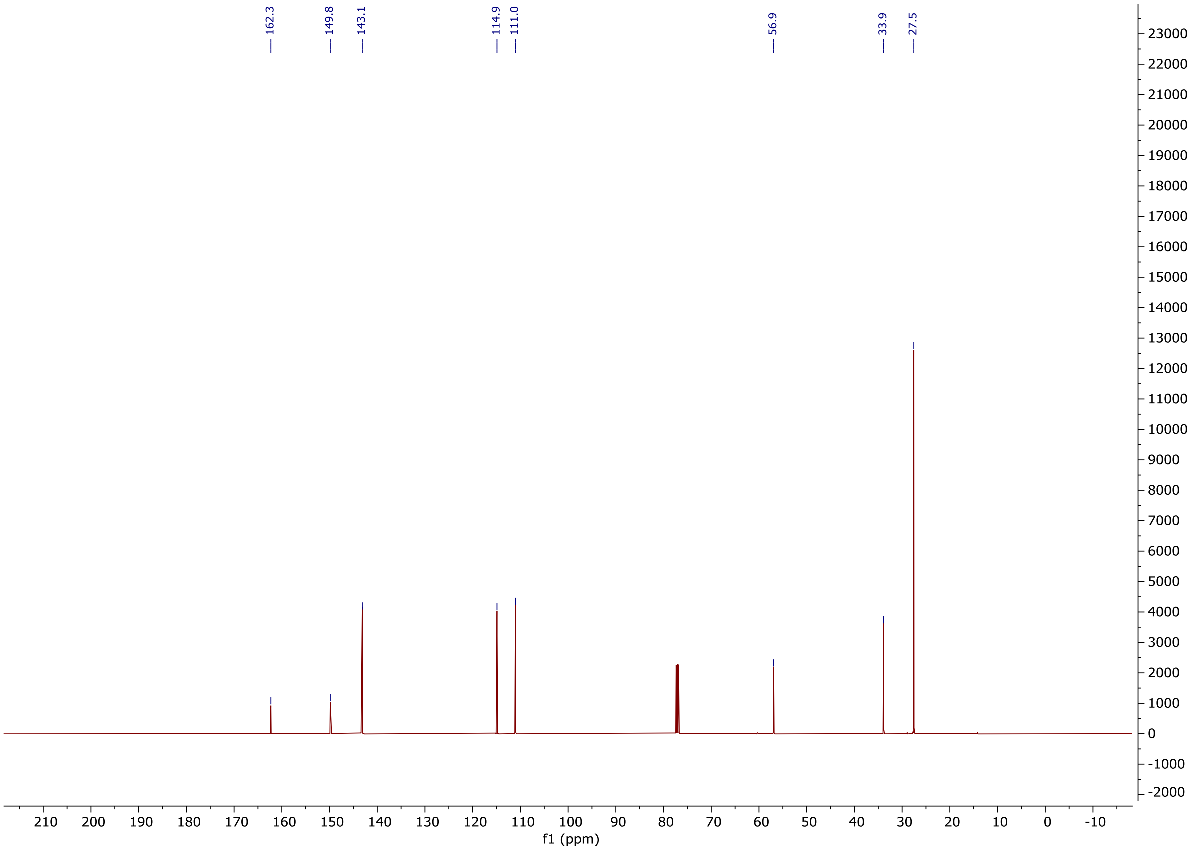
162.3
149.8
143.1
114.9
111.0
56.9
33.9
27.5
f1 (ppm)

Intramolecular N→C Alkyl Transfer Involving Other Alkyl Units: Products (Table 1B)

4p

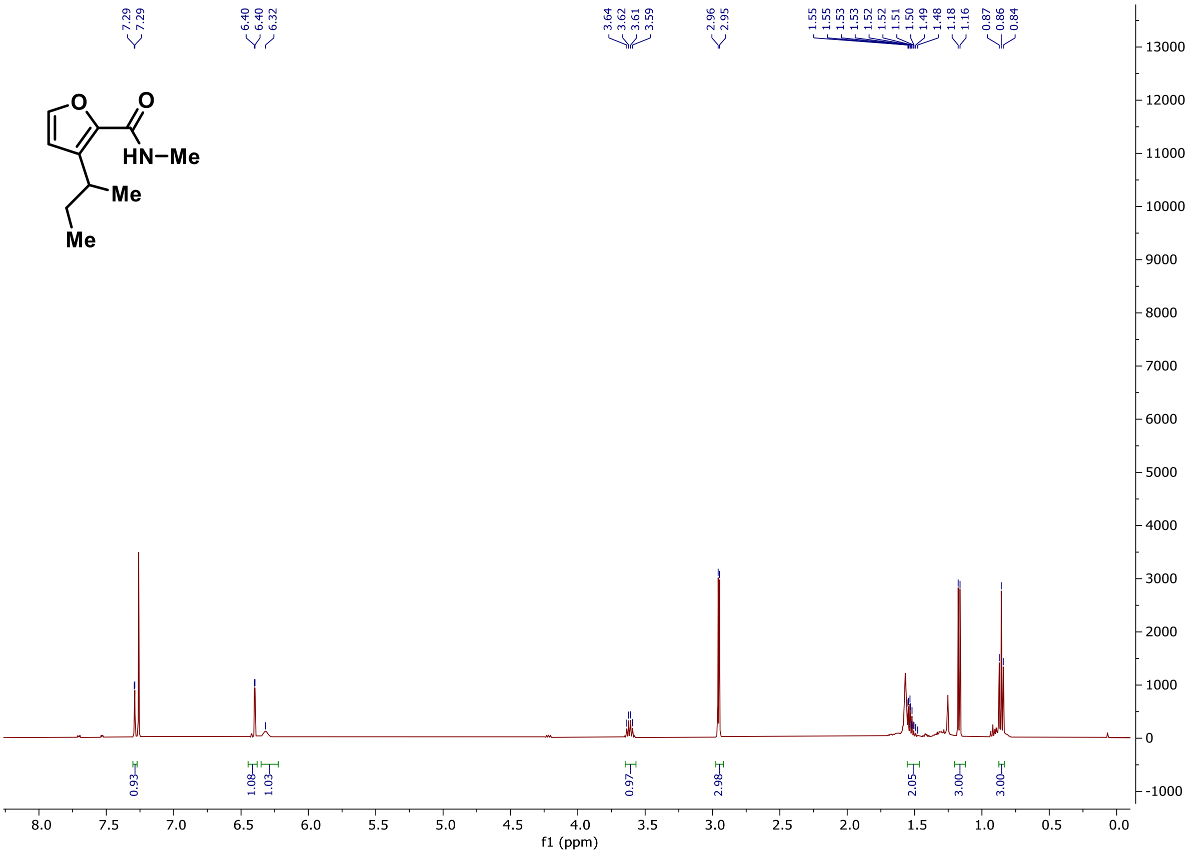

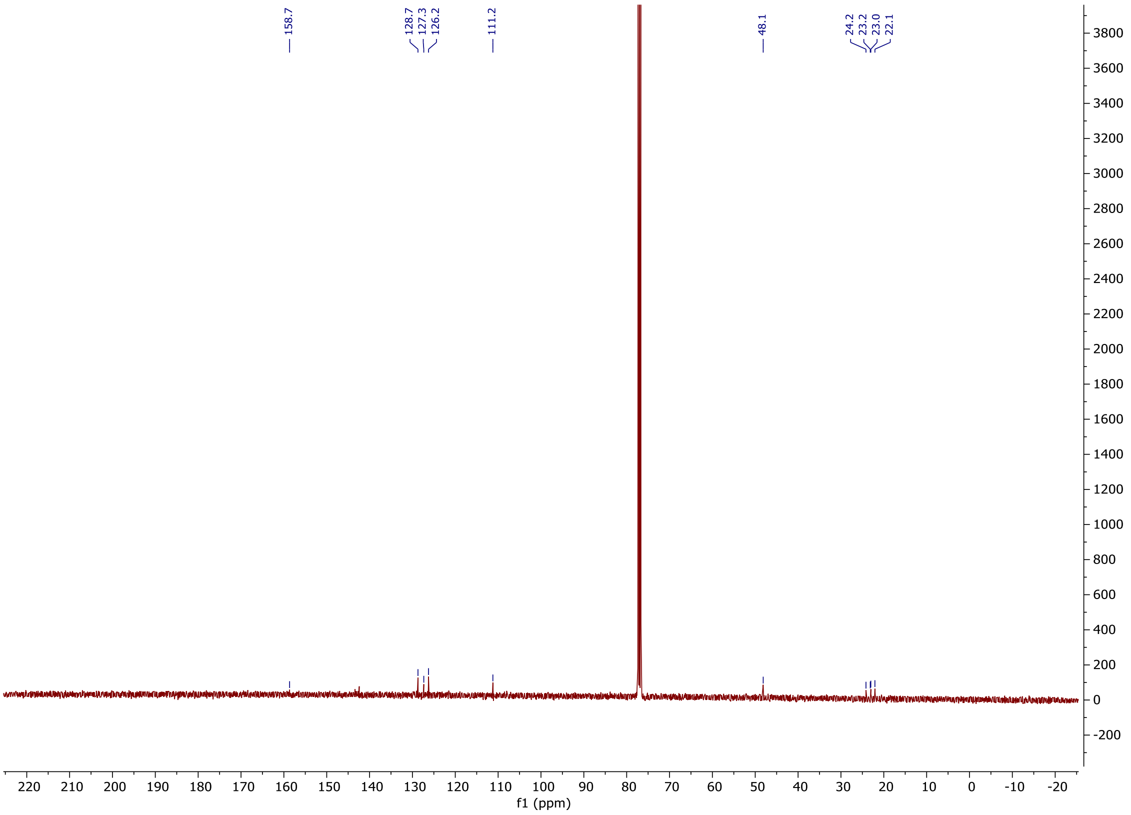

4q

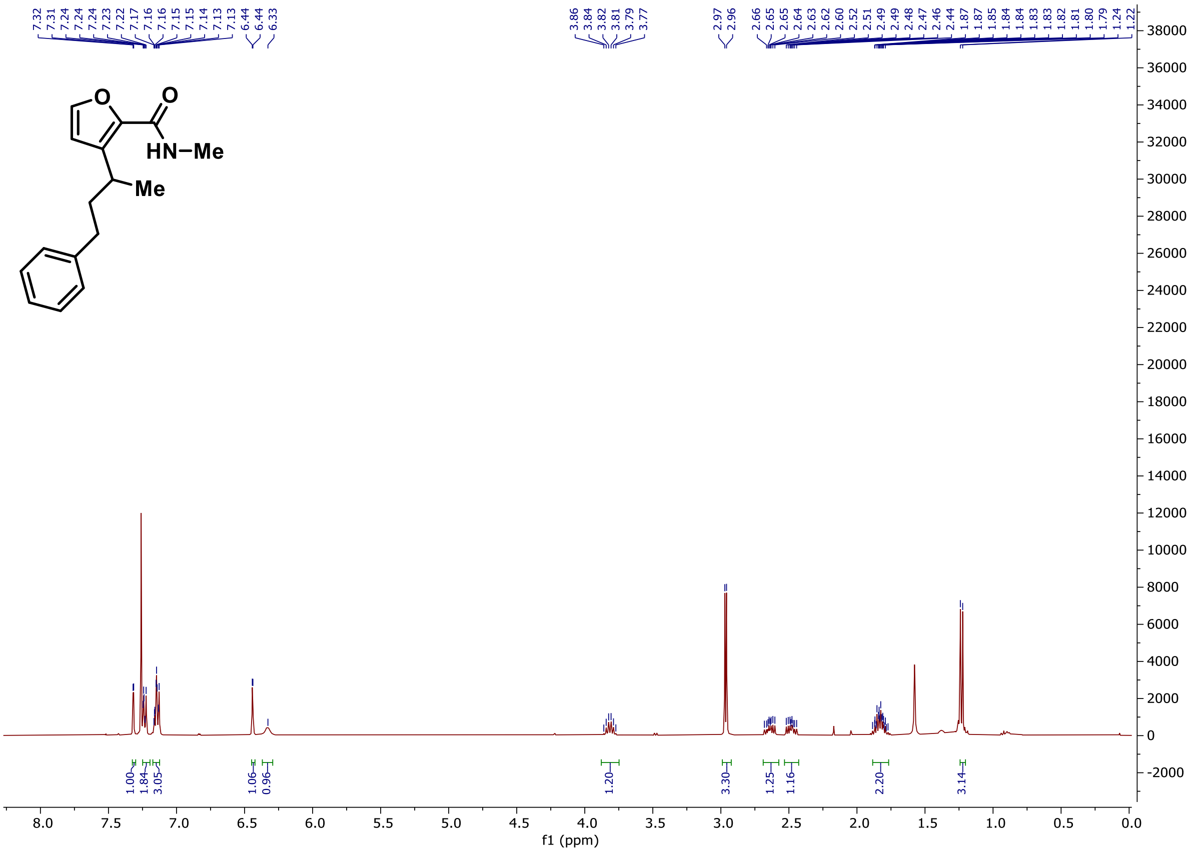
7.32 7.31 7.24 7.24 7.24 7.23 7.22 7.17 7.16 7.16 7.15 7.15 7.14 7.13 7.13 6.44 6.44 6.33
3.86 3.84 3.82 3.81 3.79 3.77
2.97 2.96
2.66 2.65 2.65 2.64 2.63 2.62 2.60 2.52 2.51 2.49 2.49 2.48 2.47 2.46 2.44 1.87 1.87 1.85 1.84 1.84 1.83 1.83 1.82 1.81 1.80 1.79 1.24 1.22
O
HN−Me
Me
1.00 1.84 3.05 1.06 0.96 1.20 3.30 1.25 1.16 2.20 3.14
f1 (ppm)

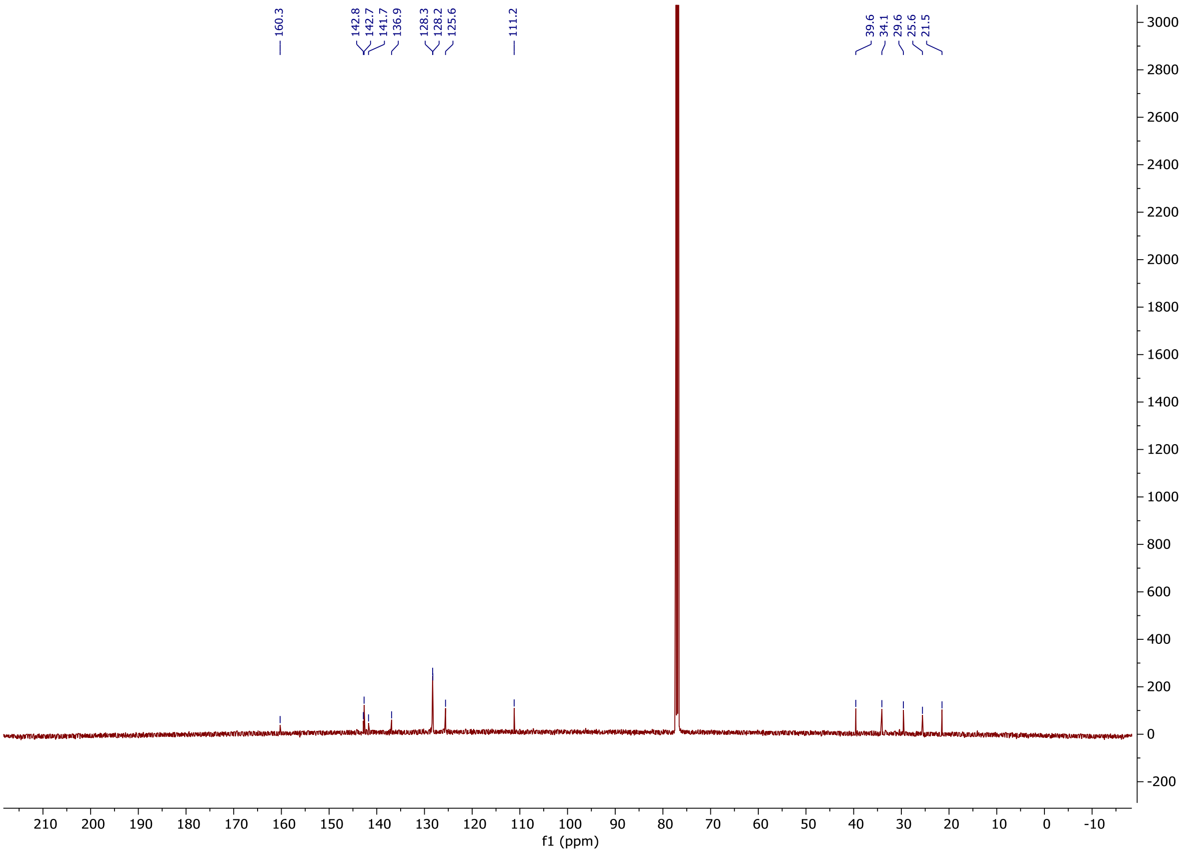
160.3
142.8 142.7 141.7 136.9
128.3 128.2 125.6
111.2
39.6 34.1 29.6 25.6 21.5
f1 (ppm)

4r

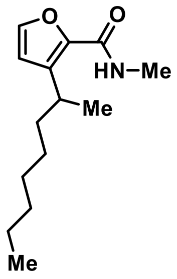

O
HN−Me
Me
Me

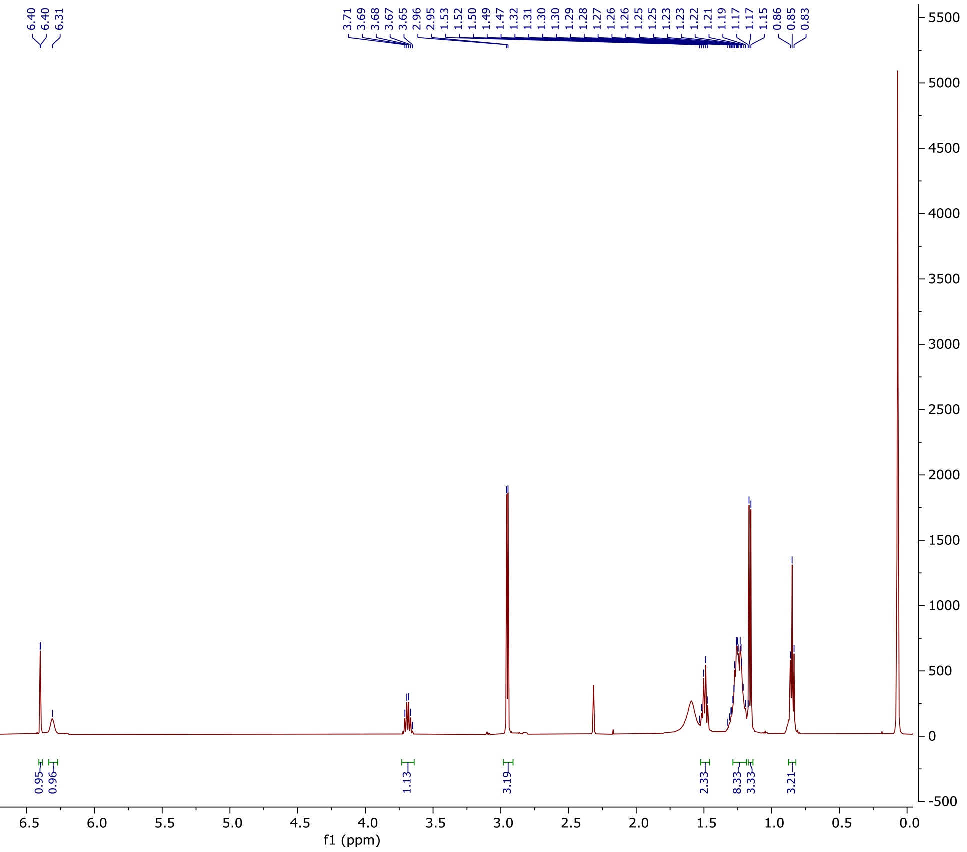

7.29
7.28
6.40
6.40
6.31
3.71
3.69
3.68
3.67
3.65
2.96
2.95
1.53
1.52
1.50
1.49
1.47
1.32
1.31
1.30
1.30
1.29
1.28
1.27
1.26
1.26
1.25
1.25
1.23
1.23
1.22
1.21
1.19
1.17
1.17
1.15
0.86
0.85
0.83
1.00
0.95
0.96
1.13
3.19
2.33
8.33
3.33
3.21
f1 (ppm)

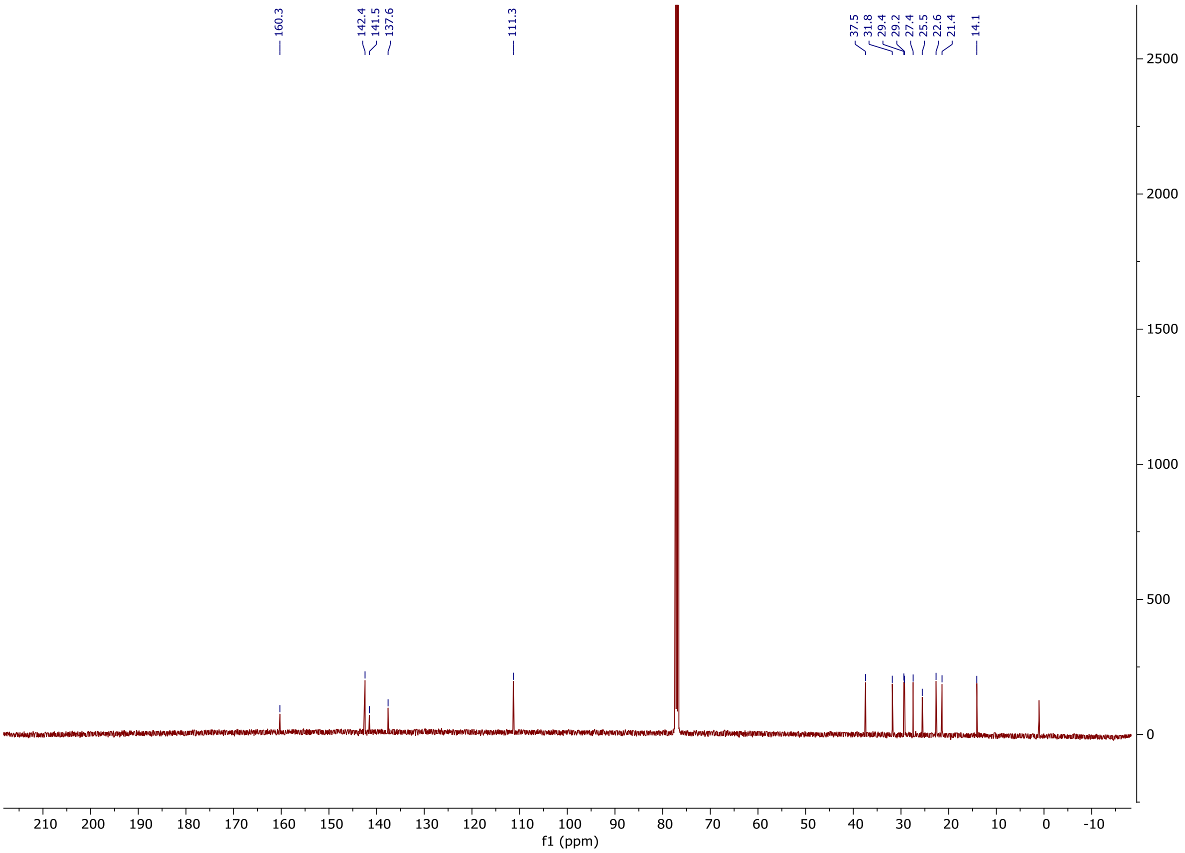

160.3
142.4
141.5
137.6
111.3
37.5
31.8
29.4
29.2
27.4
25.5
22.6
21.4
14.1
f1 (ppm)

4s

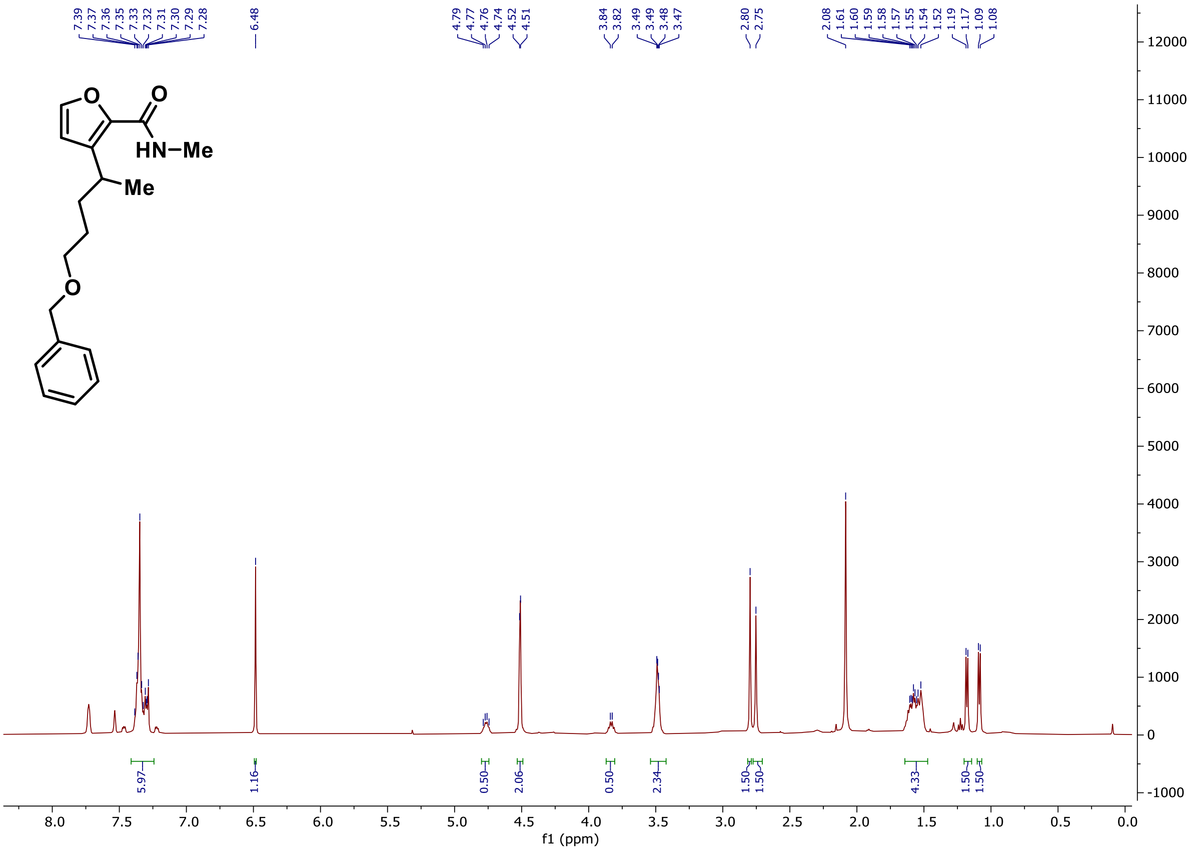
7.39 7.37 7.36 7.35 7.33 7.32 7.31 7.30 7.29 7.28
6.48
4.79 4.77 4.76 4.74 4.52 4.51
3.84 3.82 3.49 3.49 3.48 3.47
2.80 2.75
2.08 1.61 1.60 1.59 1.58 1.57 1.55 1.54 1.52 1.19 1.17 1.09 1.08
O
O
HN−Me
Me
O
5.97 1.16 0.50 2.06 0.50 2.34 1.50 1.50 4.33 1.50 1.50
f1 (ppm)

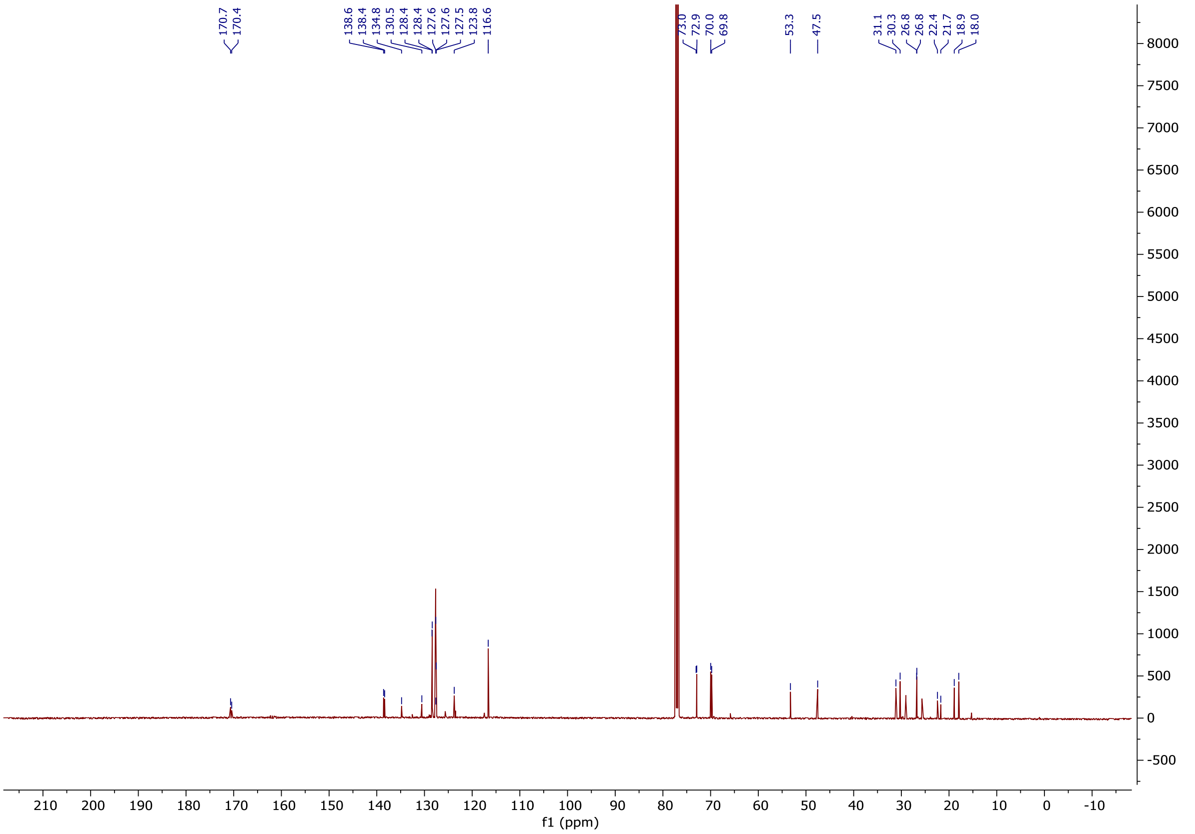
170.7 170.4
138.6 138.4 134.8 130.5 128.4 128.4 127.6 127.6 127.5 123.8 116.6
73.0 72.9 70.0 69.8
53.3
47.5
31.1 30.3 26.8 26.8 22.4 21.7 18.9 18.0
f1 (ppm)

4t

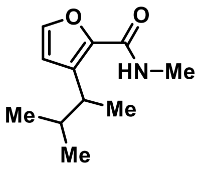
O
O
HN–Me
Me
Me
Me

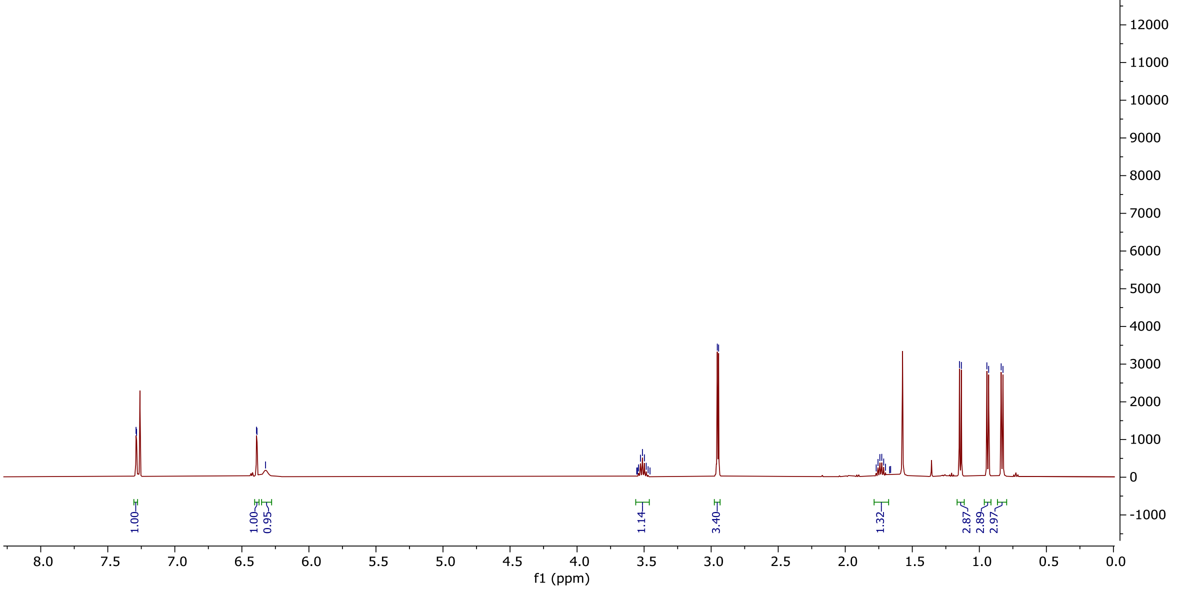
7.29
7.29
6.39
6.39
6.32
3.55
3.55
3.54
3.53
3.51
3.50
3.48
3.47
3.46
2.95
2.94
1.77
1.76
1.74
1.73
1.71
1.70
1.67
1.66
1.15
1.13
0.94
0.93
0.84
0.82
1.00
1.00
0.95
1.14
3.40
1.32
2.87
2.89
2.97
f1 (ppm)

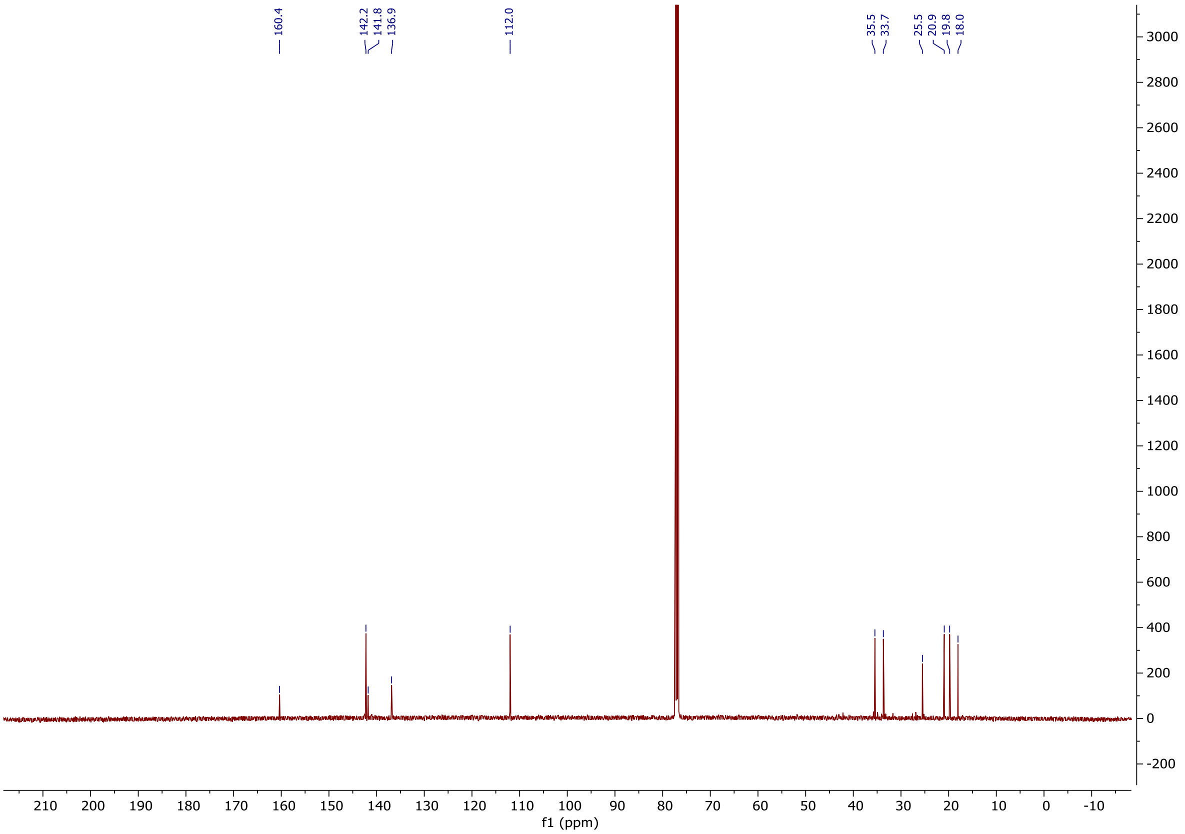
160.4
142.2
141.8
136.9
112.0
35.5
33.7
25.5
20.9
19.8
18.0
f1 (ppm)

4u

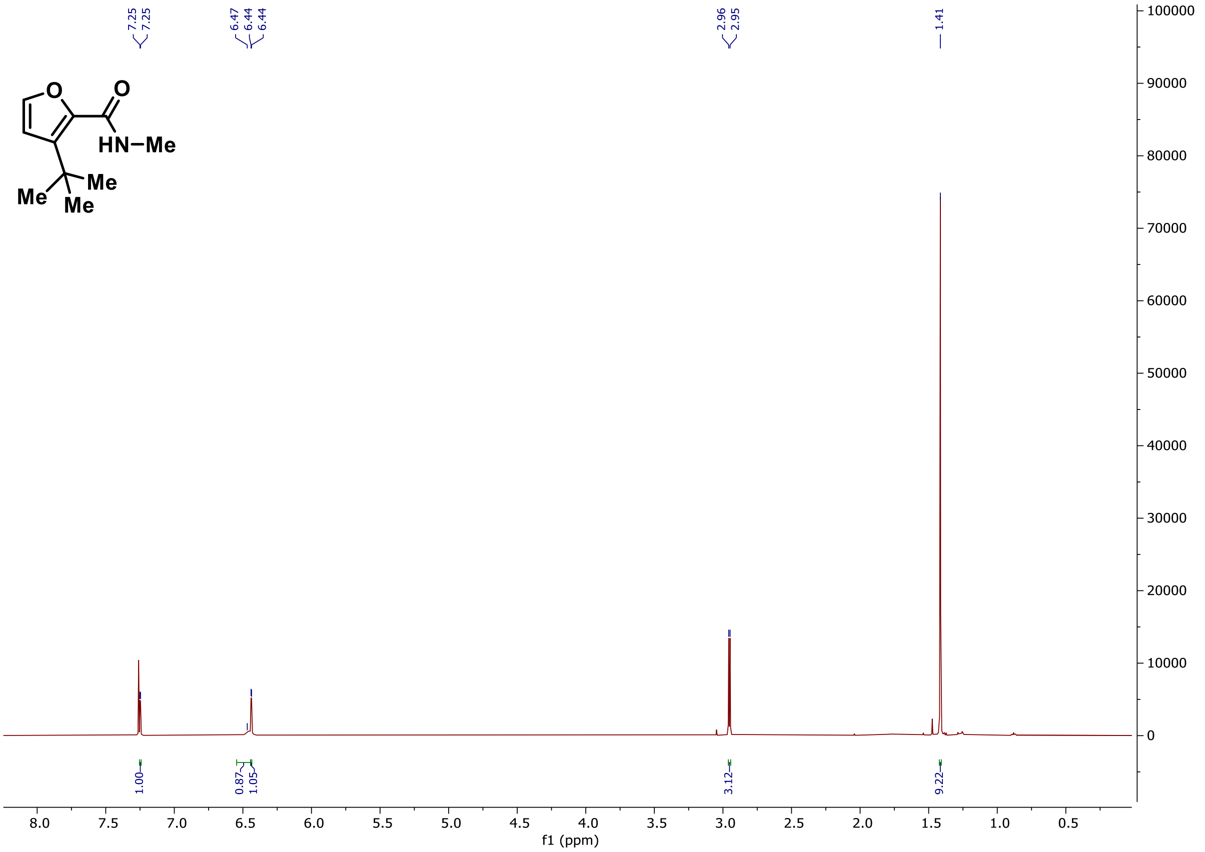

O
HN–Me
Me
Me
Me
7.25
7.25
6.47
6.44
6.44
2.96
2.95
1.41
1.00
0.87
1.05
3.12
9.22
f1 (ppm)

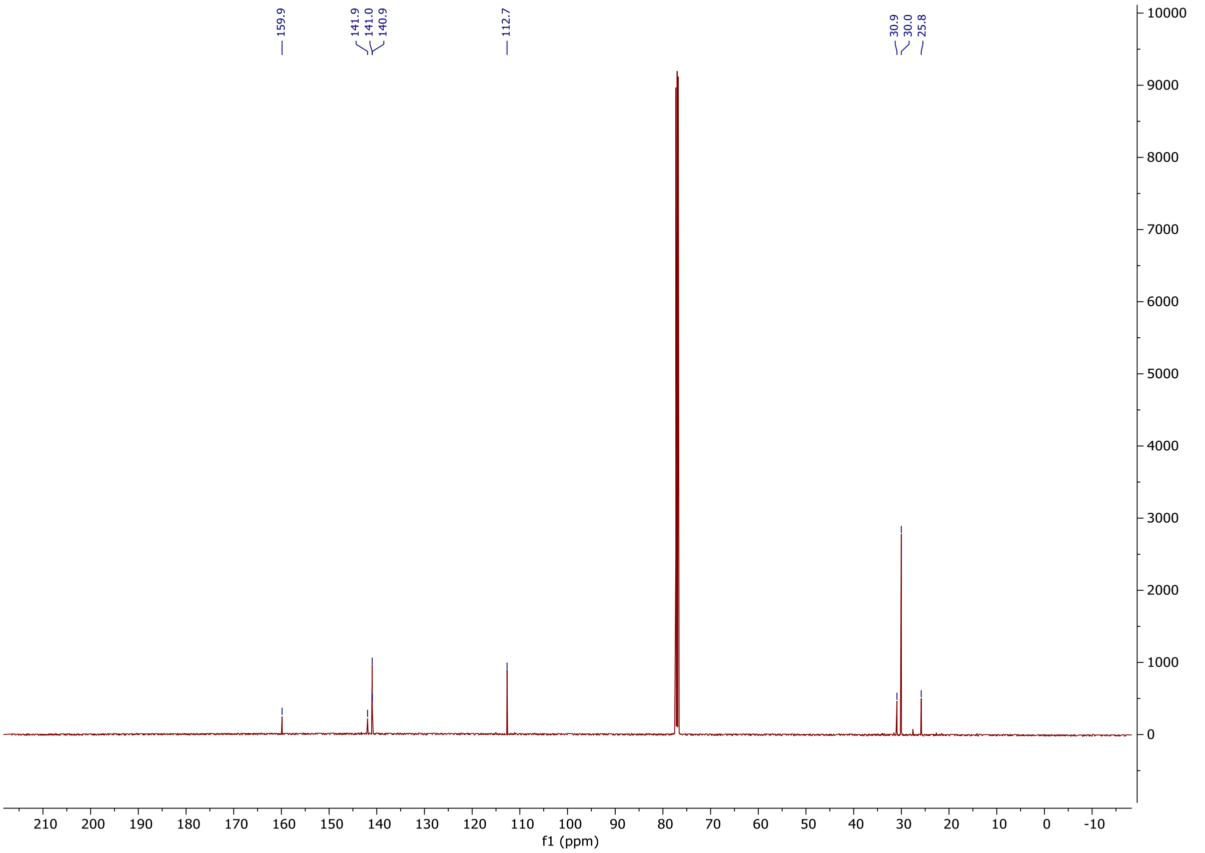

159.9
141.9
141.0
140.9
112.7
30.9
30.0
25.8
f1 (ppm)

1x

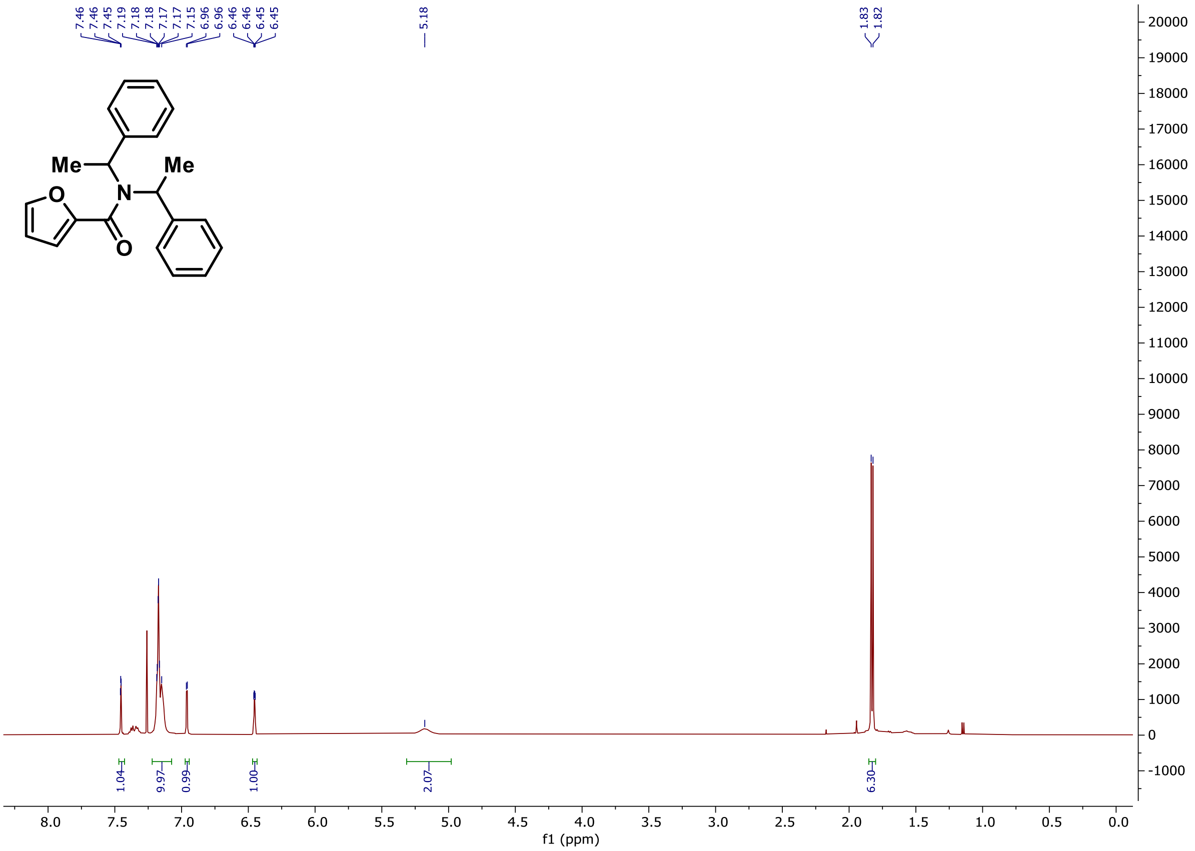
Me
Me
O
N
O
7.46
7.46
7.45
7.19
7.18
7.18
7.17
7.17
7.15
6.96
6.96
6.46
6.46
6.45
6.45
5.18
1.83
1.82
1.04
9.97
0.99
1.00
2.07
6.30
f1 (ppm)

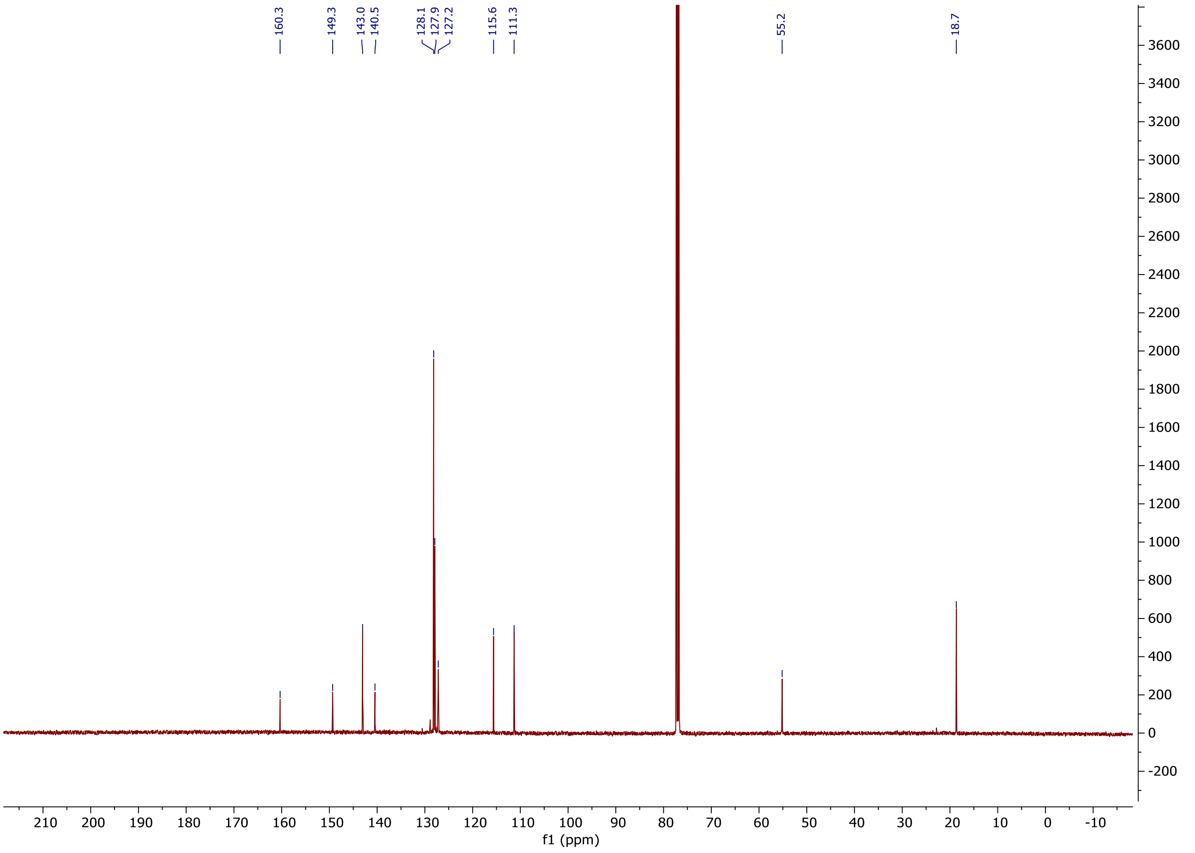
160.3
149.3
143.0
140.5
128.1
127.9
127.2
115.6
111.3
55.2
18.7
f1 (ppm)

A Key Observation: Intermolecular Variant (Scheme 4A)

1y

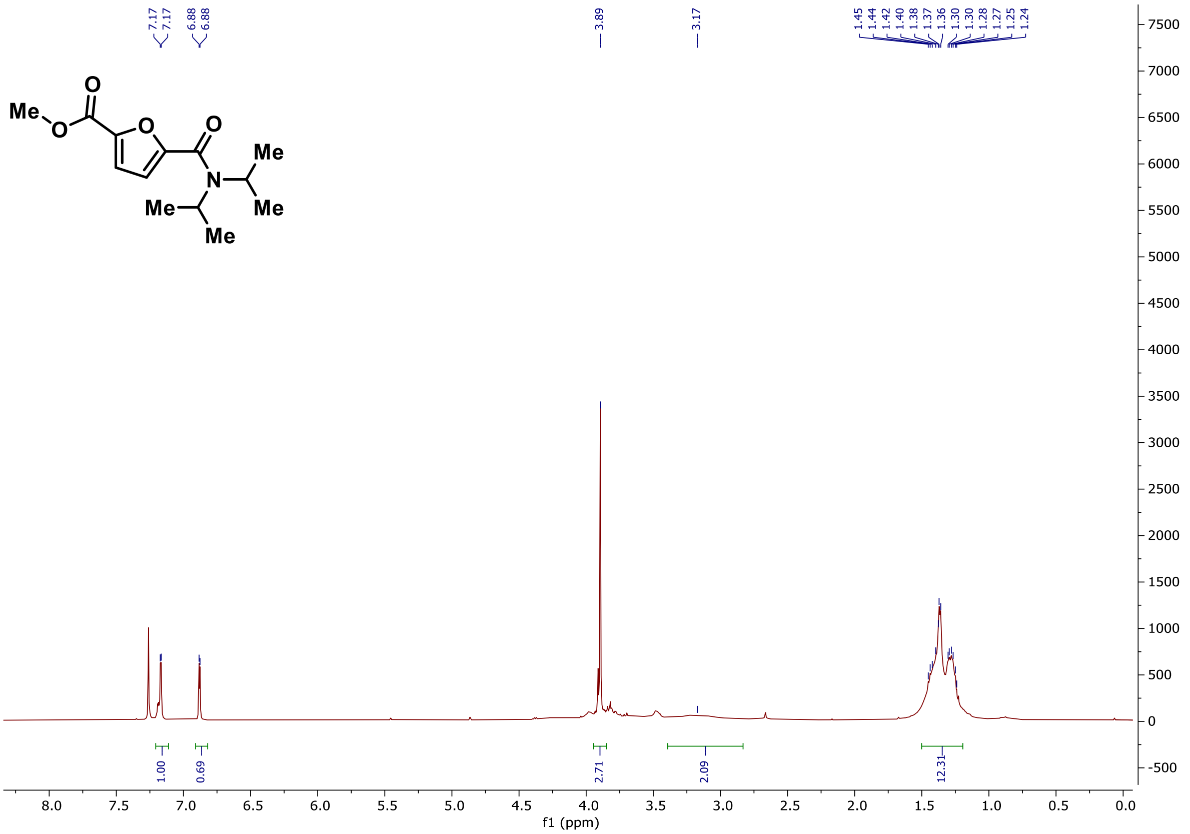

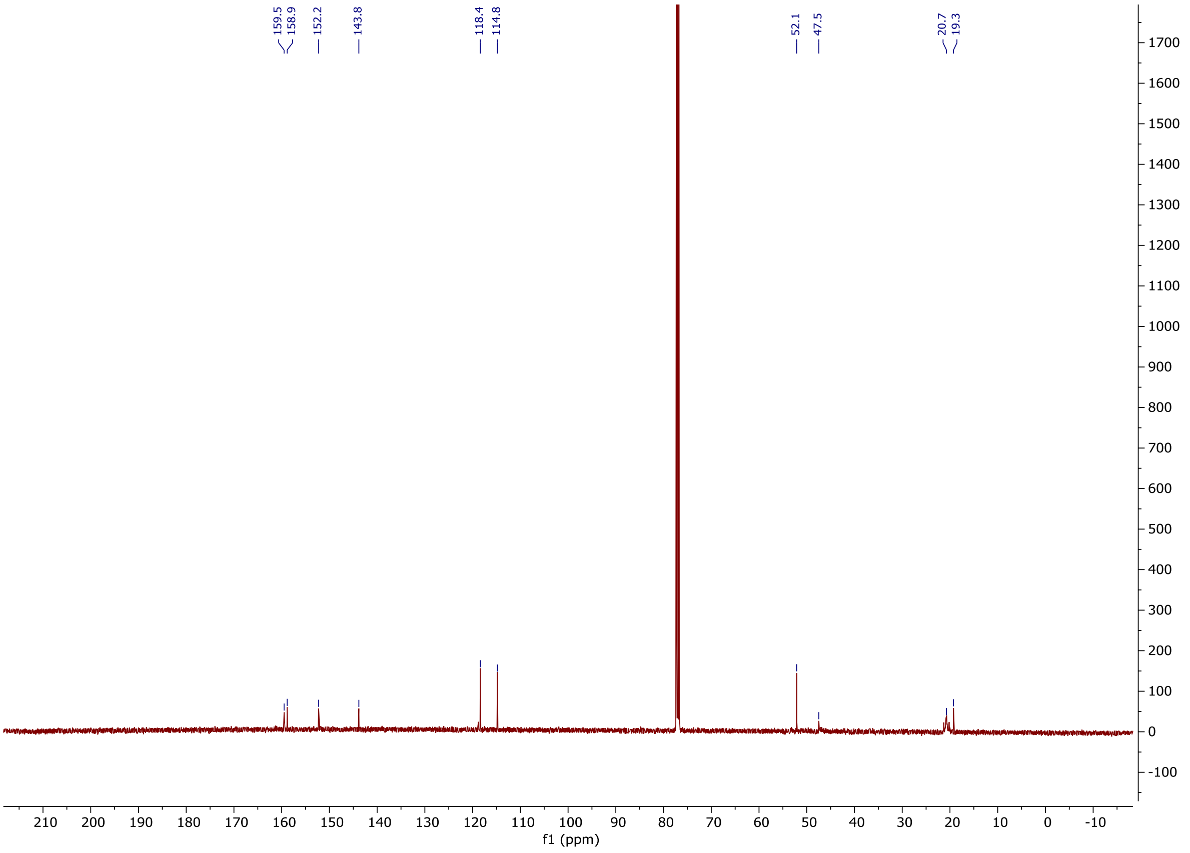

4y

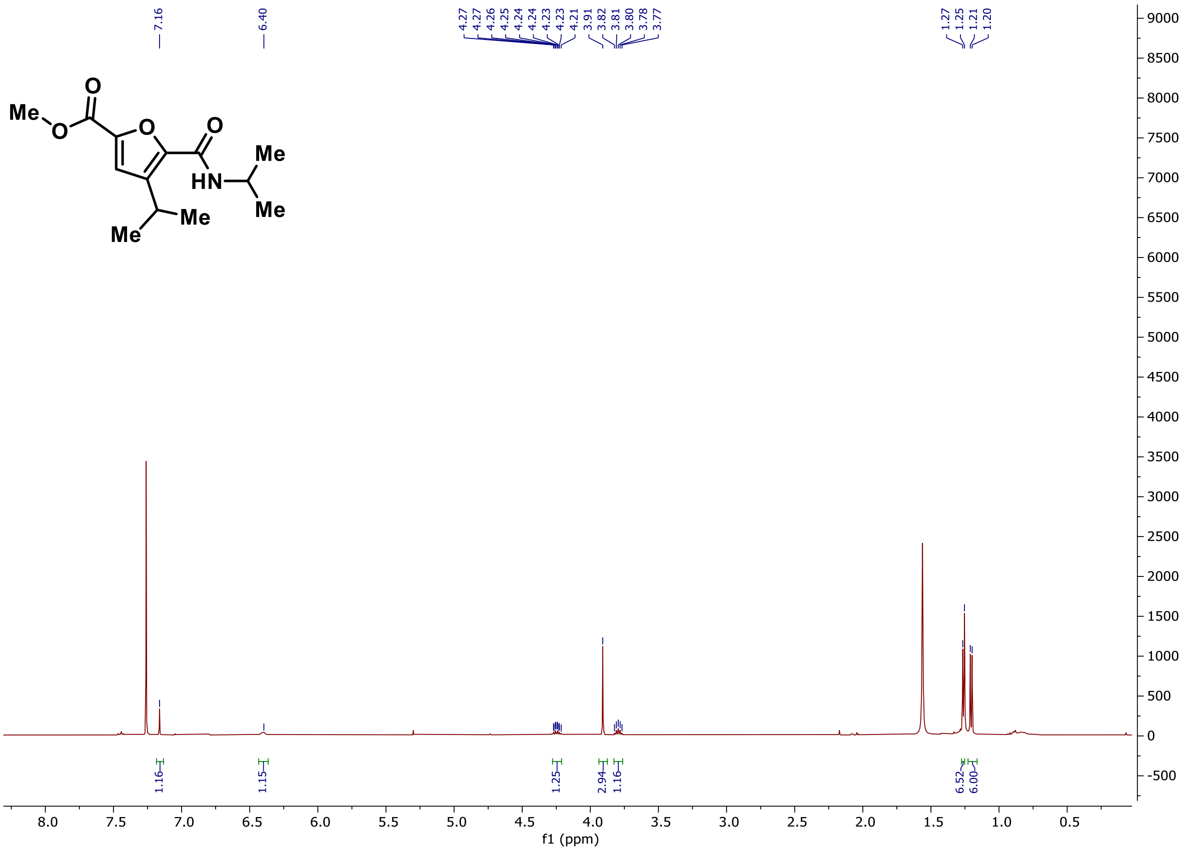
Me
O
O
O
HN
Me
Me
Me
Me
7.16
6.40
4.27
4.27
4.26
4.25
4.24
4.24
4.23
4.23
4.21
3.91
3.82
3.81
3.80
3.78
3.77
1.27
1.25
1.21
1.20
1.16
1.15
1.25
2.94
1.16
6.52
6.00
f1 (ppm)

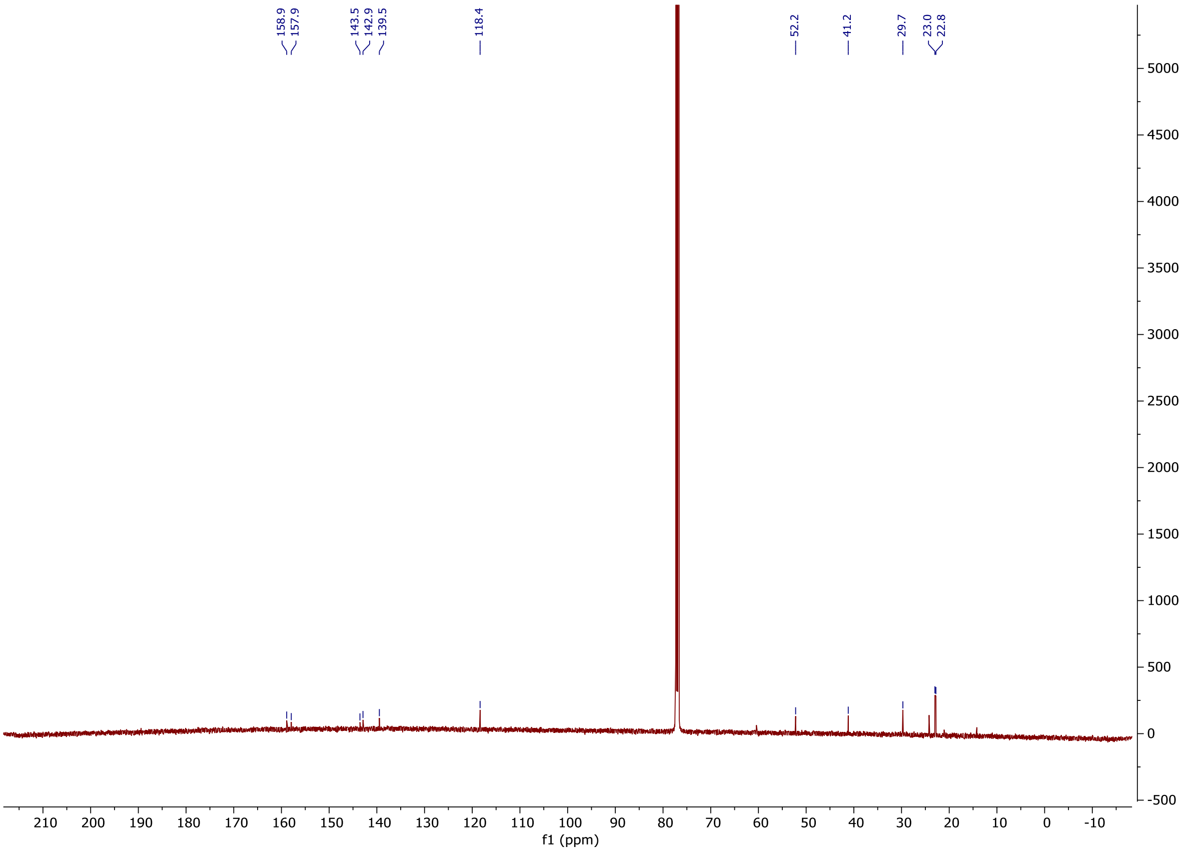
158.9
157.9
143.5
142.9
139.5
118.4
52.2
41.2
29.7
23.0
22.8
f1 (ppm)

N-*i*-Pr-4y

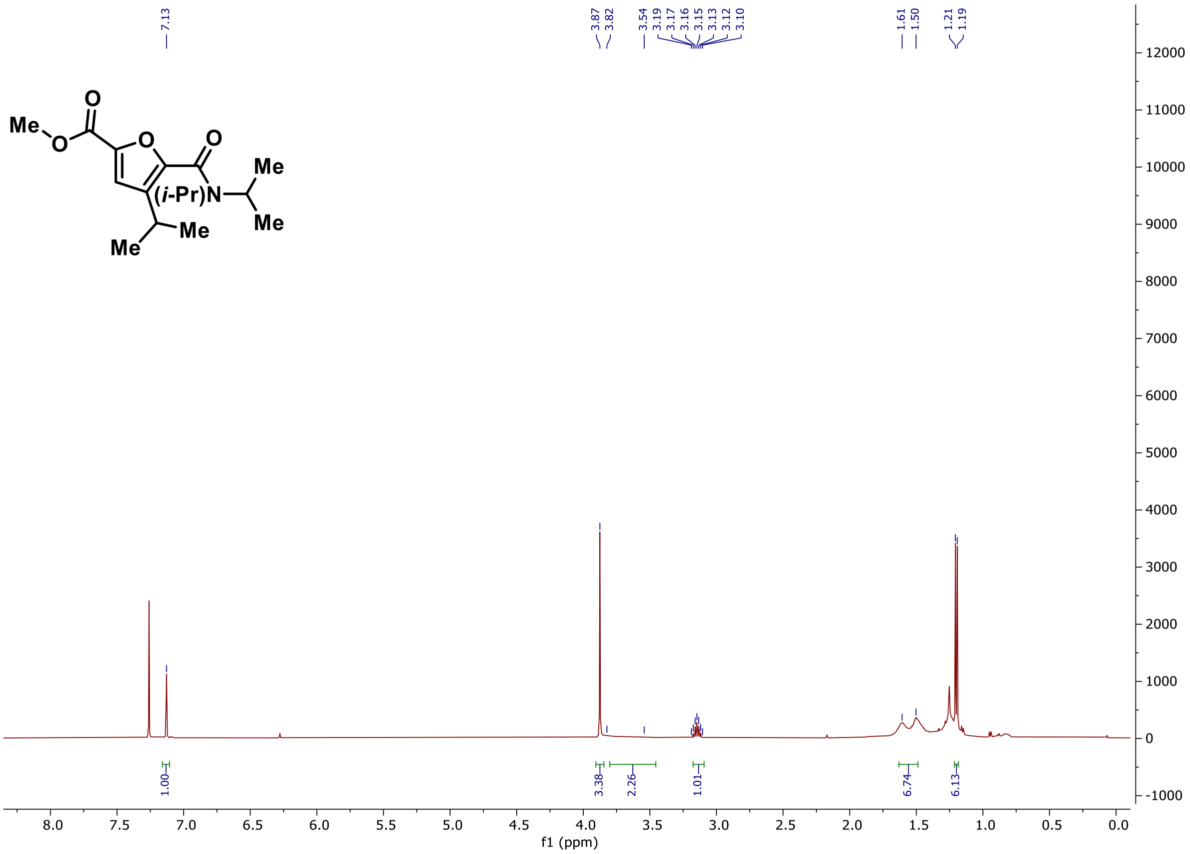

Me
O
O
O
O
(i-Pr)N
Me
Me
Me
Me
7.13
3.87
3.82
3.54
3.19
3.17
3.16
3.15
3.13
3.12
3.10
1.61
1.50
1.21
1.19
1.00
3.38
2.26
1.01
6.74
6.13
f1 (ppm)

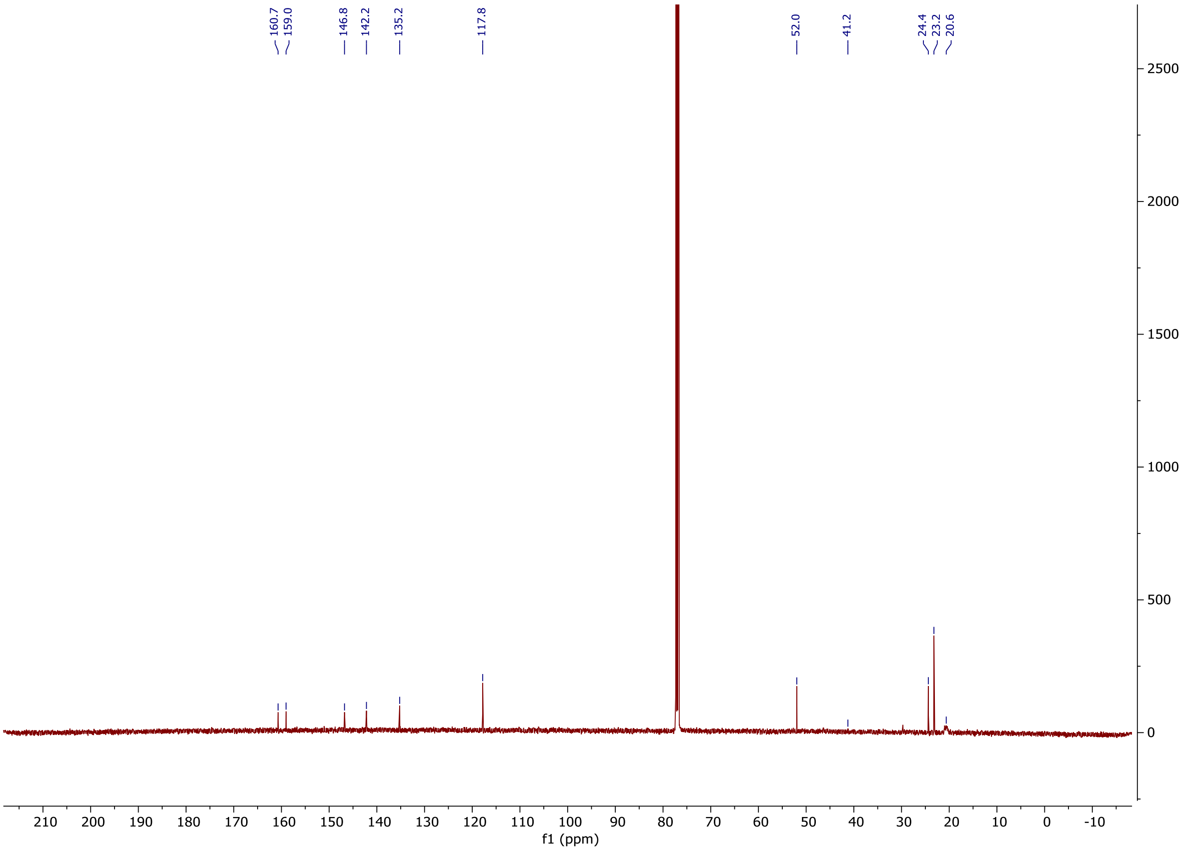

160.7
159.0
146.8
142.2
135.2
117.8
52.0
41.2
24.4
23.2
20.6
f1 (ppm)

NH-1y

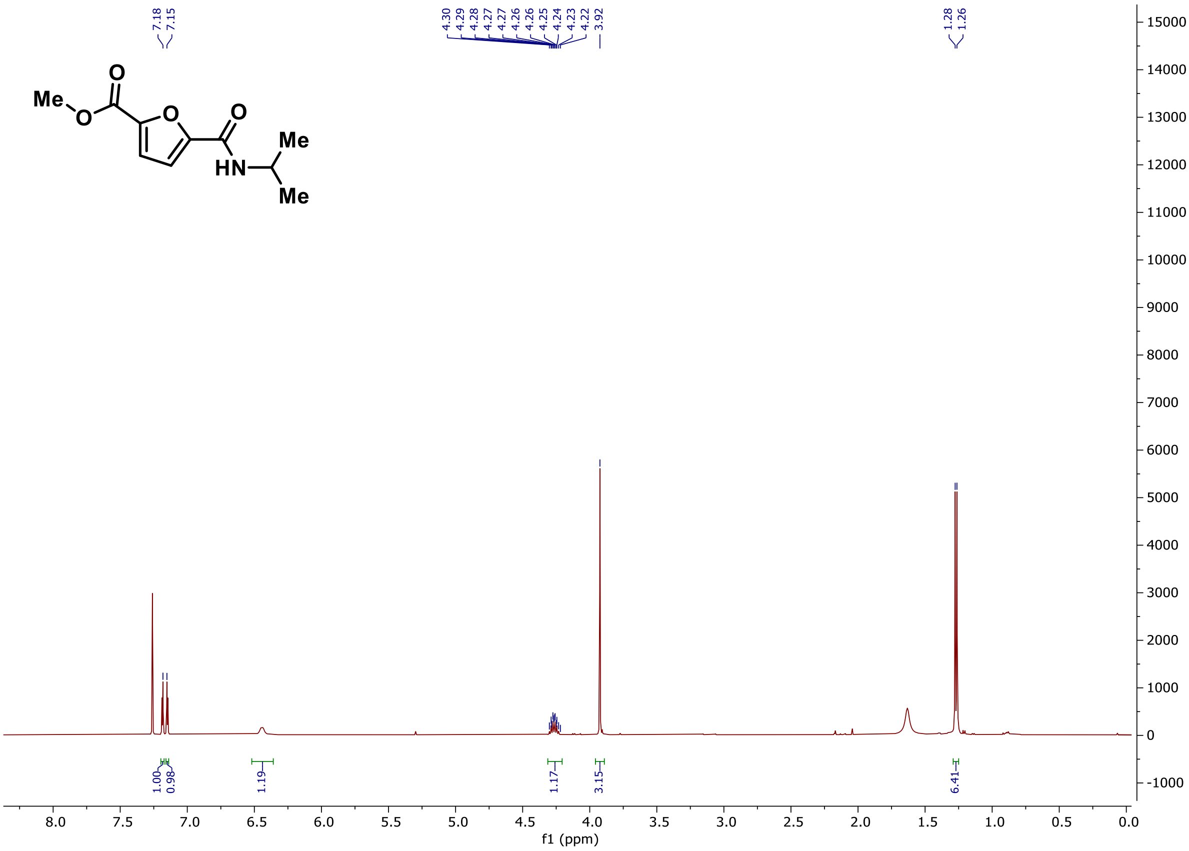
Me
O
O
O
O
HN
Me
Me
7.18
7.15
4.30
4.29
4.28
4.27
4.27
4.26
4.25
4.24
4.23
4.22
3.92
1.28
1.26
1.00
0.98
1.19
1.17
3.15
6.41
f1 (ppm)

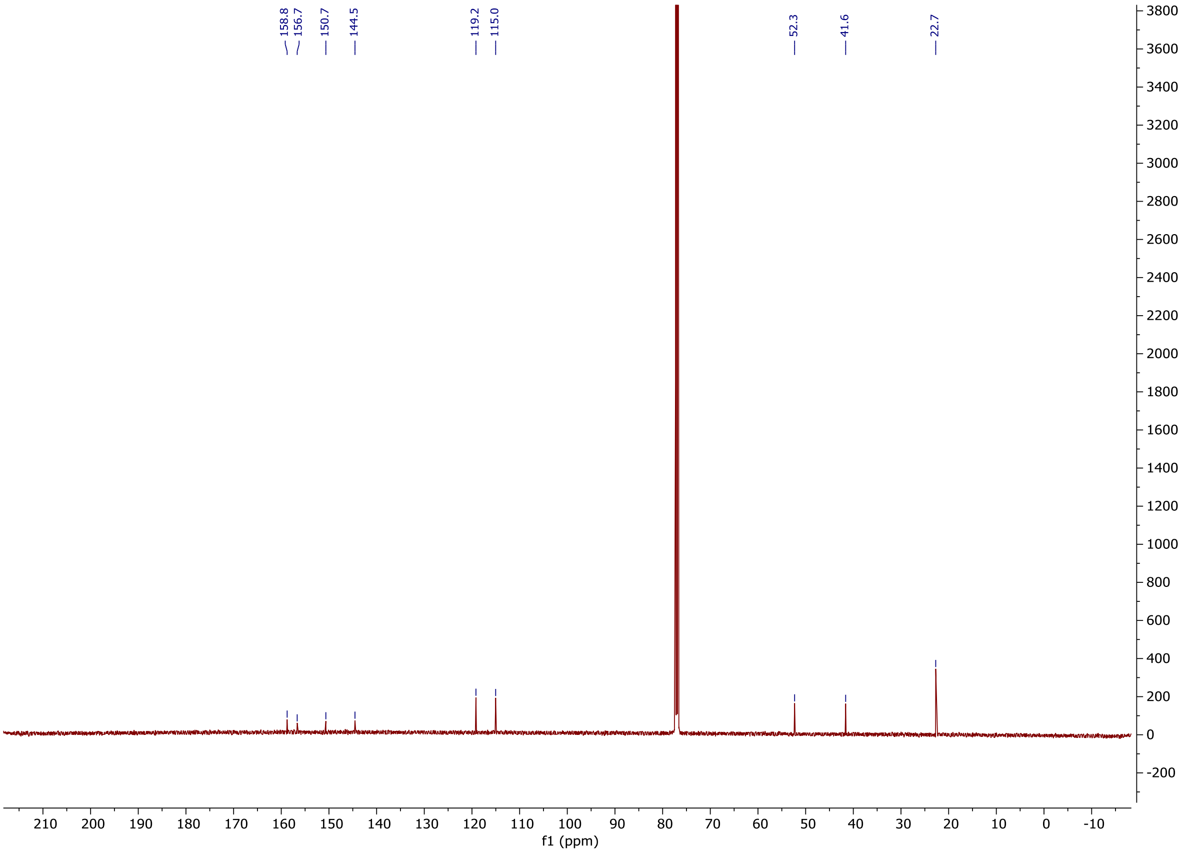
158.8
156.7
150.7
144.5
119.2
115.0
52.3
41.6
22.7
f1 (ppm)

Identification of an Effective Alkyl Transfer Reagent (Scheme 4B)

4v

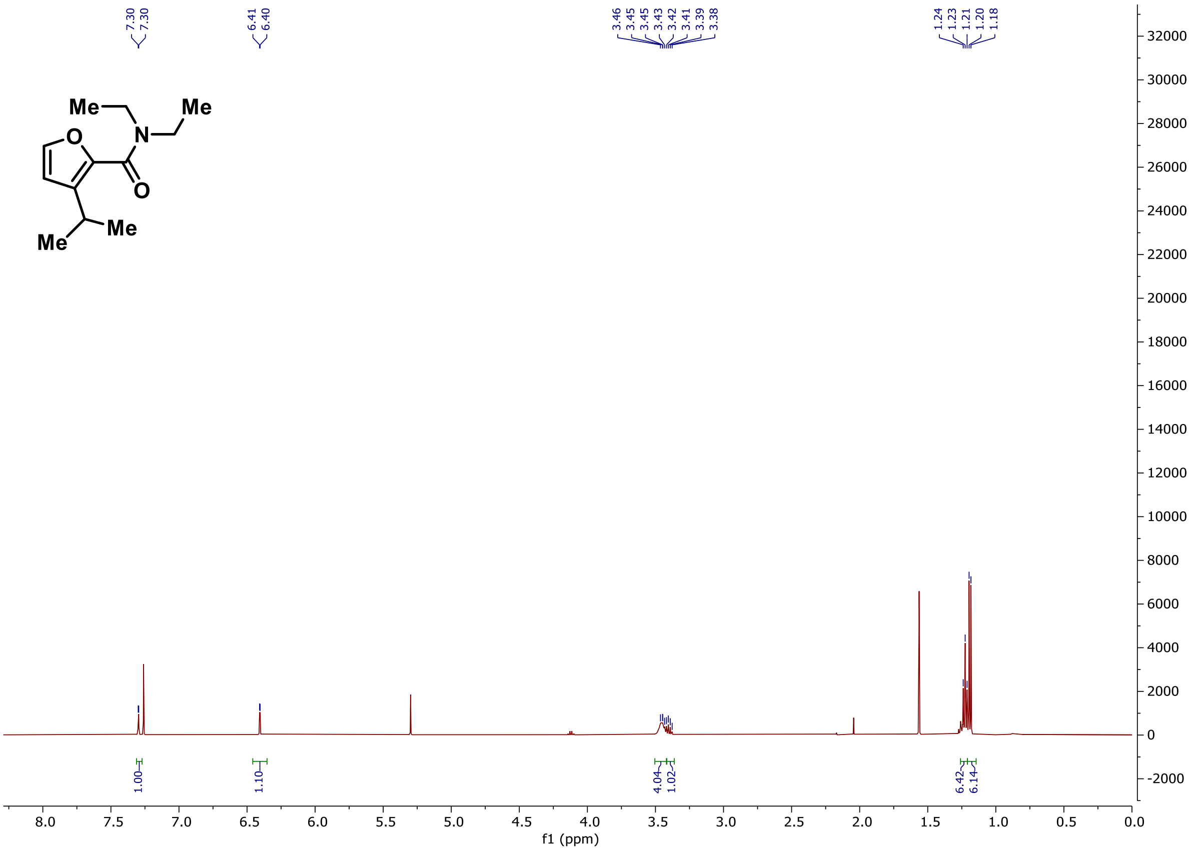

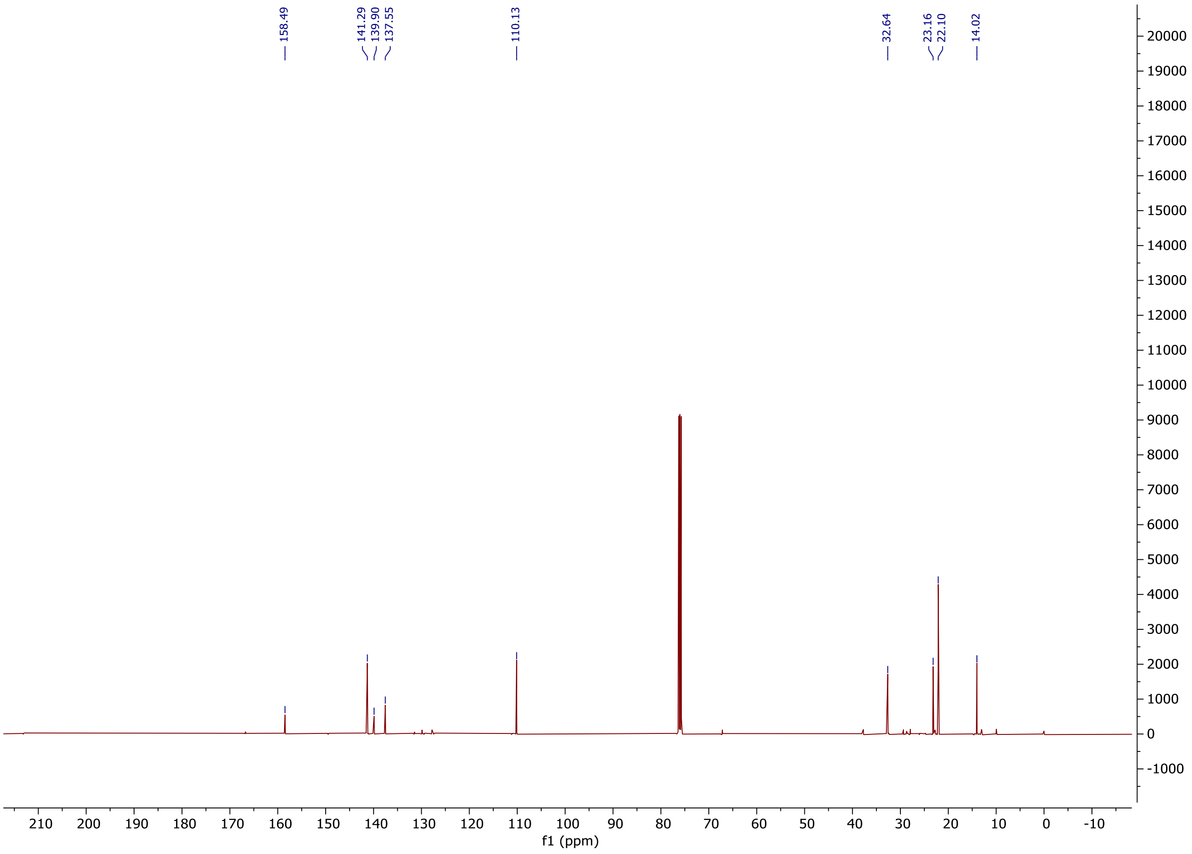

6b

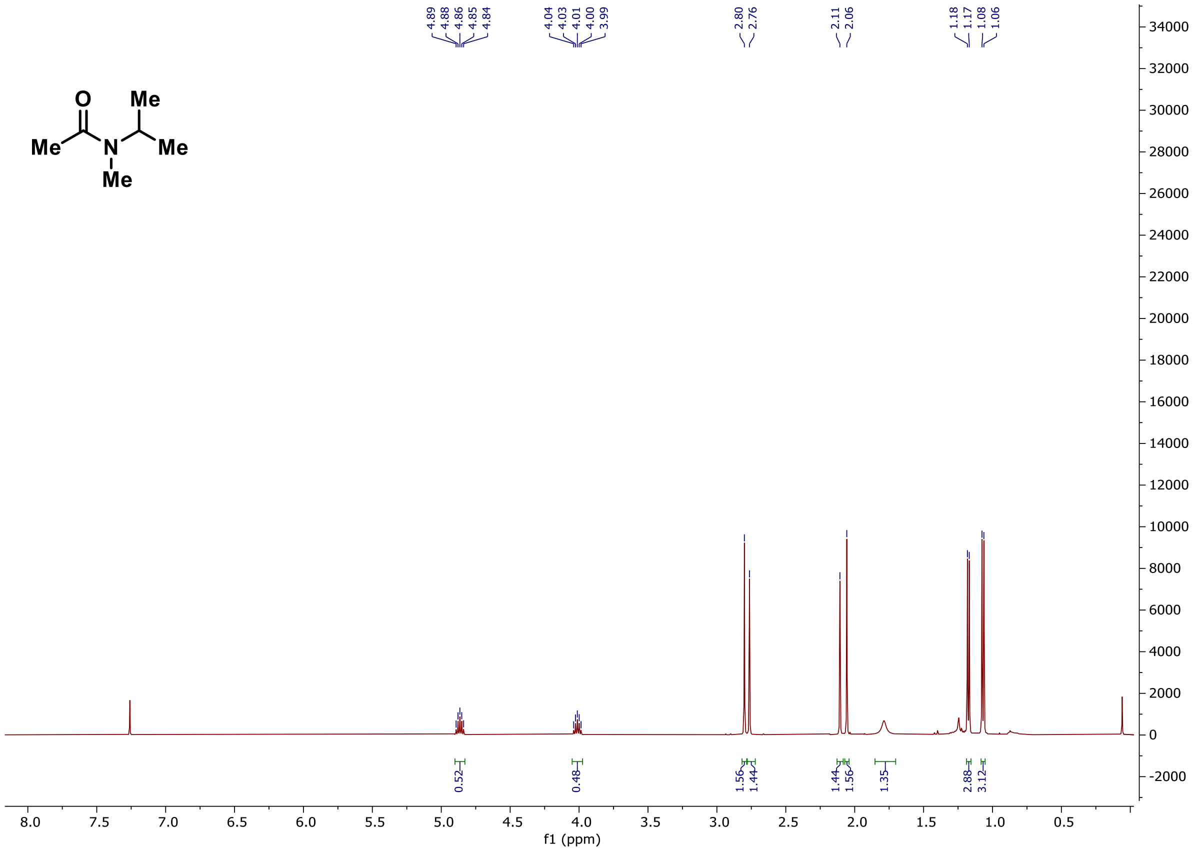
O
Me
Me
N
Me
Me
4.89
4.88
4.86
4.85
4.84
4.04
4.03
4.01
4.00
3.99
2.80
2.76
2.11
2.06
1.18
1.17
1.08
1.06
0.52
0.48
1.56
1.44
1.44
1.56
1.35
2.88
3.12
f1 (ppm)

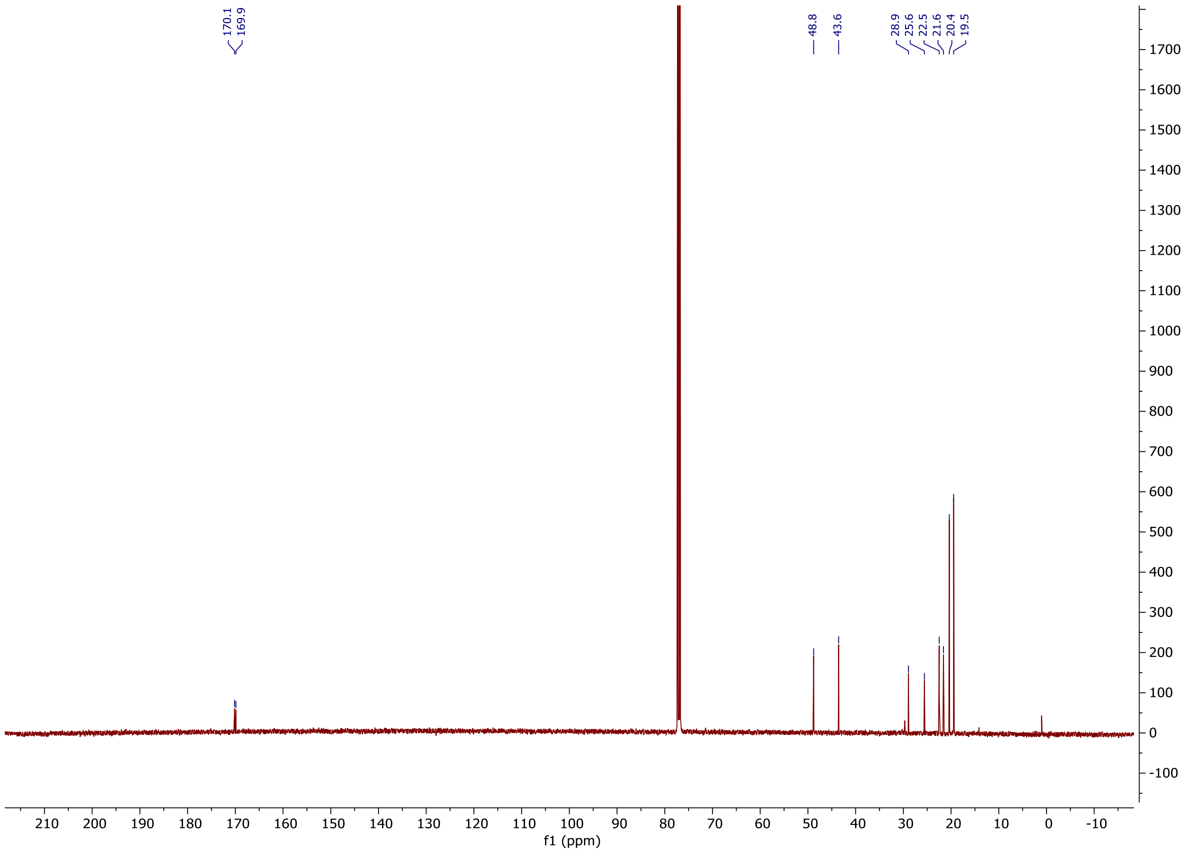
170.1
169.9
48.8
43.6
28.9
25.6
22.5
21.6
20.4
19.5
f1 (ppm)

6c

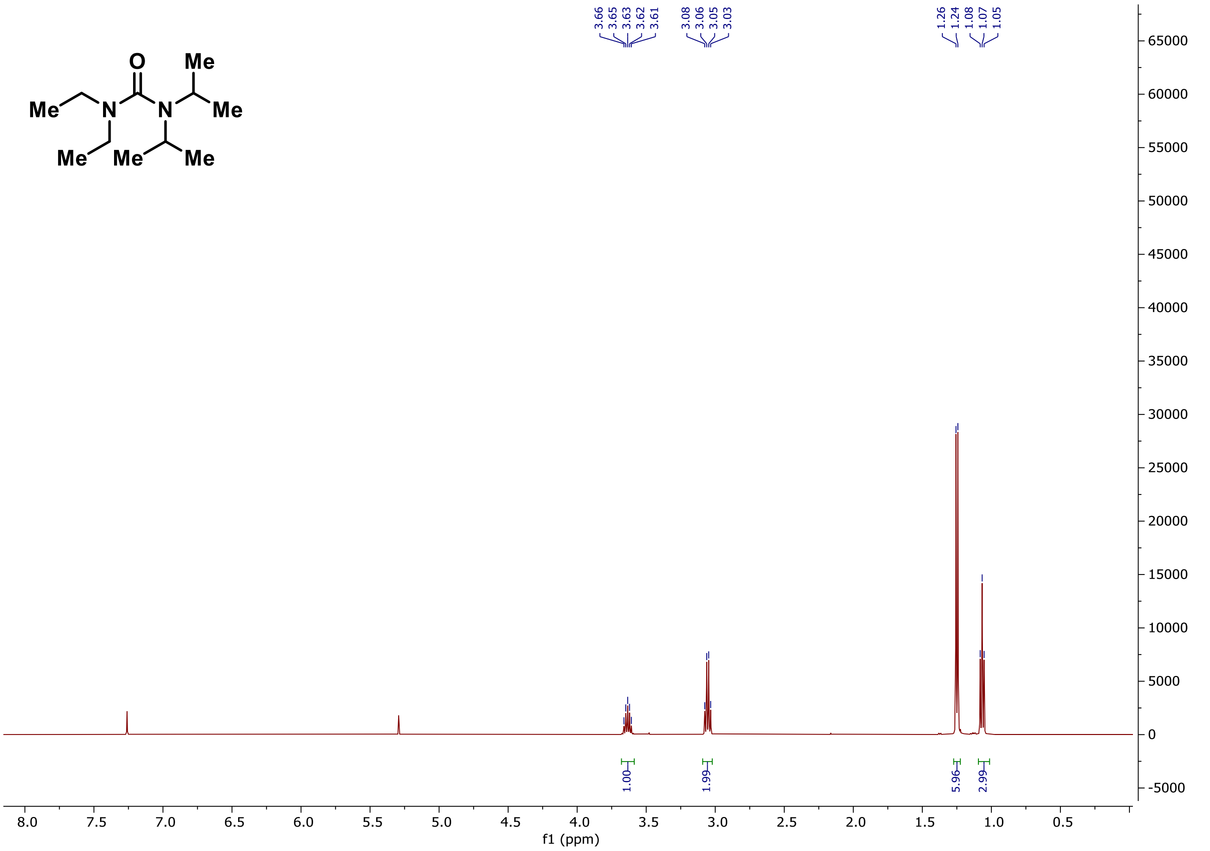
O
Me
Me
N
N
Me
Me
Me
Me
3.66
3.65
3.63
3.62
3.61
3.08
3.06
3.05
3.03
1.26
1.24
1.08
1.07
1.05
1.00
1.99
5.96
2.99
f1 (ppm)

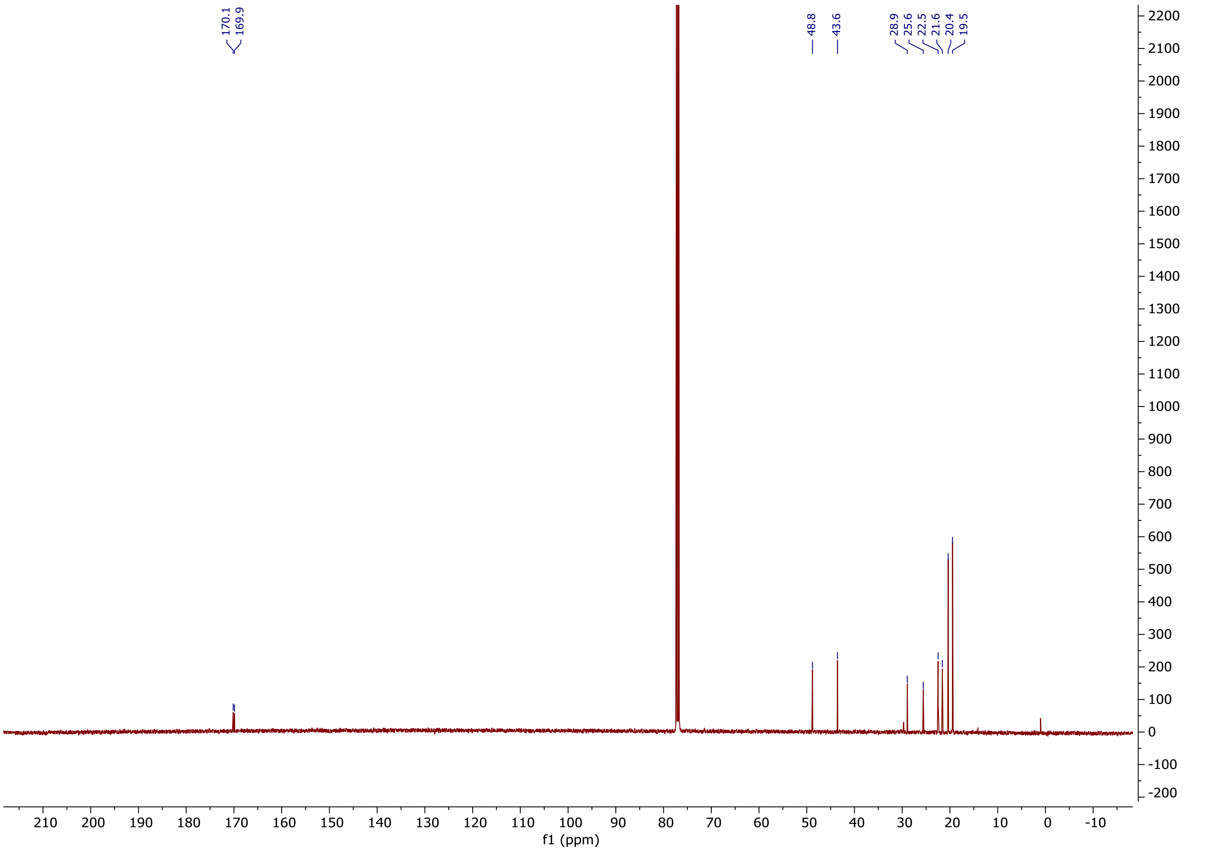
170.1
169.9
48.8
43.6
28.9
25.6
22.5
21.6
20.4
19.5
f1 (ppm)

6d

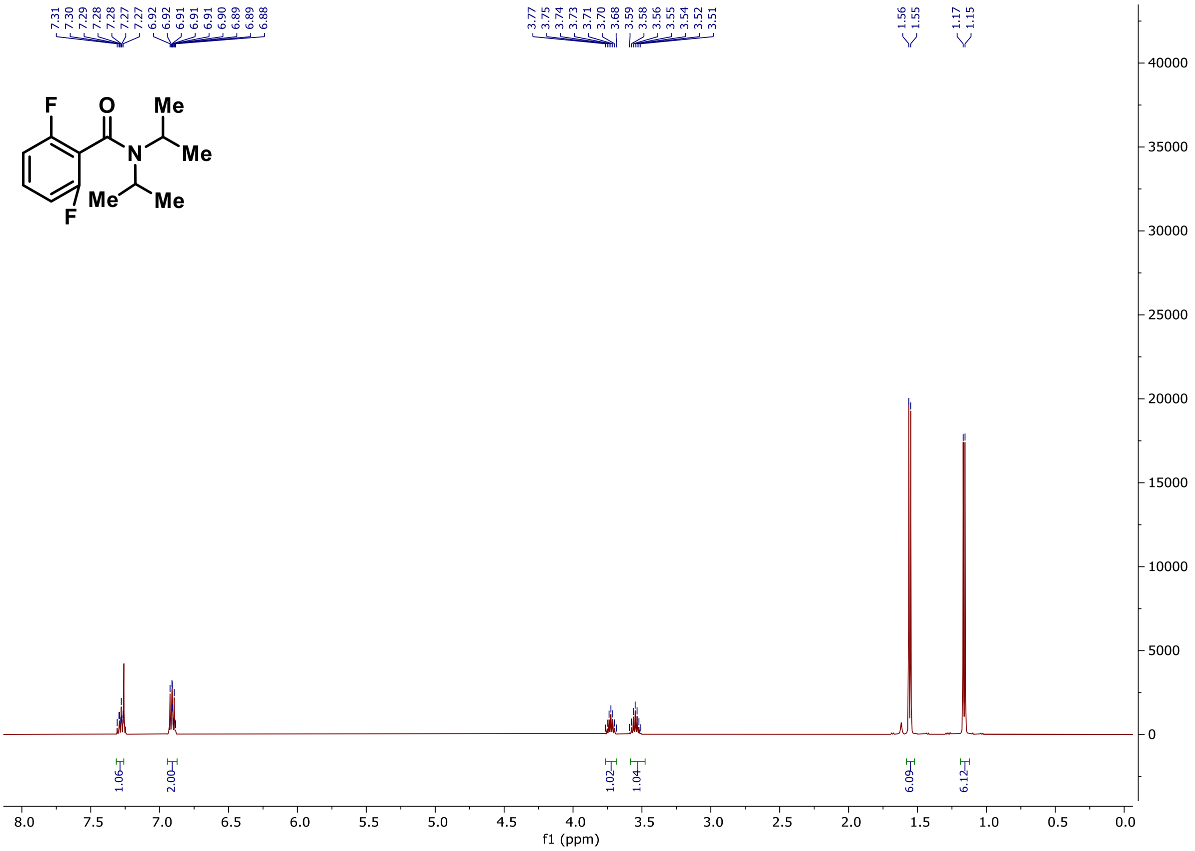
7.31
7.30
7.29
7.28
7.28
7.27
7.27
6.92
6.92
6.91
6.91
6.91
6.90
6.89
6.89
6.88
3.77
3.75
3.74
3.73
3.71
3.70
3.68
3.59
3.58
3.56
3.55
3.54
3.52
3.51
1.56
1.55
1.17
1.15
1.06
2.00
1.02
1.04
6.09
6.12
f1 (ppm)

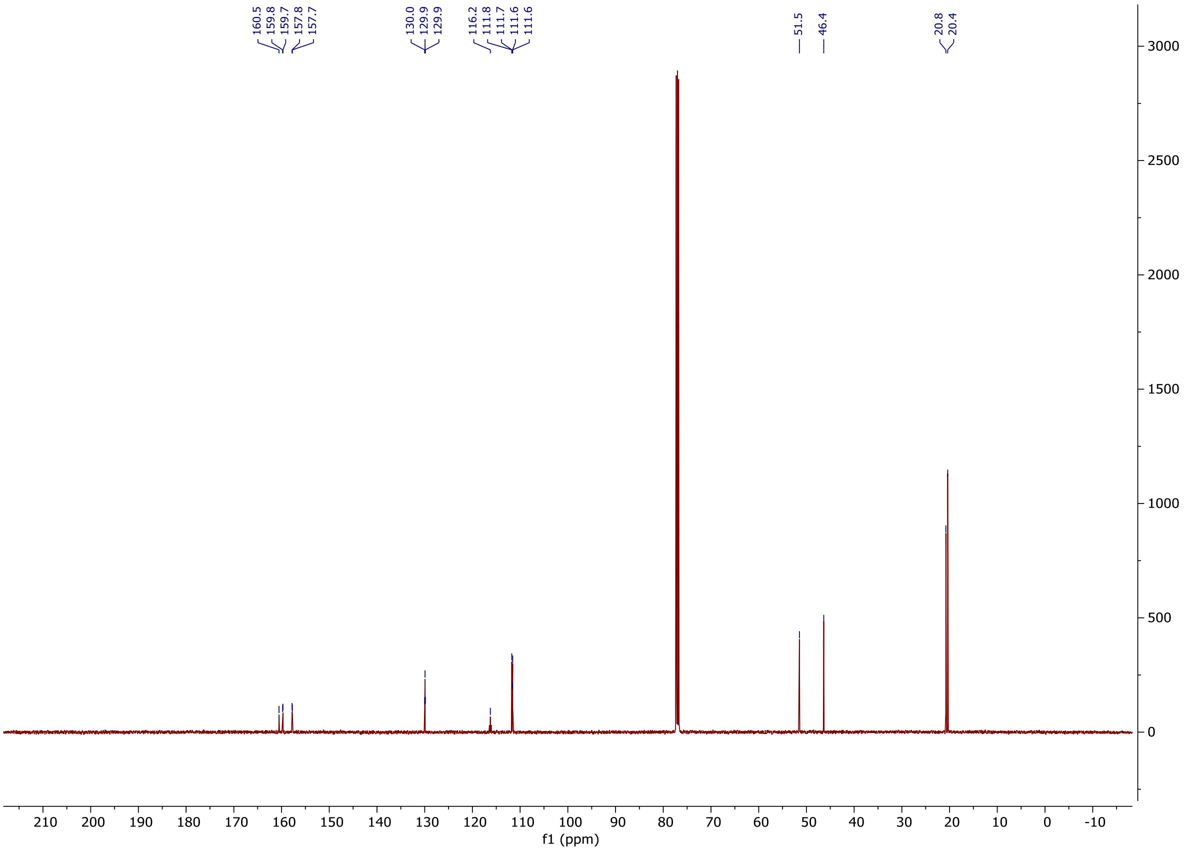
160.5
159.8
159.7
157.8
157.7
130.0
129.9
129.9
116.2
111.8
111.7
111.6
111.6
51.5
46.4
20.8
20.4
f1 (ppm)

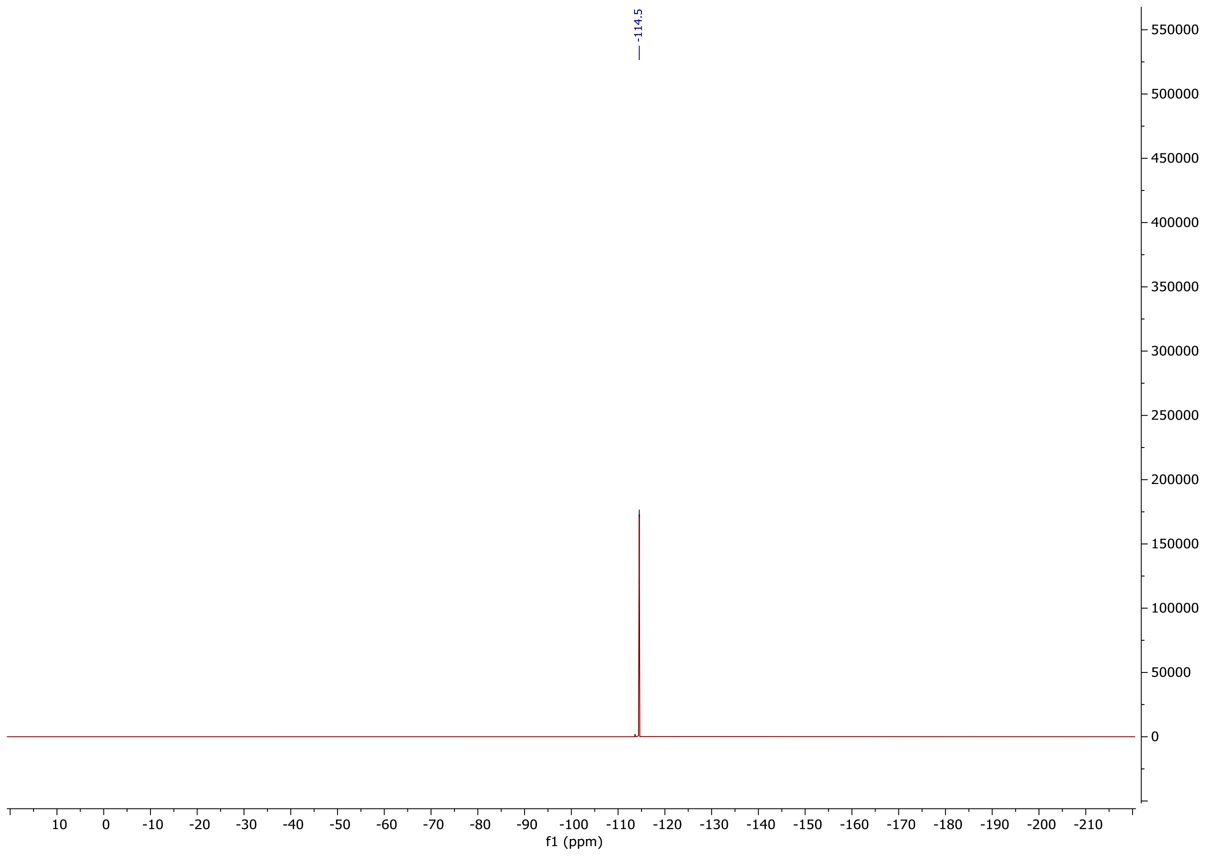
-114.5
550000
500000
450000
400000
350000
300000
250000
200000
150000
100000
50000
0
10 0 -10 -20 -30 -40 -50 -60 -70 -80 -90 -100 -110 -120 -130 -140 -150 -160 -170 -180 -190 -200 -210
f1 (ppm)

Intermolecular N→C Isopropyl Transfer: Substrates (Scheme 4C)

NH-1g

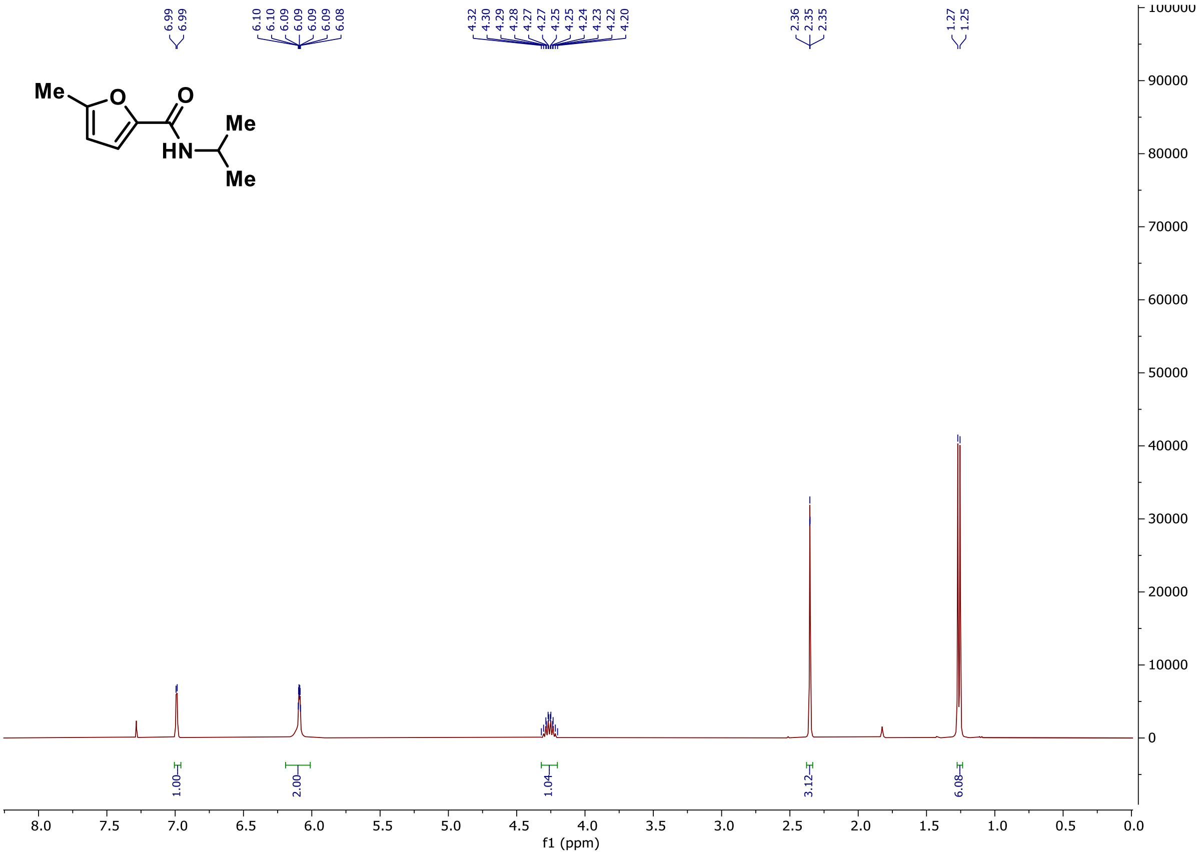

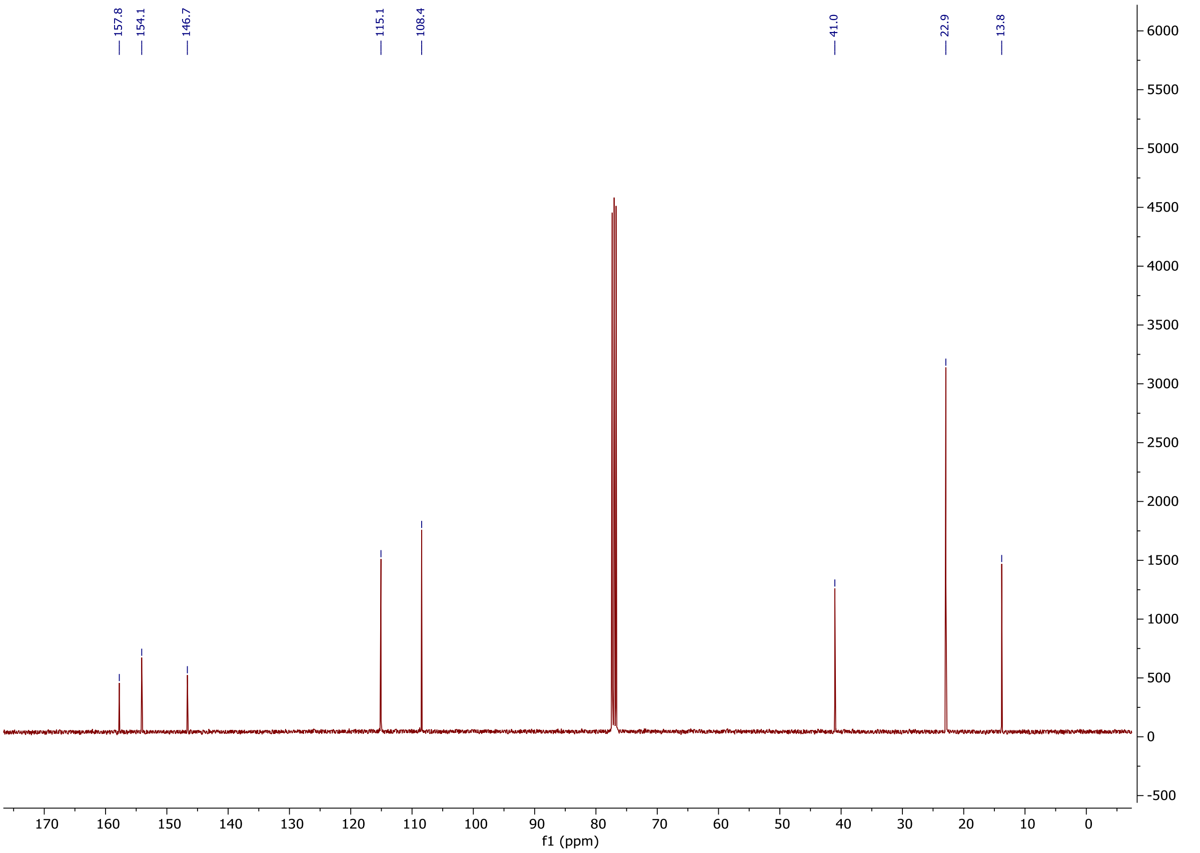

NH-1i

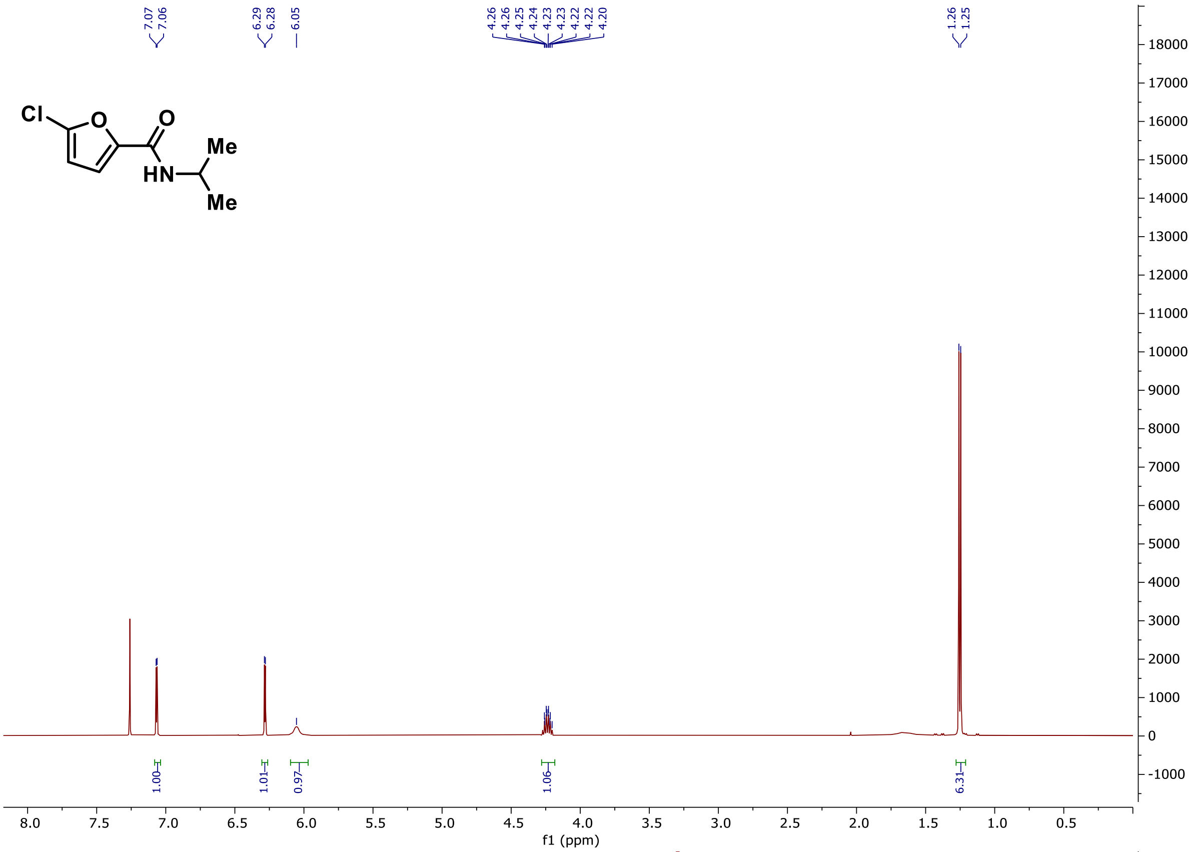

Cl
O
O
Me
HN
Me
7.07
7.06
6.29
6.28
6.05
4.26
4.26
4.25
4.24
4.23
4.23
4.22
4.22
4.20
1.26
1.25
1.00
1.01
0.97
1.06
6.31
f1 (ppm)

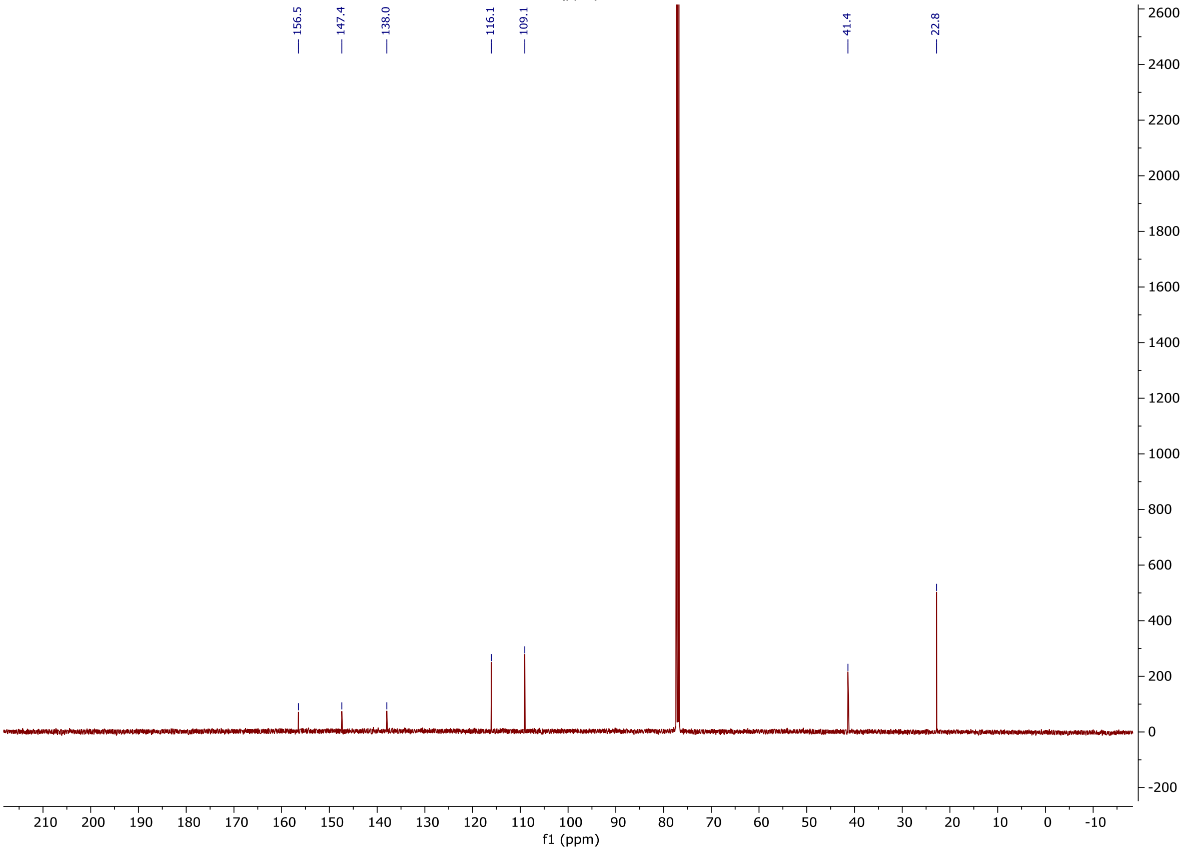

156.5
147.4
138.0
116.1
109.1
41.4
22.8
f1 (ppm)

1aa

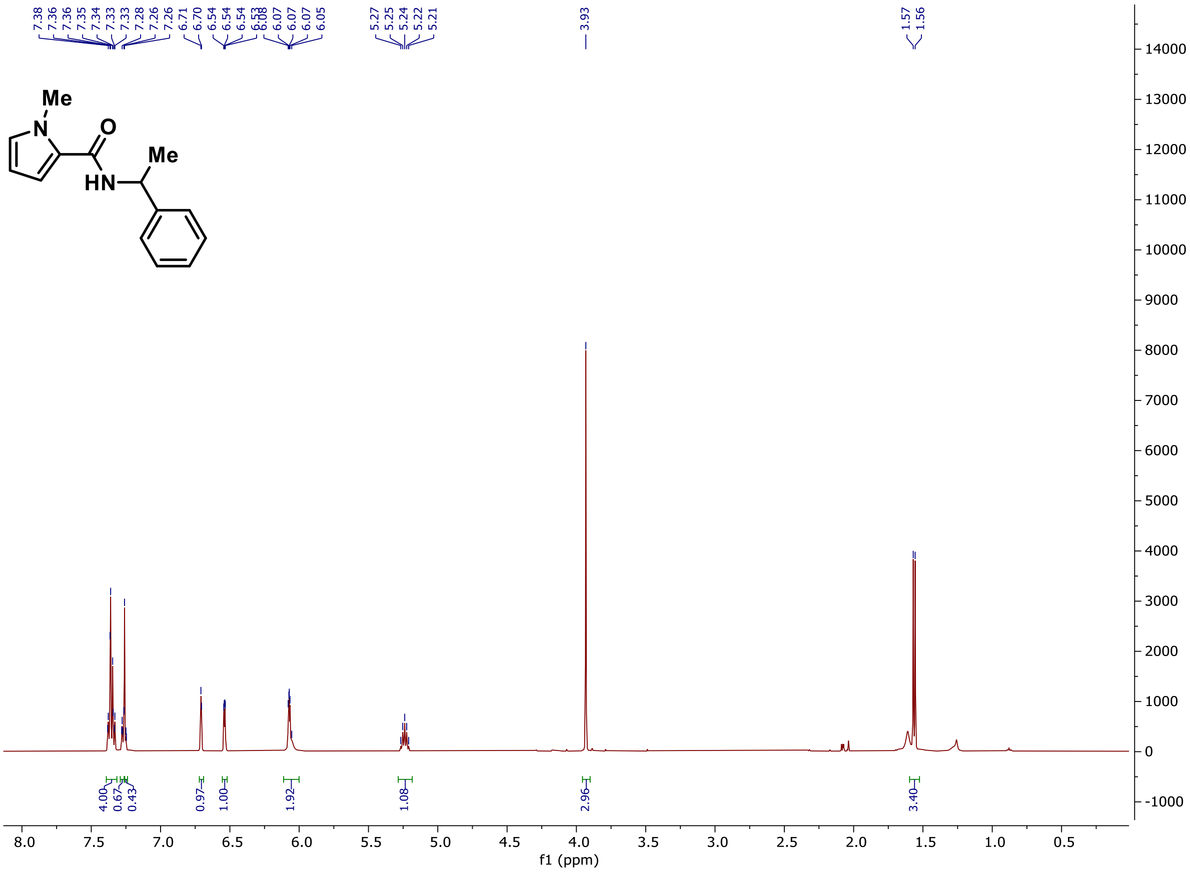

7.38 7.36 7.36 7.35 7.34 7.33 7.33 7.28 7.26 7.26 6.71 6.70 6.54 6.54 6.54 6.53 6.08 6.07 6.07 6.07 6.05
5.27 5.25 5.24 5.22 5.21
3.93
1.57 1.56
Me
N
O
Me
HN
4.00 0.67 0.43 0.97 1.00 1.92 1.08 2.96 3.40
f1 (ppm)

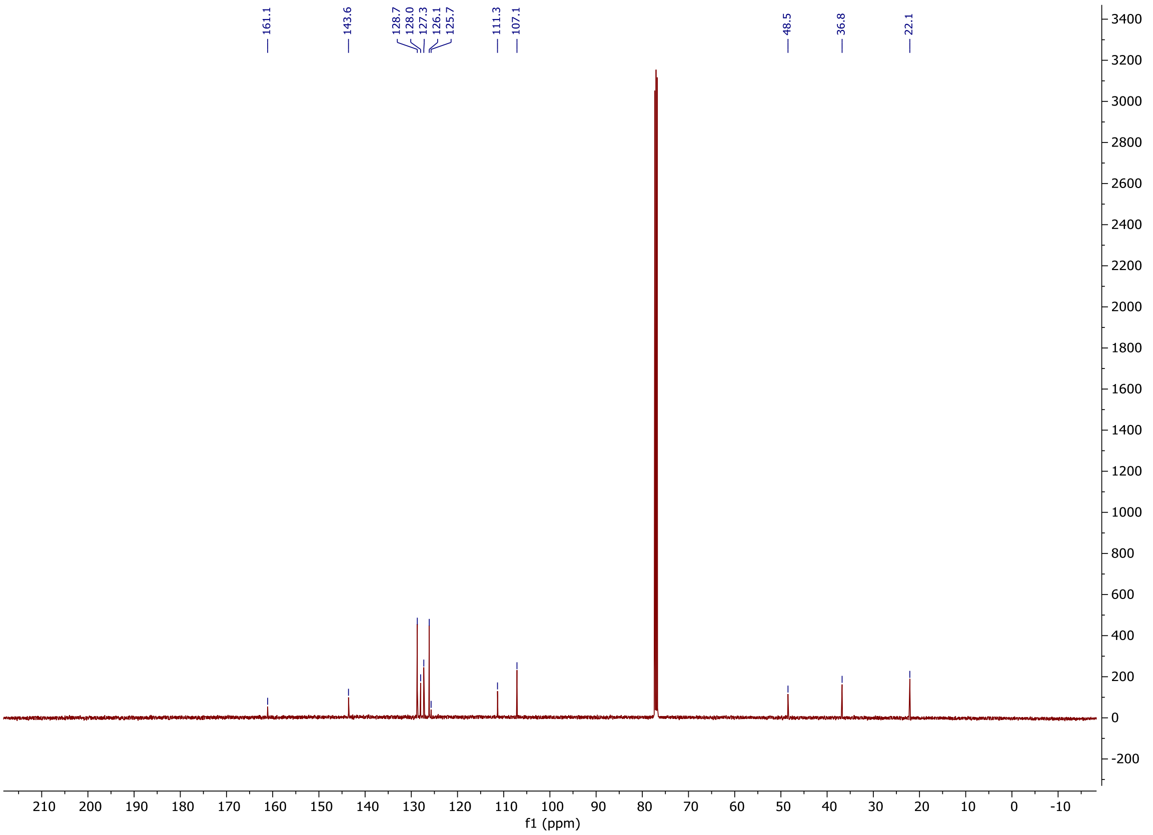

161.1
143.6
128.7 128.0 127.3 126.1 125.7
111.3 107.1
48.5
36.8
22.1
f1 (ppm)

1bb

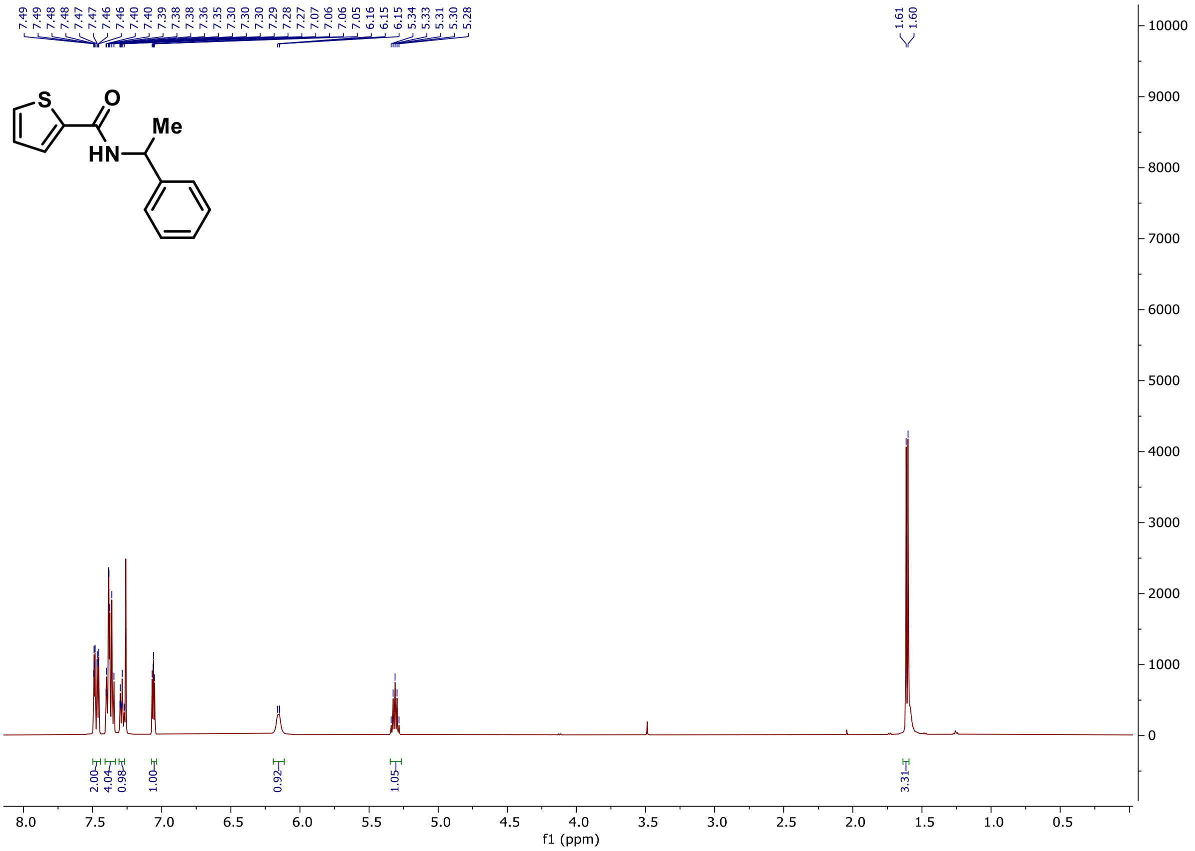
7.49 7.49 7.48 7.47 7.47 7.46 7.46 7.40 7.40 7.39 7.38 7.38 7.36 7.35 7.30 7.30 7.30 7.29 7.28 7.27 7.07 7.06 7.06 7.05 6.16 6.15 6.15 5.34 5.33 5.31 5.30 5.28
1.61 1.60
S
O
Me
HN
2.00 4.04 0.98 1.00 0.92 1.05 3.31
f1 (ppm)

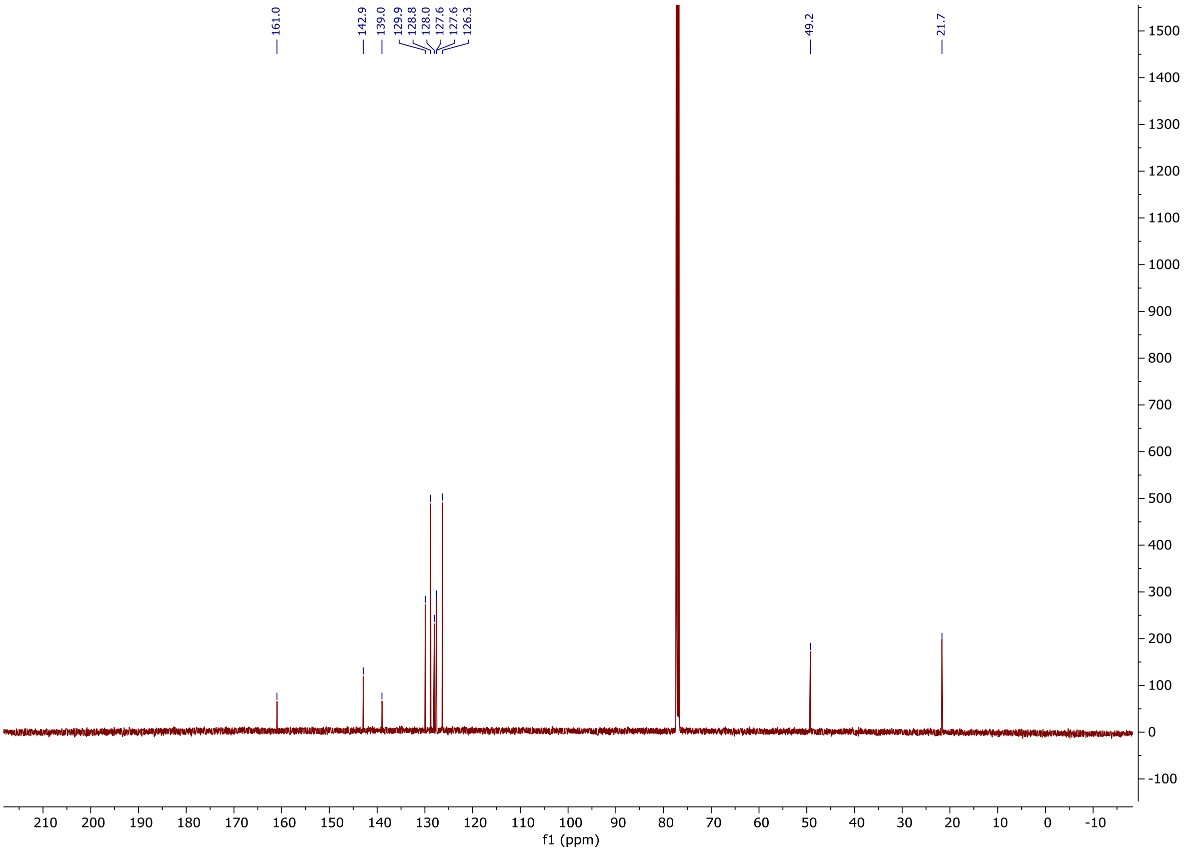
161.0 142.9 139.0 129.9 128.8 128.0 127.6 127.6 126.3 49.2 21.7
f1 (ppm)

Intermolecular N→C Isopropyl Transfer: Products (Scheme 4C)

4z

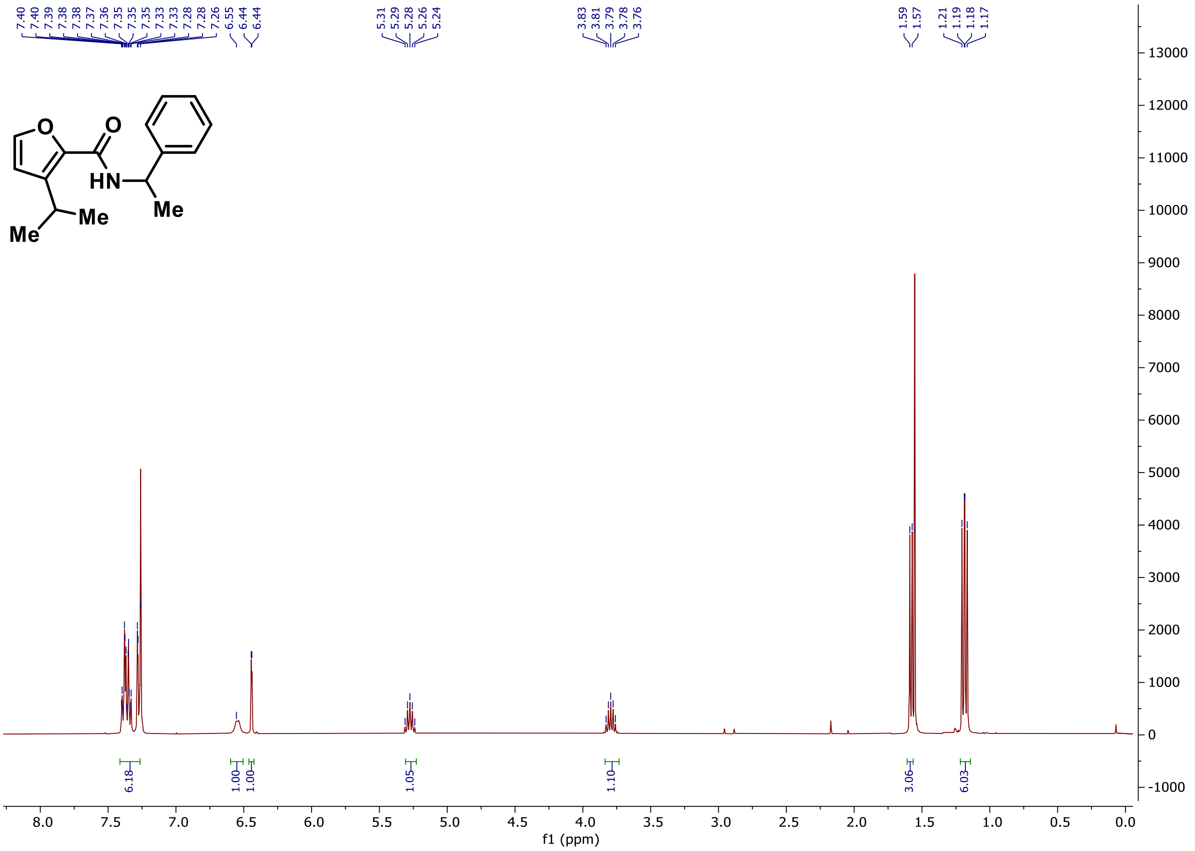

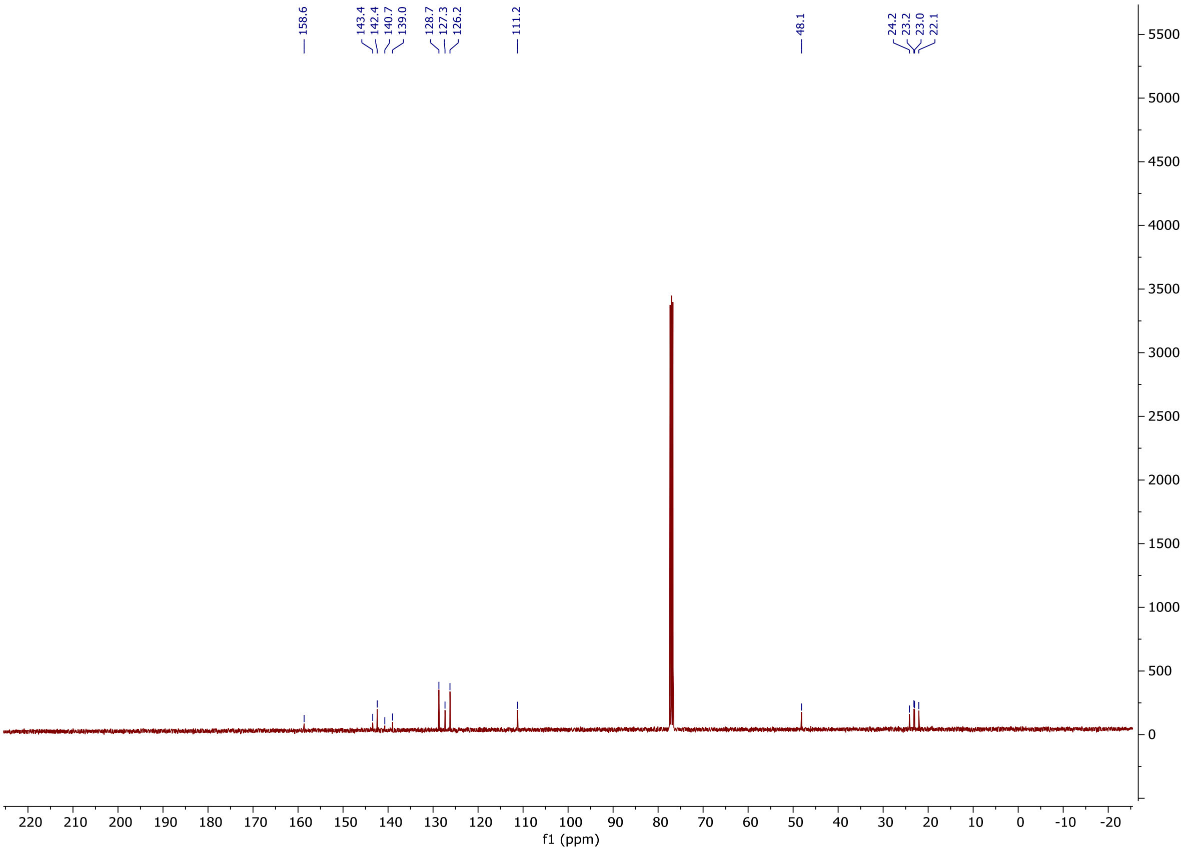

4aa

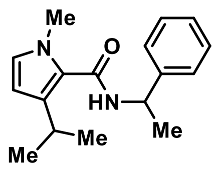
Me
N
O
HN
Me
Me
Me

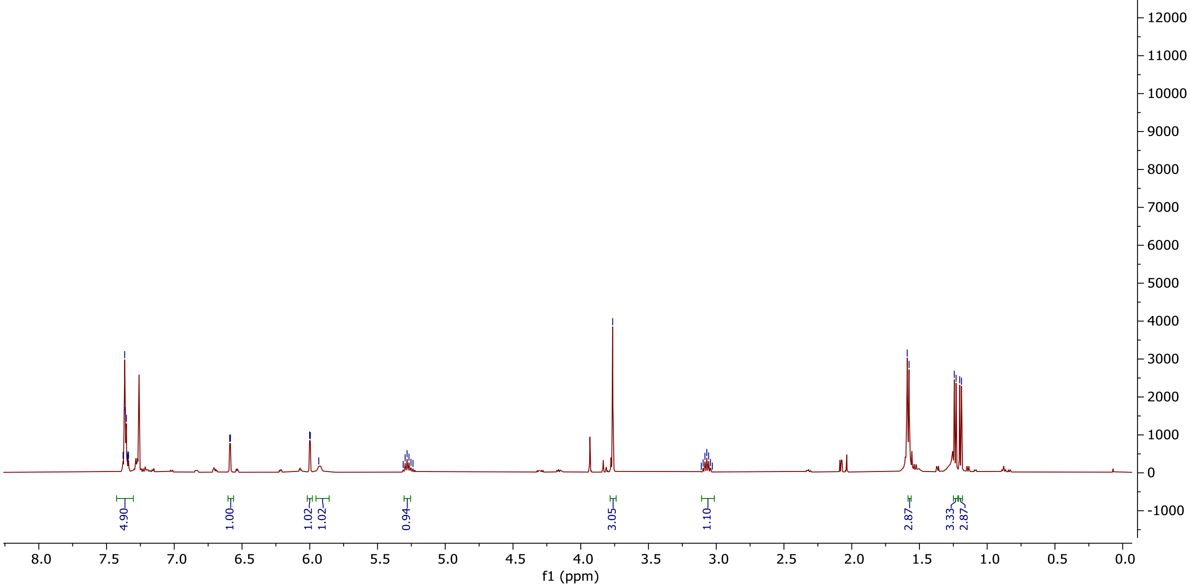
7.38 7.37 7.36 7.35 7.35 7.35 7.34 7.34
6.59 6.59
6.00 6.00 5.93
5.31 5.30 5.28 5.27 5.25 5.24
3.76
3.11 3.10 3.08 3.07 3.06 3.04 3.03
1.59 1.58
1.24 1.23 1.20 1.19
4.90
1.00
1.02
1.02
0.94
3.05
1.10
2.87
3.33
2.87
f1 (ppm)

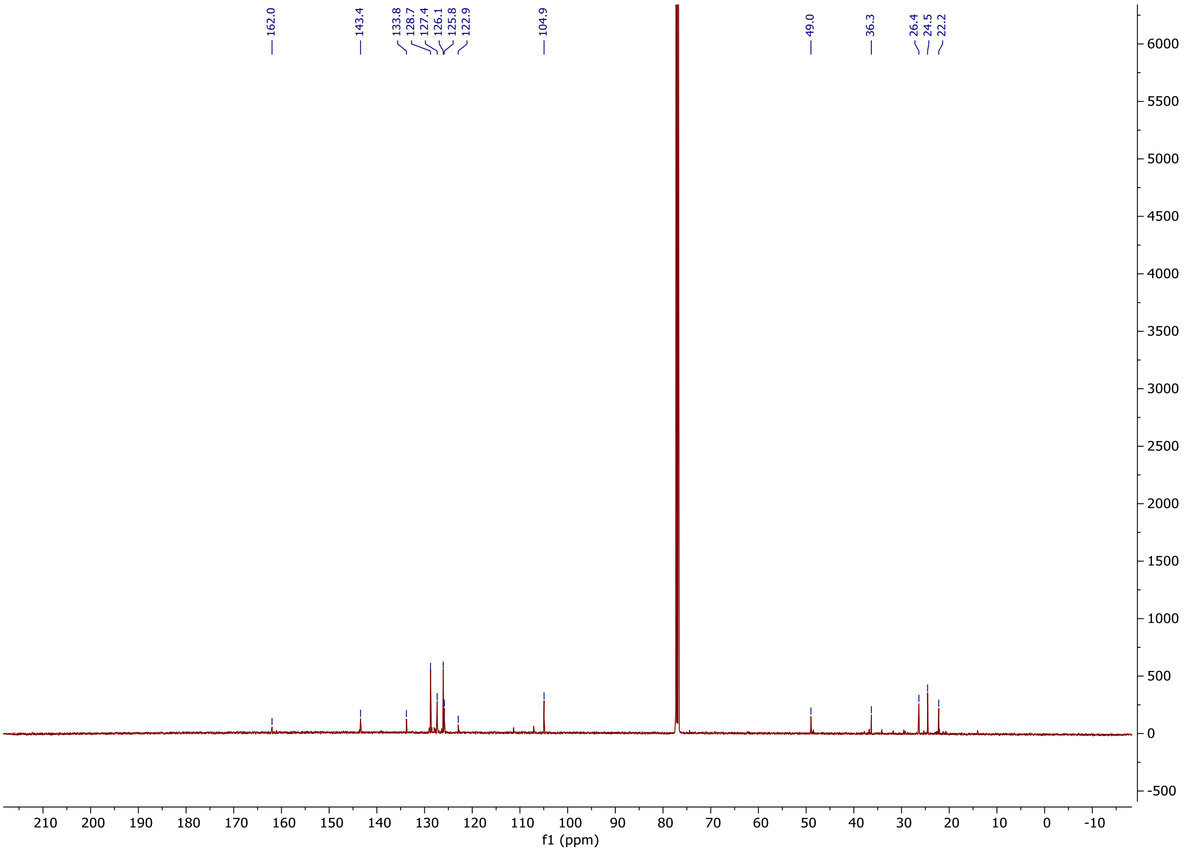
162.0
143.4
133.8 128.7 127.4 126.1 125.8 122.9
104.9
49.0
36.3
26.4 24.5 22.2
f1 (ppm)

4bb

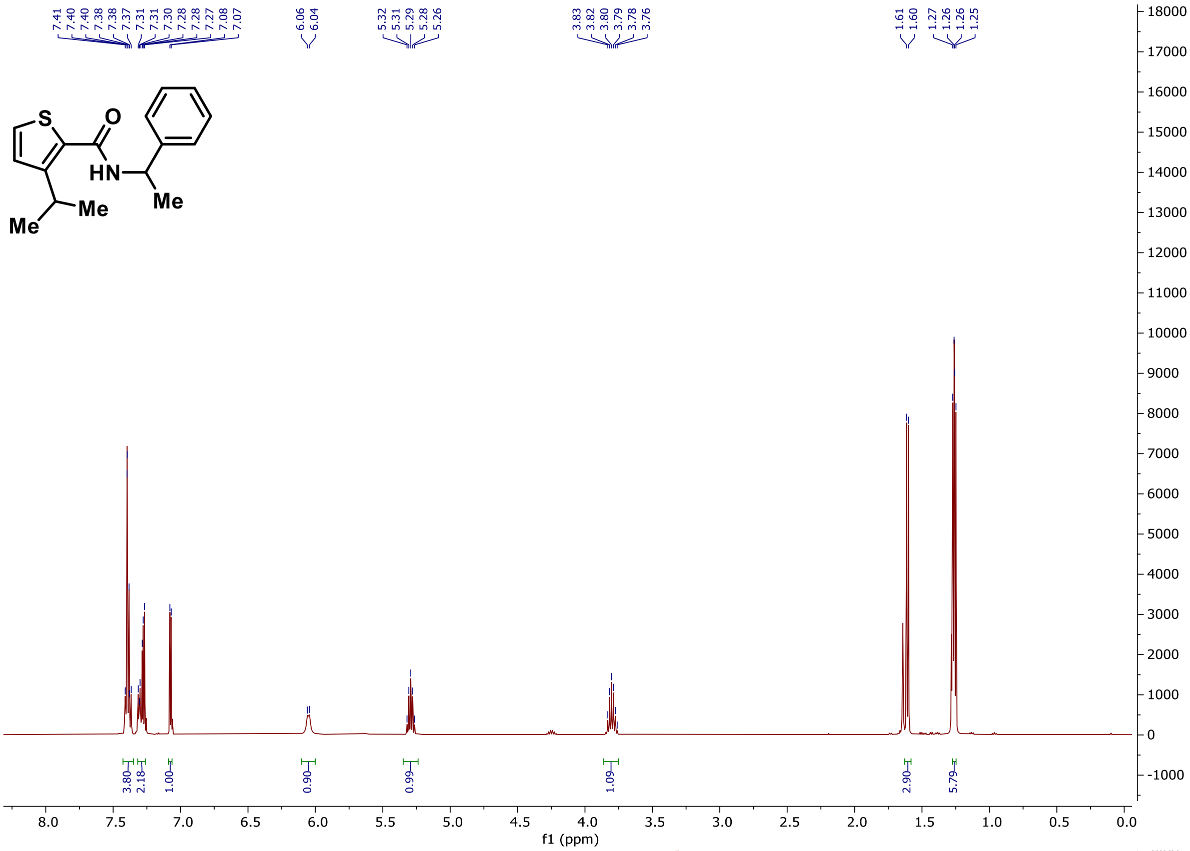

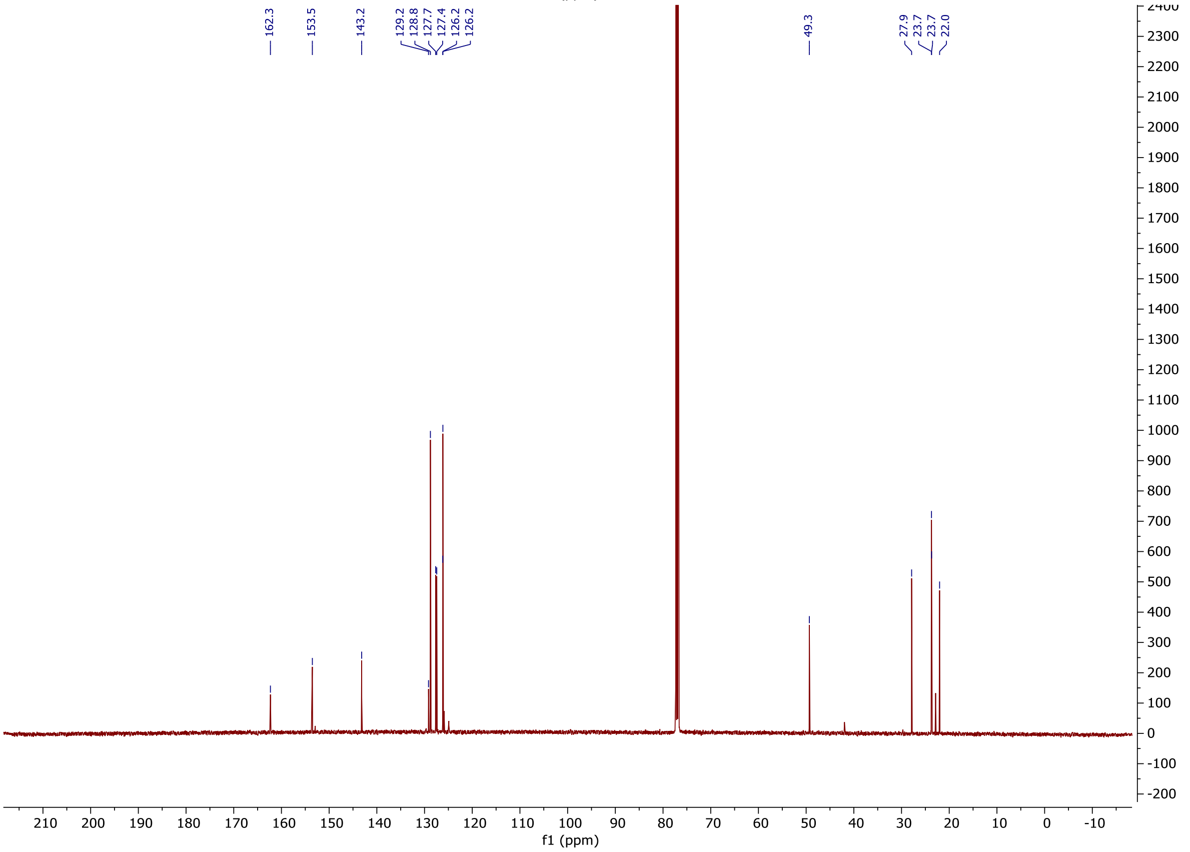

<u>Intermolecular N→C *sec*-Butyl Transfer (Scheme 4D)</u>

6f

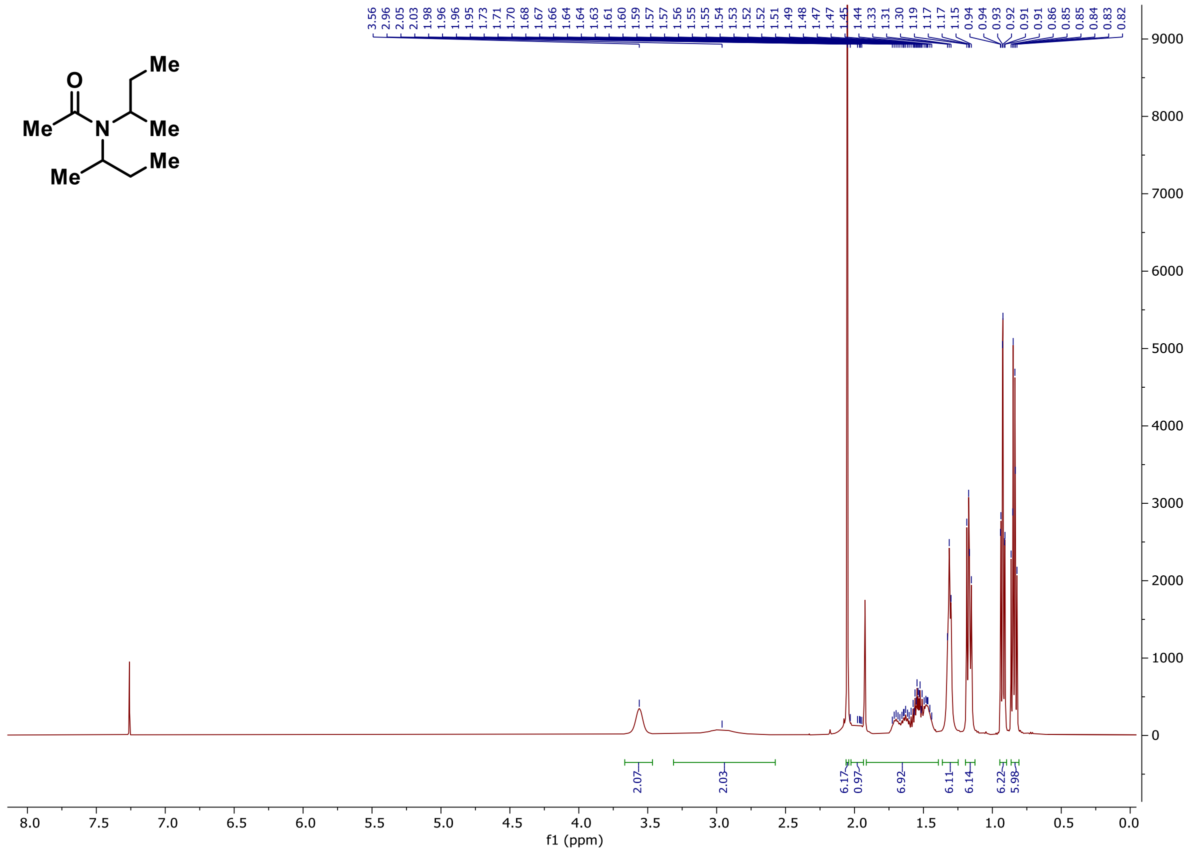

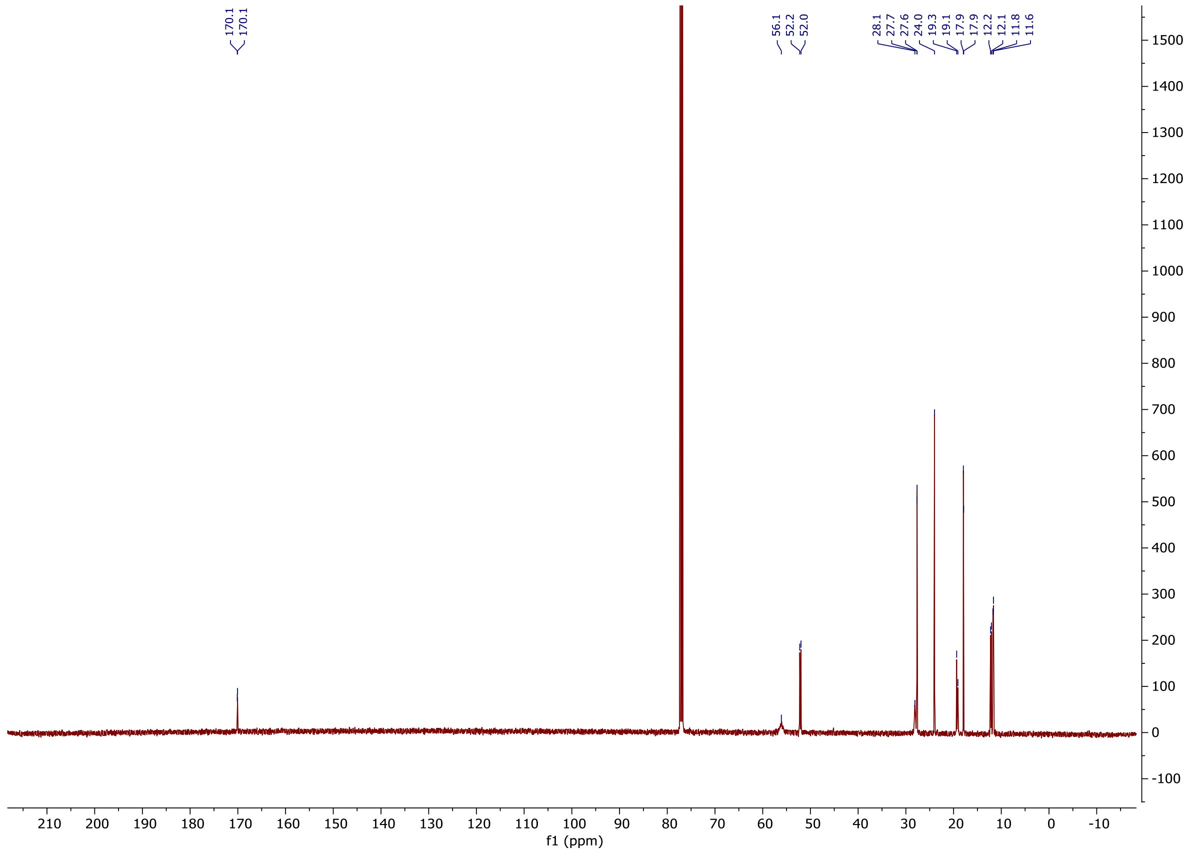

4z’

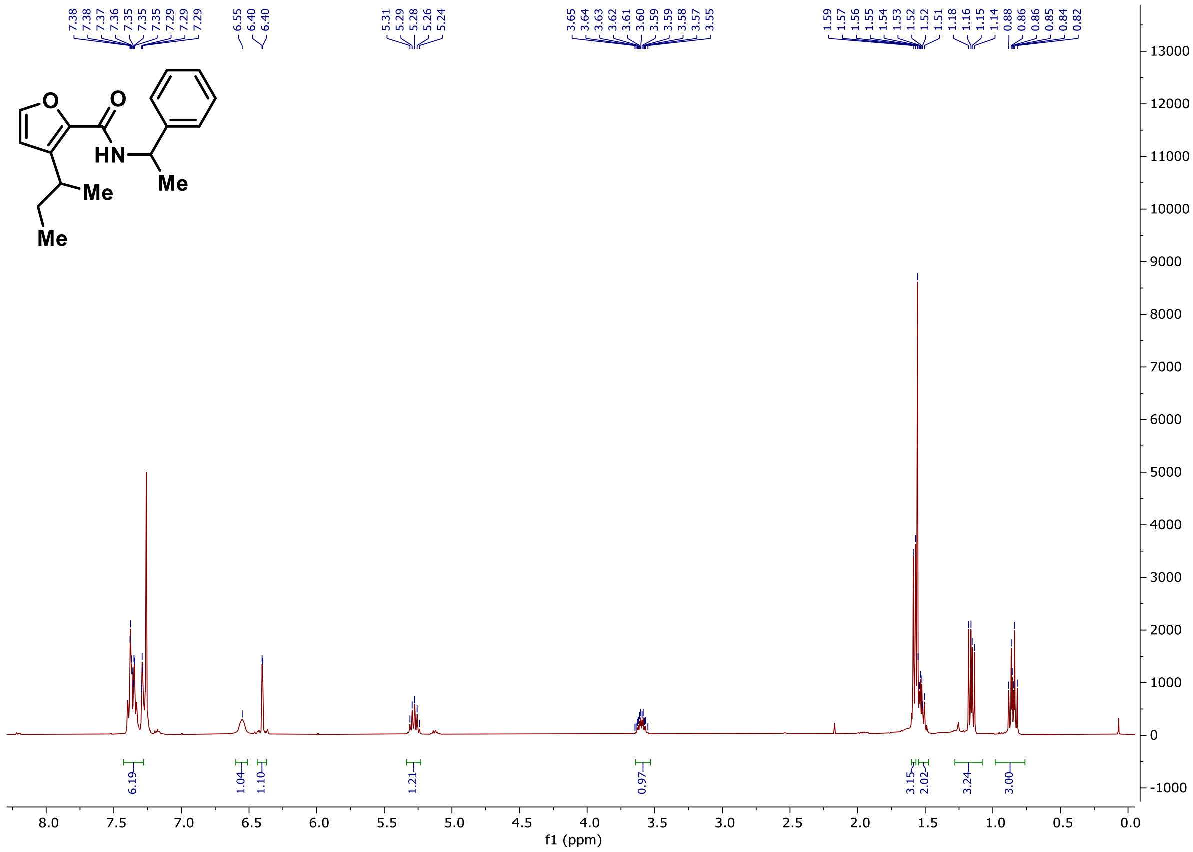
O
O
HN
Me
Me
Me
7.38
7.38
7.37
7.36
7.35
7.35
7.35
7.29
7.29
7.29
6.55
6.40
6.40
5.31
5.29
5.28
5.26
5.24
3.65
3.64
3.63
3.62
3.61
3.60
3.59
3.59
3.58
3.57
3.55
1.59
1.57
1.56
1.55
1.54
1.53
1.52
1.52
1.51
1.18
1.16
1.15
1.14
0.88
0.86
0.86
0.85
0.84
0.82
6.19
1.04
1.10
1.21
0.97
3.15
2.02
3.24
3.00
f1 (ppm)

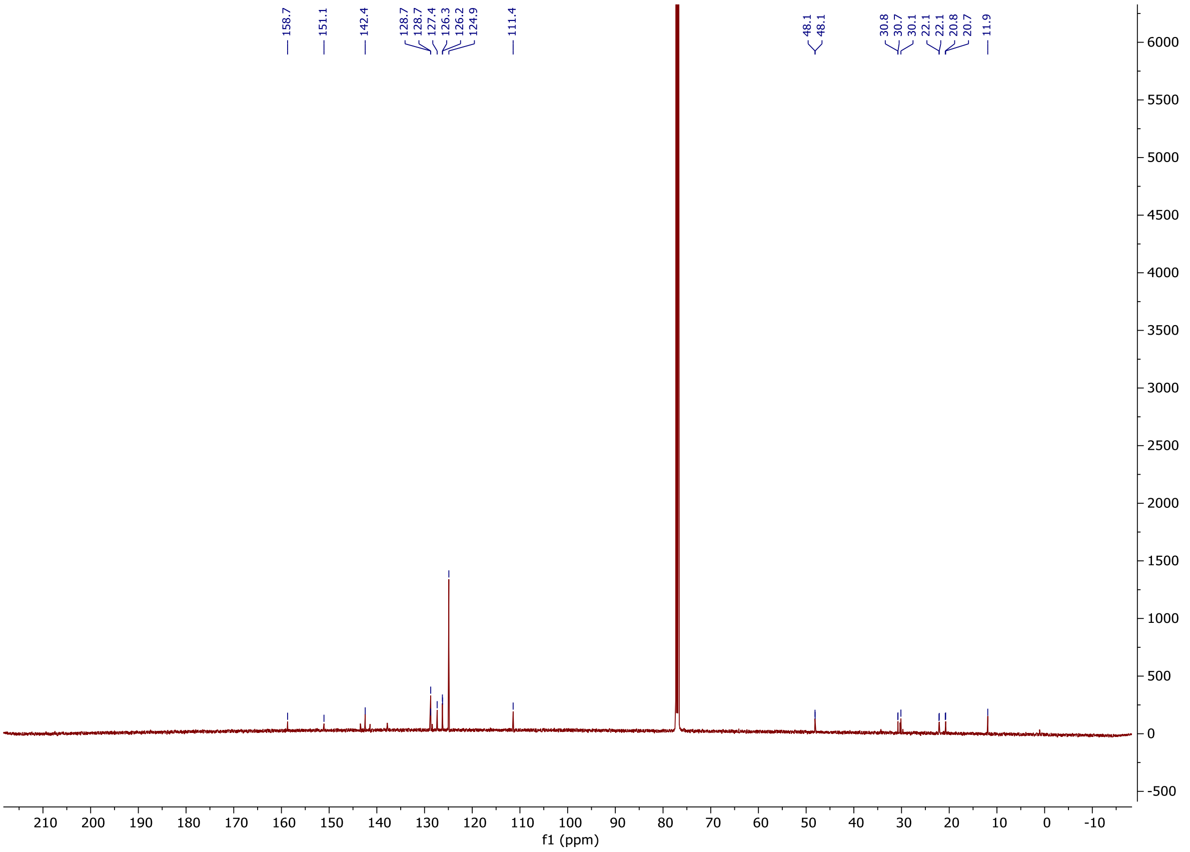
158.7
151.1
142.4
128.7
128.7
127.4
126.3
126.2
124.9
111.4
48.1
48.1
30.8
30.7
30.1
22.1
22.1
20.8
20.7
11.9
f1 (ppm)

Application to the Synthesis of an Antiparasitic Compound (Scheme 4E)

9

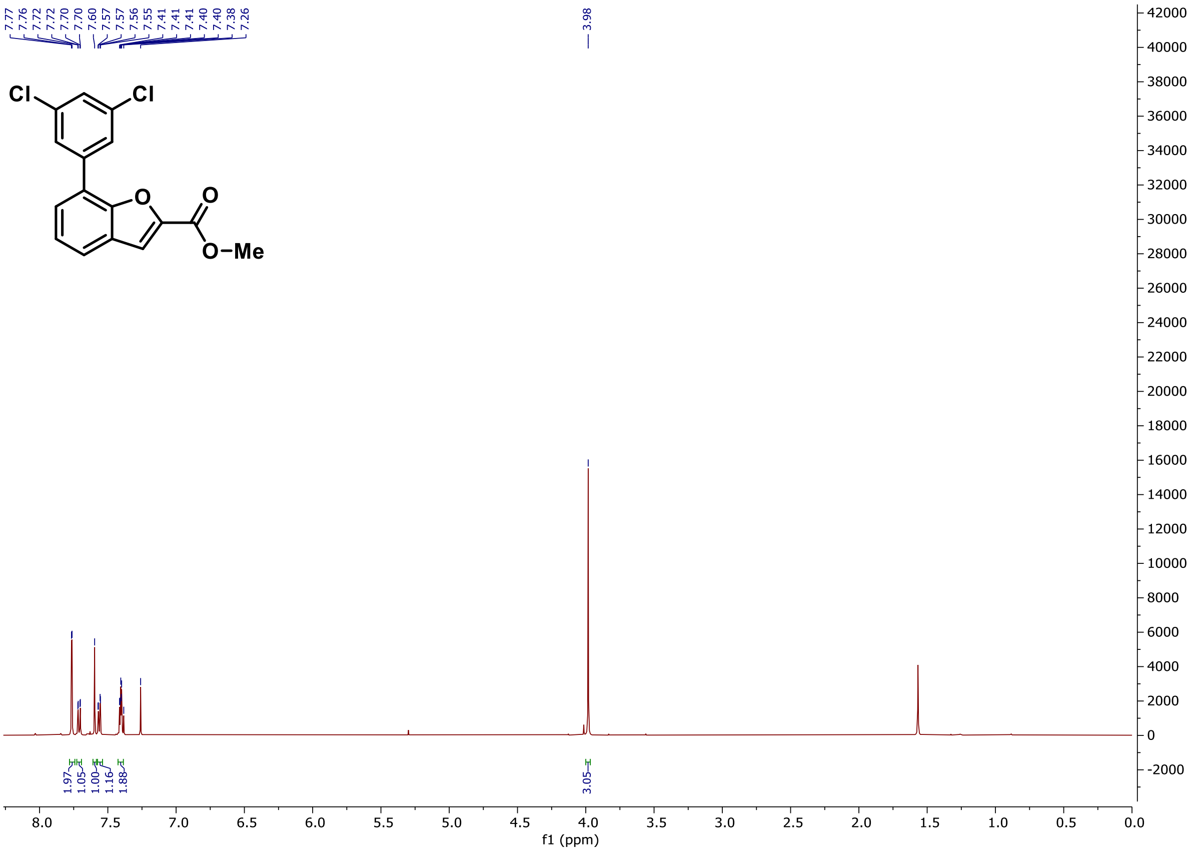

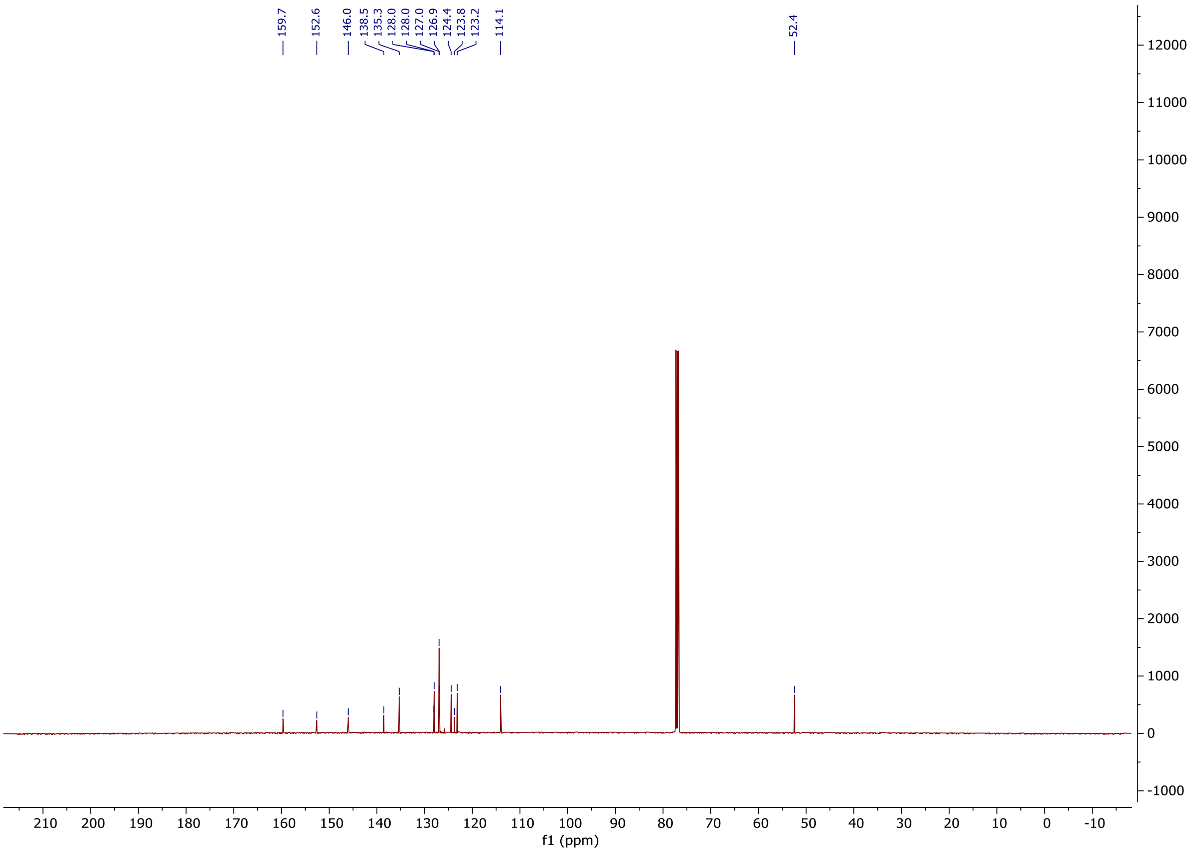

12

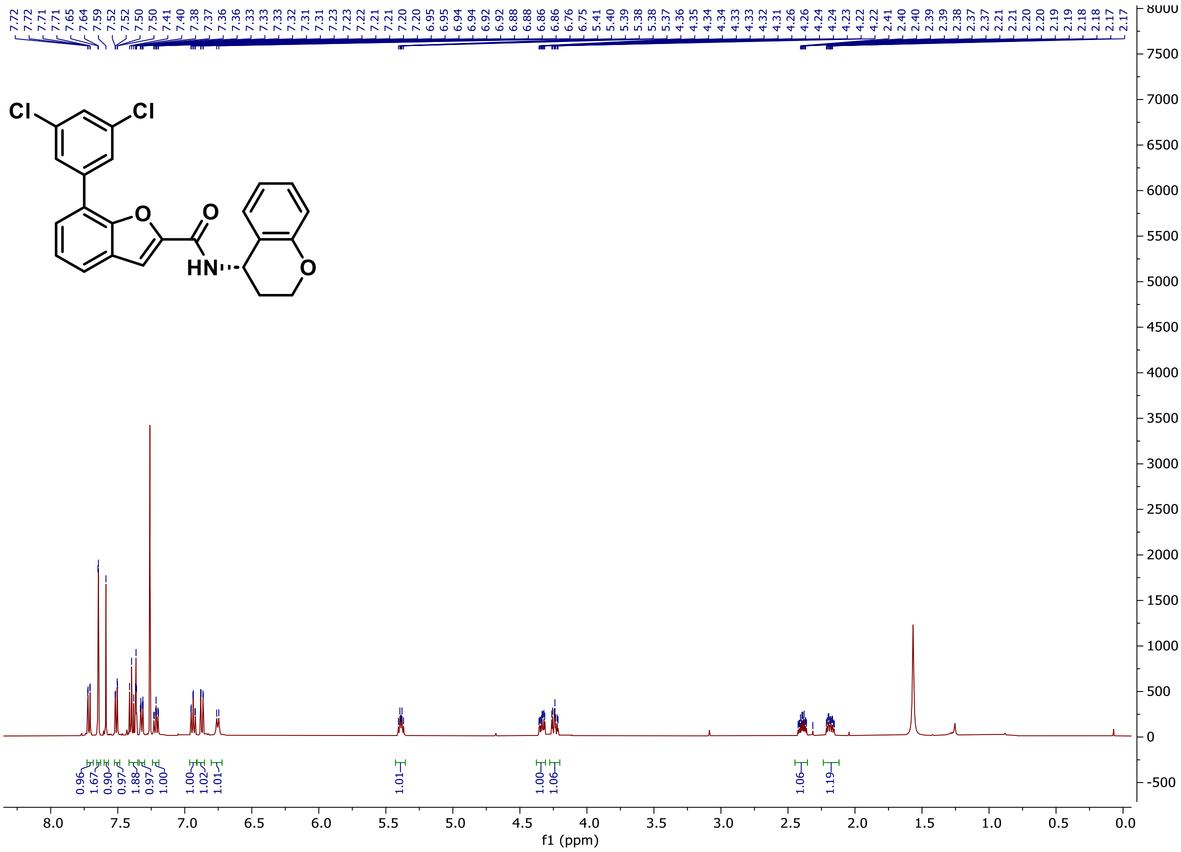

Cl
Cl
O
O
HN
O
7.72 7.72 7.71 7.65 7.64 7.59 7.52 7.52 7.50 7.50 7.41 7.40 7.38 7.37 7.36 7.36 7.33 7.33 7.33 7.32 7.31 7.31 7.23 7.23 7.22 7.21 7.21 7.20 7.20 6.95 6.95 6.94 6.94 6.92 6.92 6.88 6.86 6.86 6.76 6.75 5.41 5.40 5.39 5.38 5.38 5.37 4.36 4.35 4.34 4.34 4.33 4.32 4.31 4.26 4.26 4.24 4.24 4.23 4.22 4.22 2.41 2.40 2.40 2.39 2.39 2.38 2.37 2.37 2.21 2.21 2.20 2.19 2.19 2.18 2.17 2.17
0.96 1.67 0.90 0.97 1.88 0.97 1.00 1.00 1.02 1.01 1.01 1.00 1.06 1.06 1.19
f1 (ppm)

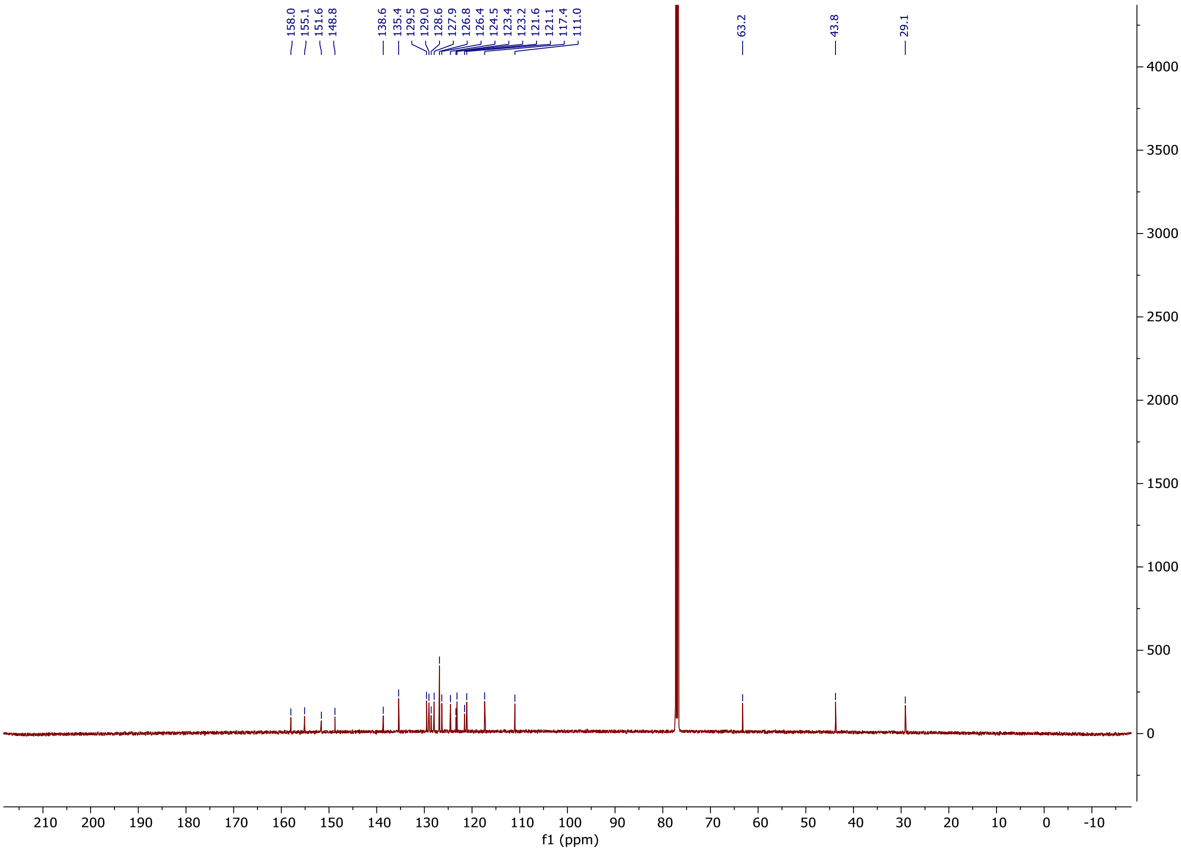

158.0 155.1 151.6 148.8 138.6 135.4 129.5 129.0 128.6 127.9 126.8 126.4 124.5 123.4 123.2 121.6 121.1 117.4 111.0 63.2 43.8 29.1
f1 (ppm)

13

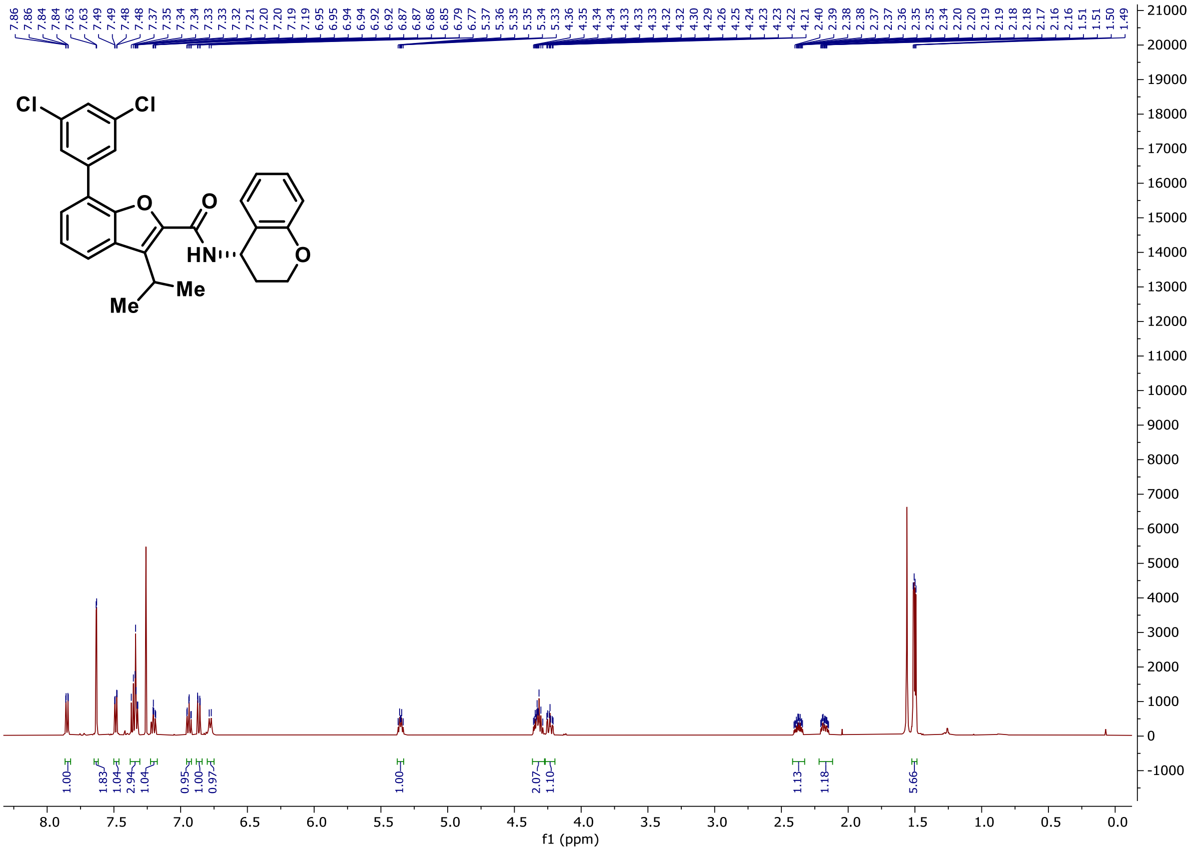
Cl
Cl
O
O
HN
O
Me
Me
7.86
7.86
7.84
7.63
7.63
7.49
7.49
7.48
7.37
7.35
7.34
7.34
7.33
7.33
7.32
7.21
7.20
7.19
7.19
6.95
6.95
6.94
6.94
6.92
6.92
6.87
6.87
6.86
6.85
6.79
6.77
5.37
5.36
5.35
5.35
5.34
5.33
4.36
4.35
4.34
4.34
4.33
4.33
4.33
4.32
4.30
4.29
4.26
4.25
4.24
4.23
4.22
4.21
2.40
2.39
2.38
2.38
2.37
2.37
2.36
2.35
2.35
2.34
2.20
2.20
2.19
2.19
2.18
2.18
2.17
2.16
1.51
1.51
1.50
1.49
1.00
1.83
1.04
2.94
1.04
0.95
1.00
0.97
1.00
2.07
1.10
1.13
1.18
5.66
f1 (ppm)

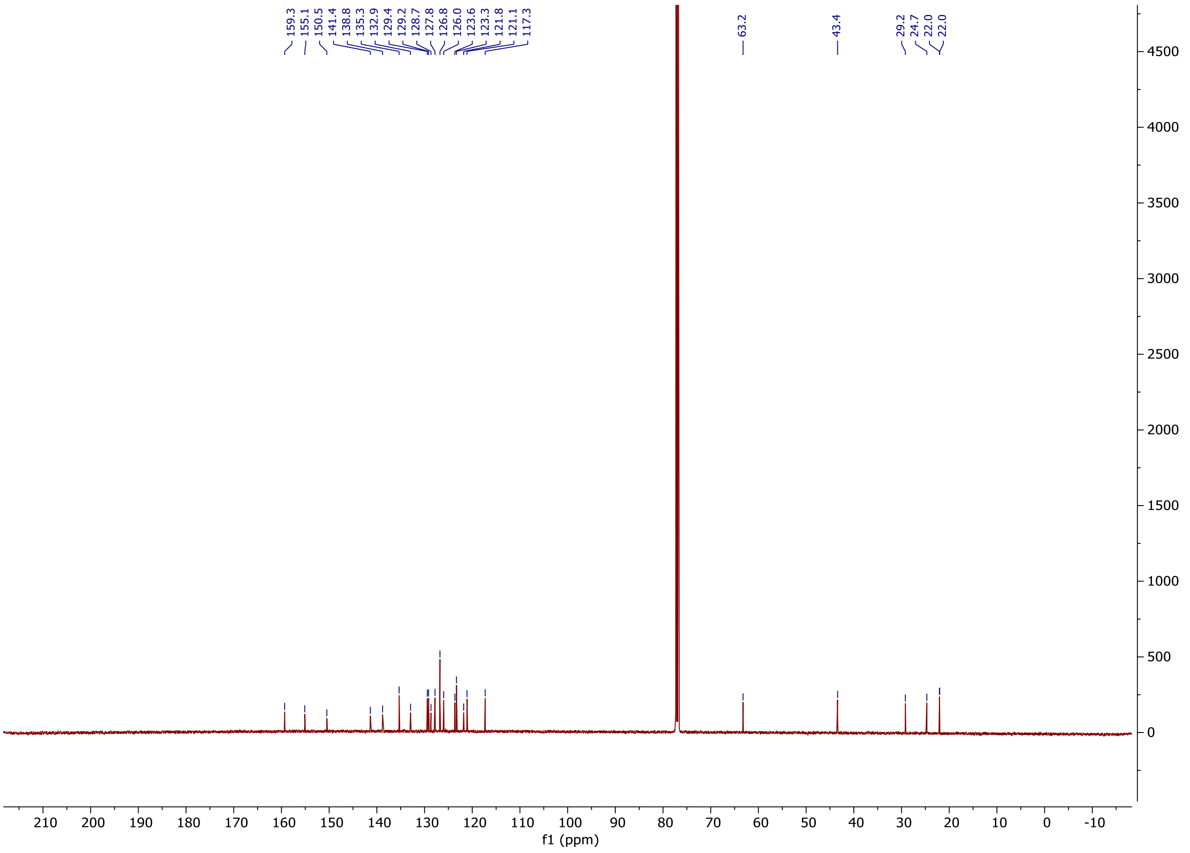
159.3
155.1
150.5
141.4
138.8
135.3
132.9
129.4
129.2
128.7
127.8
126.8
126.0
123.6
123.3
121.8
121.1
117.3
63.2
43.4
29.2
24.7
22.0
22.0
f1 (ppm)

S8

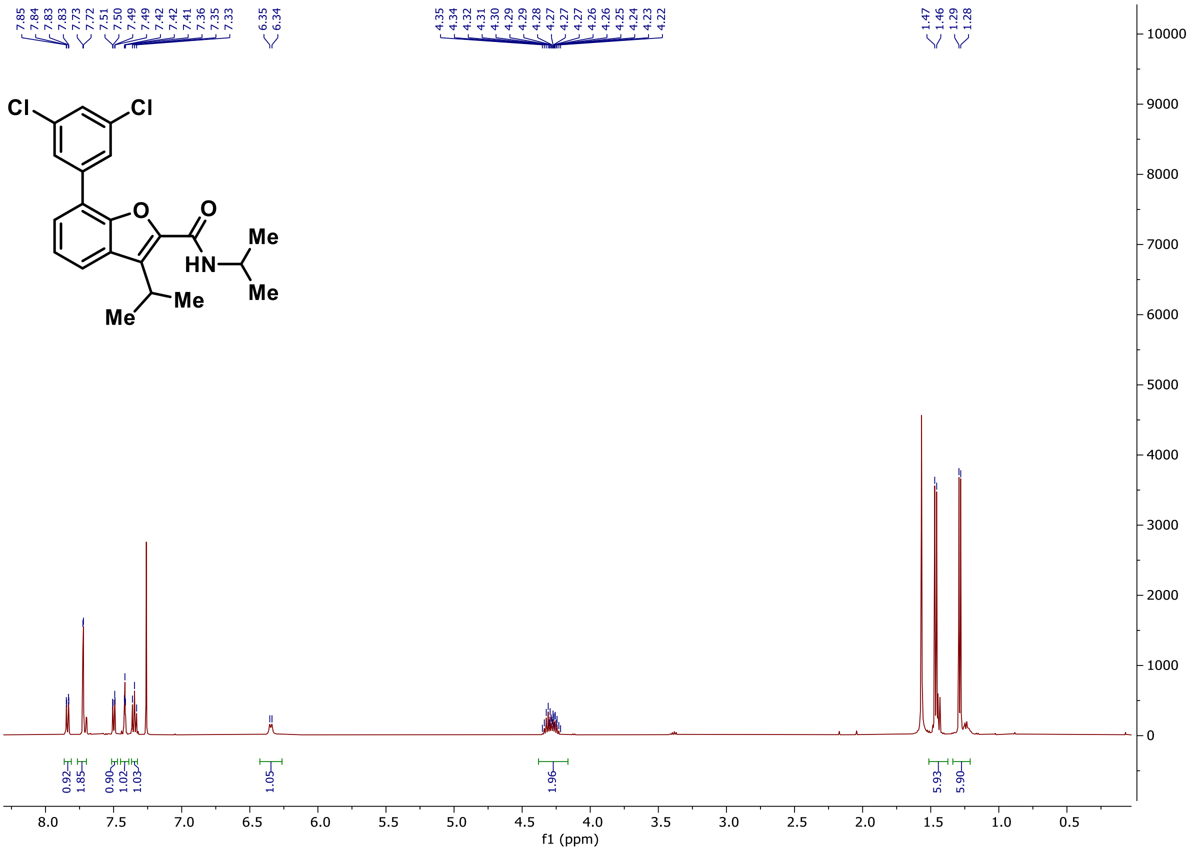
Cl
Cl
O
O
HN
Me
Me
Me
Me
7.85 7.84 7.83 7.83 7.73 7.72 7.51 7.50 7.49 7.49 7.42 7.42 7.41 7.36 7.35 7.33 6.35 6.34
4.35 4.34 4.32 4.31 4.30 4.29 4.29 4.28 4.27 4.27 4.27 4.26 4.25 4.24 4.23 4.22
1.47 1.46 1.29 1.28
0.92 1.85 0.90 1.02 1.03 1.05 1.96 5.93 5.90
f1 (ppm)

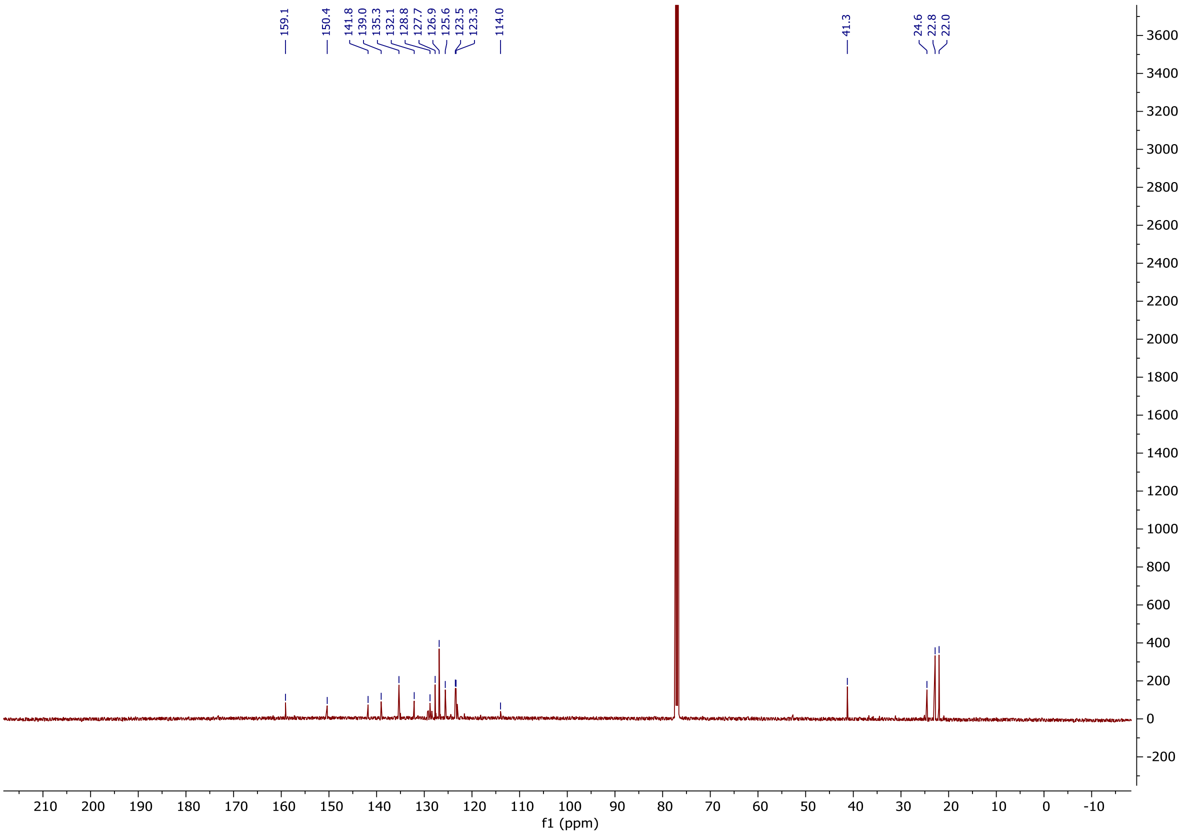
159.1
150.4
141.8 139.0 135.3 132.1 128.8 127.7 126.9 125.6 123.5 123.3
114.0
41.3
24.6 22.8 22.0
f1 (ppm)

S9

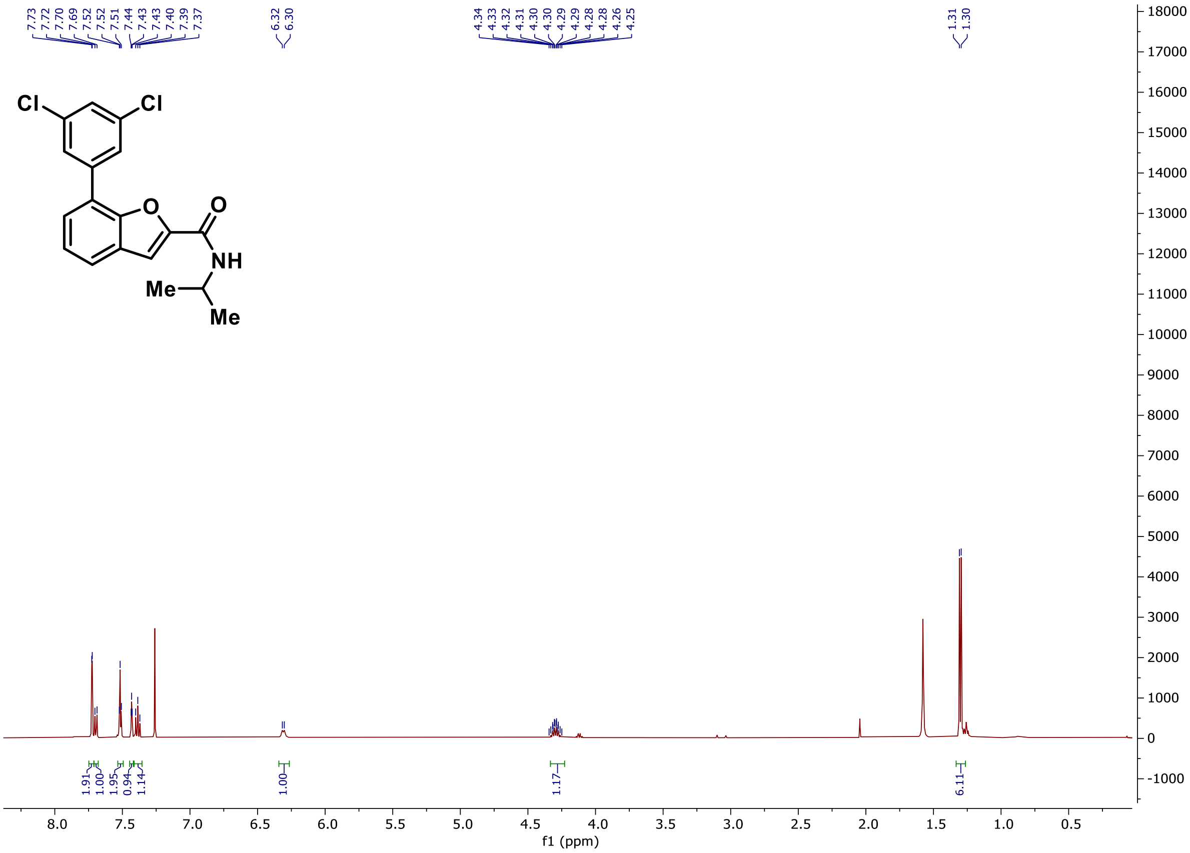

7.73
7.72
7.70
7.69
7.52
7.52
7.51
7.44
7.43
7.43
7.40
7.39
7.37
6.32
6.30
4.34
4.33
4.32
4.31
4.30
4.30
4.29
4.29
4.28
4.28
4.26
4.25
1.31
1.30
Cl
Cl
O
O
NH
Me
Me
1.91
1.00
1.95
0.94
1.14
1.00
1.17
6.11
f1 (ppm)

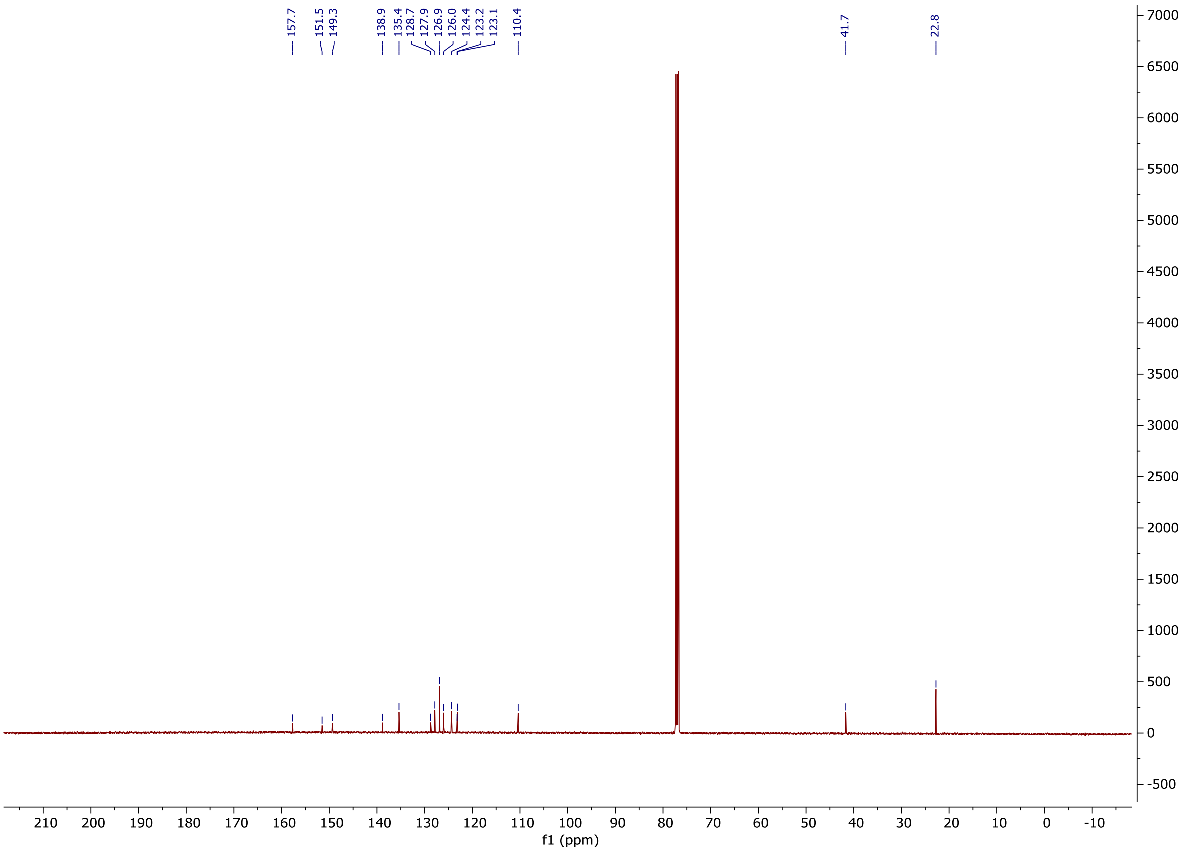

157.7
151.5
149.3
138.9
135.4
128.7
127.9
126.9
126.0
124.4
123.2
123.1
110.4
41.7
22.8
f1 (ppm)
